# N‐Heterocyclic Iod(az)olium Salts – Potent Halogen‐Bond Donors in Organocatalysis

**DOI:** 10.1002/chem.202101961

**Published:** 2021-08-05

**Authors:** Andreas Boelke, Thomas J. Kuczmera, Enno Lork, Boris J. Nachtsheim

**Affiliations:** ^1^ Institut für Organische und Analytische Chemie Universität Bremen Leobener Straße NW2C 28359 Bremen Germany; ^2^ Institut für Anorganische Chemie und Kristallographie Universität Bremen Leobener Straße NW2C 28359 Bremen Germany

**Keywords:** cyclic iodonium salts, halogen bonding, hypervalent iodine, N-heterocycles, organocatalysis

## Abstract

This article describes the application of N‐heterocyclic iod(az)olium salts (NHISs) as highly reactive organocatalysts. A variety of mono‐ and dicationic NHISs are described and utilized as potent XB‐donors in halogen‐bond catalysis. They were benchmarked in seven diverse test reactions in which the activation of carbon‐ and metal‐chloride bonds as well as carbonyl and nitro groups was achieved. *N*‐methylated dicationic NHISs rendered the highest reactivity in all investigated catalytic applications with reactivities even higher than all previously described monodentate XB‐donors based on iodine(I) and (III) and the strong Lewis acid BF_3_.

## Graphical abstract

**N‐Heterocyclic iod(az)olium** salts are introduced as highly potent halogen bonding (XB) organocatalysts reactivity in diverse test reactions based on the activation of halides and neutral species. Especially, *N*‐methylated, dicationic derivatives outperformed all known monodentate iodine(I) and (III) XB‐donors as well as the strong Lewis acid BF_3_.

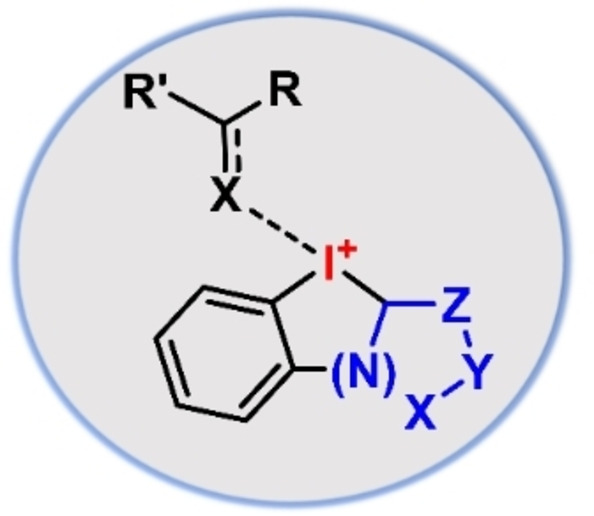

Halogen bonding (XB) is a non‐covalent interaction between an electrophilic halogen donor (XB‐donor) and a Lewis basic acceptor (XB‐acceptor). Halogen bonds are important intermolecular interactions which find widespread applications in crystal engineering, functional materials and in molecular recognition.[^1^, ^2^, ^3^, ^4^, ^5^, ^6^, ^7^, ^8^, ^9^, ^10^] In recent years XB was found to be an innovative concept in organic synthesis and in this regard XB‐donors have been established as versatile catalysts.[^11^, ^12^, ^13^, ^14^] The vast majority of XB‐donors are based on monodentate iodine(I) derivatives with either a polyfluorinated or a N‐heterocyclic backbone, for example, the triazolium derivative **1** or the pyridinium derivative **2** (Figure 1a), with cationic species being typically more reactive than neutral derivatives.[^15^, ^16^, ^17^, ^18^, ^19^, ^20^, ^21^, ^22^, ^23^] Bidentate XB‐donors such as imidazolium **3** have been described as well and were found to have a significantly higher reactivity than monodentate analogues.^[24]^

**Figure 1:**
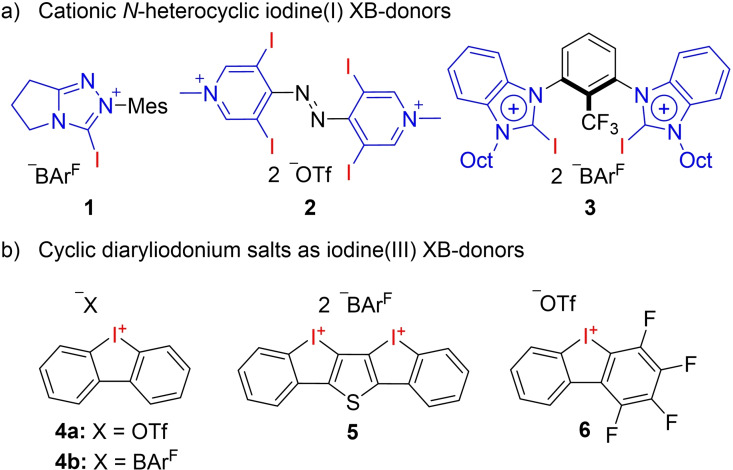
Selected examples of XB‐donors based on iodine(I) with cationic N‐heterocyclic backbones (a) and iodine(III) (b).

Besides iodine(I) species, hypervalent iodine(III) derivatives, in the form of (cyclic) diaryliodonium salts, received growing attention as XB‐donors, due to the high Lewis acidity of the hypervalent iodine atom.^[25]^ This Lewis acidity was severely investigated in a plethora of theoretical[^26^, ^27^, ^28^] and experimental studies.^[29]^ After an initial report in 2015 by Han and Liu about the use of diaryliodonium salts as catalysts for a solvent‐free Mannich reaction,^[30]^ Huber and co‐workers investigated iodolium salts **4** (Figure 1b) in the Ritter‐type solvolysis of benzhydryl chloride and [4+2] cycloadditions.^[31]^ Further XB‐mediated halide abstractions for the initiation of a cationic polymerization^[32]^ or for the activation of a metal halogen bond followed.^[33]^

Although these iodolium salts only act as monodentate XB‐donors, their performance in most reactions is comparable to bidentate iodine(I) derivatives, underlining their high potential. Thiophene‐based bidentate iodolium salt **5** as well as perfluorinated iodolium salt **6** were recently described by Huber and co‐workers, which so far show the highest reactivity among all literature‐described iodine(I) and iodine(III) XB‐donors.[^34^, ^35^]

In our group, we are strongly interested in the influence of N‐heterocyclic substituents on the chemical properties of hypervalent iodine compounds, in particular aryl‐λ^3^‐iodanes.[^36^, ^37^, ^38^, ^39^, ^40^, ^41^] We focus our investigations on the stability and the reactivity of these so far underrepresented reagents, always in direct comparison to well‐established non‐stabilized or *O*‐stabilized derivatives. In this regard, we recently introduced N‐heterocycle‐stabilized iodanes (NHIs) and found a remarkable reactivity of these reagents which outcompetes well known iodanes in a plethora of oxidative couplings ‐ even in catalytic applications (Figure 2a).[^42^, ^43^] We also developed chiral N‐heterocycle‐substituted iodoarenes as chiral iodane precursors and applied them in a plethora of highly enantioselective couplings (Figure 2b).[^44^, ^45^] Very recently, we were able to introduce iodolopyrazolium salts as a unique class of N‐heterocycle‐based iodolium salts.^[46]^ In our initial report, we demonstrated their potential reactivity as XB‐donors. Inspired by this initial, although only moderate activity, in XB catalysis, we herein want to use this structural motif and present the first systematic investigation of N‐heterocyclic iod(az)olium salts (NHISs) in general and their successful application as highly reactive XB‐donors.^[47]^

**Figure 2:**
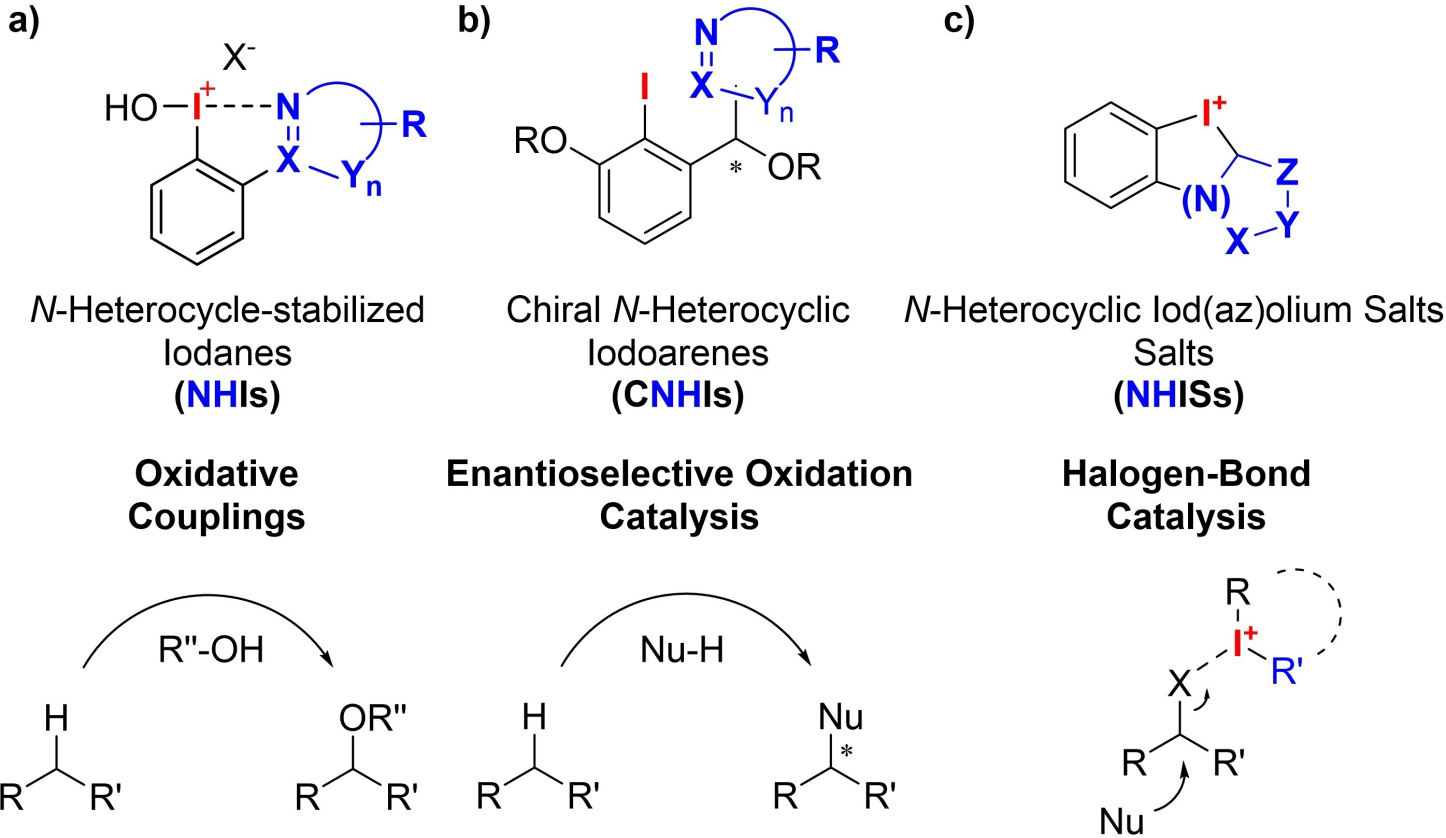
N‐Heterocycle‐substituted iodanes and aryl iodides, and their applications.

In our initial report, we described iodolopyrazolium triflates **7 a** and **7 b**. Even though these compounds could be isolated in high yields following a fast and robust synthesis, we were not able to receive structural information which would be important for a further rational variation with the goal to improve their XB‐donor properties. We were meanwhile able to obtain single crystals of the chloride salt of **7 a** (**7 c**) directly from the reaction media. As the most relevant structural information, X‐ray analysis revealed a significantly shorter I−Cl bond length *para* to the pyrazole of 2.965 Å (79 % of the sum of the *vdW* radii^[48]^) than *para* to the phenyl core of 3.015 Å (81 % of *vdW*), indicating this to be the more active site for halogen‐bonding interactions (Figure 3). Thus, enhancing the electron deficiency of the N‐heterocycle should increase the reactivity of the iodolium salt as a XB‐donor.

**Figure 3:**
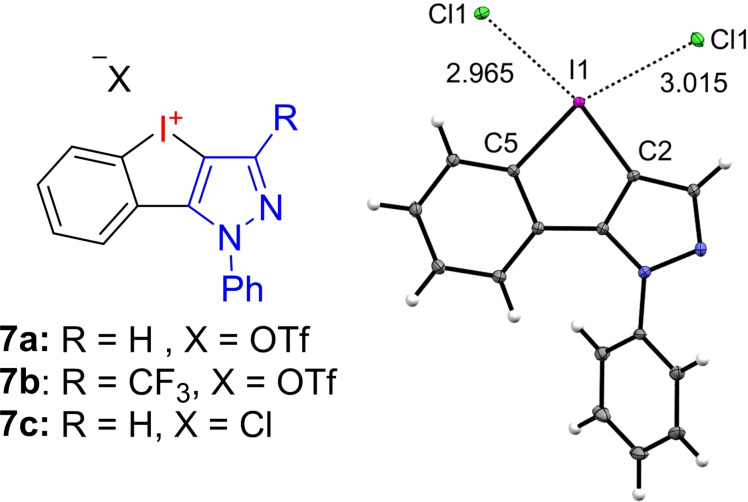
Previously synthesized iodolopyrazolium salts **7 a+b** and crystal structure (ORTEP drawing) of **7 c** (CCDC 2082275) including the coordination of a second chloride to exemplify differences in the I−Cl bond length. Thermal ellipsoids displayed with 50 % probability. Selected bond length: I1−C5: 2.135 Å; I1−C2: 2.063 Å; I1−Cl1: 2.965 Å; I1−Cl1: 3.015 Å. Selected bond angles: C2−I1−C5: 80.28°; C5−I1−Cl1: 161.48°; C2−I1−Cl1: 168.47°.

In our previous investigations we elaborated, that diazole‐substituted iodanes show an excellent relationship between reactivity and stability.[^42^, ^43^, ^49^] We therefore started our systematic search for better XB‐donors through the synthesis of different diazole‐containing NHISs. Beside the previously described iodolopyrazolium triflates **7 a+b** we generated novel NHISs (triflates and BAr^F^ salts) based on *C*‐ and *N‐*bound pyrazoles **7 d**+**8 a**–**b**, imidazoles **9**+**10 a** and imidazopyridine **11 a** (Figure 4a).

**Figure 4:**
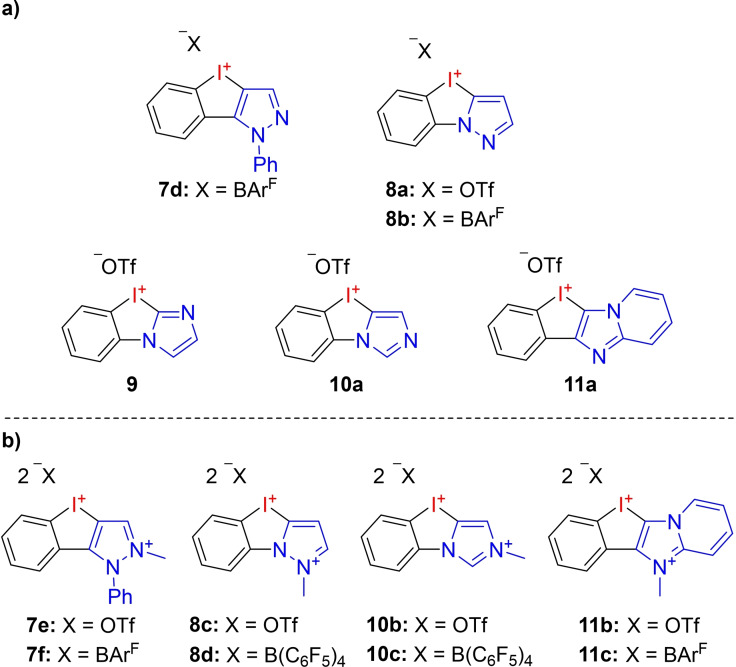
Investigated mono‐ (a) and dicationic (b) N‐heterocyclic iod(az)olium salts (NHISs).

Beside those monocationic species, we further wanted to decrease the electron density at the N‐heterocycle to strengthen the XB‐donor capability of the hypervalent iodine atom through an increased electron pull initiated by a charged N‐heterocycle. This was achieved by the synthesis of the respective *N*‐methylated, dicationic derivatives **7 e**–**f**, **8 c**–**d**, **10 b**–**c**, and **11 b**–**c** (Figure 4b).

These structurally diverse NHISs were initially tested on the well‐established Ritter‐type solvolysis of benzhydryl chloride **12** to acetamide **13** as a typical benchmark reaction for XB‐donors.[^31^, ^50^, ^51^, ^52^] The reaction was performed in wet acetonitrile, but at a lower concentration than previously described, to minimize influences of precipitating iodolium chlorides and to make the reaction even more challenging (Figure 5).[^31^, ^46^] Under these modified conditions iodolium triflate **4 a**, as a literature known standard, gave 42 % yield after 72 h of the amide **13**, while iodolopyrazolium triflate **7 a** yielded 72 %, indicating a two times higher initial rate constant (*k_rel_*). The exchange of the counterion to BAr^F^ (**7 d**) had no significant effect on the reaction rate.

**Figure 5:**
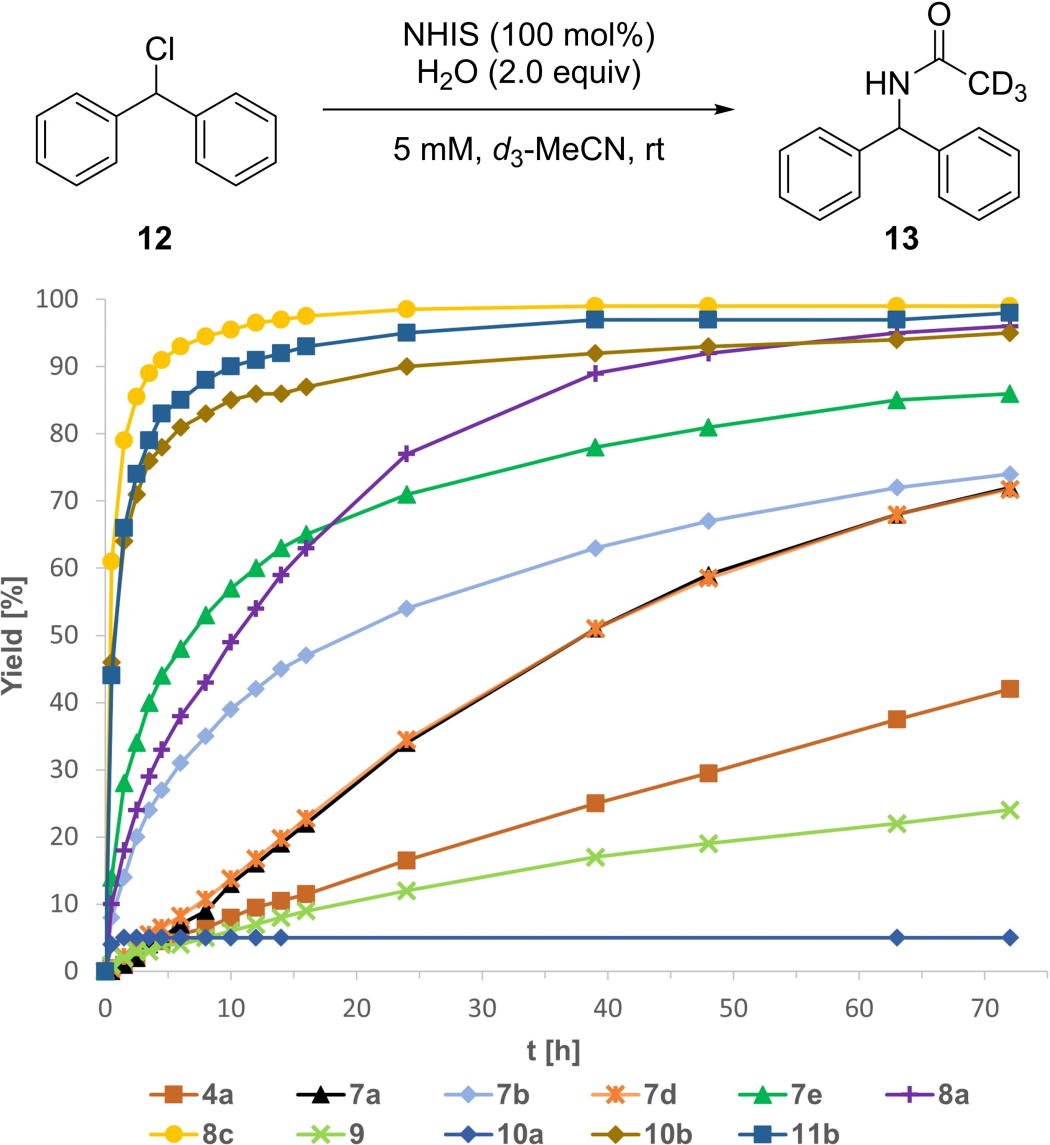
Yield‐vs.‐time profile for the XB‐mediated Ritter‐type solvolysis of benzhydryl chloride (**12**) over the course of 72 h employing stoichiometric amounts NHISs as potential activators in wet CD_3_CN. Yields determined via ^1^H NMR spectroscopy.

The strong electron‐withdrawing CF_3_‐substituted derivative **7 b** drastically increased the initial conversion rate with a seven times higher *k_rel_* value compared to the unsubstituted pyrazole **7 a**, yielding acetamide **13** in 54 % yield after only 24 h. Next, we investigated the *N*‐bound pyrazole **8 a** which performed better than the *C*‐bound derivative **7 a**, giving **13** in 77 % after 24 h and nearly quantitative yield (96 %) after three days. In contrast, imidazoles **9** and **10 a** yielded only low amounts of **13** with 24 % and 5 %, respectively. Instead, benzhydrol was formed in significant amounts. The imidazopyridine **11 a** appeared to be insoluble in acetonitrile and therefore did not promote the desired reaction.

We then investigated the dicationic *N*‐methylated derivatives **7 e**, **8 c**, **10 b** and **11 b**. Although the *C*‐bound *N*‐Me‐pyrazole **7 e** showed a higher initial reactivity, the overall performance was lower compared to the unsubstituted *N*‐bound pyrazole **8 a** with 86 % yield after 72 h. In comparison, the three other *N*‐Me species showed a significantly higher reactivity. In contrast to the low performance of the imidazole **10 a** and the incompatibility of imidazopyridine **11 a**, *N*‐Me imidazole **10 b** appeared to be among the best XB‐donors giving **13** in 90 % yield after only 24 h. The *N*‐Me imidazopyridine **11 b** was even more reactive (95 % after 24 h). However, following the already high reactivity of the unsubstituted pyrazole **8 a**, the *N*‐Me derivative **8 c** outperformed all other XB‐donors with 90 % yield after only 4 h and nearly quantitative yield (99 %) after 24 h, indicating a 174 times higher *k_rel_* value compared to iodolium triflate **4 a**, as one of the hitherto best XB‐donors tested in this transformation so far. It is worth mentioning, that the application of only 50 mol % of XB‐donor **8 c** still gave **13** in 78 % yield after 72 h (see Supporting Information ‐ Figure S3).

Following the high performance of our N‐heterocyclic iod(az)olium salts in the Ritter‐type solvolysis of benzhydryl chloride (**12**), we were eager to test the much more challenging activation of α‐methylbenzyl chloride (**14**), of which only the easier bromide variant has been studied.^[35]^ In this reaction no conversion of the starting material **14** was observed using **4 a**, whereas with the best non‐methylated derivative, pyrazole **8 a**, 18 % of the acetamide **15** was obtained after three days (Figure 6). With 23 % yield after 72 h *C*‐bound *N*‐Me pyrazole **7 e** only performed slightly better. In contrast, *N*‐Me imidazole **10 b** gave 79 % within a similar time span. For the more reactive *N*‐Me imidazopyridine **11 b** nearly the same yield (77 %) was already observed after only 24 h. *N*‐methyl pyrazole **8 c** again proved to be top of its class with 92 % conversion after 24 h and full conversion (99 % yield) after 66 h. Compared to the non‐methylated derivative **8 a**, **8 c** showed an approx. 35 times higher initial rate constant (*k_rel_* =34.5), underlining its performance as the best reported XB‐donor for these types of halide abstractions to date.

**Figure 6:**
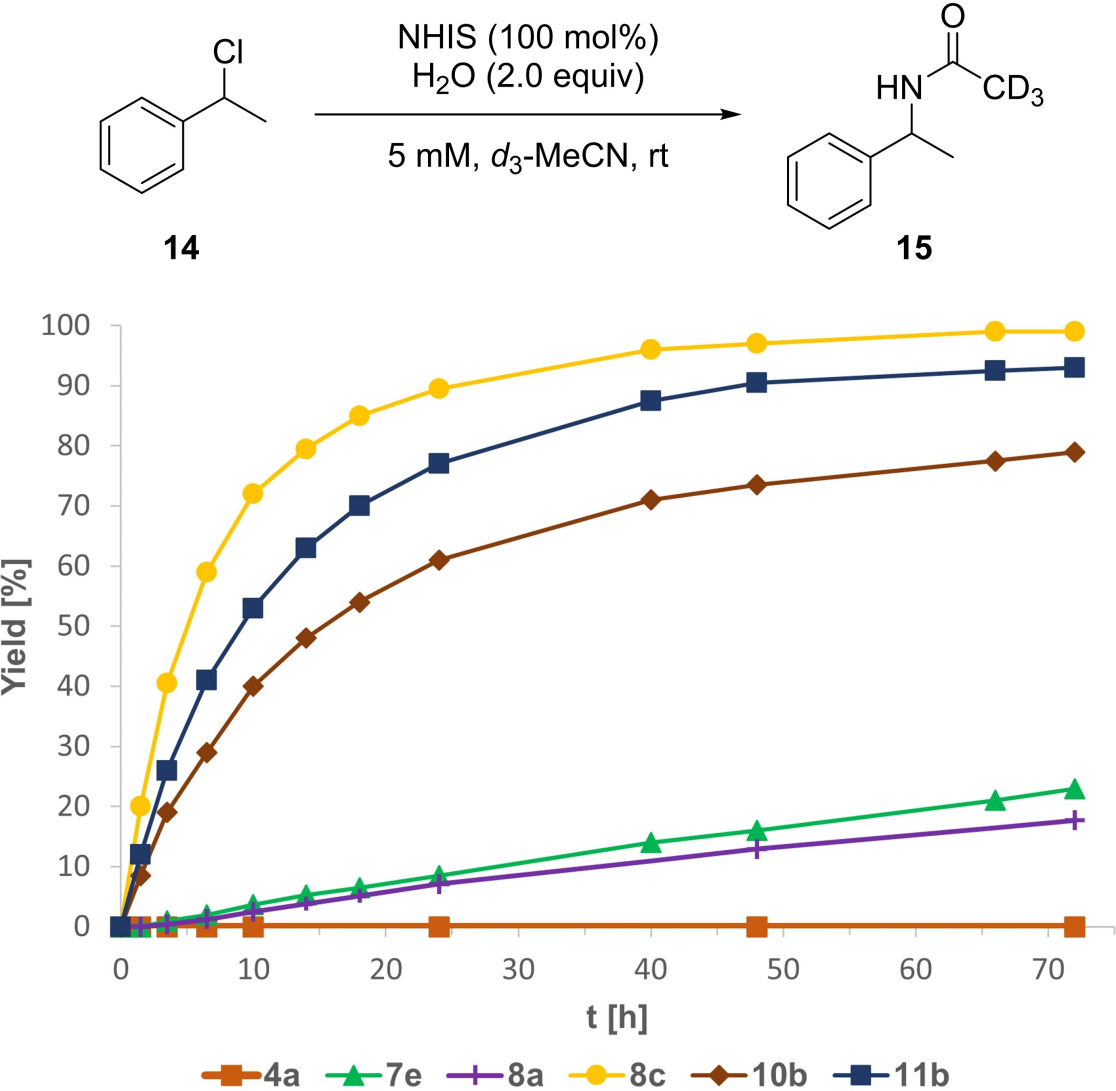
Yield‐vs.‐time profile for the XB‐mediated Ritter‐type solvolysis of α‐methylbenzyl chloride (**14**) over the course of 72 h employing stoichiometric amounts of NHISs as potential activators in wet CD_3_CN. Yields determined via ^1^H NMR spectroscopy.

Following their outstanding performance in the activation of C−Cl bonds, the activation of a metal‐Cl bond was investigated in the gold(I)‐catalyzed cyclization of propargylic amide **17** to oxazoline **18** (Figure 7). To overcome solubility issues in this reaction and since the corresponding tetrakis(3,5‐bistrifluoromethyl)borate (BAr^F^) derivatives proved to be more reactive in previous reports,^[33]^ NHISs **7 f**, **8 b+d**, **10 c** and **11 d** were prepared by anion exchange for further investigations. *N*‐Methyl pyrazole **8 c** and imidazole **10 b** proved to be troublesome. By abstracting a 3,5‐bis(trifluoromethyl)phenyl group from the borate and subsequent ring opening of the iodazole core, the formation of acyclic iodonium salts was observed as a major side reaction. After prolonged reaction time iodonium salt **16** was obtained in 74 % yield (Scheme 1). In contrast, *C*‐bound derivatives **7 d** and **11 b** showed a significantly lower affinity for this undesired side reaction and were successfully isolated as the corresponding BAr^F^ salts **7 f** and **11 c**. Switching the anion from BAr^F^ to tetrakis(pentafluorophenyl)borate finally solved the issue for the *N*‐bound derivatives **8 d** and **10 c**.

**Figure 7:**
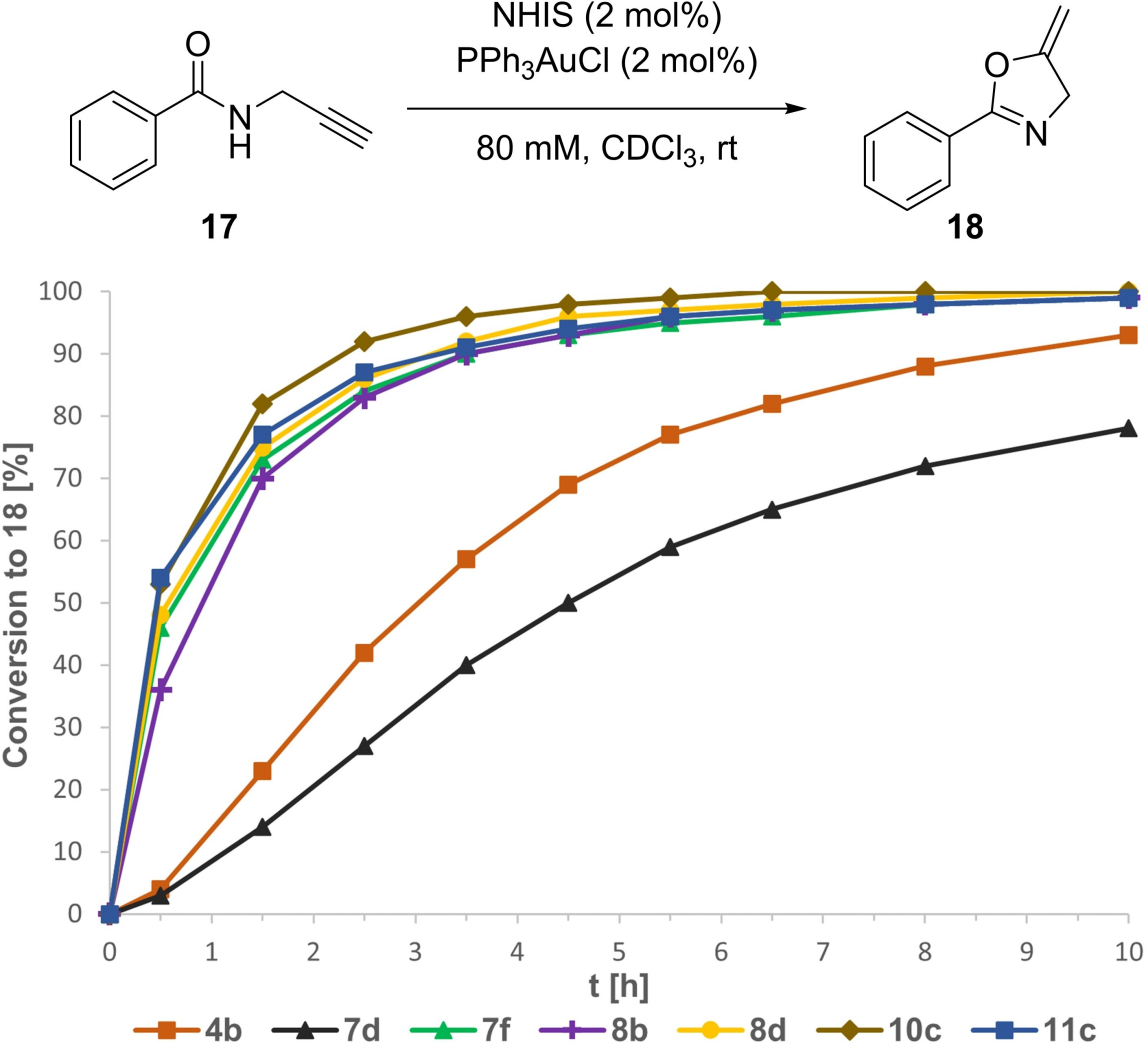
Conversion‐vs.‐time profile for the gold(I)‐catalyzed cyclization of propargylic amide **17** in the presence of NHISs as the activators. Yields determined via ^1^H NMR spectroscopy with SiEt_4_ as the internal standard.

**Scheme 1:**
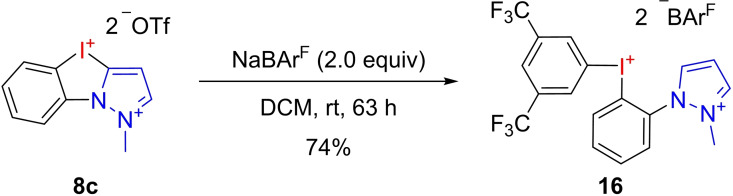
Ligand exchange and ring opening towards acyclic iodonium salt **16** as the major side reaction during anion exchange.

With the BAr^F^ salts in hand, the activation of the gold(I)‐catalyst was investigated. In contrast to the previous results, iodolopyrazole **7 b** showed a lower reactivity with 78 % conversion after 10 h (*k_rel_* =0.6) compared to iodolium salt **4 b** giving **18** in 93 % conversion after the same time span (Figure 7). Again, a much higher reactivity was observed for the *N*‐bound pyrazole **8 b** with 90 % conversion after 3.5 h. All *N*‐Me derivatives **7 f**, **8 d**, **10 c** and **11 c** showed only a slightly better performance than **8 b**, presumably as an indicator for a “close to maximum” conversion rate. Imidazole **10 c** was determined to be the most efficient activator with 92 % conversion of amide **17** after 2.5 h and full conversion after around 6 h, with a four times higher initial rate constant than iodolium salt **4 b**.

We turned our focus towards the Diels‐Alder cycloaddition of cyclopentadiene (**19**) and methyl vinyl ketone (**20**) as another benchmark reaction.[^17^, ^53^] Here, iodolium salt **4 b** has been previously investigated.^[31]^ We performed a slight adaption of the initial reaction conditions by reducing the amount of cyclopentadiene **19** to only 4 equiv. Under these conditions the blank reaction was neglectable after the investigated reaction end point of 3 h (Figure 8). Iodolium salt **4 b** provided 13 % yield (*k_rel_* =3.5), while iodolopyrazole **7 b** already gave **21** in 26 % yield (*k_rel_* =8). *N*‐Bound pyrazole **8 b** again showed a significantly enhanced reactivity with 83 % yield after 3 h (*k_rel_* =47). A better performance was observed for *C*‐bound *N*‐Me pyrazole **7 f** with a full conversion after 2.5 h (*k_rel_* =91). *N*‐Methyl imidazopyridine **11 c** was by far the most efficient XB‐donor in this transformation with a full conversion after only 10 min reaction time.

**Figure 8:**
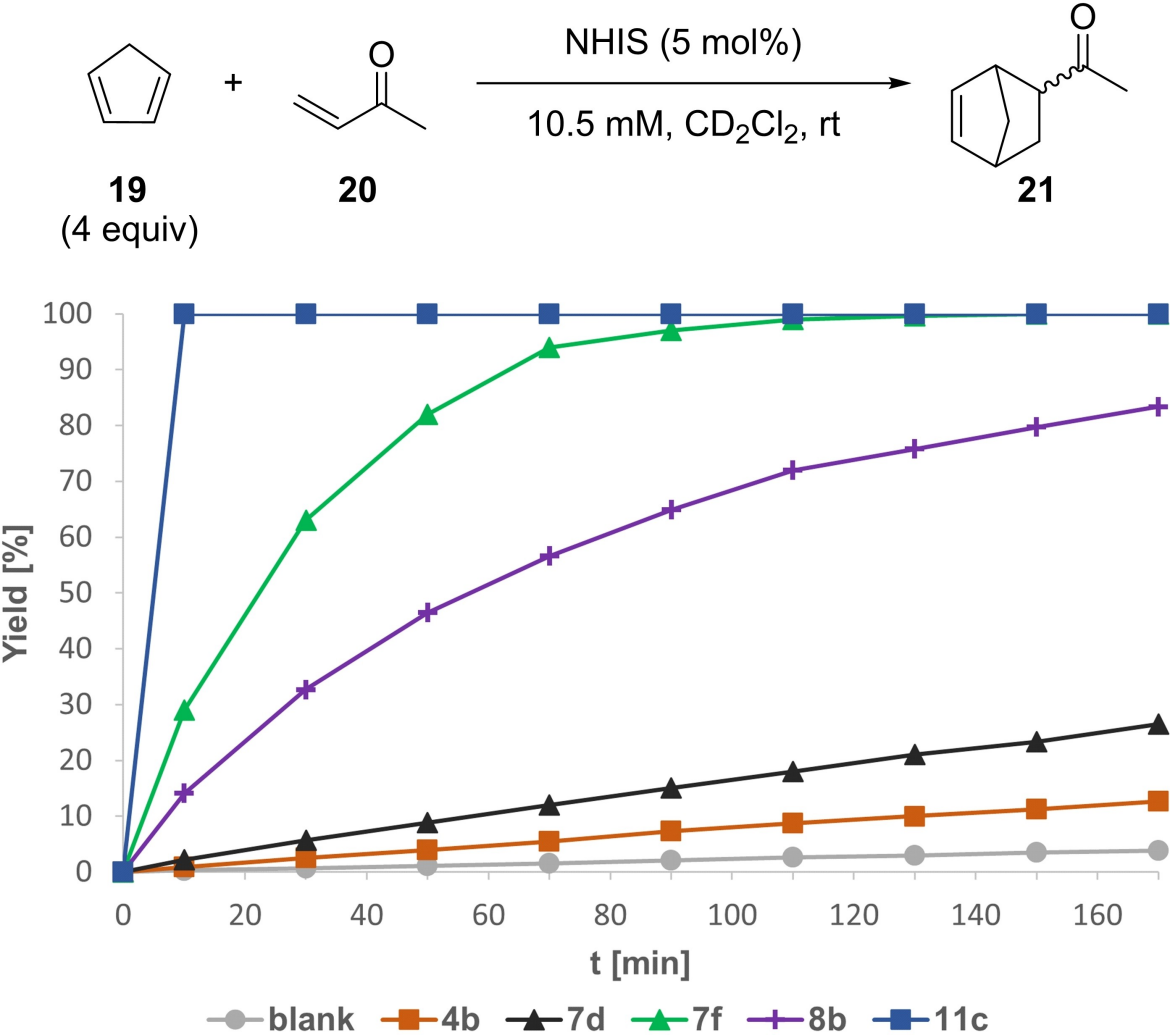
Yield‐vs.‐time profile for the XB‐mediated Diels‐Alder reaction between cyclopentadiene (**19**) and MVK (**20**) over the course of 170 min employing several NHISs as potential activators in CD_2_Cl_2_. Yields determined via ^1^H NMR spectroscopy using SiEt_4_ as the internal standard.

Next, we investigated the more challenging Diels‐Alder reaction between cyclohexadiene (**22**) and MVK (**20**), in which the bidentate iodolium salts **5** was reported to be the so far only active XB‐donor and nearly approached the activity of the strong Lewis acid BF_3_.^[34]^ In contrast to the literature report, we started the investigation with a catalyst loading of 15 mol % instead of 30 mol %. Under these conditions, pyrazole **8 b** showed a low reactivity with only 2 % yield after 12 h. In comparison, all *N*‐Me species **7 f**, **8 d**, **10 c** and **11 c** showed high and nearly equal performances giving **23** in around 80–85 % yield after 12 h. Furthermore, they even showed a slightly higher initial conversion rate than BF_3_ etherate, although just falling short in overall reactivity (see Supporting Information ‐ Figure S15). To our delight, it then became apparent that the catalyst loading for the *N*‐Me derivatives could be reduced to 5 mol % without significant loss in reactivity with yields between 76–83 % after 12 h (Figure 9). Especially imidazopyridine **11 c** showed an outstanding reactivity with 40 % yield after just 0.5 h and 83 % after 12 h. Furthermore, at this catalyst loading even BF_3_ etherate was outperformed (70 % after 12 h), indicating imidazopyridine **11 c** to be the most active XB‐donor for this transformation described so far. Additionally, even at only 2.5 mol % catalyst loading, **11 c** still gave **23** in 66 % yield after 12 h. At last, a stability test with this catalyst was conducted due to the described slow side reaction with the BAr^F^ counterion during anion exchange. For this, an additional equivalent of both starting materials was added after 10 h, revealing nearly the same conversion profile in the second run as in the first one (see Supporting Information Figure S17+S18), proving the possible side reaction to be of minor influence. The acyclic iodonium salt **16** showed a completely different conversion profile with a lower overall activity.

**Figure 9:**
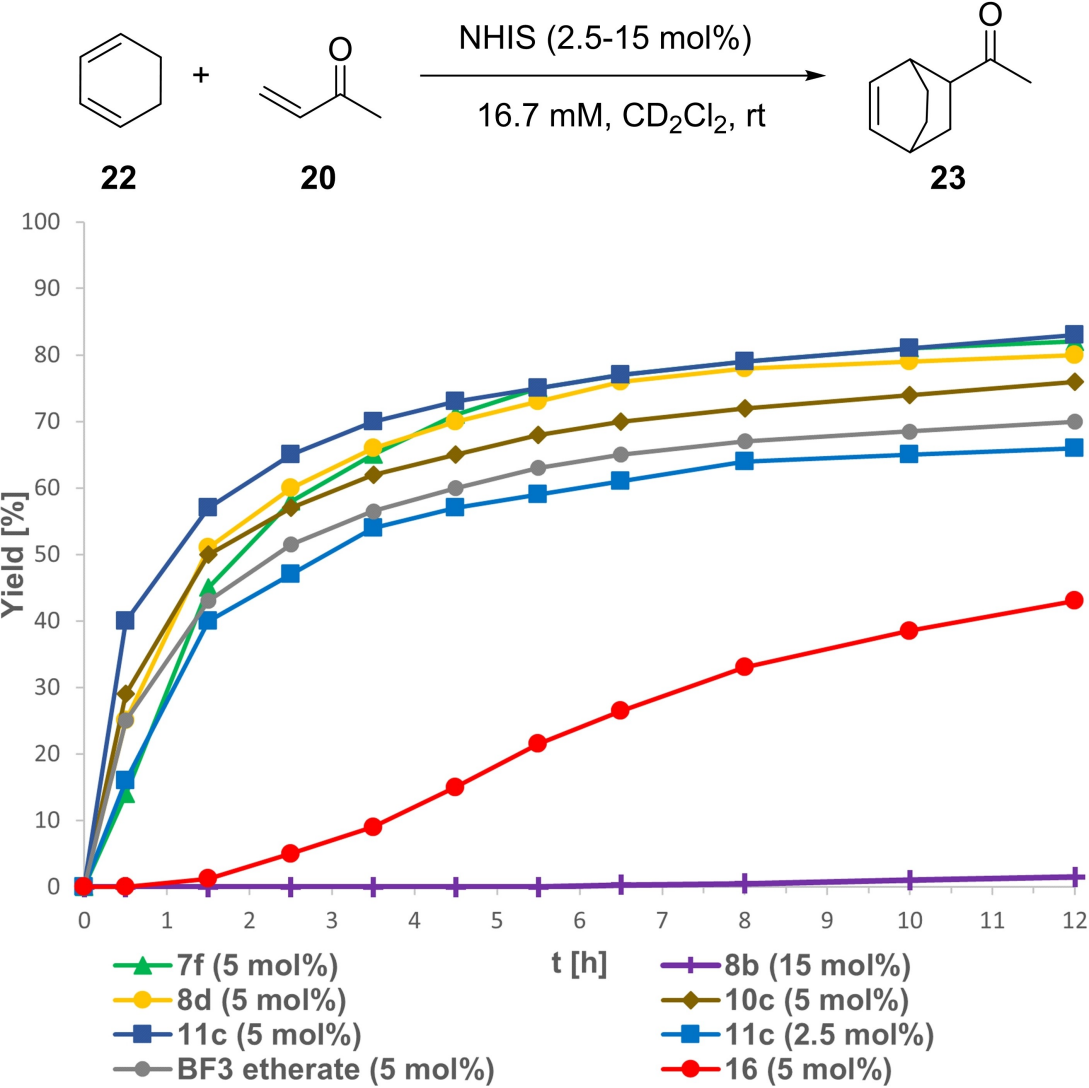
Yield‐vs.‐time profile for the XB‐mediated Diels‐Alder reaction between cyclohexadiene (**22**) and MVK (**20**) over the course of 12 h employing NHISs as potential activators in CD_2_Cl_2_. Yields determined via ^1^H NMR spectroscopy using SiEt_4_ as the internal standard.

As another carbonyl‐activating benchmark reaction, the Michael addition between 1‐methylindole (**24**) and *trans*‐β‐crotonophenone (**25**) to **26** was investigated.[^24^, ^34^, ^54^, ^55^] Due to the high performance in previous reactions, we started our investigation with only 5 mol % catalyst loading. As reported for **4 b**, both non‐methylated pyrazoles **7 d** and **8 b** showed no catalytic activity (Figure 10), whereas *N*‐Me pyrazole **7 e** gave **26** in 52 % yield after almost 5 h. Similar to their high performance in the Ritter‐type reactions **8 d**, **10 c** and **11 c** showed outstanding reactivity with close to quantitative yields after 1–1.5 h. We further decreased the catalyst loading of **8 d** and **11 c** to 1 mol % and were delighted to observe a high reactivity for both XB‐donors as well, with **8 d** clearly outperforming **11 c** with about 95 % yield of **26** after only 4 h. In comparison with the reported results for the bidentate iodolium salt **5** (62 % after 12 h^[34]^) and recently developed cyclic bromonium salts,^[56]^ this further implies pyrazole **8 d** to be the most potent organic XB‐donor for this transformation described so far.

**Figure 10:**
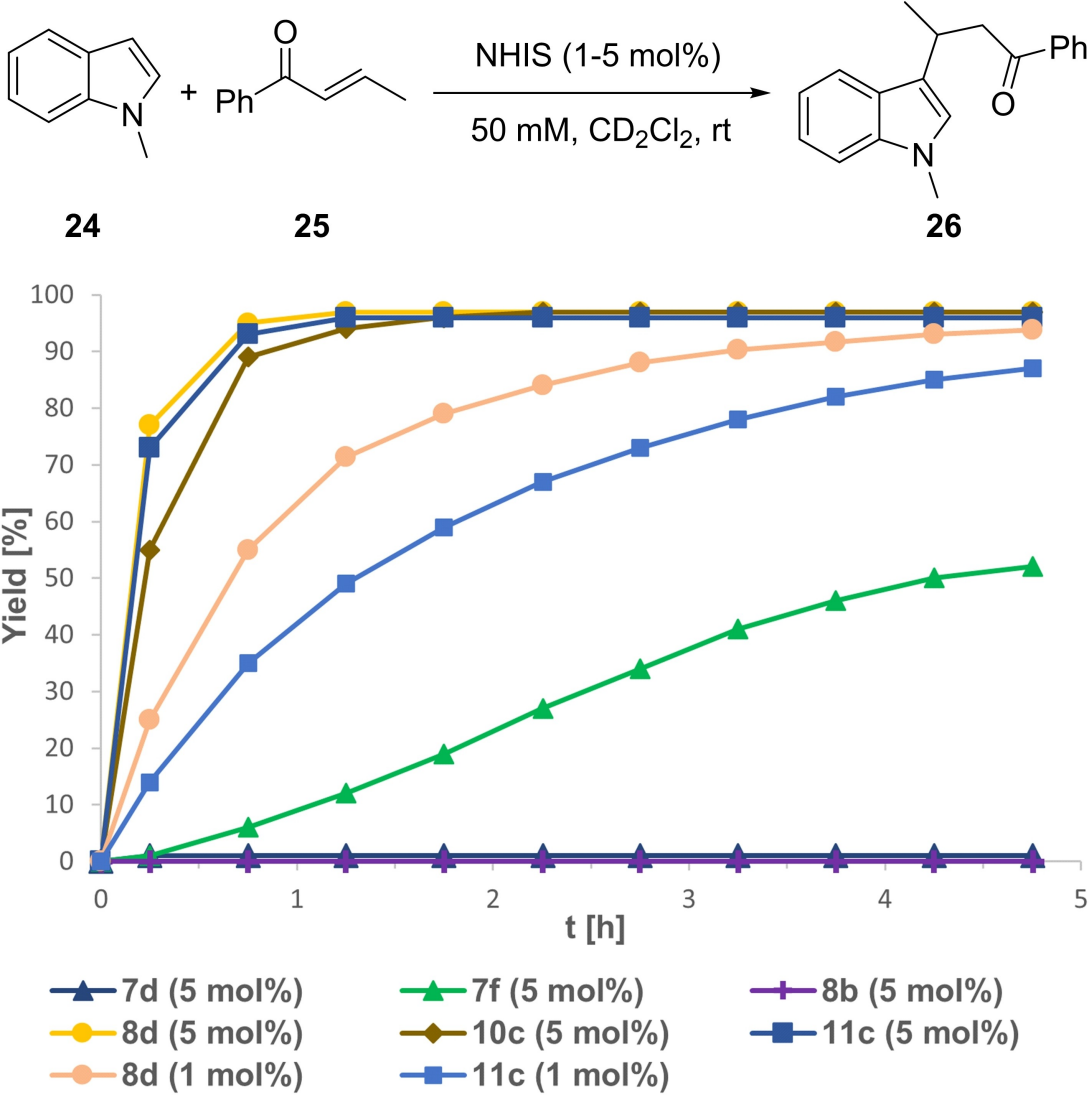
Yield‐vs.‐time profile for the XB‐mediated Michael reaction between 1‐methylindole (**24**) and (**25**) over the course of 4.75 h employing NHISs in CD_2_Cl_2_. Yields determined via ^1^H NMR spectroscopy using SiEt_4_ as the internal standard.

Finally, we investigated the nitro‐Michael addition between 5‐methoxyindole (**27**) and nitrostyrene **28**.[^24^, ^34^] With 10 mol % catalyst loading, pyrazole **7 d** was nearly inactive as XB‐donor (Figure 11), whereas *N*‐Bound pyrazole **8 b** yielded **28** in 26 % after 4 h and 61 % after 24 h. When turning to the *N*‐Me derivatives **7 f**, **8 d**, **10 c** and **11 c**, a problem was encountered. The otherwise highly reactive XB‐donors **8 d**, **10 c** and **11 c** showed only low performances with 19–29 % yield of **28** after 4.5 h. Further investigations revealed them to be incompatible with 5‐methoxyindole (**27**). Under these conditions a plethora of different reaction and decomposition products were observed when mixing catalysts and indole **27** (see Supporting Information – Figure S27), which limits their applicability. Only the least reactive *N*‐Me pyrazole **7 f**, showed satisfying levels of activity with 61 % yield after 4.5 h with 10 mol % catalyst loading and still 50 % yield at 5 mol %. For the non‐methylated iodonium salts **7 d** and **8 b**, no undesired side reactions were observed. Because of this, although pyrazole **7 f** showed the highest performance, *N*‐bound pyrazole **8 b** arguably possesses the best balance between reactivity, selectivity, and stability in comparison to all hitherto investigated monodentate XB‐donors.

**Figure 11:**
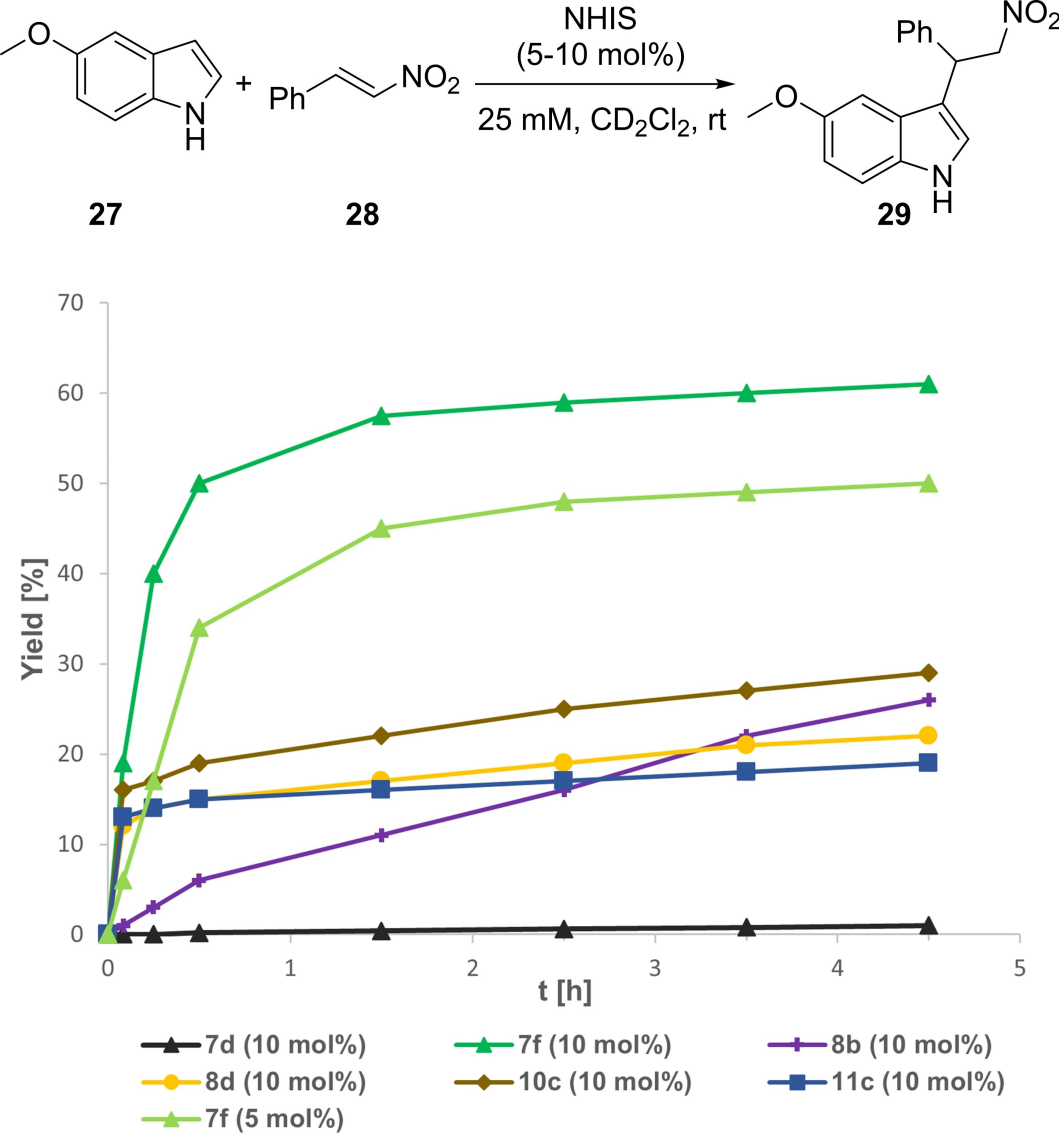
Yield‐vs.‐time profile for the XB‐mediated nitro‐Michael reaction between 5‐methoxyindole (**27**) and nitrostyrene **28** over the course of 4.5 h employing several cyclic iodonium salts as potential activators in CD_2_Cl_2_. Yields determined via ^1^H NMR spectroscopy using SiEt_4_ as the internal standard.

In conclusion, novel N‐heterocyclic iod(az)olium salts (NHISs) were synthesized and introduced as powerful XB‐donors in organocatalysis. *N*‐Methylated, dicationic derivatives showed an outstanding performance in all investigated benchmark reactions showing an even higher reactivity than all previously described monodentate organic iodine(I) and (III) donors. On top, their activation capability of unsaturated carbonyls in the Michael addition and Diels‐Alder reactions surpasses the activity of bidentate iodolium salts and the strong Lewis acid BF_3_ with catalyst loadings of only 5 mol %. Due to their straightforward synthesis, their throughout excellent performance, and their high stability, we are confident that this novel class of XB‐donors will find frequent use as organocatalysts in the near future. Further installation of chiral units should allow efficient enantioselective transformations. This further variation and their implementation into preorganized bidentate structures is under current investigation in our laboratory.

Deposition Number(s) 2082275 (for **7 c**) contains the supplementary crystallographic data for this paper. These data are provided free of charge by the joint Cambridge Crystallographic Data Centre and Fachinformationszentrum Karlsruhe Access Structures service www.ccdc.cam.ac.uk/structures.

## Conflict of interest

The authors declare no conflict of interest.

## Supporting information

As a service to our authors and readers, this journal provides supporting information supplied by the authors. Such materials are peer reviewed and may be re‐organized for online delivery, but are not copy‐edited or typeset. Technical support issues arising from supporting information (other than missing files) should be addressed to the authors.

Supporting Information
