# Supplementary material for: N‐Heterocyclic Iod(az)olium Salts – Potent Halogen‐Bond Donors in Organocatalysis
Source: Chemistry. 2021 Aug 5;27(52):13128–34. doi: 10.1002/chem.202101961 (PMC8519039; doi:10.1002/chem.202101961)
Supplement: Supplementary file 1 — Supporting Information [file CHEM-27-13128-s001.pdf]

Chemistry–A European Journal

Supporting Information

N-Heterocyclic Iod(az)olium Salts – Potent Halogen-Bond Donors in Organocatalysis

Andreas Boelke, Thomas J. Kuczmera, Enno Lork, and Boris J. Nachtsheim*

Table of Contents

1. General Information 2

2. Overview for the Synthesis of the Iod(az)olium Salts 4

3. Preparation of Precursors 5

a. (*N*-HetAr)-Aryl iodides 5

b. *N*-Methylated (*N*-HetAr)-aryl iodides 9

c. Sodium tetrakis(fluorophenyl)borates 11

4. Synthesis of iod(az)olium salts 13

5. Starting materials for the XB-mediated reactions 25

6. XB-mediated reactions 27

Ritter-type halide abstractions 28

Gold(I)-catalyzed cyclization of propargylic amide 17 32

Diels-Alder reactions 34

Michael reaction 40

Nitro-Michael reaction 42

7. References 45

8. Crystal Structure 46

9. NMR spectra 50

Precursors and iodonium salts 50

XB-mediated reactions 83

Gold(I)-catalyzed cyclization of propargylic amide 17 94

Diels-Alder-Reaction between CPD and MVK 97

Diels-Alder-Reaction between CHD and MVK (15 mol%) 100

Diels-Alder-Reaction between CHD and MVK (2.5-5 mol%) 103

Michael addition between 1-methylindole and crotonophenone 106

Nitro-Michael reaction between 5-methoxyindole and nitrostyrene 110

1. General Information

Unless otherwise stated, all reactions with moisture- or oxygen-sensitive reagents were performed using standard Schlenk techniques under a nitrogen or argon atmosphere. Reagents were used as received from their commercial supplier (abcr, Acros Organics, Alfa Aesar, Apollo Scientific, Carbolution Chemicals, Sigma Aldrich, TCI). Anhydrous dichloromethane (DCM), acetonitrile (MeCN), tetrahydrofuran (THF) and toluene were obtained from an *inert* PS-MD-6 solvent purification system. All other solvents were dried using standard methods.[1] Unless otherwise stated, all yields refer to isolated yields of compounds estimated to be >95% pure as determined by ^{1}H-NMR spectroscopy.

Thin layer chromatography was performed on fluorescence indicator marked precoated silica gel 60 plates (*Macherey-Nagel*, ALUGRAM Xtra SIL G/UV$_{254}$) and visualized by UV light (254 nm/366 nm). Flash column chromatography was performed on silica gel (0.040 – 0.063 mm) with the solvents given in the procedures.

^{1}H-, ^{13}C- and ^{19}F-NMR spectra were recorded on *Bruker Avance Neo* 600-spectrometers. Chemical shifts for ^{1}H-NMR spectra were reported as δ (parts per million) relative to the residual signal of $CHCl_3$ at 7.26 ppm (s), *d*$_3$-MeCN at 1.94 ppm (quin.), *d*$_4$-MeOH at 3.31 ppm (quin.) or *d*$_6$-DMSO at 2.50 ppm (quin.). Chemical shifts for ^{13}C-NMR spectra were reported as δ (parts per million) relative to the signal of $CDCl_3$ at 77.0 ppm (t), *d*$_4$-MeOH at 49.0 ppm (sept.) or *d*$_6$-DMSO at 39.5 ppm (sept.). ^{19}F-NMR spectra were reported as δ (parts per million) relative to $CFCl_3$ at 0.00 ppm as external standard. The following abbreviations were used to describe splitting patterns: br = broad, s = singlet, d = doublet, t = triplet, q = quartet, quin = quintet, sext. = sextet, sept = septet, m = multiplet. Coupling constants *J* are given in Hertz.

High resolution (HR) EI mass spectra were recorded on the double focussing mass spectrometer ThemoQuest MAT 95 XL from *Finnigan MAT*. HR-ESI mass spectra were recorded on a Bruker impact II. APCI mass spectra were recorded on an *Advion* Expression CMS^{L} via ASAP probe or direct inlet. EI mass spectra were obtained from an Agilent 7890B GC System with an Agilent 5977A MSD mass spectrometer. All signals were reported with the quotient from mass to charge *m/z*.

IR spectra were recorded on a *Nicolet* Thermo iS10 scientific spectrometer with a diamond ATR unit. The absorption bands were reported in cm^{-1}.

Melting points were determined on a *Büchi* M-5600 Melting Pint apparatus with a heating rate of 5 °C/min. The melting points were reported in °C. Most of the hypervalent iodine compounds underwent changes in appearance (e.g. softening) before final melting/decomposition.

Single crystals were grown from MeCN-solution. A suitable crystal was selected and measured on a Bruker D8 Venture diffractometer. The crystal was kept at 100 K during data collection. Using

Olex2,[2] the structure was solved with the ShelXT[3] structure solution program using Intrinsic Phasing and refined with the XL[4] refinement package using Least Squares minimization. The ORTEP drawing was made using the program Mercury from the CCDC.

2. Overview for the Synthesis of the Iod(az)olium Salts

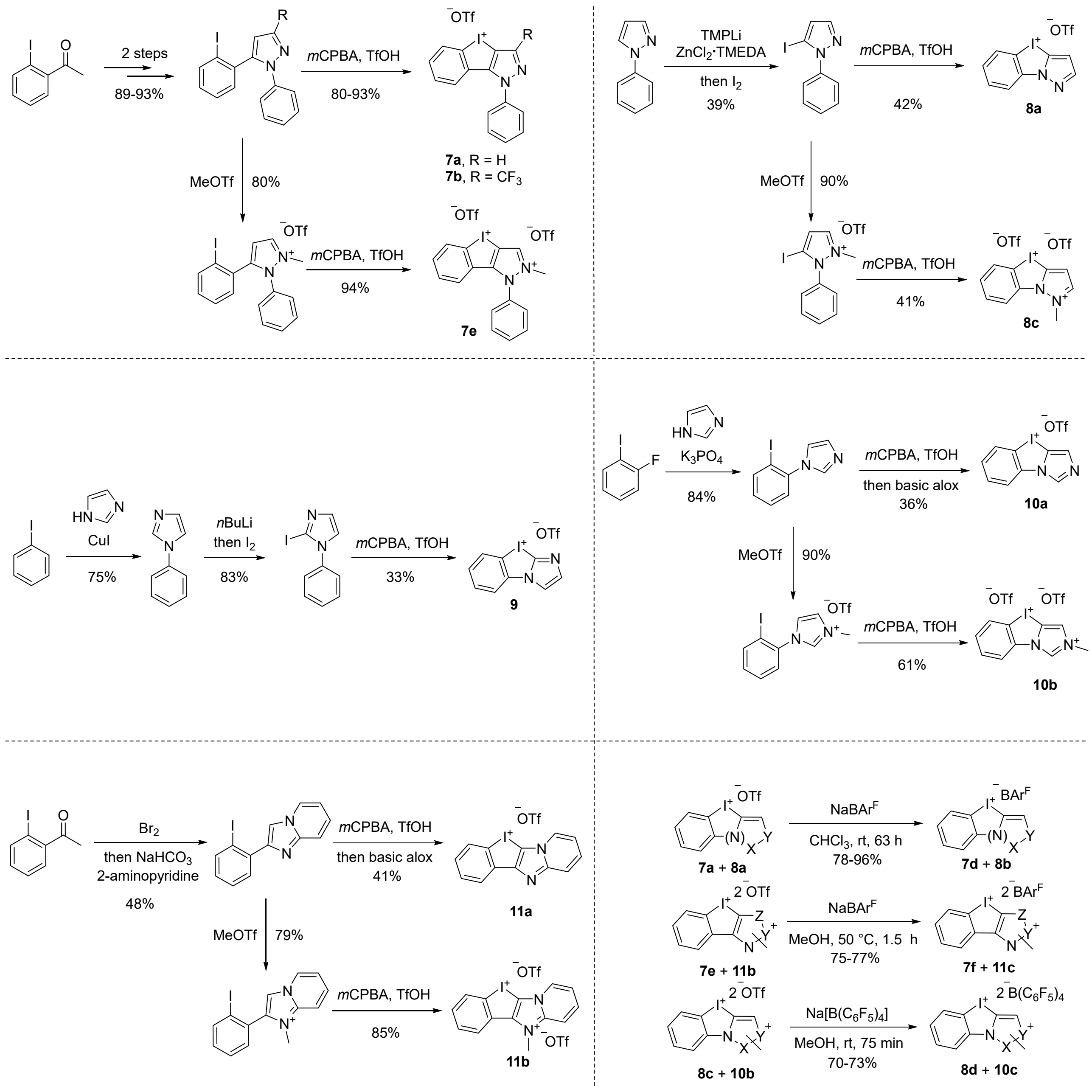

3. Preparation of Precursors

a. (*N*-HetAr)-Aryl iodides

2'-Iodoacetophenone (S2)

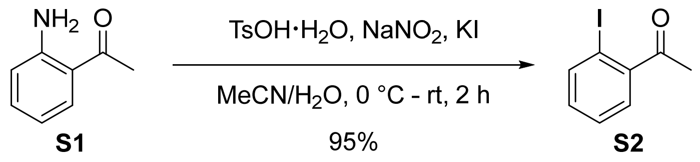

A reported literature procedure was used.[5] *p*-TsOH monohydrate (22.8 g, 120 mmol) was dissolved in MeCN (160 mL) and 2'-aminoacetophenone (**S1**, 5.41 g, 40.0 mmol) was added. The mixture was cooled to 0 °C and $NaNO_2$ (5.52 g, 80.0 mmol) in H_2O (12 mL) was added dropwise over 5 min. Afterwards KI (16.6 g, 100 mmol) in H_2O (12 mL) was added dropwise over 5 min and stirring was continued for 10 min at 0 °C and for 2 h at room temperature. H_2O (700 mL) was added, the pH was adjusted to ~9 with sat. $NaHCO_3$-sol. and sat. $Na_2S_2O_3$-sol. (80 mL) was added afterwards. The mixture was extracted with EtOAc (3 x 250 mL), the combined organic phases were washed with brine (250 mL), dried over Na_2SO_4, filtered and concentrated under reduced pressure. The residue was purified via column chromatography on silica (cyclohexane/EtOAc 10:1) to give 2'-iodoacetophenone (**S2**, 9.32 g, 37.9 mmol, 95%) as a yellow liquid.

^{1}H-NMR ($CDCl_3$, 601 MHz): δ (ppm) 7.94 (dd, *J* = 7.9, 1.1 Hz, 1H), 7.46 (dd, *J* = 7.7, 1.7 Hz, 1H), 7.41 (td, *J* = 7.5, 1.1 Hz, 1H), 7.12 (ddd, *J* = 7.9, 7.3, 1.7 Hz, 1H), 2.61 (s, 3H). ^{13}C-NMR ($CDCl_3$, 151 MHz): δ (ppm) 201.8, 144.0, 140.9, 131.8, 128.3, 128.1, 90.9, 29.5. IR (ATR): $\tilde{\nu}$ (cm^{-1}) 3058, 3000, 1691, 1580, 1460, 1421, 1353, 1279, 1240, 1013. MS (APCI) *m/z* = 247.0 $[M+H]^+$. Analytical data is in accordance with literature data.[5]

5-(2-Iodophenyl)-1-phenyl-1*H*-pyrazole (S3)

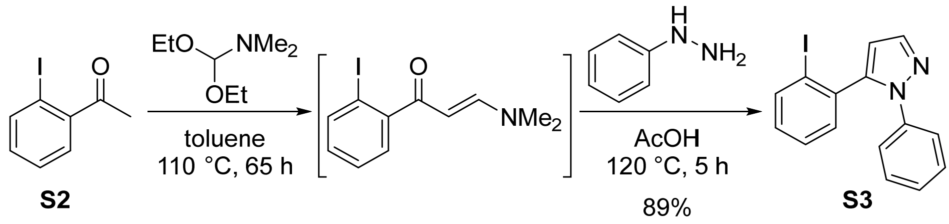

A slightly modified literature procedure was used.[6] 2'-Iodoacetophenone (**S2**, 2.46 g, 10.0 mmol) was dissolved in dry toluene (25 mL) and *N*,*N*-dimethylformamide diethyl acetal (3.43 mL, 20.0 mmol) was added. The mixture was stirred for 65 h at 110 °C, afterwards concentrated under reduced pressure and was redissolved in AcOH (40 mL). Phenylhydrazine (1.08 mL, 11.0 mmol) was added and the mixture was stirred at 120 °C for 5 h. The solvent was removed *in vacuo* and H_2O (30 mL) was added. After neutralization with sat. $NaHCO_3$-sol. the aqueous phase was extracted with EtOAc (3 x 50 mL). The combined organic phases were dried over Na_2SO_4, filtered

and concentrated under reduced pressure. The residue was purified via column chromatography on silica gel (cyclohexane/EtOAc 20:1) to give 5-(2-iodophenyl)-1-phenyl-1*H*-pyrazole (**S3**, 3.07 g, 8.87 mmol, 89%) as a light-orange solid.

^{1}H-NMR ($CDCl_3$, 601 MHz) δ (ppm) 7.87 (dd, *J* = 8.0, 1.2 Hz, 1H), 7.76 (d, *J* = 1.8 Hz, 1H), 7.33 (td, *J* = 7.5, 1.2 Hz, 1H), 7.27 – 7.26 (m, 4H), 7.25 – 7.21 (m, 2H), 7.06 (ddd, *J* = 8.0, 7.4, 1.7 Hz, 1H), 6.49 (d, *J* = 1.8 Hz, 1H). ^{13}C-NMR ($CDCl_3$, 151 MHz): δ (ppm) 144.1, 139.9, 139.8, 139.5, 136.6, 131.6, 130.2, 128.7, 128.0, 127.0, 124.2, 109.2, 100.0. IR (ATR): $\tilde{\nu}$ (cm^{-1}) 3070, 3042, 1597, 1498, 1433, 1421, 1386, 1221, 1137, 1016. MS (APCI) *m/z* = 347.1 $[M+H]^+$. Mp. 80–81 °C. Analytical data is in accordance with literature data.[6]

5-(2-Iodophenyl)-1-phenyl-3-(trifluoromethyl)-1*H*-pyrazole (S4)

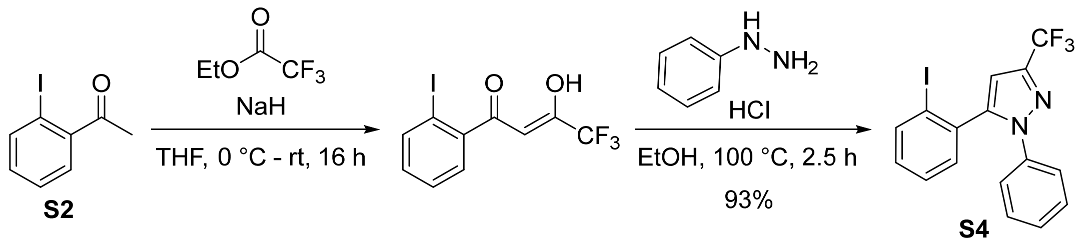

A reported literature procedure was used.[6] 2'-Iodoacetophenone (**S2**, 2.95 g, 12.0 mmol) was dissolved in dry THF (40 mL) and NaH (60% in mineral oil, 0.800 g, 20.0 mmol) was added in portions at 0 °C. After stirring for 30 min at 0 °C, ethyl trifluoroacetate (1.80 mL, 15.0 mmol) was added and the mixture was stirred at room temperature for 16 h. The reaction was quenched with H_2O (20 mL), acidified (pH 3) with 1 M HCl and extracted with EtOAc (3 x 50 mL). The combined organic phases were washed with brine (50 mL), dried over Na_2SO_4, filtered and concentrated under reduced pressure. The residue was filtered through a plug of silica (cyclohexane/EtOAc 7:1) to a red oil (4.20 g), which was dissolved in EtOH (40 mL) and added to a 150 mL pressure vial. Phenylhydrazine (1.23 mL, 12.5 mmol) and 6 M HCl (2.00 mL, 12.0 mmol) were added and the vial was sealed. The reaction was stirred at 100 °C for 2.5 h and afterwards concentrated *in vacuo*. The residue was dissolved in EtOAc (100 mL) and washed with sat. $NaHCO_3$-sol., H_2O, and brine (50 mL each). The organic phase was dried over Na_2SO_4, filtered and concentrated *in vacuo*. The residue was purified via column chromatography on silica (cyclohexane/EtOAc 30:1) to give 5-(2-iodophenyl)-1-phenyl-3-(trifluoromethyl)-1*H*-pyrazole (**S4**, 4.62 g, 11.2 mmol, 93%) as a yellow oil.

^{1}H-NMR ($CDCl_3$, 601 MHz) δ (ppm) 7.88 (dd, *J* = 8.0, 1.2 Hz, 1H), 7.35 (td, *J* = 7.6, 1.2 Hz, 1H), 7.31 – 7.27 (m, 5H), 7.24 (dd, *J* = 7.6, 1.7 Hz, 1H), 7.09 (ddd, *J* = 8.0, 7.4, 1.7 Hz, 1H), 6.74 (s, 1H). ^{13}C-NMR ($CDCl_3$, 151 MHz): δ (ppm) 145.6, 142.8 (q, *J* = 37.8 Hz), 139.7, 139.0, 135.1, 131.6, 130.9, 128.9, 128.2, 128.2, 124.7, 121.2 (q, *J* = 269.1 Hz), 107.1, 99.6. IR (ATR): $\tilde{\nu}$ (cm^{-1}) 3134, 3056, 1597, 1488, 1428, 1375, 1232, 1098, 1127, 975. MS (APCI) *m/z* = 415.1 $[M+H]^+$. Analytical data is in accordance with literature data.[6]

5-Iodo-1-phenyl-1H-pyrazole (S6)

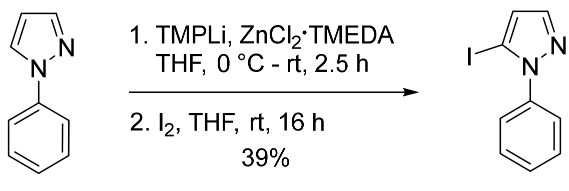

A modified literature procedure was used.[7] 2,2,6,6-Tetramethylpiperidine (1.52 mL, 9.00 mmol) was dissolved in dry THF (10 mL), cooled to 0 °C and n-BuLi (2.5 M in hexanes, 3.6 mL, 9.00 mmol) was added dropwise over the course of 5 min. The yellow solution was stirred for 15 min at this temperature, before $ZnCl_2$-TMEDA complex (750 mg, 3.00 mmol) was added and stirring continued for further 15 min at 0 °C. Then 1-phenyl-1H-pyrazole (**S5**, 870 mg, 6.00 mmol) was added and the mixture was warmed to room temperature and stirred for 2 h. A solution of iodine (2.28 g, 9.00 mmol) in dry THF (15 mL) was added over the course of 5 min and stirring continued for 16 h at room temperature. Sat. $Na_2S_2O_3$-sol. (30 mL) was added and the mixture was extracted with DCM (3 x 60 mL). The combined organic phases were washed with brine (60 mL), dried over Na_2SO_4, filtered and concentrated under reduced pressure. The residue was dried at 100 °C *in vacuo* to remove unreacted starting material, before purification via column chromatography on silica (cyclohexane/EtOAc 30:1) to give 5-iodo-1-phenyl-1H-pyrazole (**S6**, 637 mg, 2.36 mmol, 39%) as a colorless solid.

^{1}H-NMR ($CDCl_3$, 601 MHz) δ (ppm) 7.69 (d, J = 1.9 Hz, 1H), 7.55 – 7.51 (m, 2H), 7.51 – 7.47 (m, 2H), 7.47 – 7.43 (m, 1H), 6.63 (d, J = 1.9 Hz, 1H). ^{13}C-NMR ($CDCl_3$, 151 MHz): δ (ppm) 142.6, 140.1, 128.8, 128.6, 126.3, 117.4, 80.7. IR (ATR): $\tilde{\nu}$ (cm^{-1}) 3124, 3045, 1751, 1596, 1496, 1412, 1380, 1092, 957, 919. MS (APCI) m/z = 271.1 [M+H]$^+$. Mp. 92–93 °C. Analytical data is in accordance with literature data.[7]

2-Iodo-1-phenyl-1H-imidazole (S10)

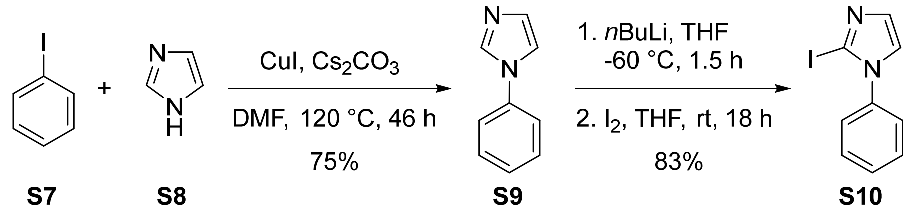

A reported literature procedure was used.[8] Iodobenzene (**S7**, 2.04 g, 10.0 mmol), imidazole (**S8**, 953 mg, 14.0 mmol), CuI (380 mg, 2.00 mmol) and Cs_2CO_3 (6.52 g, 20.0 mmol) were suspended in dry DMF (15 mL) and stirred at 120 °C for 46 h. EtOAc (20 mL) was added and the mixture was filtered through a plug of silica, which was further washed with EtOAc (300 mL). The combined organic phases were washed with brine (2 x 150 mL), dried over Na_2SO_4, filtered and concentrated under reduced pressure. The residue was purified via column chromatography on silica

(cyclohexane/EtOAc 1:1) to give 1-phenyl-1*H*-imidazole (**S9**, 1.08 g, 7.49 mmol, 75%) as a colorless oil.

^{1}H-NMR ($CDCl_3$, 601 MHz) δ (ppm) 7.90 (s, 1H), 7.50 (m, 2H), 7.42 (m, 2H), 7.39 (m, 1H), 7.33 (s, 1H), 7.25 (s, 1H). ^{13}C-NMR ($CDCl_3$, 151 MHz): δ (ppm) 137.6, 136.0, 130.7, 130.0, 127.6, 121.6, 118.7. IR (ATR): $\tilde{\nu}$ (cm^{-1}) 3374, 3114, 1599, 1504, 1459, 1303, 1247, 1180, 1112, 1056. MS (APCI) *m/z* = 145.1 $[M+H]^+$. Analytical data is in accordance with literature data.[8]

A reported literature procedure was used.[9] 1-Phenyl-1*H*-imidazole (**S9**, 288 mg, 2.00 mmol) was dissolved in dry THF (15 mL), cooled to –60 °C and *n*-BuLi (1.6 M in hexanes, 1.67 mL, 2.67 mmol) was added dropwise over the course of 5 min. After 1.5 h at this temperature, a solution of iodine (1.52 g, 6.00 mmol) dissolved in dry THF (15 mL) was added over the course over 10 min and the reaction mixture was allowed to reach room temperature over 18 h. The reaction was quenched by the addition of sat. $Na_2S_2O_3$-sol. (20 mL) and the mixture was extracted with EtOAc (3 x 20 mL). The combined organic phases were dried over Na_2SO_4, filtered and concentrated under reduced pressure. The residue was purified via column chromatography on silica (cyclohexane/EtOAc 2:1) to give 2-iodo-1-phenyl-1*H*-imidazole (**S10**, 447 mg, 1.65 mmol, 83%) as a colorless solid.

^{1}H-NMR ($CDCl_3$, 601 MHz) δ (ppm) 7.54 – 7.47 (m, 3H), 7.39 – 7.32 (m, 2H), 7.21 (s, 2H). ^{13}C-NMR ($CDCl_3$, 151 MHz): δ (ppm) 138.6, 133.1, 129.5, 129.30, 126.9, 124.9, 90.4. IR (ATR): $\tilde{\nu}$ (cm^{-1}) 3141, 3117, 3059, 3043, 1595, 1496, 1421, 1354, 1294, 1093, 1074. HR-MS (ESI) Calculated for $C_9H_8IN_2^+$ $[M+H]^+$: *m/z* 270.97267, found: *m/z* = 270.97262. Mp. 101–102 °C. Analytical data is in accordance with literature data.[9]

1-(2-Iodophenyl)-1*H*-imidazole (S12)

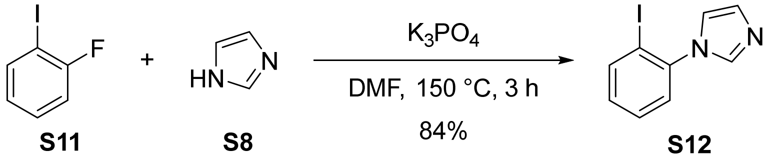

A modified literature procedure was used.[10] Imidazole (**S8**, 816 mg, 12.0 mmol), 2-fluoroiodobenzene (**S11**, 4.00 g, 18.0 mmol) and K_3PO_4 (12.7 g, 60.0 mmol) were dissolved in DMF (120 mL) and stirred for 3 h at 150 °C. After cooling to room temperature H_2O (200 mL) was added and the mixture was extracted with Et_2O (4 x 100 mL). The combined organic phases were dried over Na_2SO_4, filtered and concentrated under reduced pressure. The residue was purified via column chromatography on silica (petrol ether/EtOAc 2:1) to give 1-(2-iodophenyl)-1*H*-imidazole (**S12**, 2.73 g, 10.1 mmol, 84%) as an off-white solid.

^{1}H-NMR ($CDCl_3$, 601 MHz) δ (ppm) 7.93 (dd, *J* = 7.9, 1.4 Hz, 1H), 7.60 (t, *J* = 1.2 Hz, 1H), 7.42 (td, *J* = 7.6, 1.4 Hz, 1H), 7.26 (dd, *J* = 7.9, 1.6 Hz, 1H), 7.16 (t, *J* = 1.4 Hz, 1H), 7.13 (dd, *J* = 7.7, 1.6 Hz, 1H), 7.04 (t, *J* = 1.3 Hz, 1H). ^{13}C-NMR ($CDCl_3$, 151 MHz): δ (ppm) 140.0 (2 x C), 137.2, 130.4, 129.2,

129.1, 127.6, 120.4, 95.5. IR (ATR): $\tilde{\nu}$ (cm^{-1}) 3141, 3114, 3064, 1985, 1945, 1908, 1822, 1687, 1577, 1497, 1307, 1235, 1109, 1055, 1020. HR-MS (ESI) Calculated for $C_9H_8IN_2^+$ [M+H]$^+$: *m/z* 270.97267, found: *m/z* = 270.97251. Mp. 84–85 °C.

2-(2-Iodophenyl)imidazo[1,2-*a*]pyridine (S14)

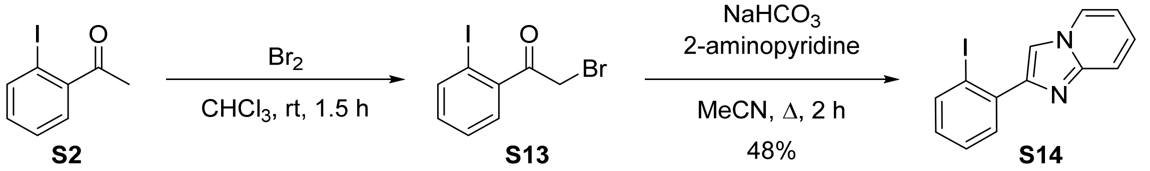

A slightly modified literature procedure was used.[11] 2'-Iodoacetophenone (**S2**, 3.69 g, 15.0 mmol) was dissolved in $CHCl_3$ (15 mL) under an atmosphere of nitrogen and bromine (790 µL, 15.5 mmol) was added dropwise. The formed yellow solution was stirred for 1.5 h before it was quenched with sat. $Na_2S_2O_3$-sol. (50 mL). The mixture was extracted with $CHCl_3$ (3 x 50 mL) and the combined organic phases were dried over Na_2SO_4, filtered and concentrated under reduced pressure. The residue (4.94 g, yellow oil) consisted of ca. 5% starting material **S2**, 78% product **S13** and 17% dibrominated by-product (^{1}H-NMR) and was used without further purification.

A slightly modified literature was procedure used.[12] The residue, 2-aminopyridine (1.46 g, 15.5 mmol) and $NaHCO_3$ (2.52 g, 30.0 mmol) were suspended in MeCN (75 mL) and stirred under reflux for 2 h. H_2O (100 mL) was added and the mixture was extracted with DCM (3 x 100 mL). The combined organic phases were dried over Na_2SO_4, filtered and concentrated under reduced pressure. The residue was purified via column chromatography on silica (cyclohexane/EtOAc 3:1) to give 2-(2-iodophenyl)imidazo[1,2-*a*]pyridine (**S14**, 2.31 g, 7.22 mmol, 48%) as a greyish solid.

^{1}H-NMR ($CDCl_3$, 601 MHz) δ (ppm) 8.16 (d, *J* = 6.8 Hz, 1H), 8.15 (s, 1H), 7.98 (dd, *J* = 7.9, 1.2 Hz, 1H), 7.88 (dd, *J* = 7.7, 1.7 Hz, 1H), 7.66 (dd, *J* = 9.3, 1.3 Hz, 1H), 7.44 (td, *J* = 7.5, 1.3 Hz, 1H), 7.20 (ddd, *J* = 9.1, 6.7, 1.3 Hz, 1H), 7.03 (td, *J* = 7.6, 1.7 Hz, 1H), 6.82 (td, *J* = 6.8, 1.2 Hz, 1H). ^{13}C-NMR ($CDCl_3$, 151 MHz): δ (ppm) 146.0, 144.5, 140.3, 138.5, 131.4, 129.3, 128.2, 125.8, 124.8, 117.7, 112.6, 111.2, 96.8. IR (ATR): $\tilde{\nu}$ (cm^{-1}) 3124, 3071, 2921, 2852, 1634, 1505, 1449, 1353, 1314, 1276, 1238, 1199, 1089. HR-MS (ESI) Calculated for $C_{13}H_{11}IN_2$ [M+H]$^+$: *m/z* 320.98832, found: *m/z* = 320.98810. Mp. 89-90 °C.

b. *N*-Methylated (*N*-HetAr)-aryl iodides

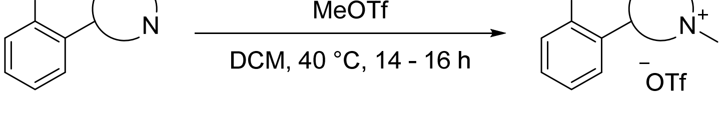

General Procedure 1 (GP1): A modified literature procedure was used.[13] The corresponding *N*-heterocyclic aryl iodide (1.00 equiv.) was dissolved in dry DCM (5 mL/mmol) and MeOTf (1.50 equiv)

was added dropwise. The reaction mixture was stirred at 40 °C for 14–16 h and afterwards concentrated under reduced pressure. The residue was either suspended in EtOAc/Et_2O, filtered, and washed with additional EtOAc/Et_2O or was dissolved in $CHCl_3$, added dropwise to Et_2O, and left standing for crystallization to give the corresponding product.

5-(2-Iodophenyl)-2-methyl-1-phenyl-1*H*-pyrazol-2-ium triflate (S15)

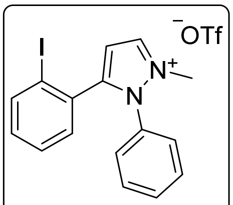
S15

Following GP1, 5-(2-iodophenyl)-1-phenyl-1*H*-pyrazole (**S3**, 1.38 g, 4.00 mmol) with MeOTf (680 µL, 6.00 mmol) after 16 h gave 5-(2-iodophenyl)-2-methyl-1-phenyl-1*H*-pyrazol-2-ium triflate (**S15**, 1.64 g, 3.21 mmol, 80%) as a colorless solid, after dissolving the crude residue in $CHCl_3$ (6 mL) and the addition to Et_2O (50 mL) followed by filtration.

^{1}H-NMR ($CDCl_3$, 601 MHz) δ (ppm) 8.67 (d, *J* = 2.9 Hz, 1H), 7.80 (dd, *J* = 8.1, 1.1 Hz, 1H), 7.63 (d, *J* = 7.6 Hz, 2H), 7.59 – 7.53 (m, 1H), 7.53 – 7.47 (m, 2H), 7.44 (dd, *J* = 7.7, 1.6 Hz, 1H), 7.32 (td, *J* = 7.6, 1.2 Hz, 1H), 7.08 (td, *J* = 7.8, 1.7 Hz, 1H), 6.86 (d, *J* = 3.0 Hz, 1H), 4.04 (s, 3H).^{13}C-NMR ($CDCl_3$, 151 MHz): δ (ppm) 151.2, 139.2, 138.8, 132.6, 132.3, 132.1, 131.4, 130.6, 130.3, 128.7, 128.5, 120.7 (q, *J* = 320.4 Hz), 109.6, 97.8, 38.5. ^{19}F-NMR ($CDCl_3$, 565 MHz): δ (ppm) –78.2. IR (ATR): $\tilde{\nu}$ (cm^{-1}) 3133, 3068, 1593, 1519, 1496, 1411, 1276, 1252, 1223, 1146, 1026. HR-MS (ESI) Calculated for $C_{16}H_{14}IN_2^+$ [M-OTf$^-$]$^+$: *m/z* 361.01962, found: *m/z* = 361.01891. Mp. 102–103 °C.

5-Iodo-2-methyl-1-phenyl-1*H*-pyrazol-2-ium triflate (S16)

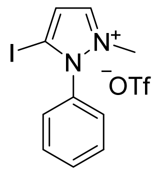
S16

Following GP1, 5-iodo-1-phenyl-1*H*-pyrazole (**S6**, 405 mg, 1.50 mmol) with MeOTf (255 µL, 2.25 mmol) after 14 h gave 5-iodo-2-methyl-1-phenyl-1*H*-pyrazol-2-ium triflate (**S16**, 586 mg, 1.35 mmol, 90%) as a colorless solid, after dissolving the crude residue in $CHCl_3$ (4 mL) and the addition to Et_2O (12 mL) followed by filtration.

^{1}H-NMR ($CDCl_3$, 601 MHz) δ (ppm) 8.57 (d, *J* = 3.0 Hz, 1H), 7.79 – 7.73 (m, 1H), 7.73 – 7.66 (m, 2H), 7.60 – 7.48 (m, 2H), 7.10 (d, *J* = 3.0 Hz, 1H), 4.03 (s, 3H). ^{13}C-NMR ($CDCl_3$, 151 MHz): δ (ppm) 140.7, 133.3, 132.3, 130.9, 128.9, 120.5 (q, *J* = 319.9 Hz), 118.1, 95.9, 40.0. ^{19}F-NMR ($CDCl_3$, 565 MHz): δ (ppm) –78.3. IR (ATR): $\tilde{\nu}$ (cm^{-1}) 3153, 3127, 3063, 1595, 1498, 1407, 1282, 1244, 1220, 1149, 1027. HR-MS (ESI) Calculated for $C_{10}H_{10}IN_2^+$ [M-OTf$^-$]$^+$: *m/z* 284.98832, found: *m/z* = 284.98810. Mp. 105–107 °C.

1-(2-Iodophenyl)-3-methyl-1*H*-imidazol-3-ium triflate (S17)

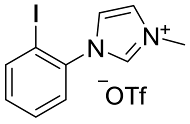
S17

Following GP1, 1-(2-iodophenyl)-1*H*-imidazole (**S12**, 540 mg, 2.00 mmol) with MeOTf (340 µL, 3.00 mmol) after 15 h gave 1-(2-iodophenyl)-3-methyl-1*H*-imidazol-3-ium triflate (**S17**, 784 mg, 1.81 mmol, 90%) as a colorless

solid, after suspending the residue in EtOAc/Et_2O (1:3, 4 mL) followed by filtration and washing with Et_2O (2 x 2 mL).

^{1}H-NMR (d_6-DMSO, 601 MHz) δ (ppm) 9.53 (s, 1H), 8.14 (dd, J = 7.9, 1.2 Hz, 1H), 8.06 (t, J = 1.8 Hz, 1H), 7.98 (t, J = 1.8 Hz, 1H), 7.72 – 7.63 (m, 2H), 7.43 (ddd, J = 7.9, 6.7, 2.3 Hz, 1H), 3.99 (s, 3H). ^{13}C-NMR (d_6-DMSO, 151 MHz): δ (ppm) 139.9, 137.8, 137.6, 132.6, 129.8, 128.2, 124.2, 123.9, 120.7 (q, J = 322.2 Hz), 96.3, 36.2. ^{19}F-NMR (d_6-DMSO, 565 MHz): δ (ppm) –77.8. IR (ATR): $\tilde{\nu}$ (cm^{-1}) 3145, 3101, 3077, 1549, 1474, 1244, 1150, 1030, 769. HR-MS (ESI) Calculated for $C_{10}H_{10}IN_2^+$ [M-OTf$^-$]$^+$: *m/z* 284.98832, found: *m/z* = 284.98817. Mp. 82–83 °C.

2-(2-Iodophenyl)-1-methylimidazo[1,2-*a*]pyridin-1-ium triflate (S18)

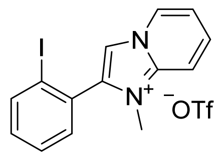

S18

Following GP1, 2-(2-iodophenyl)imidazo[1,2-*a*]pyridine (**S14**, 960 mg, 3.00 mmol) with MeOTf (510 µL, 4.50 mmol) after 15 h gave 2-(2-iodophenyl)-1-methylimidazo[1,2-*a*]pyridin-1-ium triflate (**S18**, 1.15 g, 2.37 mmol, 79%) as a colorless solid, after suspending the residue in EtOAc/Et_2O (1:2, 6 mL) followed by filtration and washing with EtOAc/Et_2O (1:1, 2 x 2 mL).

^{1}H-NMR (d_6-DMSO, 601 MHz) δ (ppm) 9.00 (d, J = 6.7 Hz, 1H), 8.60 (s, 1H), 8.33 (d, J = 9.1 Hz, 1H), 8.25 – 8.07 (m, 2H), 7.74 – 7.62 (m, 2H), 7.60 (dd, J = 7.6, 1.7 Hz, 1H), 7.43 (td, J = 7.7, 1.7 Hz, 1H), 3.75 (s, 3H). ^{13}C-NMR (d_6-DMSO, 151 MHz): δ (ppm) 139.4, 139.1, 138.5, 134.1, 132.9, 132.6, 130.4, 129.5, 128.8, 120.7 (q, J = 322.5 Hz), 117.8, 114.1, 111.6, 101.0, 31.8. ^{19}F-NMR (d_6-DMSO, 565 MHz): δ (ppm) –77.8. IR (ATR): $\tilde{\nu}$ (cm^{-1}) 3134, 1653, 1537, 1245, 1156, 1027, 765. HR-MS (ESI) Calculated for $C_{14}H_{12}IN_2^+$ [M-OTf$^-$]$^+$: *m/z* 335.00397, found: *m/z* = 335.00383. Mp. 182–183 °C.

c. Sodium tetrakis(fluorophenyl)borates

Sodium tetrakis(3,5-bis(trifluoromethyl)phenyl)borate (S20)

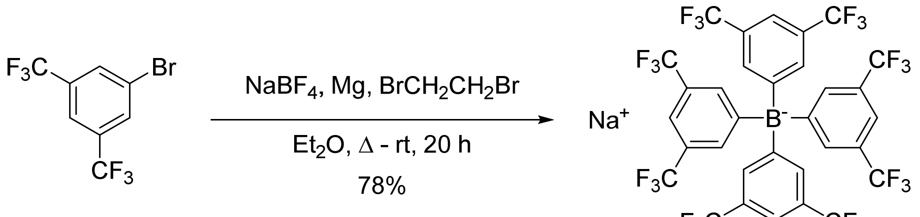

S19

S20

A slightly modified literature procedure was used.[14] To a three-necked-round bottom flask equipped with a reflux condenser and a dropping funnel was added magnesium turnings (3.15 g, 130 mmol), $NaBF_4$ (2.20 g, 20.0 mmol) and dry Et_2O (450 mmol). To this was added dibromoethane (1.55 mL, 18.0 mmol) and the mixture was refluxed for 10 min to initiate the reaction, the heating was stopped and the mixture was stirred for 45 min. Then, 3,5-bis(trifluoromethyl)bromobenzene (**S19**, 32.8 g,

112 mmol) dissolved in dry Et_2O (150 mL) was added dropwise over the course of 1.5 h. Afterwards the mixture was refluxed for 1 h and then stirred at room temperature overnight (~16 h). The reaction was quenched with Na_2CO_3 (50.0 g) dissolved in H_2O (600 mL) and the mixture was stirred vigorously for 45 min. The precipitate was filtered off, the phases were separated and the aqueous phase was extracted with Et_2O (3 x 150 mL). The combined organic phases were dried over Na_2SO_4 and activated charcoal (6.25 g), filtered and concentrated *in vacuo*. The residue was dissolved in benzene (70 mL) and residual water was removed using a Dean–Stark apparatus. The solvent was removed in vacuo and the residue was washed with heptane/DCM (5:1, 2 x 15 mL) and heptane (15 mL). Drying (110 °C, 10^{-2} mbar) for 15 h gave sodium tetrakis(3,5-bis(trifluoromethyl)phenyl)borate (**S20**, 13.9 g, 15.7 mmol, 78%) as an off-white solid.

^{1}H-NMR (d_6-DMSO, 601 MHz): δ (ppm) 7.65 (s, 4H), 7.61 (br s, 8H). ^{11}B-NMR (d_6-DMSO, 193 MHz): δ (ppm) –6.8. ^{13}C-NMR (d_6-DMSO, 151 MHz): δ (ppm) δ 161.0 (dd, J = 99.6, 50.0 Hz), 134.0, 128.5 (q, J = 31.1 Hz), 124.0 (q, J = 272.6 Hz), 117.6. ^{19}F-NMR (d_6-DMSO, 565 MHz): δ (ppm) –61.9. IR (ATR): $\tilde{\nu}$ (cm^{-1}) 1629, 1610, 1357, 1281, 1119, 1065, 945, 932, 886, 837. MS (APCI) *m/z* = 863.0 [M-Na$^+$]$^-$. Analytical data is in accordance with literature data.[14]

Sodium tetrakis(pentafluorophenyl)borate (S22)

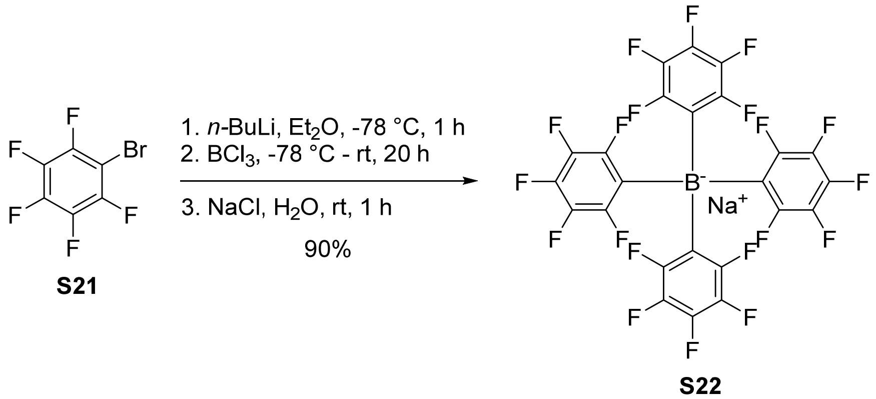

S21 **S22**

A slightly modified literature procedure was used.[15] Pentafluorobromobenzene (**S21**, 5.12 g, 20.8 mmol) was dissolved in dry Et_2O (100 mL) and cooled to -78 °C. *n*-BuLi (2.5 M in hexanes, 6.4 mL, 16.0 mmol) was added dropwise over the course of 5 min and the mixture was stirred for 1 h at -78 °C. At this temperature BCl_3 (1 M in heptane, 4.0 mL, 4.00 mmol) was added dropwise over the course of 10 min and the mixture was slowly allowed to reach room temperature over 20 h. Then, NaCl (4.84 g, 82.8 mmol) and H_2O (80 mL) were added and the mixture was stirred vigorously at room temperature for 1 h. The phases were separated and the organic phase was washed with H_2O (2 x 80 mL) and brine (2 x 80 mL), dried over Na_2SO_4, filtered and concentrated *in vacuo*. The residue was suspended in pentane (50 mL) in a sonication bath for 20 min. The supernatant was removed by decantation and the residue was washed with pentane (50 mL) one more time. The

residue was dried (170 °C/10^{-2} mbar) for 20 h to give sodium tetrakis(pentafluorophenyl)borate (**S22**, 2.52 g, 3.59 mmol, 90%) as a colorless solid.

^{11}B-NMR (d_6-DMSO, 193 MHz): δ (ppm) –16.7. ^{13}C-NMR (d_6-DMSO, 151 MHz): δ (ppm) 147.5 (br d, *J* = 241.1 Hz), 137.6 (br d, *J* = 245.7 Hz), 135.6 (br d, *J* = 245.6 Hz), 126.3 – 121.4 (br s/m). ^{19}F-NMR (d_6-DMSO, 565 MHz): δ (ppm) –132.4 (d, J = 13.5 Hz), –161.3 (t, J = 21.5 Hz), –165.9 (t, J = 20.5 Hz). IR (ATR): $\tilde{\nu}$ (cm^{-1}) 1650, 1519, 1451, 1364, 1276, 1109, 1088, 964, 772. MS (APCI) *m/z* = 678.9 [M-Na$^+$]$^-$. Analytical data is in accordance with literature data.[15]

4. Synthesis of iod(az)olium salts

1-Phenyl-1*H*-benzo[4,5]iodolo[3,2-*c*]pyrazol-4-ium triflate (7a)

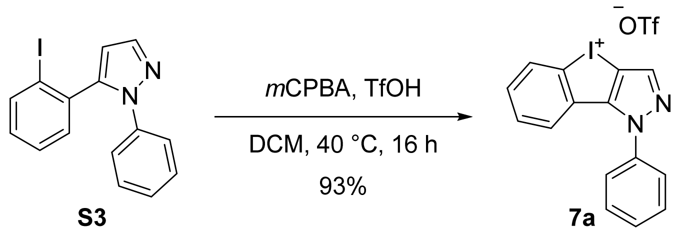

A modified literature procedure was used.[6] 5-(2-Iodophenyl)-1-phenyl-1*H*-pyrazole (**S3**, 692 mg, 2.00 mmol) and *m*CPBA (85%, 490 mg, 2.40 mmol) were dissolved in DCM (10 mL) and TfOH (352 µL, 4.00 mmol) was added dropwise. The reaction mixture was stirred for 16 h at 40 °C and afterwards Et_2O (5 mL) was added. The mixture was stored at 4 °C for 30 min and the formed precipitate was filtered off and washed with Et_2O (2x5 mL) to give 1-phenyl-1*H*-benzo[4,5]iodolo[3,2-*c*]pyrazol-4-ium triflate (**7a**, 920 mg, 1.86 mmol, 93%) as a colorless solid.

^{1}H-NMR (d_6-DMSO, 601 MHz) δ (ppm) 8.31 (dd, *J* = 8.2, 1.3 Hz, 1H), 8.18 (s, 1H), 7.74 – 7.70 (m, 3H), 7.69 – 7.66 (m, 2H), 7.65 (td, *J* = 7.6, 1.3 Hz, 1H), 7.61 (ddd, *J* = 8.9, 7.4, 1.7 Hz, 1H), 7.23 (dd, *J* = 7.7, 1.7 Hz, 1H). ^{13}C NMR (d_6-DMSO, 151 MHz) δ (ppm) 147.9, 138.8, 138.7, 131.6, 130.6, 130.5, 130.4, 130.0, 128.0, 126.7, 126.4, 125.3, 120.7 (q, *J* = 322.4 Hz), 94.6. ^{19}F-NMR (d_6-DMSO, 565 MHz) δ (ppm) -77.7. IR (ATR): $\tilde{\nu}$ (cm^{-1}) 3128, 3103, 1593, 1498, 1425, 1395, 1289, 1220, 1182, 1022. MS (APCI) *m/z* = 345.0 [M-OTf]$^+$. Mp. 229–231 °C (decomp.). Analytical data is in accordance with literature data.[6]

1-Phenyl-3-(trifluoromethyl)-1*H*-benzo[4,5]iodolo[3,2-*c*]pyrazol-4-ium triflate (7b)

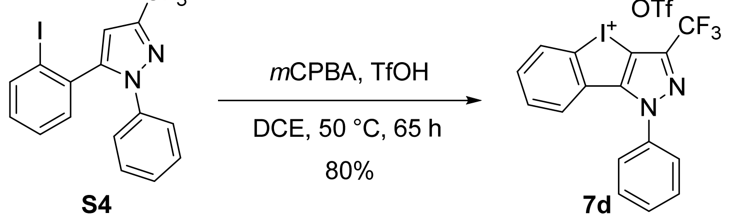

A slightly modified literature procedure was used.[6] 5-(2-Iodophenyl)-1-phenyl-3-(trifluoromethyl)-1*H*-pyrazole (**S4**, 1.24 g, 3.00 mmol) and *m*CPBA (85%, 740 mg, 3.60 mmol) were dissolved in DCE (7.5 mL) and TfOH (530 µL, 6.00 mmol) was added dropwise. The reaction mixture was stirred for 65 h at 50 °C and afterwards Et_2O (10 mL) was added. The mixture was stored at 4 °C for 30 min and the formed precipitate was filtered off and washed with Et_2O (3 x 5 mL) to give 1-phenyl-3-(trifluoromethyl)-1*H*-benzo[4,5]iodolo[3,2-*c*]pyrazol-4-ium triflate (**7d**, 1.35 g, 2.40 mmol, 80%) as a colorless solid.

^{1}H-NMR (d_6-DMSO, 601 MHz) δ (ppm) 8.70 – 8.21 (m, 1H), 7.82 – 7.73 (m, 5H), 7.73 – 7.67 (m, 2H), 7.35 – 7.00 (m, 1H). ^{13}C NMR (d_6-DMSO, 151 MHz) δ (ppm) 152.0, 138.7 (q, *J* = 40.2 Hz), 137.7, 131.9, 131.8, 131.4, 130.9, 130.3, 127.3, 126.9, 126.6, 125.9, 120.7 (q, *J* = 322.3 Hz), 119.9 (q, *J* = 269.3 Hz), 91.4. ^{19}F-NMR (d_6-DMSO, 565 MHz) δ (ppm) -60.2 (s, 3F), -77.8 (s, 3F). IR (ATR): $\tilde{\nu}$ (cm^{-1}) 3106, 1595, 1487, 1434, 1282, 1236, 1165, 1134, 1023, 995. MS (APCI) *m/z* = 413.0 [M-OTf$^-$]$^+$. Mp. 173–175 °C (decomp.). Analytical data is in accordance with literature data.[6]

1-Phenyl-1*H*-benzo[4,5]iodolo[3,2-*c*]pyrazol-4-ium chloride (7c)

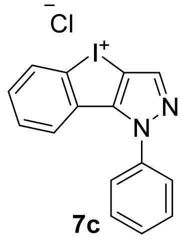

7c

A crystal suitable for X-ray diffraction of 1-phenyl-1*H*-benzo[4,5]iodolo[3,2-*c*]pyrazol-4-ium chloride (**7c**) was obtained directly from the reaction mixture of 1-phenyl-1*H*-benzo[4,5]iodolo[3,2-*c*]pyrazol-4-ium triflate (**7a**) with benzhydryl chloride and was solely used to gain structural information of such halogen bond donors (CCDC 2082275).

1-Phenyl-1*H*-benzo[4,5]iodolo[3,2-*c*]pyrazol-4-ium tetrakis(3,5-bis(trifluoromethyl)phenyl)borate (7d)

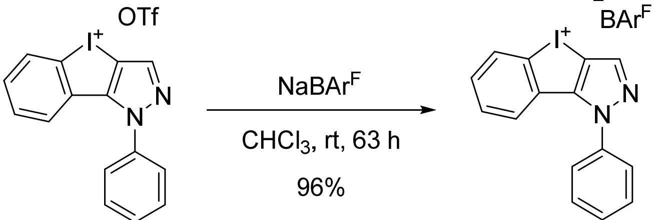

7a **7d**

A slightly modified literature procedure was used.[16] 1-Phenyl-1*H*-benzo[4,5]iodolo[3,2-*c*]pyrazol-4-ium triflate (**7a**, 198 mg, 0.400 mmol) and sodium tetrakis(3,5-bis(trifluoromethyl)phenyl)borate (355 mg, 0.400 mmol) were dissolved in $CHCl_3$ (8 mL) and stirred at room temperature for 63 h. Afterwards the reaction mixture was stored for 1 h at –17 °C, filtered and the filtrate was concentrated to ~1 mL. *n*-Pentane (10 mL) was added and the formed precipitate was separated via decantation and dried *in vacuo* to give 1-phenyl-1*H*-benzo[4,5]iodolo[3,2-*c*]pyrazol-4-ium tetrakis(3,5-bis(trifluoromethyl)phenyl)borate (**7d**, 264 mg, 0.233 mmol, 78%) as a light beige solid.

^{1}H-NMR (d_6-DMSO, 601 MHz) δ (ppm) 8.32 (dd, J = 8.2, 1.2 Hz, 1H), 8.19 (s, 1H), 7.74 – 7.70 (m, 7H), 7.69 – 7.66 (m, 2H), 7.66 – 7.59 (m, 10H), 7.23 (dd, J = 7.8, 1.6 Hz, 1H). ^{13}C-NMR (d_6-DMSO, 151 MHz): δ (ppm) 161.0 (dd, J = 99.6, 49.8 Hz), 147.9, 138.9, 138.7, 134.1, 131.6, 130.5, 130.5, 130.4, 130.0, 128.5 (dq, J = 31.4, 2.5 Hz), 128.1, 126.7, 126.4, 125.3, 124.0 (q, J = 272.6 Hz), 117.6, 94.7. ^{19}F-NMR (d_6-DMSO, 565 MHz): δ (ppm) –61.6. IR (ATR): $\tilde{\nu}$ (cm^{-1}) 3101, 1611, 1501, 1354, 1274, 1116, 970, 939, 887. MS (APCI) m/z = 345.0 [M-BAr^{F-}]$^+$. HR-MS (EI) Calculated for $C_{15}H_{11}IN_2^{+\bullet}$ [M+H-BArF$^-$]$^{+\bullet}$: m/z 345.99614, found: m/z = 345.99555. Mp. 137–140 °C.

2-Methyl-1-phenyl-1*H*-benzo[4,5]iodolo[3,2-*c*]pyrazole-2,4-diium bistriflate (7e)

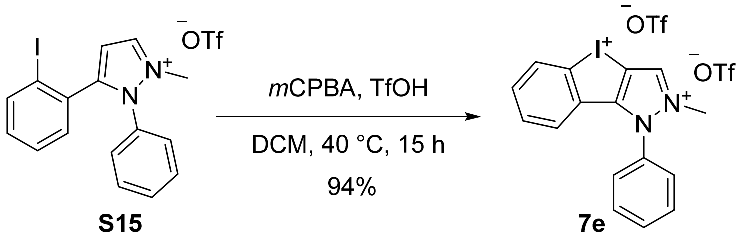

A modified literature procedure was used.[6] 5-(2-Iodophenyl)-2-methyl-1-phenyl-1*H*-pyrazol-2-ium triflate (**S15**, 765 mg, 1.50 mmol) and *m*CPBA (85%, 368 mg, 1.80 mmol) were dissolved in DCM (8 mL) and TfOH (265 µL, 3.00 mmol) was added dropwise. The reaction mixture was stirred for 16 h at 40 °C and afterwards Et_2O (5 mL) was added. The mixture was stored at 4 °C for 30 min and the formed precipitate was filtered off and washed with Et_2O (2 x 5 mL) to give 2-methyl-1-phenyl-1*H*-benzo[4,5]iodolo[3,2-*c*]pyrazole-2,4-diium bistriflate (**7e**, 925 mg, 1.41 mmol, 94%) as a colorless solid.

^{1}H-NMR (d_6-DMSO, 601 MHz) δ (ppm) 9.18 (s, 1H), 8.39 (d, J = 8.4 Hz, 1H), 8.02 – 7.97 (m, 1H), 7.96 – 7.88 (m, 4H), 7.83 (t, J = 7.8 Hz, 1H), 7.71 (t, J = 7.6 Hz, 1H), 6.78 (d, J = 7.8 Hz, 1H), 4.05 (s, 3H). ^{13}C NMR (d_6-DMSO, 151 MHz) δ (ppm) 151.6, 138.7, 134.1, 134.0, 131.9, 131.5, 131.2, 130.6, 129.1, 128.8, 126.8, 126.3, 120.7 (q, J = 322.3 Hz), 95.0, 38.4. ^{19}F-NMR (d_6-DMSO, 565 MHz) δ (ppm) -77.7. IR (ATR): $\tilde{\nu}$ (cm^{-1}) 3093, 1582, 1494, 1453, 1283, 1212, 1170, 1017, 960. HR-MS (ESI) Calculated for $C_{16}H_{12}IN_2^+$ [M-H-2OTf$^-$]$^+$: m/z = 359.00397, found: m/z = 359.00351. Mp. 252–254 °C (decomp.).

2-Methyl-1-phenyl-1*H*-benzo[4,5]iodolo[3,2-*c*]pyrazole-2,4-diium bis(tetrakis(3,5-bis(trifluoromethyl)phenyl)borate) etherate complex (7f)

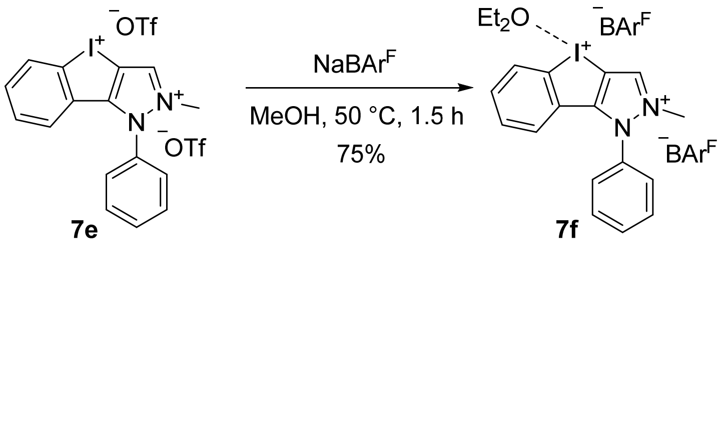

A modified literature procedure was used.[17] 2-Methyl-1-phenyl-1*H*-benzo[4,5]iodolo[3,2-*c*]pyrazole-2,4-diium bistriflate (**7e**, 65.8 mg, 100 µmol) and sodium tetrakis(3,5-bis(trifluoromethyl)phenyl)borate (177 mg, 200 µmol) were dissolved in dry MeOH (2.5 mL) and stirred at 50 °C for 1.5 h. Afterwards the solvent was removed under reduced pressure and the residue was suspended in DCM (3 mL). The mixture was stirred for 20 min at room temperature, then stored for 30 min at 4 °C and afterwards filtered. *n*-Pentane (10 mL) was added to the filtrate and after decantation, the formed residue was dried *in vacuo* to give 2-methyl-1-phenyl-1*H*-benzo[4,5]iodolo[3,2-*c*]pyrazole-2,4-diium bis(tetrakis(3,5-bis(trifluoromethyl)phenyl)borate) as a mono-MeOH complex (167 mg, 78.8 µmol, 79%) as a beige solid. This was suspended in Et_2O (2 mL) and *n*-pentane (10 mL) was added. After decantation and drying *in vacuo* 2-methyl-1-phenyl-1*H*-benzo[4,5]iodolo[3,2-*c*]pyrazole-2,4-diium bis(tetrakis(3,5-bis(trifluoromethyl)phenyl)borate) was obtained as a mono-Et_2O complex (**7f**, 162 mg, 75.0 µmol, 75%) as a colorless solid.

^{1}H-NMR (d_6-DMSO, 601 MHz) δ (ppm) 9.18 (d, J = 0.7 Hz, 1H), 8.41 (d, J = 8.4 Hz, 1H), 8.06 – 7.95 (m, 1H), 7.95 – 7.87 (m, 4H), 7.83 (ddd, J = 8.6, 7.4, 1.5 Hz, 1H), 7.76 – 7.68 (m, 9H), 7.65 – 7.54 (m, 16H), 6.78 (dd, J = 8.0, 1.5 Hz, 1H), 4.05 (d, J = 0.6 Hz, 3H), 3.38 (q, J = 7.0 Hz, 4H), 1.09 (t, J = 7.0 Hz, 6H). ^{13}C NMR (d_6-DMSO, 151 MHz) δ (ppm) 160.9 (dd, J = 99.6, 49.8 Hz), 151.5, 138.7, 134.0, 131.9, 131.5, 131.2, 130.6, 129.1, 128.8, 128.5 (dq, J = 31.5, 3.0 Hz), 126.8, 126.3, 124.0 (q, J = 272.5 Hz), 117.7, 95.2, 64.9, 38.4, 15.1. ^{19}F-NMR (d_6-DMSO, 565 MHz) δ (ppm) -61.6. IR (ATR): $\tilde{\nu}$ (cm^{-1}) 3154, 1610, 1444, 1354, 1272, 1109, 955, 886, 838. HR-MS (ESI) Calculated for $C_{16}H_{12}IN_2^+$ [M-H-2BAr^{F-}]$^+$: *m/z* = 359.00397, found: *m/z* = 359.00314. Mp. 64–67 °C.

Benzo[*d*]pyrazolo[5,1-*b*][1,3]iodazol-4-ium triflate (8a)

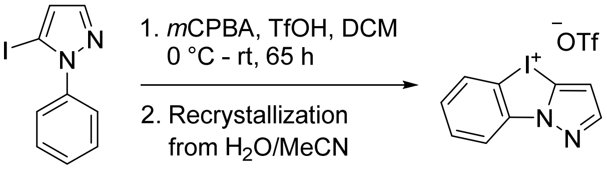

S6 42% **8a**

A slightly modified literature procedure was used.[6] 5-Iodo-1-phenyl-1*H*-pyrazole (**S6**, 810 mg, 3.00 mmol) and *m*CPBA (85%, 738 mg, 3.60 mmol) were dissolved in DCM (30 mL) and cooled to 0 °C. TfOH (663 µL, 7.50 mmol) was added dropwise and the mixture was stirred for 65 h at room temperature. Afterwards the solvent was removed under reduced pressure and the residue was suspended in Et_2O (20 mL) and stored at 4 °C for 15 min. The precipitate was filtered and washed with Et_2O (4 x 5 mL) to give the desired product (839 mg, purity 94%). Recrystallization from H_2O with few drops of MeCN gave benzo[*d*]pyrazolo[5,1-*b*][1,3]iodazol-4-ium triflate (**8a**, 523 mg, 1.25 mmol, 42%) as a colorless solid.

^{1}H-NMR (d_6-DMSO, 601 MHz) δ (ppm) 8.15 (dd, J = 8.4, 1.1 Hz, 1H), 8.09 (dd, J = 8.1, 1.4 Hz, 1H), 8.07 (d, J = 1.9 Hz, 1H), 7.89 – 7.79 (m, 1H), 7.58 (ddd, J = 8.5, 7.3, 1.4 Hz, 1H), 6.92 (d, J = 2.0

Hz, 1H). ^{13}C NMR (d_6-DMSO, 151 MHz) δ (ppm) 144.2, 136.9 132.1, 130.7, 128.4, 120.7 (q, J = 322.2 Hz), 116.9, 111.4, 111.0, 110.7. ^{19}F-NMR (d_6-DMSO, 565 MHz) δ (ppm) -77.8. IR (ATR): $\tilde{\nu}$ (cm^{-1}) 3142, 3099, 1580, 1504, 1475, 1382, 1284, 1222, 1183, 1021. MS (APCI) m/z = 269.0 [M-OTf$^-$]$^+$. HR-MS (EI) Calculated for $C_9H_7IN_2^{+\bullet}$ [M+H-OTf$^-$]$^{+\bullet}$: m/z = 269.96484, found: m/z = 269.96513. Mp. 264–267 °C (decomp.).

Benzo[*d*]pyrazolo[5,1-*b*][1,3]iodazol-4-ium tetrakis(3,5-bis(trifluoromethyl)phenyl)borate (8b)

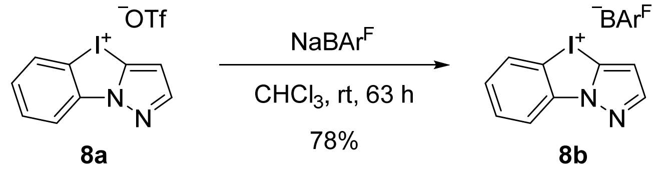

A slightly modified literature procedure was used.[16] Benzo[*d*]pyrazolo[5,1-*b*][1,3]iodazol-4-ium triflate (**8a**, 125 mg, 0.300 mmol) and sodium tetrakis(3,5-bis(trifluoromethyl)phenyl)borate (266 mg, 0.300 mmol) were dissolved in $CHCl_3$ (6 mL) and stirred at room temperature for 63 h. Afterwards the reaction mixture was stored for 2 h at 4 °C, filtered and *n*-pentane (10 mL) was added to the filtrate. The formed oil was separated via decantation and dried *in vacuo* to give benzo[*d*]pyrazolo[5,1-*b*][1,3]iodazol-4-ium tetrakis(3,5-bis(trifluoromethyl)phenyl)borate (**8b**, 264 mg, 0.233 mmol, 78%) as a yellow solid.

^{1}H-NMR ($CDCl_3$, 601 MHz) δ (ppm) 8.19 (dd, J = 8.0, 1.6 Hz, 1H), 7.98 (d, J = 2.1 Hz, 1H), 7.86 (ddd, J = 8.2, 7.4, 1.0 Hz, 1H), 7.81 (d, J = 8.7 Hz, 1H), 7.76 – 7.68 (m, 8H), 7.55 – 7.47 (m, 5H), 6.77 (d, J = 2.1 Hz, 1H). ^{13}C-NMR (d_6-DMSO, 151 MHz): δ (ppm) 161.0 (dd, J = 99.6, 49.8 Hz), 144.2, 136.9, 134.0, 132.1, 130.7, 128.5 (dq, J = 31.4, 2.5 Hz), 128.4, 124.0 (q, J = 272.5 Hz), 117.6, 116.9, 111.4, 111.2, 110.7. ^{19}F-NMR ($CDCl_3$, 565 MHz): δ (ppm) –62.3. IR (ATR): $\tilde{\nu}$ (cm^{-1}) 3158, 1610, 1509, 1475, 1382, 1352, 1272, 1112, 885, 837. HR-MS (ESI) Calculated for $C_9H_8IN_2^+$ [M-BAr^{F-} +2H]$^+$: m/z 270.97267, found: m/z = 270.97216. Mp. 85–87 °C.

1-Methylbenzo[*d*]pyrazolo[5,1-*b*][1,3]iodazole-1,4-diium bistriflate (8c)

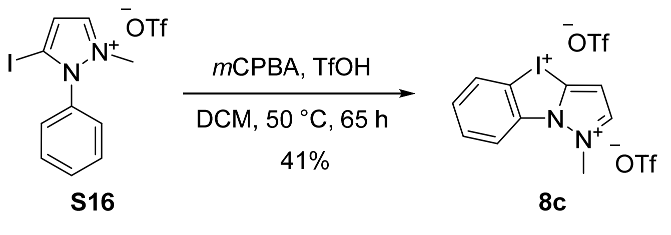

A modified literature procedure was used.[6] 5-Iodo-2-methyl-1-phenyl-1*H*-pyrazol-2-ium triflate (**S16**, 130 mg, 0.300 mmol) and *m*CPBA (85%, 73.9 mg, 0.360 mmol) were dissolved in DCM (2 mL) and TfOH (66.0 µL, 0.750 mmol) was added dropwise. The reaction mixture was stirred for 65 h at 50 °C and afterwards the mixture was concentrated to ~1 mL and Et_2O (2 mL) was added. The

mixture was stored at 4 °C for 30 min and the formed precipitate was filtered and washed with Et_2O (2 x 1 mL) to give 1-methylbenzo[*d*]pyrazolo[5,1-*b*][1,3]iodazole-1,4-diium bistriflate (**8c**, 72.0 mg, 0.124 mmol, 41%) as a colorless solid.

^{1}H-NMR (d_6-DMSO, 601 MHz) δ (ppm) 8.88 (d, *J* = 3.1 Hz, 1H), 8.59 (dd, *J* = 8.5, 1.2 Hz, 1H), 8.35 (dd, *J* = 8.3, 1.3 Hz, 1H), 8.01 (ddd, *J* = 8.6, 7.4, 1.3 Hz, 1H), 7.82 (ddd, *J* = 8.4, 7.4, 1.1 Hz, 1H), 7.45 (d, *J* = 3.1 Hz, 1H), 4.79 (s, 3H). ^{13}C NMR (d_6-DMSO, 151 MHz) δ (ppm) 143.4, 135.0, 132.2, 131.3, 130.6, 122.8, 120.7 (q, *J* = 322.3 Hz), 119.3, 112.6, 112.2, 42.4. ^{19}F-NMR (d_6-DMSO, 565 MHz) δ (ppm) -77.7. IR (ATR): $\tilde{\nu}$ (cm^{-1}) 3136, 3043, 1460, 1142, 1408, 1302, 1231, 1208, 1157, 1019. HR-MS (ESI) Calculated for $C_{10}H_{10}IN_2^+$ [M+H-2OTf$^-$]$^+$: *m/z* = 284.98832, found: *m/z* = 284.98803. Mp. 254–256 °C (decomp.).

1-(2-((3,5-Bis(trifluoromethyl)phenyl)iodonio)phenyl)-2-methyl-1*H*-pyrazol-2-ium bis(tetrakis(3,5-bis(trifluoromethyl)phenyl)borate (16)

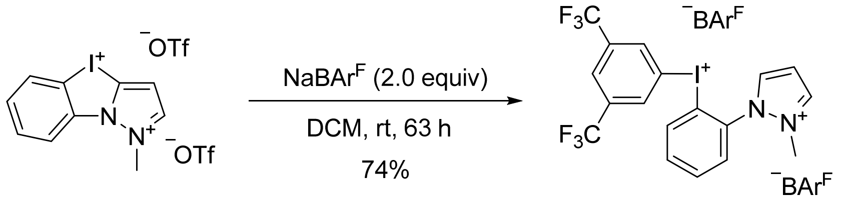

8c **16**

1-Methylbenzo[*d*]pyrazolo[5,1-*b*][1,3]iodazole-1,4-diium bistriflate (**8c**, 23.3 mg, 40.0 µmol) and sodium tetrakis(3,5-bis(trifluoromethyl)phenyl)borate (70.9 mg, 80.0 µmol) were dissolved in dry DCM (1 mL) and stirred at room temperature for 63 h. Afterwards the reaction mixture was stored for 2 h at 4 °C, filtered and *n*-pentane (3 mL) was added to the filtrate. The formed precipitate was filtered, washed with *n*-pentane and dried *in vacuo* to give 1-(2-((3,5-bis(trifluoromethyl)phenyl)iodonio)phenyl)-2-methyl-1*H*-pyrazol-2-ium bis(tetrakis(3,5-bis(trifluoromethyl)phenyl)borate (**16**, 66.2 mg, 29.5 µmol, 74%) as a slightly yellowish solid.

^{1}H-NMR (d_6-DMSO, 601 MHz) δ (ppm) 8.98 (d, *J* = 2.9 Hz, 1H), 8.94 (s, 2H), 8.90 (dd, *J* = 8.0, 1.5 Hz, 1H), 8.61 (dd, *J* = 3.1, 1.0 Hz, 1H), 8.52 (s, 1H), 8.12 (dd, *J* = 7.8, 1.7 Hz, 1H), 8.06 (td, *J* = 7.6, 1.5 Hz, 1H), 8.02 (td, *J* = 7.8, 1.7 Hz, 1H), 7.72 (s, 8H), 7.62 (s, 16H), 7.21 (t, *J* = 3.0 Hz, 1H), 3.80 (s, 3H). ^{13}C NMR (d_6-DMSO, 151 MHz) δ (ppm) 160.9 (dd, *J* = 99.5, 49.8 Hz), 141.4, 140.1, 138.6, 136.3, 135.9, 134.9, 134.0, 132.9, 132.5 (q, *J* = 33.9 Hz), 132.0, 128.5 (dq, *J* = 31.7, 2.6 Hz), 124.0 (q, *J* = 272.4 Hz), 123.9 (q, *J* = 274.6 Hz), 117.7, 108.9, 37.7. ^{19}F-NMR (d_6-DMSO, 565 MHz) δ (ppm) –61.2 (s, 6F), –61.6 (s, 48F). IR (ATR): $\tilde{\nu}$ (cm^{-1}) 3150, 1611, 1145, 1354, 1273, 1109, 886, 838. HR-MS (ESI) Calculated for $C_{10}H_{10}IN_2^+$ [M-($C_8H_2F_6$)-2BArF$^-$]$^+$: *m/z* = 284.98832, found: *m/z* = 284.98797 (no observation of the dication possible; MS only obtained with reduction of the iodine center by elimination of the 3,5-bis(trifluoromethyl)phenyl moiety). Mp. 61–64 °C.

1-Methylbenzo[*d*]pyrazolo[5,1-*b*][1,3]iodazole-1,4-diium tetrakis(pentafluorophenyl)borate dietherate complex (8d)

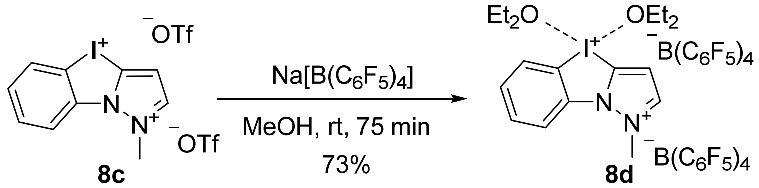

A modified literature procedure was used.[17] 1-Methylbenzo[*d*]pyrazolo[5,1-*b*][1,3]iodazole-1,4-diium bistriflate (**8c**, 58.2 mg, 100 µmol) and sodium tetrakis(pentafluorophenyl)borate (141 mg, 200 µmol) were dissolved in dry MeOH (2.5 mL) and stirred at room temperature for 75 min. Afterwards the solvent was removed under reduced pressure at room temperature and the residue was suspended in DCM (3 mL) with a minimum amount of Et_2O (~0.2 mL). The mixture was stirred for 20 min at room temperature, then stored for 30 min at 4 °C and afterwards filtered. *n*-Pentane (10 mL) was added to the filtrate and after decantation, the formed residue was dried *in vacuo*. The residue was suspended in Et_2O (1 mL) and *n*-pentane (10 mL) was added. After decantation and drying *in vacuo* the residue was dissolved in DCM (6 mL) and Et_2O (2 mL) and washed with H_2O (3 mL). The solvent was removed *in vacuo* to give 1-methylbenzo[*d*]pyrazolo[5,1-*b*][1,3]iodazole-1,4-diium tetrakis(pentafluorophenyl)borate (**8d**) as a dietherate complex (130 mg, 72.6 µmol, 73%) as an off-white solid.

^{1}H-NMR (d_6-DMSO, 601 MHz) δ (ppm) 8.87 (s, 1H), 8.59 (d, *J* = 8.5 Hz, 1H), 8.37 (d, *J* = 8.3 Hz, 1H), 8.00 (t, *J* = 7.7 Hz, 1H), 7.82 (t, *J* = 7.9 Hz, 1H), 7.43 (s, 1H), 4.78 (s, 3H), 3.38 (q, *J* = 7.0 Hz, 8H), 1.09 (t, *J* = 7.0 Hz, 12H). ^{13}C NMR (d_6-DMSO, 151 MHz) δ (ppm) 147.5 (br d, *J* = 241.0 Hz), 143.4, 137.6 (br d, *J* = 245.3 Hz), 135.6 (br d, *J* = 245.5 Hz), 135.0, 132.2, 131.4, 130.6, 125.0 – 121.5 (br s/m), 119.3, 112.7, 112.1, 64.9, 42.4, 15.1. ^{19}F-NMR (d_6-DMSO, 565 MHz) δ (ppm) -132.4 (d, *J* = 13.1 Hz, 16F), -161.3 (t, *J* = 21.6 Hz, 8F), -165.9 (t, *J* = 20.6 Hz, 16F). IR (ATR): $\tilde{\nu}$ (cm^{-1}) 3144, 2981, 1644, 1513, 1456, 1411, 1274, 1083, 974. HR-MS (ESI) Calculated for $C_{10}H_{10}IN_2^+$ [M+H-2BAr^{F-}]$^+$: *m/z* = 284.98832, found: *m/z* = 284.98809. Mp. 115–119 °C.

Benzo[*d*]imidazo[2,1-*b*][1,3]iodazol-9-ium triflate (9)

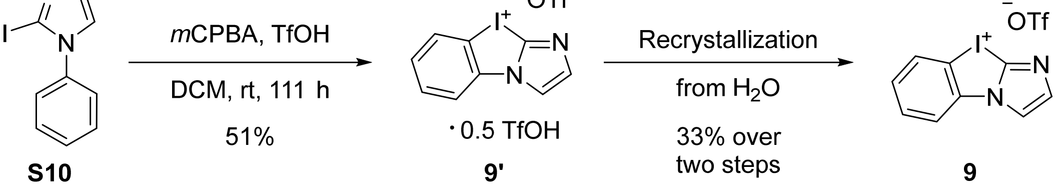

A slightly modified literature procedure was used.[6] 2-Iodo-1-phenyl-1*H*-imidazole (**S10**, 270 mg, 1.00 mmol) and *m*CPBA (85%, 245 mg, 1.20 mmol) were dissolved in DCM (5 mL) and cooled to 0 °C. TfOH (220 µL, 2.50 mmol) was added dropwise and the mixture was stirred for 111 h at room

temperature. Afterwards the mixture was concentrated to ~1 mL, Et_2O (10 mL) was added and stored at 4 °C for 30 min. The precipitate was filtered and washed with Et_2O (2 x 5 mL) to give the desired product with 0.5 equiv additional TfOH (251 mg, 0.509 mmol, 51%). Recrystallization from H_2O gave benzo[*d*]imidazo[2,1-*b*][1,3]iodazol-9-ium triflate (**9**, 140 mg, 0.335 mmol, 33%) as a colorless solid.

^{1}H-NMR (d_6-DMSO, 601 MHz) δ (ppm) 8.69 (s, 1H), 8.29 (d, J = 8.0 Hz, 1H), 8.12 (d, J = 8.3 Hz, 1H), 7.82 (t, J = 7.6 Hz, 1H), 7.63 – 7.38 (m, 2H). ^{13}C NMR (d_6-DMSO, 151 MHz) δ (ppm) 134.8, 134.1, 132.0, 131.0, 128.3, 120.7 (q, J = 322.3 Hz), 120.3, 116.8, 116.6, 113.3. ^{19}F-NMR (d_6-DMSO, 565 MHz) δ (ppm) -77.7. IR (ATR): $\tilde{\nu}$ (cm^{-1}) 3129, 3108, 1481, 1446, 1291, 1214, 1176, 1145, 1022, 748. HR-MS (EI) Calculated for $C_9H_8IN_2^+$ [M+2H-OTf$^-$]$^+$: *m/z* = 270.97267, found: *m/z* = 270.97224. Mp. 220–222 °C (decomp.).

Benzo[*d*]imidazo[5,1-*b*][1,3]iodazol-4-ium (bis)triflate (10a)

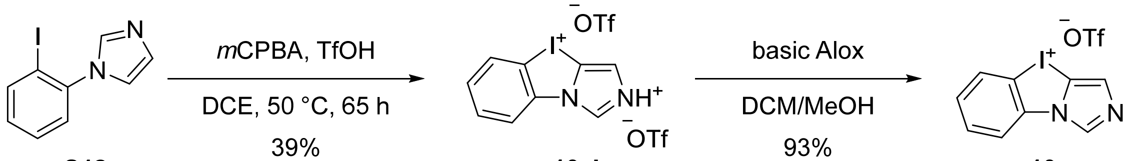

A modified literature procedure was used.[6] 1-(2-Iodophenyl)-1*H*-imidazole (**S12**, 270 mg, 1.00 mmol) and *m*CPBA (85%, 245 mg, 1.20 mmol) were dissolved in DCE (4 mL) and TfOH (221 µL, 2.50 mmol) was added dropwise. The reaction mixture was stirred for 65 h at 50 °C and afterwards Et_2O (10 mL) was added. The mixture was stored at 4 °C for 15 min and the formed precipitate was filtered and washed with Et_2O (2 x 3 mL) to give benzo[*d*]imidazo[5,1-*b*][1,3]iodazol-4-diium bistriflate (**10a’**, 224 mg, 0.394 mmol, 39%) as a colorless solid.

^{1}H-NMR (d_6-DMSO, 601 MHz) δ (ppm) 9.27 (s, 1H), 8.34 (dd, J = 8.1, 1.4 Hz, 1H), 8.09 (dd, J = 8.3, 1.1 Hz, 1H), 7.90 – 7.77 (m, 1H), 7.55 (ddd, J = 8.5, 7.3, 1.3 Hz, 1H), 7.47 (s, 1H). ^{13}C NMR (d_6-DMSO, 151 MHz) δ (ppm) 135.6, 134.7, 132.0, 131.1, 130.5, 129.0, 120.7 (q, J = 322.3 Hz), 117.5, 112.3, 98.2. ^{19}F-NMR (d_6-DMSO, 565 MHz) δ (ppm) -77.8. IR (ATR): $\tilde{\nu}$ (cm^{-1}) 3197, 3109, 3040, 1600, 1571, 1531, 1467, 1369, 1281, 1209, 1159, 1016. HR-MS (ESI) Calculated for $C_9H_8IN_2^+$ [M+H-2OTf$^-$]$^+$: *m/z* = 270.97267, found: *m/z* = 270.97263. Mp. 301-303 °C (decomp.).

A reported literature procedure was used. Benzo[*d*]imidazo[5,1-*b*][1,3]iodazol-4-diium bistriflate (**10a’**, 114 mg, 0.200 mmol) was dissolved in DCM/MeOH (5:1, 1 mL) and submitted to a plug of basic alox (diameter 2 cm, thickness 0.5 cm). The product was eluted with DCM/MeOH (20:1, 100 mL) and removal of the solvent gave benzo[*d*]imidazo[5,1-*b*][1,3]iodazol-4-ium triflate (**10a**, 77.5 mg, 0.185 mmol, 93%) as a colorless solid.

^{1}H-NMR (d_6-DMSO, 601 MHz) δ (ppm) 9.17 (s, 1H), 8.32 (dd, *J* = 8.0, 1.3 Hz, 1H), 8.13 (d, *J* = 8.3 Hz, 1H), 7.81 (t, *J* = 7.7 Hz, 1H), 7.55 – 7.49 (m, 1H), 7.40 (s, 1H). ^{13}C NMR (d_6-DMSO, 151 MHz) δ (ppm) 135.51, 134.82, 131.85, 131.28, 131.20, 128.58, 120.7 (q, *J* = 322.3 Hz) , 117.21, 112.48, 98.83. ^{19}F-NMR (d_6-DMSO, 565 MHz) δ (ppm) -77.8. IR (ATR): $\tilde{\nu}$ (cm^{-1}) 3106, 1605, 1483, 1274, 1232, 1171, 1081, 1022, 915. HR-MS (ESI) Calculated for $C_9H_8IN_2^+$ [M+2H-OTf$^-$]$^+$: *m/z* = 270.97267, found: *m/z* = 270.97239. Mp. 157–160 °C (decomp.).

2-Methylbenzo[*d*]imidazo[5,1-*b*][1,3]iodazole-2,4-diium bistriflate (10b)

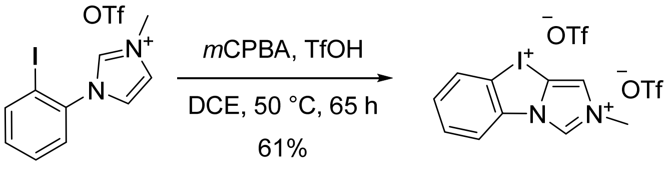

S17 **10b**

A modified literature procedure was used.[6] 1-(2-Iodophenyl)-3-methyl-1*H*-imidazol-3-ium triflate (**S17**, 651 mg, 1.50 mmol) and *m*CPBA (85%, 370 mg, 1.80 mmol) were dissolved in DCE (7.5 mL) and TfOH (325 µL, 3.75 mmol) was added dropwise. The reaction mixture was stirred for 65 h at 50 °C and afterwards Et_2O (5 mL) was added. The mixture was stored at 4 °C for 30 min and the formed precipitate was filtered and washed with Et_2O (2 x 5 mL) to give 2-methylbenzo[*d*]imidazo[5,1-*b*][1,3]iodazole-2,4-diium bistriflate (**10b**, 530 mg, 0.910 mmol, 61%) as a colorless solid.

^{1}H-NMR (d_6-DMSO, 601 MHz) δ (ppm) 10.57 (s, 1H), 8.36 (d, *J* = 8.1 Hz, 1H), 8.18 (d, *J* = 8.3 Hz, 1H), 8.15 (d, *J* = 1.4 Hz, 1H), 7.99 (t, *J* = 7.7 Hz, 1H), 7.77 (t, *J* = 7.9 Hz, 1H), 4.12 (s, 3H). ^{13}C NMR (d_6-DMSO, 151 MHz) δ (ppm) 135.9, 134.3, 132.4, 131.3, 131.2, 126.7, 120.7 (q, *J* = 322.4 Hz), 118.7, 113.8, 103.2, 37.7. ^{19}F-NMR (d_6-DMSO, 565 MHz) δ (ppm) -77.8. IR (ATR): $\tilde{\nu}$ (cm^{-1}) 3175, 3091, 3045, 1540, 1461, 1281, 1218, 1156, 1019, 760. HR-MS (ESI) Calculated for $C_{10}H_{10}IN_2^+$ [M+H-2OTf$^-$]$^+$: *m/z* = 284.98832, found: *m/z* = 284.98823. Mp. 246-248 °C (decomp.).

2-Methylbenzo[*d*]imidazo[5,1-*b*][1,3]iodazole-2,4-diium tetrakis(pentafluorophenyl)borate dietherate complex (10c)

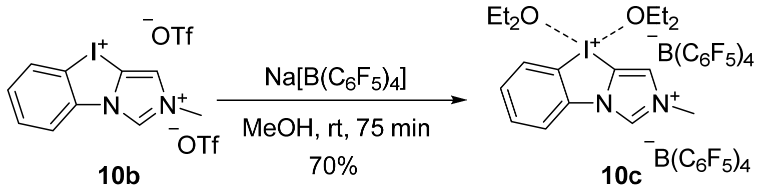

10b **10c**

A modified literature procedure was used.[17] 2-Methylbenzo[*d*]imidazo[5,1-*b*][1,3]iodazole-2,4-diium bistriflate (**10b**, 58.2 mg, 100 µmol) and sodium tetrakis(pentafluorophenyl)borate (141 mg, 200 µmol) were dissolved in dry MeOH (2.5 mL) and stirred at room temperature for 75 min. Afterwards the solvent was removed under reduced pressure at room temperature and the residue

was suspended in DCM (3 mL). The mixture was stirred for 20 min at room temperature, then stored for 30 min at 4 °C and afterwards filtered. *n*-Pentane (10 mL) was added to the filtrate and after decantation, the formed residue was dried *in vacuo*. The residue was suspended in Et_2O (1 mL) and *n*-pentane (10 mL) was added. After decantation and drying *in vacuo* 1-methylbenzo[*d*]pyrazolo[5,1-*b*][1,3]iodazole-1,4-diium tetrakis(pentafluorophenyl)borate (**10c**) was obtained as a dietherate complex (126 mg, 70.4 µmol, 70%) as a colorless solid.

^{1}H-NMR (d_6-DMSO, 601 MHz) δ (ppm) 10.57 (s, 1H), 8.36 (dd, *J* = 8.1, 1.3 Hz, 1H), 8.19 (d, *J* = 8.3 Hz, 1H), 8.14 (s, 1H), 7.99 (ddd, *J* = 8.3, 7.4, 1.2 Hz, 1H), 7.77 (ddd, *J* = 8.6, 7.4, 1.3 Hz, 1H), 4.12 (s, 3H), 3.38 (q, *J* = 7.0 Hz, 8H), 1.09 (t, *J* = 7.0 Hz, 12H). ^{13}C NMR (d_6-DMSO, 151 MHz) δ (ppm) 147.5 (d, *J* = 241.0 Hz), 137.6 (d, *J* = 244.6 Hz), 135.8, 135.6 (d, *J* = 245.1 Hz), 134.3, 132.4, 131.3, 131.1, 126.7, 124.6 – 121.9 (m), 118.7, 113.8, 103.4, 64.9, 37.7, 15.1. ^{19}F-NMR (d_6-DMSO, 565 MHz) δ (ppm) -132.4 (d, *J* = 13.1 Hz, 16F), -161.3 (t, *J* = 21.6 Hz, 8F), -165.9 (t, *J* = 20.6 Hz, 16F). IR (ATR): $\tilde{\nu}$ (cm^{-1}) 3159, 2983, 1644, 1513, 1456, 1374, 1274, 1083, 974. HR-MS (ESI) Calculated for $C_{10}H_{10}IN_2^+$ [M+H-2BAr^{F-}]$^+$: *m/z* = 284.98832, found: *m/z* = 284.98800. Mp. 108–112 °C.

Benzo[4',5']iodolo[3',2':4,5]imidazo[1,2-*a*]pyridin-5-ium triflate (11)

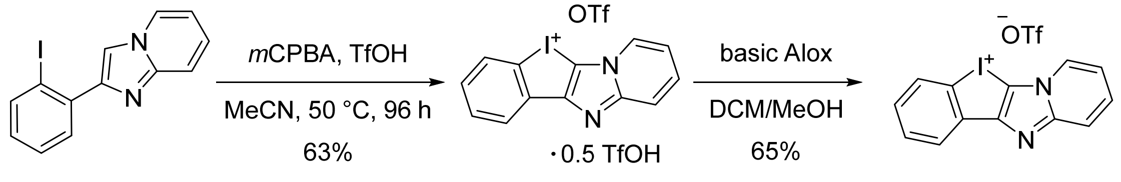

A modified literature procedure was used.[6] 2-(2-Iodophenyl)imidazo[1,2-*a*]pyridine (**S14**, 258 mg, 0.800 mmol) and *m*CPBA (85%, 197 mg, 0.960 mmol) were dissolved in MeCN (4 mL) and TfOH (211 µL, 2.40 mmol) was added dropwise and the mixture was stirred for 96 h at 50 °C. Afterwards the solvent was removed under reduced pressure and the residue was suspended in EtOAc (2 mL) and stored at 4 °C for 30 min. The precipitate was filtered and washed with EtOAc (1 mL) and Et_2O (2 x 3 mL) to give the desired product with 0.5 equiv additional TfOH (275 mg, 0.506 mmol, 63%) as a yellow solid. A fraction of **11a’** (54.0 mg, 0.100 mmol) was dissolved in DCM/MeOH (5:1, 0.5 mL) and submitted to a plug of basic alox (diameter 1 cm, thickness 0.5 cm). The product was eluted with DCM/MeOH (20:1, 50 mL) and removal of the solvent gave benzo[4',5']iodolo[3',2':4,5]imidazo[1,2-*a*]pyridin-5-ium triflate (**11a**, 30.4 mg, 64.9 µmol, 65%) as a yellowish solid.

^{1}H-NMR (d_6-DMSO, 601 MHz) δ (ppm) 9.02 (d, *J* = 6.8 Hz, 1H), 8.43 (d, *J* = 8.4 Hz, 1H), 8.21 (d, *J* = 7.0 Hz, 1H), 7.87 – 7.71 (m, 2H), 7.59 (t, *J* = 7.5 Hz, 1H), 7.44 (t, *J* = 7.8 Hz, 1H), 7.12 (t, *J* = 7.0 Hz, 1H). ^{13}C NMR (d_6-DMSO, 151 MHz) δ (ppm) 153.2, 149.7, 132.0, 131.2, 130.8, 130.6, 128.1, 127.6, 127.5, 126.2, 120.7 (q, *J* = 322.2 Hz), 117.9, 113.9, 92.9. ^{19}F-NMR (d_6-DMSO, 565 MHz) δ (ppm) -77.8. IR (ATR): $\tilde{\nu}$ (cm^{-1}) 3063, 2918, 1603, 1488, 1258, 1233, 1170, 1037, 1026, 993. HR-

MS (EI) Calculated for $C_{13}H_{10}IN_2^+$ [M+2H-OTf$^-$]$^+$: *m/z* = 320.98832, found: *m/z* = 320.98806. Mp. 175–178 °C (decomp.).

11-Methylbenzo[4',5']iodolo[3',2':4,5]imidazo[1,2-*a*]pyridine-5,11-diium bistriflate (11b)

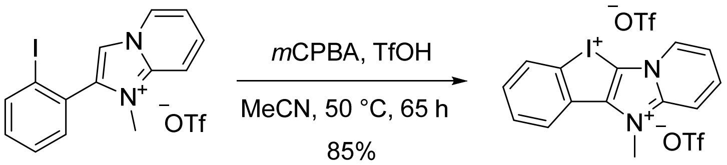

S18 **11b**

A modified literature procedure was used.[6] 2-(2-Iodophenyl)-1-methylimidazo[1,2-*a*]pyridin-1-ium triflate (**S18**, 968 mg, 2.00 mmol) and *m*CPBA (85%, 493 mg, 2.40 mmol) were dissolved in MeCN (10 mL) and TfOH (443 µL, 5.00 mmol) was added dropwise. The reaction mixture was stirred for 65 h at 50 °C and afterwards Et_2O (5 mL) was added. The mixture was stored at 4 °C for 30 min and the formed precipitate was filtered and washed with Et_2O (2 x 5 mL) to give 11-methylbenzo[4',5']iodolo[3',2':4,5]imidazo[1,2-*a*]pyridine-5,11-diium bistriflate (**11b**, 1.07 g, 1.69 mmol, 85%) as a colorless solid.

^{1}H-NMR (*d*$_3$-MeOD, 601 MHz) δ (ppm) 9.21 (d, *J* = 6.8 Hz, 1H), 8.73 (dd, *J* = 7.9, 1.5 Hz, 1H), 8.48 (d, *J* = 9.3 Hz, 1H), 8.42 – 8.39 (m, 1H), 8.30 (ddd, *J* = 9.2, 7.1, 1.2 Hz, 1H), 8.07 (t, *J* = 7.6 Hz, 1H), 7.90 (ddd, *J* = 8.7, 7.4, 1.5 Hz, 1H), 7.82 (dd, *J* = 7.0, 1.0 Hz, 1H), 4.59 (s, 3H). ^{13}C NMR (*d*$_3$-MeOD, 151 MHz) δ (ppm) 146.3, 146.0, 137.6, 134.2, 133.3, 132.3, 130.9, 129.8, 128.9, 127.4, 121.7 (q, *J* = 318.6 Hz), 120.0, 113.1, 97.2, 34.5. ^{19}F-NMR (*d*$_3$-MeOD, 565 MHz) δ (ppm) -80.1. IR (ATR): $\tilde{\nu}$ (cm^{-1}) 3095, 1498, 1482, 1458, 1278, 1215, 1151, 1020, 756. HR-MS (ESI) Calculated for $C_{14}H_{12}IN_2^+$ [M+H-2OTf$^-$]$^+$: *m/z* = 335.00397, found: *m/z* = 335.00385. Mp. 255-257 °C (decomp.).

11-Methylbenzo[4',5']iodolo[3',2':4,5]imidazo[1,2-*a*]pyridine-5,11-diium bis(tetrakis(3,5-bis(trifluoromethyl)phenyl)borate) etherate complex (11c)

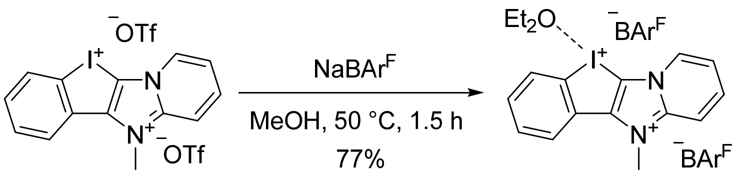

11b **11c**

A modified literature procedure was used.[17] 11-Methylbenzo[4',5']iodolo[3',2':4,5]imidazo[1,2-*a*]pyridine-5,11-diium bistriflate (**11b**, 63.2 mg, 100 µmol) and sodium tetrakis(3,5-bis(trifluoromethyl)phenyl)borate (177 mg, 200 µmol) were dissolved in dry MeOH (2.5 mL) and stirred at 50 °C for 1.5 h. Afterwards the solvent was removed under reduced pressure and the residue was suspended in DCM (3 mL). The mixture was stirred for 20 min at room temperature and was then stored for 30 min at 4 °C and afterwards filtered. *n*-Pentane (10 mL) was added to the filtrate and after decantation, the formed residue was dried *in vacuo* to give 11-

methylbenzo[4',5']iodolo[3',2':4,5]imidazo[1,2-*a*]pyridine-5,11-diium bis(tetrakis(3,5-bis(tri-fluoromethyl)phenyl)borate) as a mono-MeOH complex (175 mg, 81.1 µmol, 81%) as a yellowish solid. This was suspended in Et_2O (2 mL) and *n*-pentane (10 mL) was added. After decantation and drying *in vacuo* 11-methylbenzo[4',5']iodolo[3',2':4,5]imidazo[1,2-*a*]pyridine-5,11-diium bis(tetrakis(3,5-bis(trifluoromethyl)phenyl)borate) (**11c**) was obtained as a mono-Et_2O complex (165 mg, 77.3 µmol, 77%) as a colorless solid.

^{1}H-NMR (d_6-DMSO, 601 MHz) δ (ppm) 9.38 (d, J = 6.8 Hz, 1H), 8.69 (dd, J = 8.0, 1.5 Hz, 1H), 8.59 (d, J = 9.3 Hz, 1H), 8.53 – 8.41 (m, 1H), 8.38 – 8.25 (m, 1H), 8.06 – 7.99 (m, 1H), 7.88 (ddd, J = 8.7, 7.3, 1.5 Hz, 1H), 7.85 (t, J = 6.9 Hz, 1H), 7.73 (s, 8H), 7.67 – 7.53 (m, 16H), 4.54 (s, 3H), 3.38 (q, J = 7.0 Hz, 4H), 1.09 (t, J = 7.0 Hz, 6H). ^{13}C NMR (d_6-DMSO, 151 MHz) δ (ppm) 160.9 (dd, J = 99.7, 49.8 Hz), 144.0, 143.5, 135.8, 134.0, 132.6, 131.5, 130.1, 128.5 (dq, J = 31.7, 2.7 Hz), 128.2, 127.1, 126.9, 124.0 (q, J = 272.5 Hz), 118.2, 117.66, 112.1, 99.2, 64.9, 33.9, 15.1. ^{19}F-NMR (d_6-DMSO, 565 MHz) δ (ppm) -61.7. IR (ATR): $\tilde{\nu}$ (cm^{-1}) 3100, 1610, 1520, 1354, 1273, 1112, 886, 838. HR-MS (ESI) Calculated for $C_{14}H_{12}IN_2^+$ [M+H-2BArF$^-$]$^+$: *m/z* = 335.00397, found: *m/z* = 335.00346. Mp. 65–68 °C.

Dibenzo[*b*,*d*]iodol-5-ium triflate (4a)

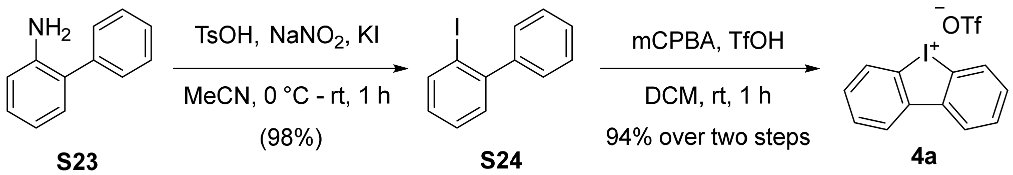

S23 **S24** **4a**

A reported literature procedure was used.[6] *p*-TsOH monohydrate (1.71 g, 9.00 mmol) was dissolved in MeCN (12 mL) and [1,1'-biphenyl]-2-amine (**S23**, 508 mg, 3.00 mmol) was added. The mixture was cooled to 0 °C and $NaNO_2$ (414 mg, 6.00 mmol) in H_2O (1 mL) was added dropwise over 2 min. Afterwards KI (1.25 g, 7.50 mmol) in H_2O (1 mL) was added dropwise over 5 min and stirring was continued for 5 min at 0 °C and for 1 h at room temperature. H_2O (40 mL) was added, the pH was adjusted to ~9 with sat. $NaHCO_3$-sol. and sat. $Na_2S_2O_3$-sol. (10 mL) was added afterwards. The mixture was extracted with EtOAc (2 x 50 mL), the combined organic phases were dried over Na_2SO_4, filtered and concentrated under reduced pressure. Crude 2-iodo-1,1'-biphenyl (**S24**, 824 mg, 2.94 mmol, corresponding to 98% yield) was directly dissolved in DCM (8 mL) and *m*CPBA (85%, 766 mg, 3.75 mmol) was added followed by the dropwise addition of TfOH (792 µL, 9.00 mmol). The mixture was stirred at room temperature for 1 h before the solvent was removed *in vacuo*. The residue was suspended in Et_2O (8 mL), stirred vigorously for 10 min and was stored at 4 °C for 15 min. The formed precipitate was filtered and washed with additional Et_2O (3 x 2 mL) to give dibenzo[*b*,*d*]iodol-5-ium triflate (**4a**, 1.21 g, 2.82 mmol, 94% over two steps) as an "off-white" solid.

^{1}H-NMR (d_6-DMSO, 601 MHz) δ (ppm) 8.48 (d, J = 7.8 Hz, 2H), 8.22 (d, J = 8.2 Hz, 2H), 7.86 (t, J = 7.5 Hz, 2H), 7.72 (t, J = 7.7 Hz, 2H). ^{13}C NMR (d_6-DMSO, 151 MHz) δ (ppm) 141.7, 131.1, 130.7, 130.6, 127.0, 121.6, 120.7 (q, J = 322.2 Hz). ^{19}F-NMR (d_6-DMSO, 565 MHz) δ (ppm) -77.7. IR (ATR): $\tilde{\nu}$ (cm^{-1}) 3102, 1456, 1443, 1425, 1275, 1243, 1222, 1153, 1024, 748. MS (APCI) *m/z* = 279.1 [M-TfO^{-}]$^{+}$. Mp. 238–242 °C. Analytical data is in accordance with literature data.[6]

Dibenzo[*b*,*d*]iodol-5-ium tetrakis(3,5-bis(trifluoromethyl)phenyl)borate (4b)

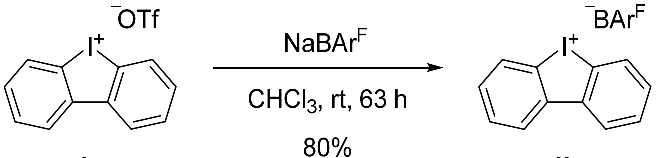

4a **4b**

A slightly modified literature procedure was used.[16] Dibenzo[*b*,*d*]iodol-5-ium triflate (**4a**, 129 mg, 0.300 mmol) and sodium tetrakis(3,5-bis(trifluoromethyl)phenyl)borate (266 mg, 0.300 mmol) were dissolved in $CHCl_3$ (6 mL) and stirred at room temperature for 63 h. Afterwards the reaction mixture was stored for 2 h at 4 °C, filtered and *n*-pentane (10 mL) was added to the filtrate. The formed precipitate was filtered and dried *in vacuo* to give dibenzo[*b*,*d*]iodol-5-ium tetrakis(3,5-bis(trifluoromethyl)phenyl)borate (**4b**, 275 mg, 0.241 mmol, 80%) as a yellow solid.

^{1}H-NMR ($CDCl_3$, 601 MHz) δ (ppm) 8.10 (dd, J = 7.9, 1.5 Hz, 2H), 7.83 (ddd, J = 8.1, 6.3, 2.0 Hz, 2H), 7.78 – 7.68 (m, 8H), 7.66 – 7.57 (m, 4H), 7.50 (s, 4H). ^{13}C-NMR ($CDCl_3$, 151 MHz): δ (ppm) 161.7 (dd, J = 99.5, 49.8 Hz), 141.5, 134.7, 133.3, 132.5, 130.1, 129.3 – 129.0 (m), 128.8, 124.4 (q, J = 272.7 Hz), 121.4, 118.0 – 117.3 (m). ^{19}F-NMR ($CDCl_3$, 565 MHz): δ (ppm) –62.3. IR (ATR): $\tilde{\nu}$ (cm^{-1}) 3092, 1610, 1456, 1353, 1272, 1112, 1004, 886, 838. MS (APCI) *m/z* = 279.1 [M-$BArF^{-}$]$^{+}$. Mp. 120–123 °C. Analytical data is in accordance with literature data.[16]

5. Starting materials for the XB-mediated reactions

α-Methylbenzyl chloride

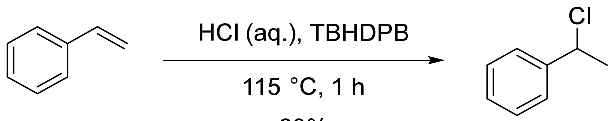

S25 **14**

A slightly modified literature procedure was used.[18] Styrene (**S25**, 1.04 g, 10.0 mmol), conc. aq. HCl (4.2 mL, 50.0 mmol) and tributylhexadecylphosphonium bromide (510 mg, 1.00 mmol) were added to a 25 mL round-bottom flask and the mixture was stirred at 115 °C for 1 h. After cooling to room temperature, the mixture was extracted with DCM (2 x 5 mL). The combined organic phases were dried over Na_2SO_4, filtered and concentrated under reduced pressure. The residue was dissolved in cyclohexane (5 mL) and filtered over a plug of silica (width x height = 2 cm x 2 cm) and

eluted with further cyclohexane (30 mL). The solvent was removed under reduced pressure and the residue was further dried (30 mbar, 45 °C) to give α-methylbenzyl chloride (**14**, 887 mg, 6.31 mmol, 63%) as a colorless liquid.

^{1}H-NMR ($CDCl_3$, 601 MHz) δ (ppm) 7.47 – 7.41 (m, 2H), 7.40 – 7.34 (m, 2H), 7.33 – 7.28 (m, 1H), 5.11 (q, *J* = 6.8 Hz, 1H), 1.86 (d, *J* = 6.8 Hz, 3H). ^{13}C-NMR ($CDCl_3$, 151 MHz): δ (ppm) 142.8, 128.6, 128.2, 126.5, 58.8, 26.5. IR (ATR): $\tilde{\nu}$ (cm^{-1}) 3064, 3030, 2976, 2927, 1493, 1454, 1376, 1231, 1048, 1026, 969. MS (EI) *m/z* = 140.08 $[M]^{+\bullet}$. Analytical data is in accordance with literature data.[19]

***N*-(Prop-2-yn-1-yl)benzamide (17)**

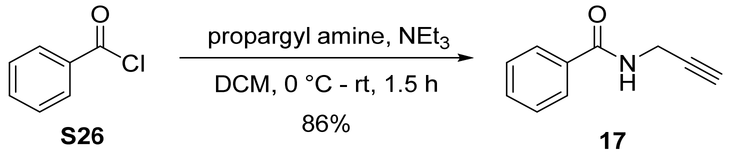

A slightly modified literature procedure was used.[20] Benzoyl chloride (**S26**, 581 µL, 5.00 mmol) was dissolved in dry DCM (10 mL) and propargyl amine (352 µL, 5.50 mmol) followed by NEt_3 (836 µL, 6.00 mmol) were added at 0 °C. After stirring for 1.5 h at room temperature, the reaction mixture was poured into 1 M HCl (25 mL). The mixture was extracted with DCM (2 x 15 mL) and the combined organic phases were washed with brine (25 mL), dried over Na_2SO_4, filtered and the solvent was removed under reduced pressure to give *N*-(prop-2-yn-1-yl)benzamide (**17**, 681 mg, 4.28 mmol, 86%) as a colorless solid.

^{1}H-NMR ($CDCl_3$, 601 MHz) δ (ppm) 7.84 – 7.73 (m, 2H), 7.55 – 7.48 (m, 1H), 7.48 – 7.41 (m, 2H), 6.31 (s, 1H), 4.26 (dd, *J* = 5.2, 2.5 Hz, 2H), 2.29 (t, *J* = 2.6 Hz, 1H). ^{13}C-NMR ($CDCl_3$, 151 MHz): δ (ppm) 167.2, 133.7, 131.7, 128.5, 127.0, 79.5, 71.7, 29.7. IR (ATR): $\tilde{\nu}$ (cm^{-1}) 3289, 3059, 2930, 1638, 1602, 1538, 1488, 1413, 1307, 1262, 1047. MS (EI) *m/z* = 159.09 $[M]^{+\bullet}$. Mp. 109–110 °C. Analytical data is in accordance with literature data.[20]

***trans*-β-Crotonophenone (25)**

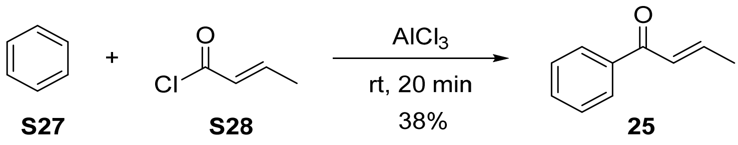

A slightly modified literature procedure was used.[21] $AlCl_3$ (3.40 g, 25.5 mmol) was suspended in benzene (13 mL) under an atmosphere of argon. Crotonic acid chloride (**S28**, 2.09 g, 20.0 mmol) was added over the course of 5 min under vigorous stirring resulting in a clear solution. After 20 min the mixture was added to a mixture of ice water and 1 M HCl (75 mL each) and extracted with DCM (3 x 30 mL). The combined organic phases were washed with 1 M NaOH (50 mL), dried over Na_2SO_4, filtered and concentrated under reduced pressure. The residue was purified by distillation

(10 mbar, 135 °C oil bath temperature) to give β-*trans*-crotonophenone (**25**, 1.76 g, 12.0 mmol, 60%, 95% purity) as a colorless liquid. The product was further purified via column chromatography on silica (cyclohexane/EtOAc 30:1) to give pure *trans*-β-crotonophenone (**25**, 1.12 g, 7.66 mmol, 38%) as a colorless liquid.

^{1}H-NMR ($CDCl_3$, 601 MHz) δ (ppm) 7.99 – 7.84 (m, 2H), 7.64 – 7.51 (m, 1H), 7.52 – 7.43 (m, 2H), 7.07 (dq, *J* = 15.3, 6.8 Hz, 1H), 6.91 (dq, *J* = 15.3, 1.6 Hz, 1H), 2.00 (dd, *J* = 6.9, 1.7 Hz, 3H). ^{13}C-NMR ($CDCl_3$, 151 MHz): δ (ppm) 190.7, 145.0, 137.9, 132.5, 128.5 127.5, 18.6. IR (ATR): $\tilde{\nu}$ (cm^{-1}) 3057, 2971, 2939, 1668, 1622, 1447, 1331, 1293, 1218, 963. MS (EI) *m/z* = 146.06 $[M]^{+\bullet}$. Analytical data is in accordance with literature data.[21]

(*E*)-(2-Nitrovinyl)benzene (28)

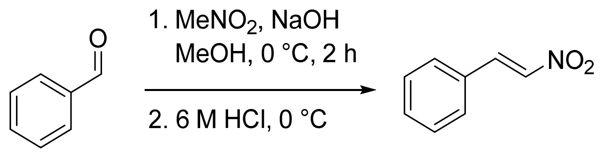

S29 **28**

A slightly modified literature procedure was used.[6] Benzaldehyde (**S29**, 531 mg, 5.00 mmol) and nitromethane (0.67 mL, 12.5 mmol) were dissolved in MeOH (15 mL) and aqueous 10% NaOH solution (4.5 mL, 12.5 mmol) was added at 0 °C. The reaction mixture was stirred at 0 °C for 2 h and was then slowly transferred to 6 M HCl (20 mL) under vigorous stirring at 0 °C. The formed precipitate was filtered, washed with H_2O (30 mL) and dried *in vacuo* to give (*E*)-(2-nitrovinyl)benzene (**28**, 652 mg, 4.37 mmol, 87%) as a yellowish solid.

^{1}H-NMR ($CDCl_3$, 601 MHz) δ (ppm) 8.01 (d, *J* = 13.7 Hz, 1H), 7.59 (d, *J* = 13.7 Hz, 1H), 7.57 – 7.53 (m, 2H), 7.53 – 7.49 (m, 1H), 7.49 – 7.42 (m, 2H). ^{13}C-NMR ($CDCl_3$, 151 MHz): δ (ppm) 139.0, 137.1, 132.1, 130.0, 129.4, 129.1. IR (ATR): $\tilde{\nu}$ (cm^{-1}) 3109, 3044, 2827, 1630, 1577, 1511, 1494, 1448, 1338, 1263, 967. MS (EI) *m/z* = 149.08 $[M]^{+\bullet}$. Mp. 57–58 °C. Analytical data is in accordance with literature data.[22]

6. XB-mediated reactions

All NMR-solvents were stored over activated 4 Å molecular sieves and under an atmosphere of argon in Schlenk tubes.

Ritter-type halide abstractions

Benzhydryl chloride

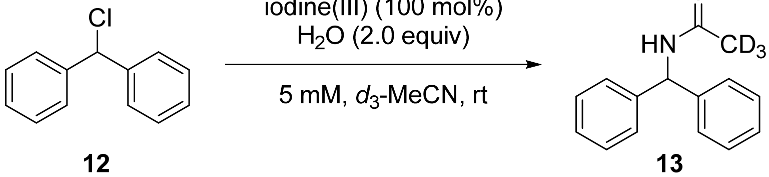

12 **13**

A slightly modified literature procedure was used.[16] To an NMR-tube was added a 20 mM stock solution of the corresponding XB-donor in d_3-MeCN (150 µL, 3.00 µmol), followed by d_3-MeCN (150 µL) and a 10 mM stock solution of benzhydryl chloride (**12**, BHC) in d_3-MeCN incl. 2.0 equiv. H_2O (300 µL, 3.00 µmol). The NMR-tube was sealed, shaken and afterwards time-dependent ^{1}H-NMR measurements were conducted (8 scans, d_1 = 15 s, suitable for integration, an error margin of 5% is assumed). Important measurements were conducted at least twice.

The yields were determined by integration of the benzylic proton signals of the starting material (6.29 ppm, s), the product (6.25 ppm, d, J = 7.9 Hz), and activated/intermediary species or benzhydrol (5.77 ppm, s; when benzhydrol is present a broad singlet at 3.80 ppm is also observed for the OH-group) and a possible side product (5.45 ppm, s). In some reactions the formation of another intermediate is observed as a singlet at 5.42 ppm. The sum of these integrals was set to 1 and the integrals for each signal directly gave the corresponding yield.

Initial rate constants (k_{rel}) were determined by a linear plot (intersection with the zero point) using the first two measurement points after 0.5 h and 1.5 h. (Due to the high reactivity of **8c** only the first measurement point was used, as the second was already far from being linear.)

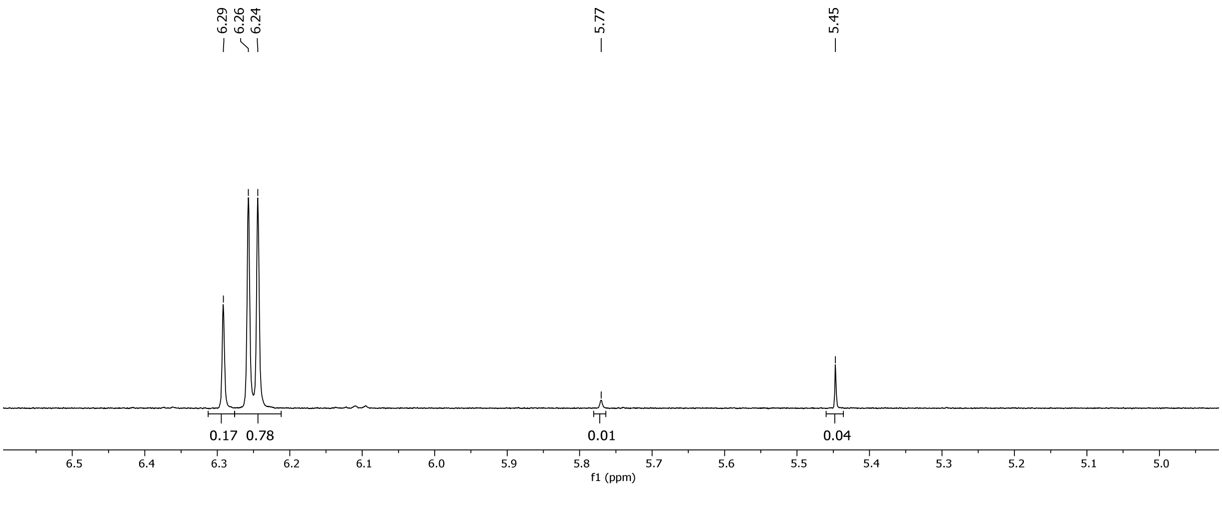

*Figure S1: 600 MHz 1H-NMR spectrum for the Ritter type solvolysis of benzhydryl chloride (**12**) in d_3-MeCN after 39 h employing 2-methyl-1-phenyl-1H-benzo[4,5]iodolo[3,2-c]pyrazole-2,4-diium bistriflate **7e** as the XB-donor. The spectrum is zoomed in to show the relevant signals for the reaction progress.*

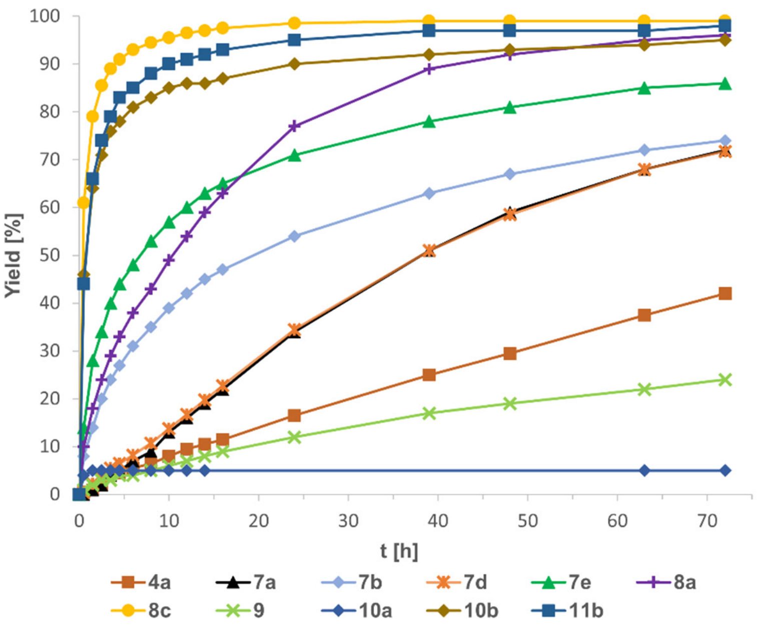

*Figure S2: Yield-vs.-time profile for the Ritter-type solvolysis of benzhydryl chloride (**12**) over the course of 72 h.*

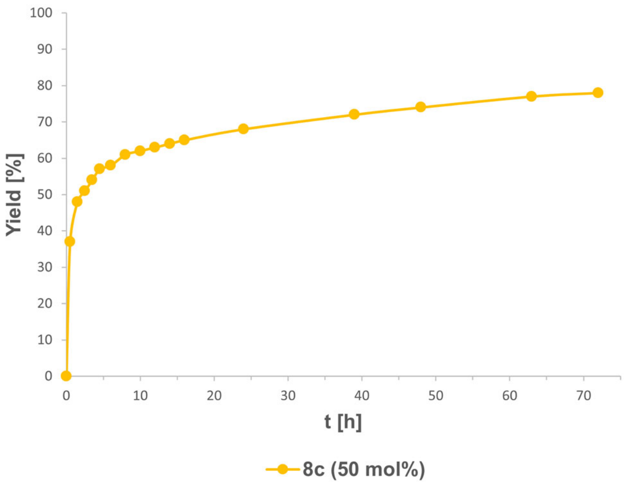

*Figure S3: Yield-vs.-time profile for the Ritter-type solvolysis of benzhydryl chloride (**12**) over the course of 72 h employing XB-donor **8c** in 50 mol%*

α-Methylbenzyl chloride

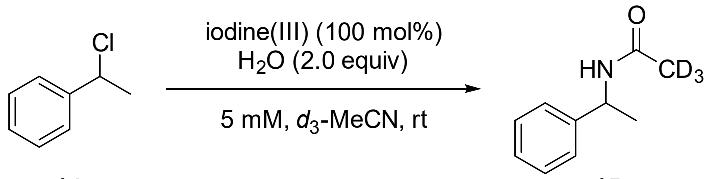

14 **15**

To an NMR-tube was added a 20 mM stock solution of the corresponding XB-donor in d_3-MeCN (150 µL, 3.00 µmol) (or weighed in directly into the NMR-tube + 150 µL d_3-MeCN), followed by d_3-MeCN (150 µL) and a 10 mM stock solution of α-methylbenzyl chloride (**14**) in d_3-MeCN incl. 2.0

equiv. H_2O (300 µL, 3.00 µmol). The NMR-tube was sealed, shaken and afterwards time-dependent ^{1}H-NMR measurements were conducted (8 scans, d_1 = 15 s, suitable for integration, an error margin of 5% is assumed). Important measurements were conducted at least twice.

The yields were determined by integration of the benzylic proton signals of the starting material (5.21 ppm, q, J = 6.8 Hz), the product (5.12 ppm, quint, J = 7.2 Hz) and an activated/intermediary species (4.79 ppm, q, J = 6.4 Hz). The sum of these integrals was set to 1 and the integrals for each signal directly gave the corresponding yield.

Additionally, the conversion of the starting material into the product can be observed by the consumption of the methyl group at 1.82 ppm (d, J = 6.9 Hz) and the formation of the methyl group of the product at 1.55 ppm (d, 7.0 Hz) and the formation and consumption of the methyl group of the intermediate at 1.38 ppm (d, 6.5 Hz).

Initial rate constants (k_{rel}) were determined by a linear plot (intersection with the zero point) using the first two measurement points after 0.5 h and 1.5 h.

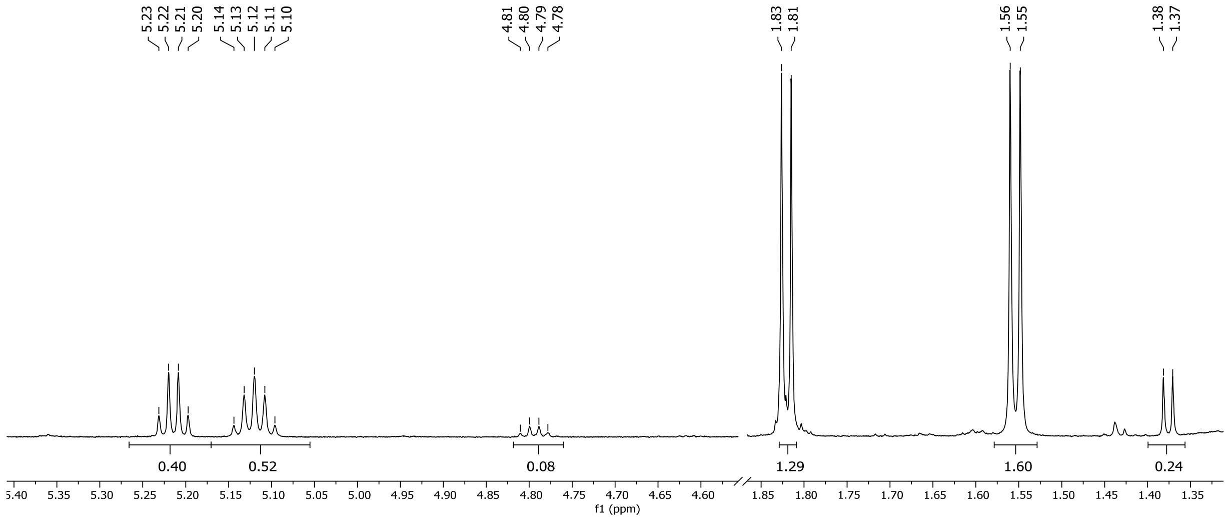

*Figure S4: 600 MHz 1H-NMR spectrum for the Ritter type solvolysis of a-methylbenzyl chloride (**14**) in d_3-MeCN after 10 h employing 11-methylbenzo[4',5']iodolo[3',2':4,5]imidazo[1,2-a]pyridine-5,11-diium bistriflate **11b** as the XB-donor. The spectrum is zoomed in to show the relevant signals for the reaction progress.*

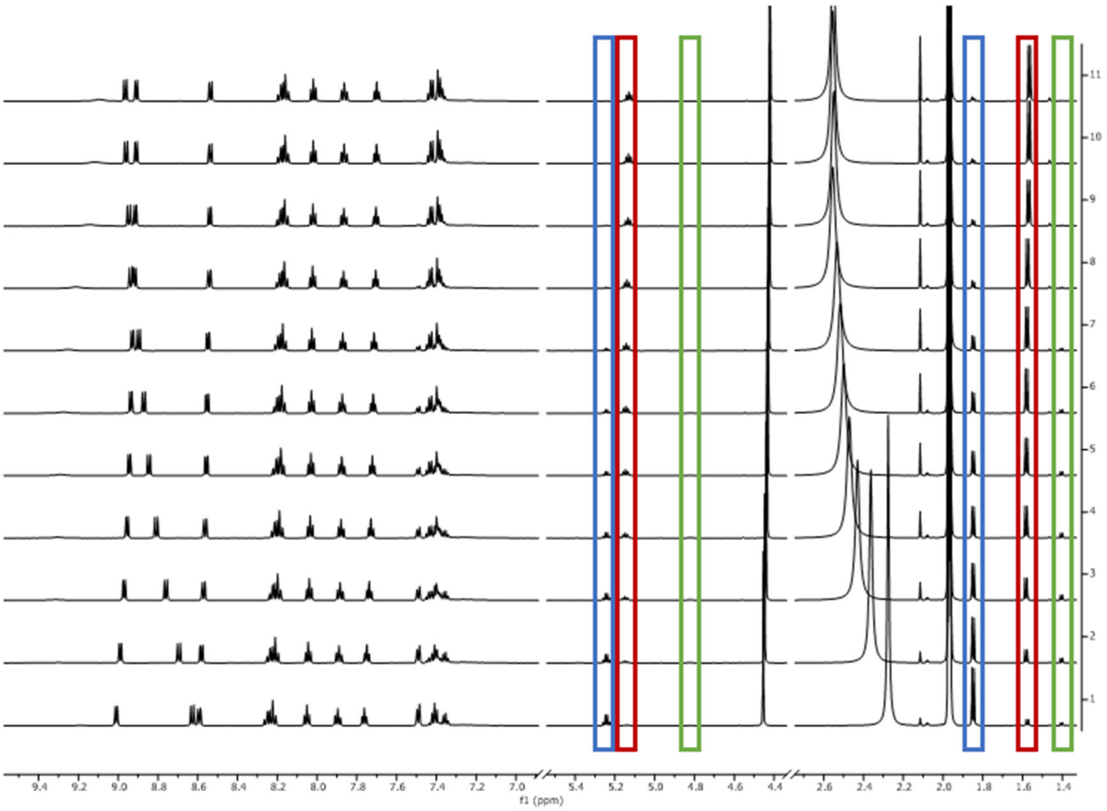

*Figure S5: 600 MHz ^{1}H-NMR spectra for the Ritter type solvolysis of α-methylbenzyl chloride (**14**) in d_3-MeCN over the course of 72 h employing 11-methylbenzo[4',5']iodolo[3',2':4,5]imidazo[1,2-a]pyridine-5,11-diium bistriflate **11b** as the XB-donor. In blue: important signals of the starting material; in red: important signals of the product; in green: important signals of the intermediate.*

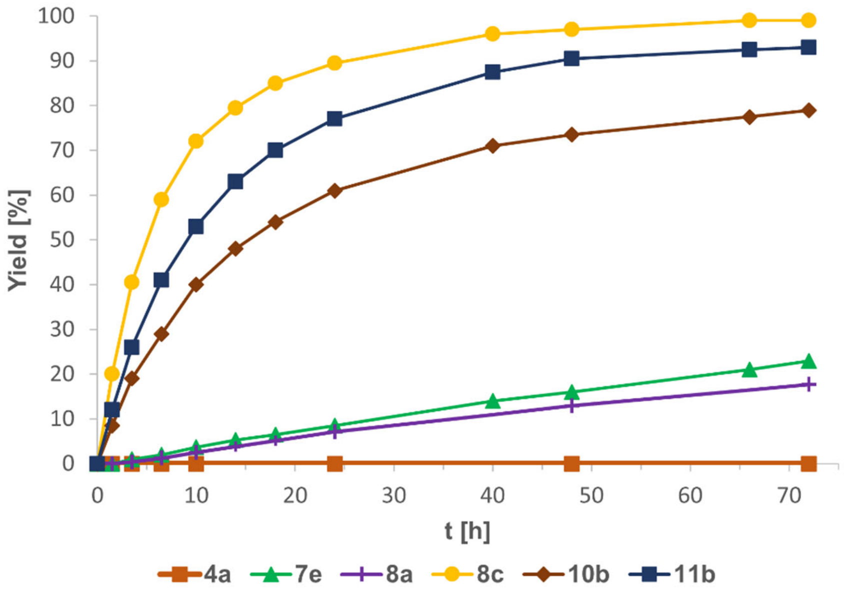

Figure S6: Yield-vs.-time profile *for the Ritter-type solvolysis of α-methylbenzyl chloride (**14**) over the course of 72 h.*

Gold(I)-catalyzed cyclization of propargylic amide 17

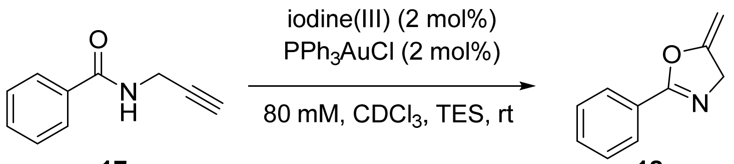

17 **18**

A reported literature procedure was followed.[23] To an NMR-tube was added a 8 mM stock solution of the corresponding XB-donor in $CDCl_3$ (100 µL, 0.80 µmol, 2 mol%) (or weighed in directly into the NMR-tube + 100 µL $CDCl_3$), a 133 mM stock solution of propargylic amide (**17**) in $CDCl_3$ (300 µL, 40.0 µmol, incl. 0.25 equiv. TES) and a 8 mM stock solution of PPh_3AuCl (100 µL, 0.8 µmol, 2 mol%). The NMR-tube was sealed, shaken and afterwards time-dependent ^{1}H-NMR measurements were conducted (8 scans, d_1 = 15 s, suitable for integration, an error margin of 5% is assumed). Important measurements were conducted at least twice.

As the oxazoline **18** slowly transforms into the oxazole (up to 4% after 14 h), the conversion of amide **17** into oxazoline **18** was determined instead of the absolute yield of **18**. The conversion of **17** was determined by integration of the C2-unit of the starting material (4.26 ppm, dd, *J* = 5.2, 2.6 Hz) in comparison with ethyl peak of TES (0.50 ppm, q, *J* = 7.9 Hz) which was set to 1. For a double check, the C2-unit of the product **18** (4.65 ppm, t, *J* = 2.9 Hz) was also integrated and the TES signal slightly corrected if necessary.

Further more or less integratable signals of **17** are the alkyne proton (2.28 ppm, t, *J* = 2.6 Hz) and the amide NH (6.34 ppm, s) and of **18** the protons of the exocyclic double bond (4.38 ppm, q, *J* = 2.7 Hz and 4.83 ppm, q, *J* = 3.0 Hz).

Initial rate constants (k_{rel}) were determined by a linear plot (intersection with the zero point) using the first two measurement points after 0.5 h and 1.5 h.

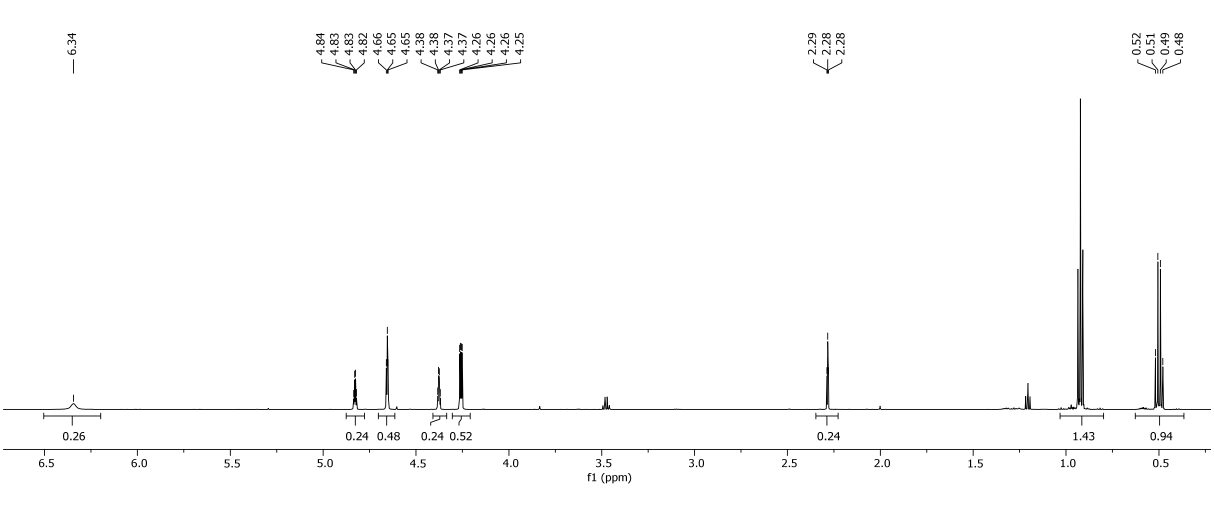

*Figure S7: 600 MHz ^{1}H-NMR spectrum for the gold(I)-catalyzed cyclization of propargylic amide **17** in $CDCl_3$ after 30 min employing 1-methylbenzo[d]pyrazolo[5,1-b][1,3]iodazole-1,4-diium tetrakis(pentafluorophenyl)borate dietherate complex (**8d**) as the XB-donor. The spectrum is zoomed in to show the relevant signals for the reaction progress.*

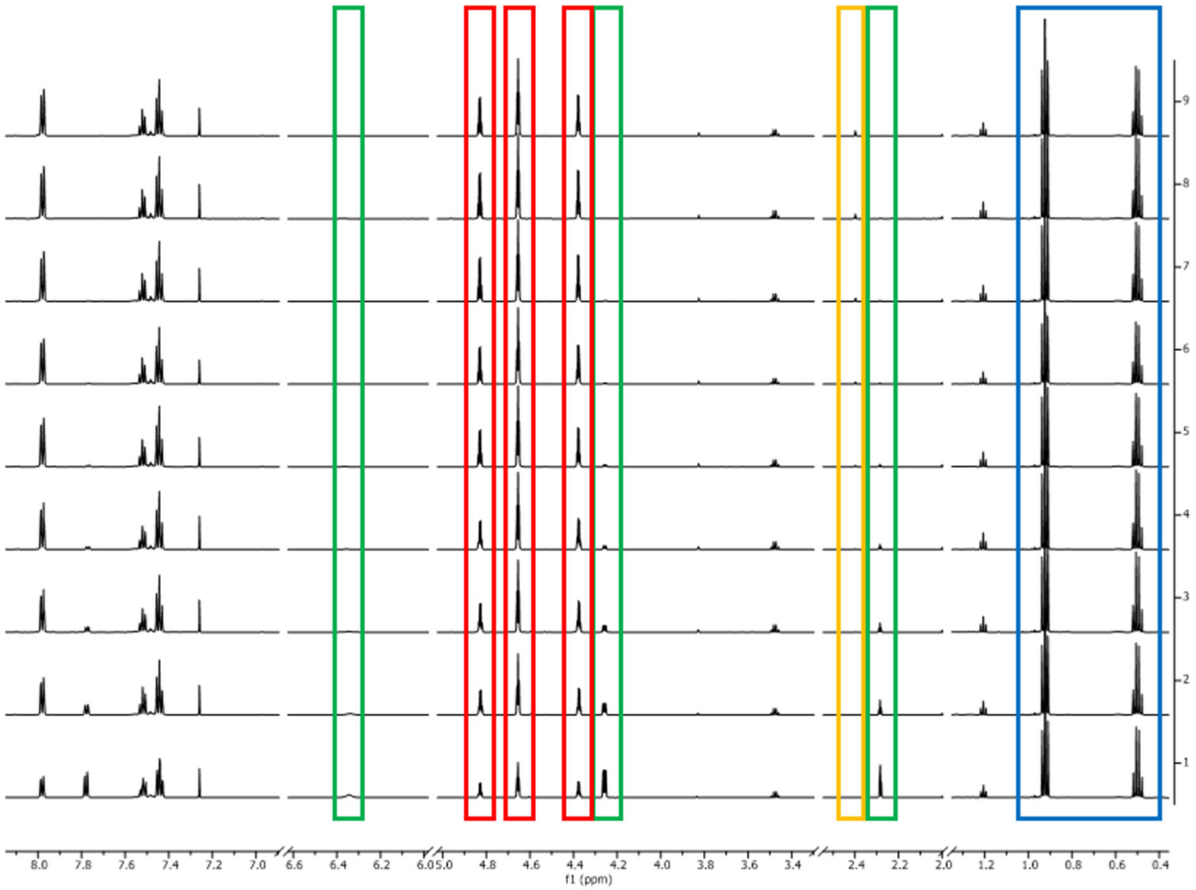

*Figure S8: 600 MHz ¹H-NMR spectra for the gold(I)-catalyzed cyclization of propargylic amide **17** in $CDCl_3$ over the course of 10 h employing 1-methylbenzo[d]pyrazolo[5,1-b][1,3]iodazole-1,4-diium tetrakis(pentafluorophenyl)borate dietherate complex (**8d**) as the XB-donor. In blue: TES as the internal standard; in green: important signals of the starting material; in red: important signals of the product; in orange: emerging oxazole.*

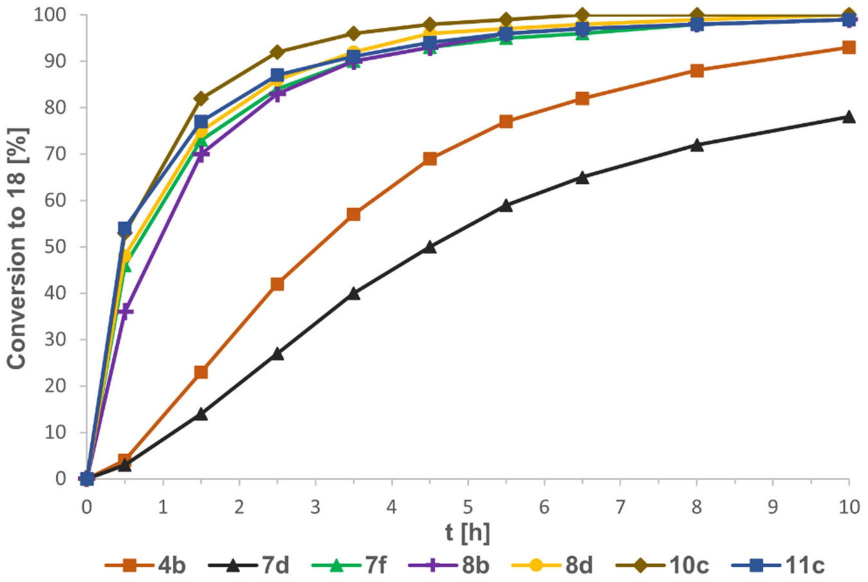

*Figure S9: Conversion-vs.-time profile for the gold(I)-catalyzed cyclization of propargylic amide **17** in the presence of different cyclic iodonium salts as the activators over the course of 10 h.*

Diels-Alder reactions

Between CPD and MVK

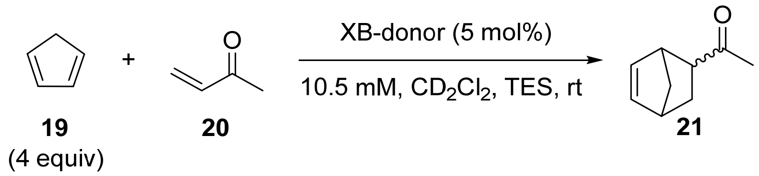

A modified literature procedure was used.[16] To an NMR-tube were added a 12.6 mM stock solution of the corresponding XB-donor in CD_2Cl_2 (25 µL, 0.315 µmol, 5 mol%) and a 252 mM stock solution of freshly cracked cyclopentadiene (**19**) in CD_2Cl_2 (100 µL, 25.2 µmol, 4.0 equiv.), followed by CD_2Cl_2 (455 µL). Then followed the addition a 630 mM stock solution of freshly distilled methyl vinyl ketone (**20**, MVK) incl. 0.25 equiv. of tetraethyl silane (TES) as the internal standard (10 µL, 6.30 µmol). The tube was sealed, shaken and afterwards time-dependent ^{1}H-NMR measurements were conducted (8 scans, d_1 = 15 s, suitable for integration, an error margin of 5% is assumed). Important measurements were conducted at least twice.

The yields were determined as follows: The integral of the ethyl peak of TES (0.51 ppm, q, J = 7.9 Hz) was calibrated to 0.67 and/or the integral of the methyl peak of TES (0.93 ppm, t, J = 7.9 Hz) was calibrated to 1. Afterwards the yields were determined by integration of the methyl groups of the starting material (2.26 ppm, s), the *exo* product (2.19 ppm, s) and the *endo* product (2.11 ppm, s).

Initial rate constants (k_{rel}) were determined by a linear plot (intersection with the zero point) using the first two measurement points after 10 min and 30 min.

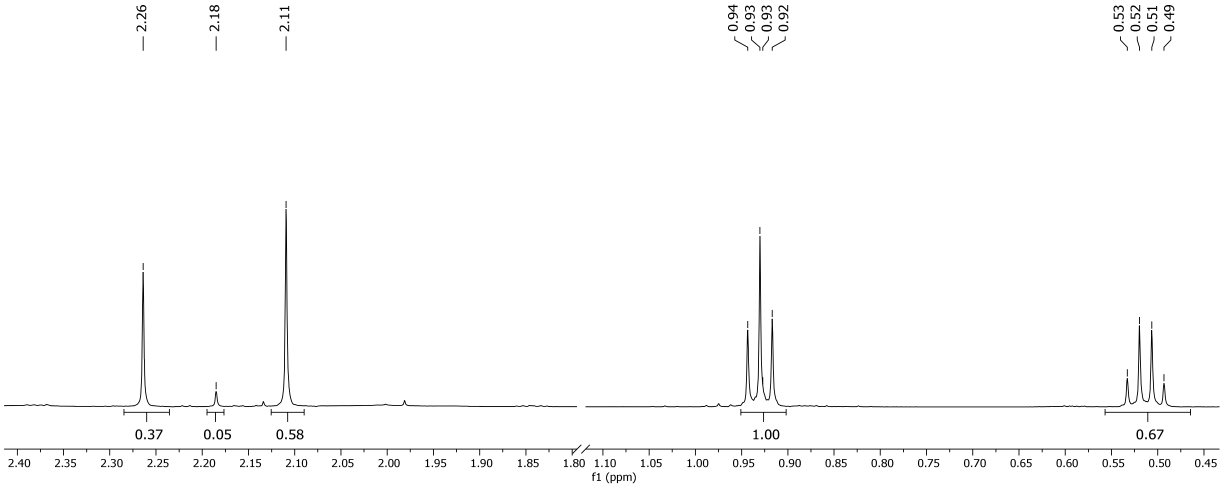

*Figure S10: 600 MHz ^{1}H-NMR spectrum for the Diels-Alder-reaction between CPD and MVK after 30 min employing 2-methyl-1-phenyl-1H-benzo[4,5]iodolo[3,2-c]pyrazole-2,4-diium bis(tetrakis(3,5-bis(trifluoromethyl)phenyl)borate (**7f**) as the XB-donor. The spectrum is zoomed in to show the relevant signals for the reaction progress.*

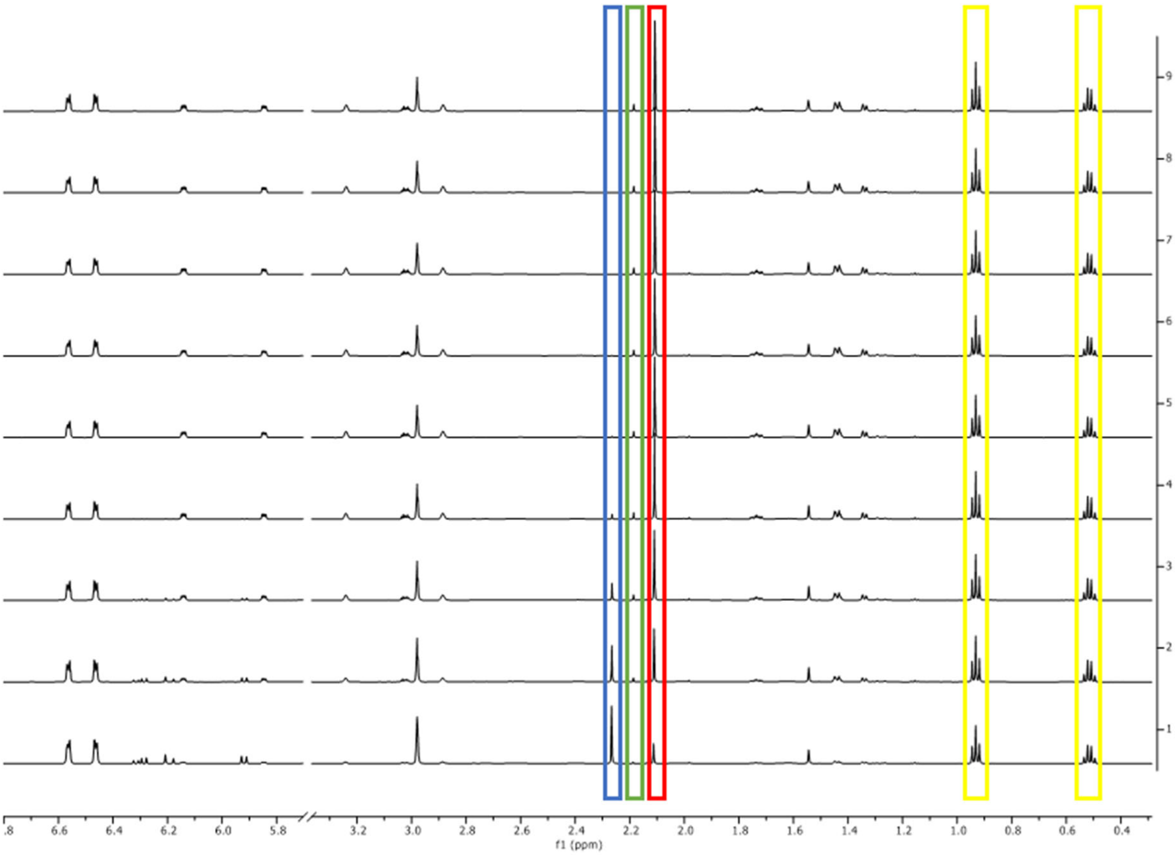

Figure S11: 600 MHz ^{1}H-NMR spectra for the Diels-Alder-reaction between CPD and MVK over the course of 170 min employing 2-methyl-1-phenyl-1H-benzo[4,5]iodolo[3,2-c]pyrazole-2,4-diium bis(tetrakis(3,5-bis(trifluoromethyl)-phenyl)borate) ***7f*** *as the XB-donor. Important signals for integration highlighted. In blue: Methyl group of MVK; in green: Methyl group of the exo product; in red: Methyl group of the endo product; in yellow: TES as the internal standard.*

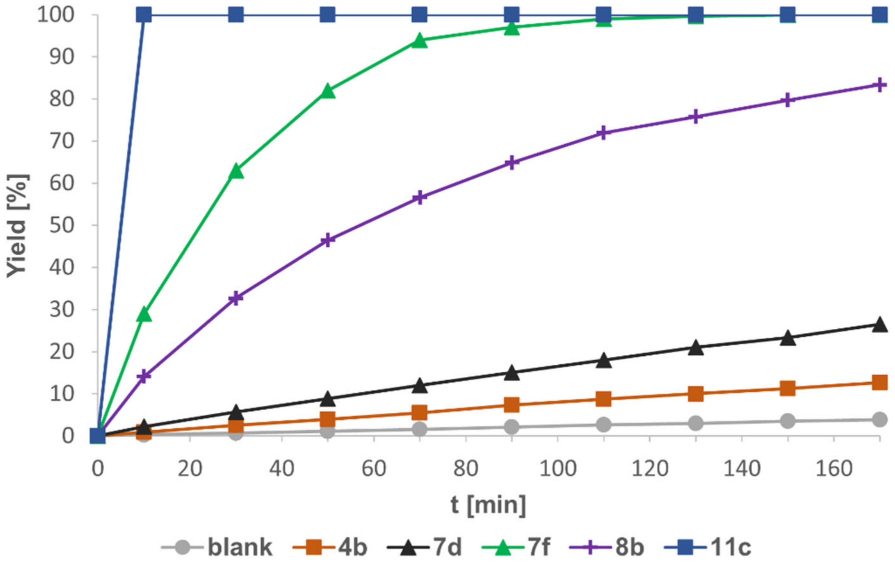

Figure S12 Yield-vs.-time profile *for the Diels-Alder-Reaction between CPD and MVK over the course of 170 min.*

Between CHD and MVK

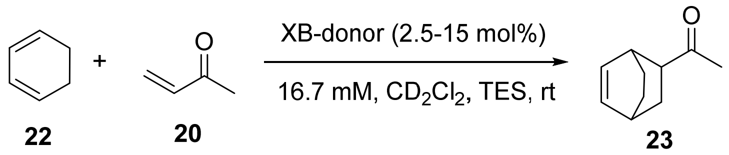

A modified literature procedure was used.[17] The corresponding XB-donor (0.25-1.5 µmol, 2.5-15 mol%) was directly weighed into a fresh NMR-tube (except for BF_3•Et_2O for which a 50 mM stock solution was prepared) and CD_2Cl_2 (400 µL) was added, followed by a 100 mM stock solution of cyclohexadiene (100 µL, 10 µmol, 1.0 equiv.) and a 100 mM stock solution of freshly distilled methyl vinyl ketone (100 µL, 10 µmol, 1.0 equiv., incl. 0.25 equiv. TES). The tube was sealed, shaken and afterwards time-dependent ^{1}H-NMR measurements were conducted (8 scans, d_1 = 15 s, suitable for integration, an error margin of 5% is assumed). Important measurements were conducted at least twice.

The yields were determined as follows: The integral of the ethyl peak of TES (0.51 ppm, q, *J* = 7.9 Hz) was calibrated to 2 and/or the integral of the methyl peak of TES (0.93 ppm, t, *J* = 7.9 Hz) was calibrated to 3. Afterwards the yields were determined by integration of three characteristic and equal signals at 3.02 – 2.85 ppm (m, 1H), 2.74 ppm (ddd, *J* = 9.7, 5.7, 2.1 Hz, 1H), 2.64 – 2.54 ppm (m, 1H).

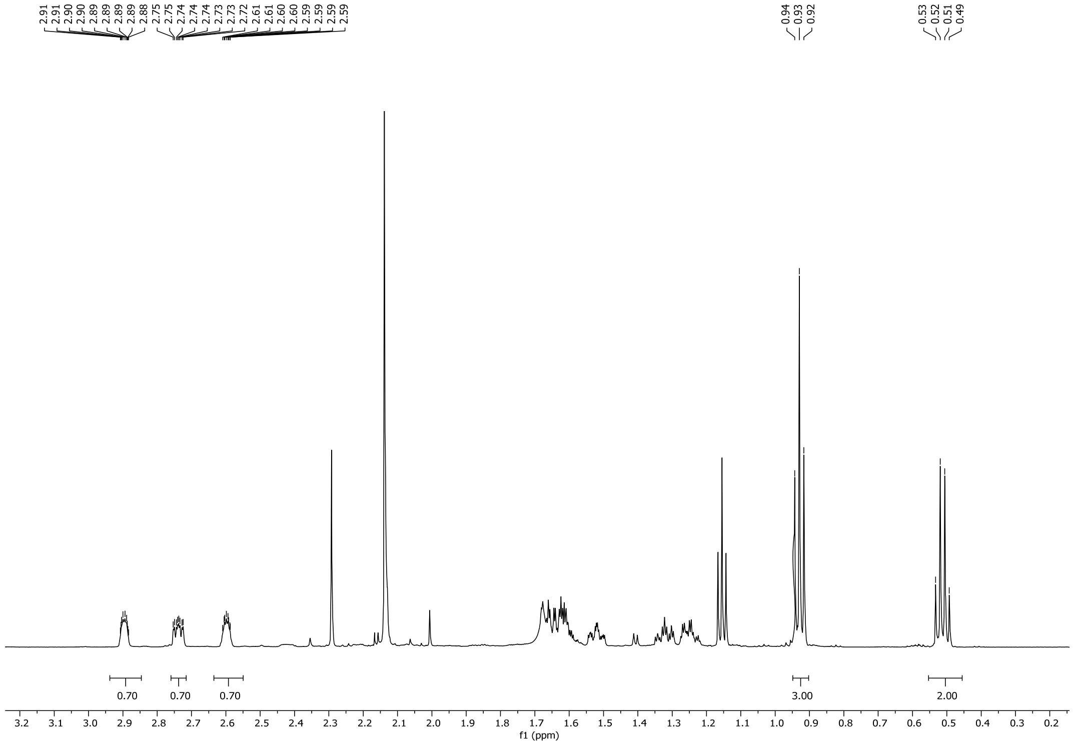

*Figure S13: 600 MHz ^{1}H-NMR spectrum for the Diels-Alder-reaction between CHD and MVK after 2.5 h employing 5 mol% of 1-methylbenzo[d]pyrazolo[5,1-b][1,3]iodazole-1,4-diium tetrakis(pentafluorophenyl)borate dietherate complex (**8d**) as the XB-donor. The spectrum is zoomed in to show the relevant signals for the reaction progress.*

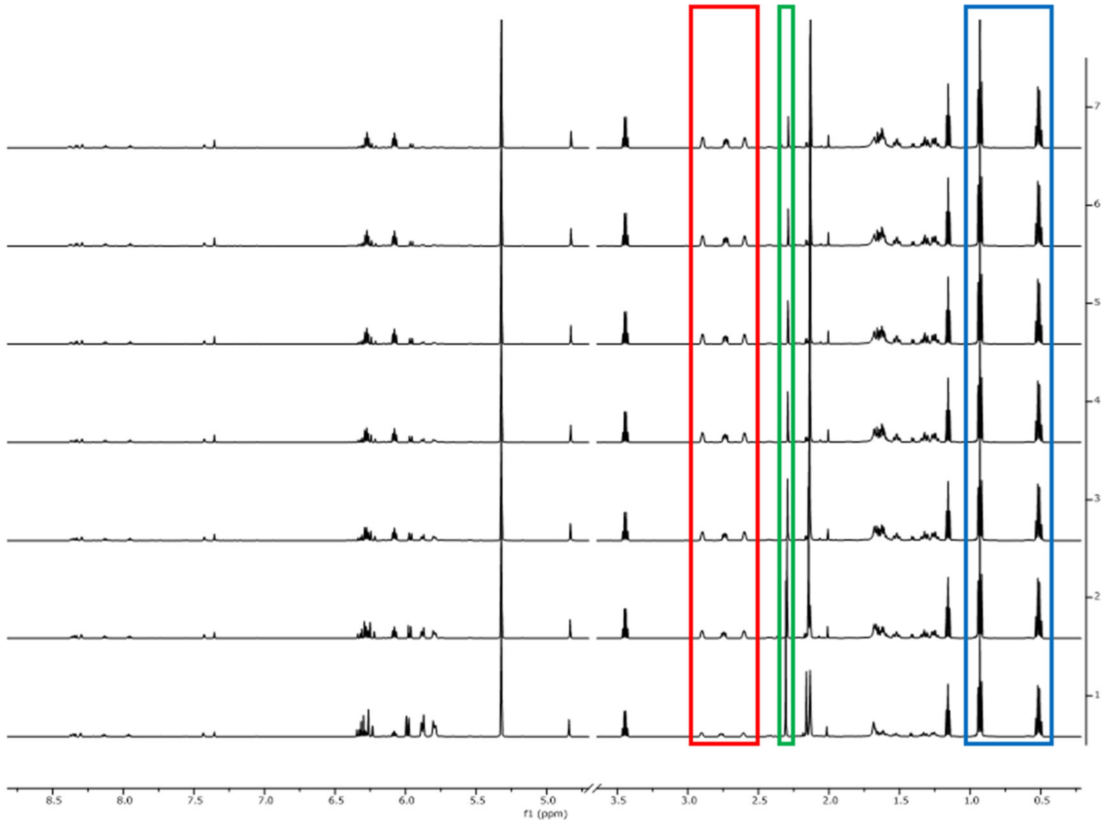

*Figure S14: 600 MHz ^{1}H-NMR spectra for the Diels-Alder-reaction between CHD and MVK employing 5 mol% of 1-methylbenzo[d]pyrazolo[5,1-b][1,3]iodazole-1,4-diium tetrakis(pentafluorophenyl)borate dietherate complex (**8d**) as the XB-donor. Important signals for integration highlighted. In blue: TES as the internal standard; in green: Consumption of the methyl group of the MVK; in red: important signals of the product.*

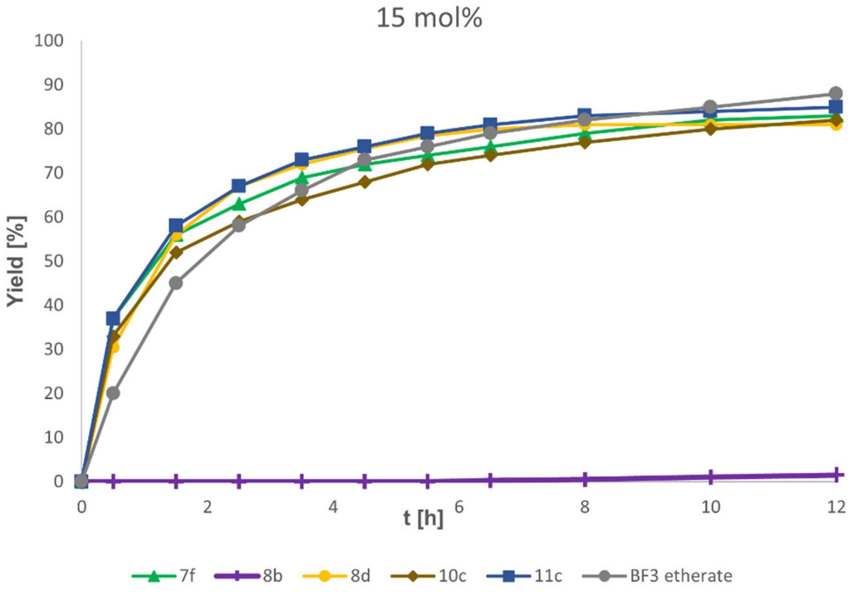

*Figure S15: Yield-vs.-time profile for the XB-mediated Diels-Alder reaction between CHD (**22**) and MVK (**20**) over the course of 12 h employing 15 mol% of several cyclic iodonium salts as potential activators in CD_2Cl_2.*

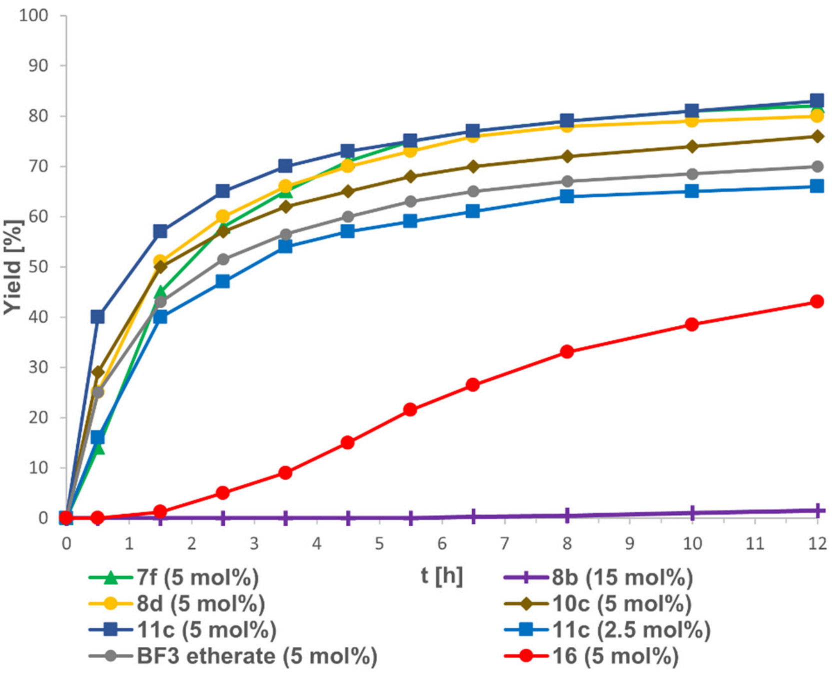

*Figure S16: Yield-vs.-time profile for the XB-mediated Diels-Alder reaction between CHD (**22**) and MVK (**20**) over the course of 12 h employing several cyclic iodonium salts as potential activators in CD_2Cl_2.*

Stability test

Preparation as described above. After 10 h, a second portion of both stock solutions of starting materials **22** and **20** were added and the reaction was monitored for further 10 h.

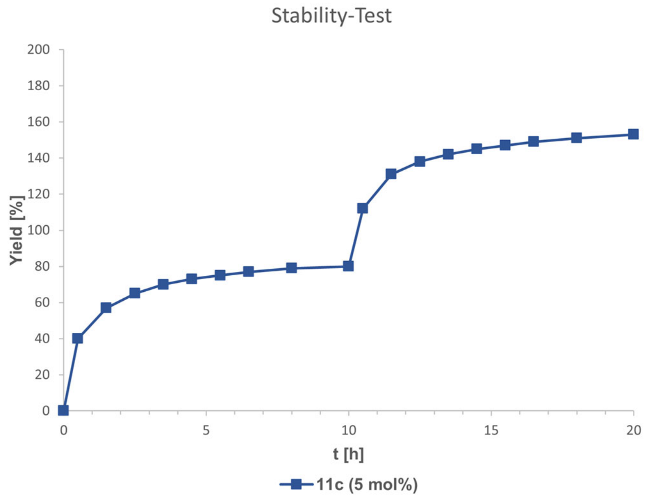

*Figure S17: Yield-vs.-time profile for the stability test for the XB-mediated Diels-Alder reaction between CHD (**22**) and MVK (**20**) over the course of 2 x 10 h employing 5 mol% of 11-methylbenzo[4',5']iodolo[3',2':4,5]imidazo[1,2-a]pyridine-5,11-diium bis(tetrakis(3,5-bis(trifluoromethyl)phenyl)borate) etherate complex (**11c**) as the catalyst.*

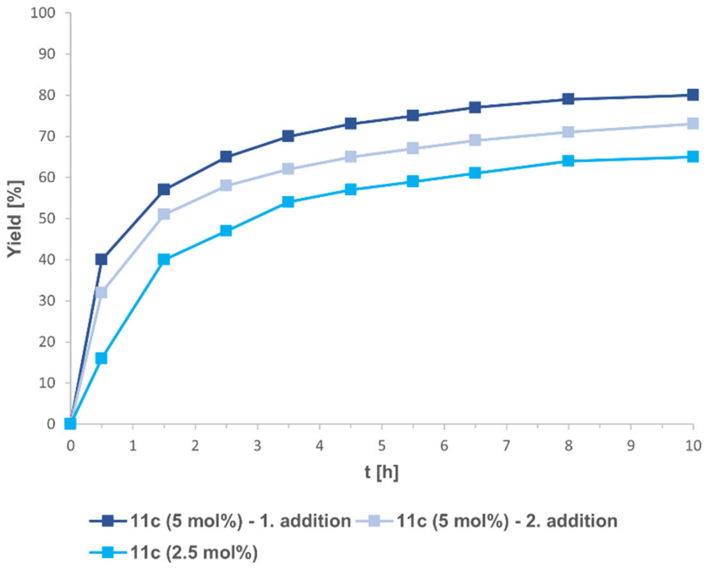

*Figure S18: Comparison in yield-vs.-time profile for the XB-mediated Diels-Alder reaction between CHD (**22**) and MVK (**20**) over the course of 10 h employing 11-methylbenzo[4',5']iodolo[3',2':4,5]imidazo[1,2-a]pyridine-5,11-diium bis(tetrakis(3,5-bis(trifluoromethyl)phenyl)borate) etherate complex (**11c**) as the catalyst in 5 mol% for the stability test (1. and 2. addition) and in 2.5 mol%.*

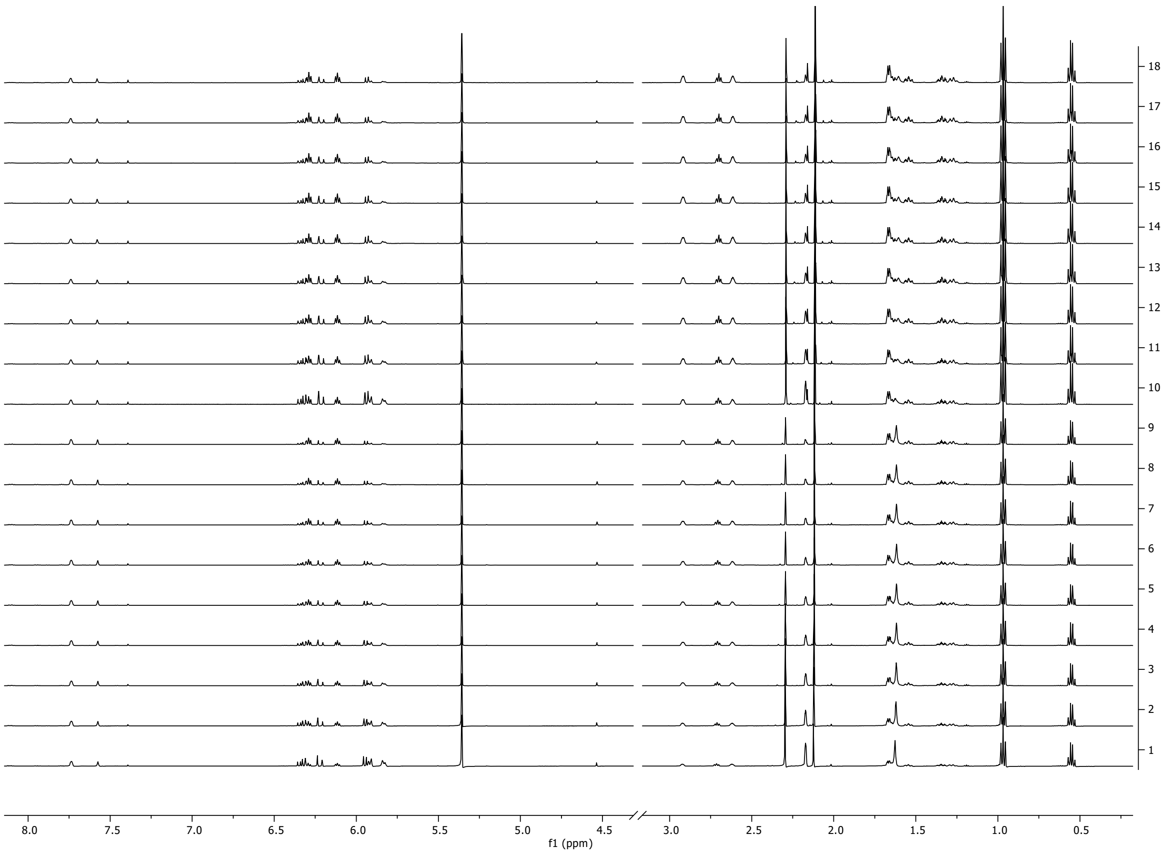

Figure 19: Periodic 600 MHz ^{1}H-NMR spectra for the stability test.

Michael reaction

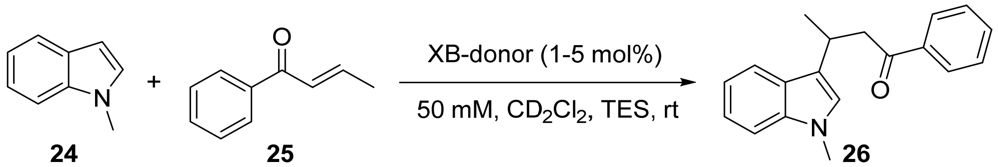

24 **25** **26**

A slightly modified literature procedure was used.[17] The corresponding XB-donor (0.3-1.5 µmol, 1-5 mol%) was directly weighed into a fresh NMR-tube or added as a 15 mM stock solution (20 µL) for 1 mol% and CD_2Cl_2 (180-200 µL) was added, followed by a 150 mM stock solution of *trans*-β-crotonophenone (200 µL, 30 µmol, 1.0 equiv., incl. 0.25 equiv. TES) and a 150 mM stock solution of 1-methylindole (200 µL, 30 µmol, 1.0 equiv.). The tube was sealed, shaken and afterwards time-dependent ^{1}H-NMR measurements were conducted (8 scans, d_1 = 15 s, suitable for integration, an error margin of 5% is assumed). Important measurements were conducted at least twice.

The yields were determined as follows: The integral of the ethyl peak of TES (0.52 ppm, q, J = 7.9 Hz) was calibrated to 2 and/or the integral of the methyl peak of TES (0.94 ppm, t, J = 7.9 Hz) was calibrated to 3. Afterwards the yields were determined by integration of the characteristic signal of product **26** at 3.24 ppm (dd, J = 16.4, 8.4 Hz, 1H) (and/or at 3.45 ppm (dd, J = 16.4, 5.4 Hz, 1H)). Furthermore, the consumption of the CH_3 signal of the starting material **25** at 2.00 ppm (dd, J = 6.8, 1.5 Hz, 3H) can be monitored against the formation that of the product **26** at 1.42 ppm (d, J = 6.9 Hz, 3H).

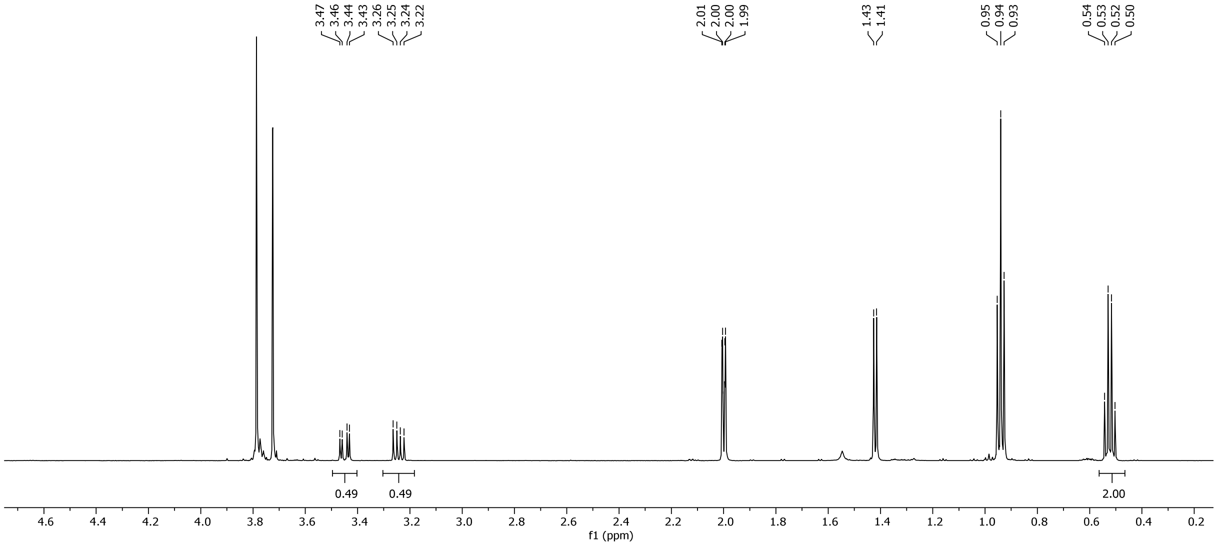

*Figure S20: 600 MHz ^{1}H-NMR spectrum for the Michael addition between 1-methylindole (**24**) and crotonophenone **25** after 2.5 h employing 1 mol% of 11-methylbenzo[4',5']iodolo[3',2':4,5]imidazo[1,2-a]pyridine-5,11-diium bis(tetrakis(3,5-bis(trifluoromethyl)phenyl)borate) etherate complex (**11c**) as the XB-donor. The spectrum is zoomed in to show the relevant signals for the reaction progress.*

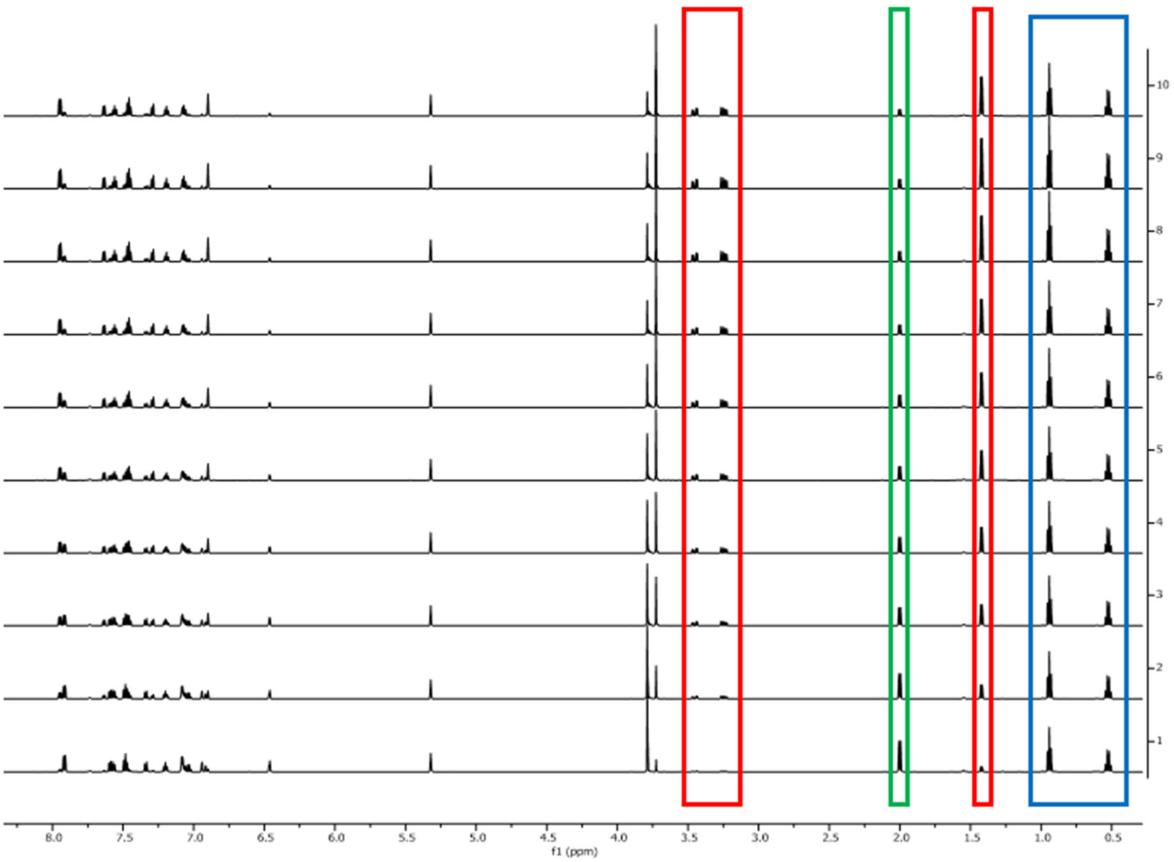

*Figure S21: 600 MHz ^{1}H-NMR spectra for the Michael addition between 1-methylindole (**24**) and crotonophenone **25** employing 1 mol% of 11-methylbenzo[4',5']iodolo[3',2':4,5]imidazo[1,2-a]pyridine-5,11-diium bis(tetrakis(3,5-bis(trifluoromethyl)phenyl)borate) etherate complex (**11c**) as the XB-donor. Important signals for integration highlighted. In blue: TES as the internal standard; in green: Consumption of the methyl group of crotonophenone; in red: important signals of the product.*

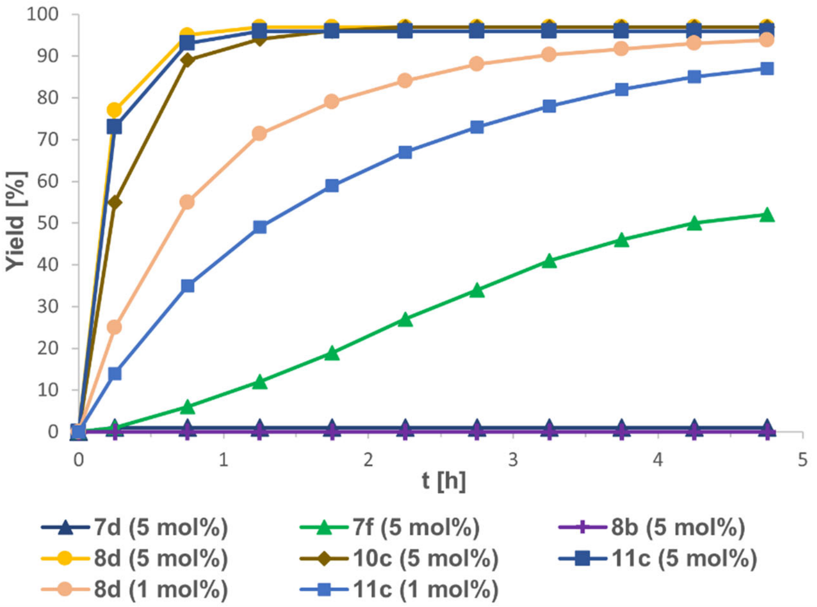

*Figure S22: Yield-vs.-time profile for the XB-mediated Micheal reaction between 1-methylindole (**24**) and trans-β-crotonophenone (**25**) over the course of 4.75 h employing several cyclic iodonium salts as potential activators in CD_2Cl_2*

Nitro-Michael reaction

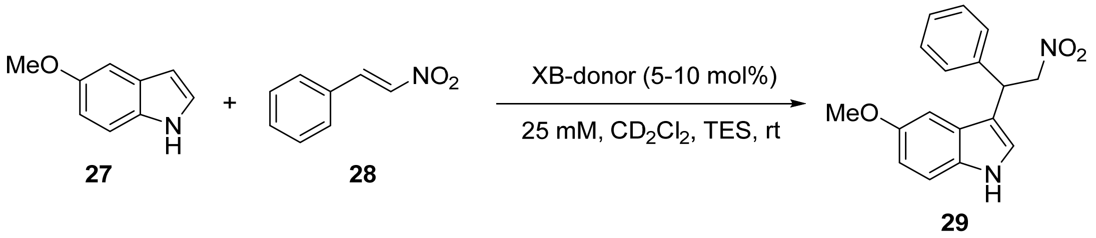

A reported literature procedure was used.[17] The corresponding XB-donor (0.75-1.5 µmol, 5-10 mol%) was directly weighed into a fresh NMR-tube and CD_2Cl_2 (200 µL) was added, followed by a 75 mM stock solution of nitrostyrene **28** (200 µL, 15 µmol, 1.0 equiv., incl. 0.25 equiv. TES) and a 75 mM stock solution of 5-methoxyindole (200 µL, 15 µmol, 1.0 equiv.). The tube was sealed, shaken and afterwards time-dependent ^{1}H-NMR measurements were conducted (8 scans, d_1 = 15 s, suitable for integration, an error margin of 5% is assumed). Important measurements were conducted at least twice.

The yields were determined as follows: The integral of the methyl peak of TES (0.94 ppm, t, *J* = 7.9 Hz) was calibrated to 1. Afterwards the yields were determined by integration of the characteristic methoxy signal of product **29** at 3.75 ppm (s) at 4.98 (dd, *J* = 12.1, 8.0 Hz, 1H)). Furthermore, the consumption of the OCH_3 signal of the starting material **27** at 3.82 ppm (s) can be used to double check the yield. Additionally, the characteristic signal of product **29** at 4.98 ppm (dd, *J* = 12.1, 8.0 Hz, 1H) can be used, when calibrating TES triplet to 3.

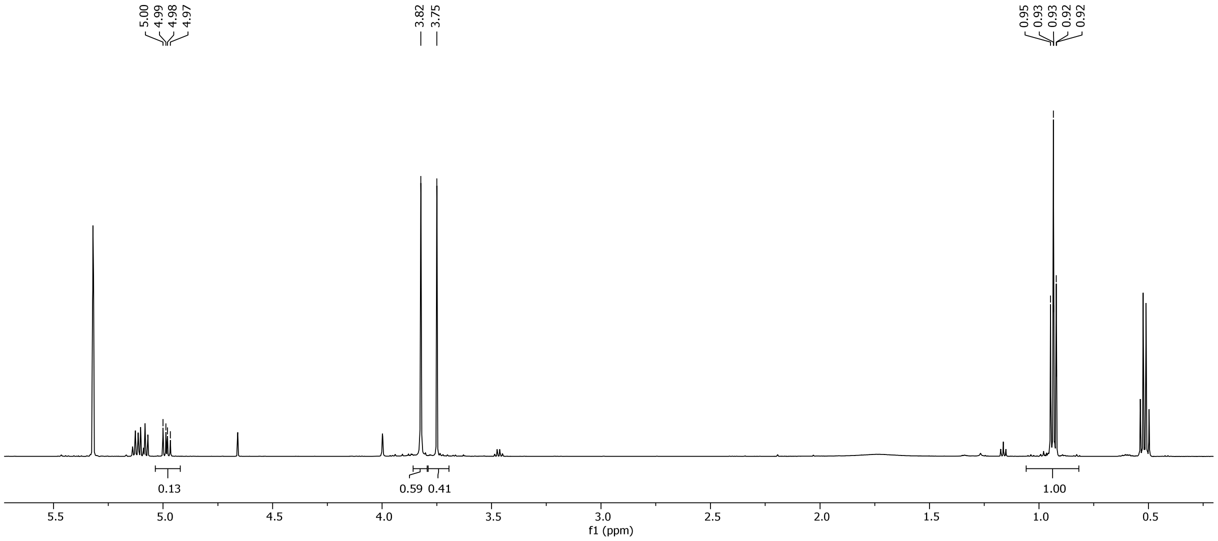

*Figure S23: 600 MHz ^{1}H-NMR spectrum for the nitro-Michael addition between 5-methoxyindole (**27**) and nitrostyrene **28** after 0.5 h employing 10 mol% of 2-methyl-1-phenyl-1H-benzo[4,5]iodolo[3,2-c]pyrazole-2,4-diium bis(tetrakis(3,5-bis(trifluoromethyl)phenyl)borate etherate complex (**7f**) as the XB-donor. The spectrum is zoomed in to show the relevant signals for the reaction progress.*

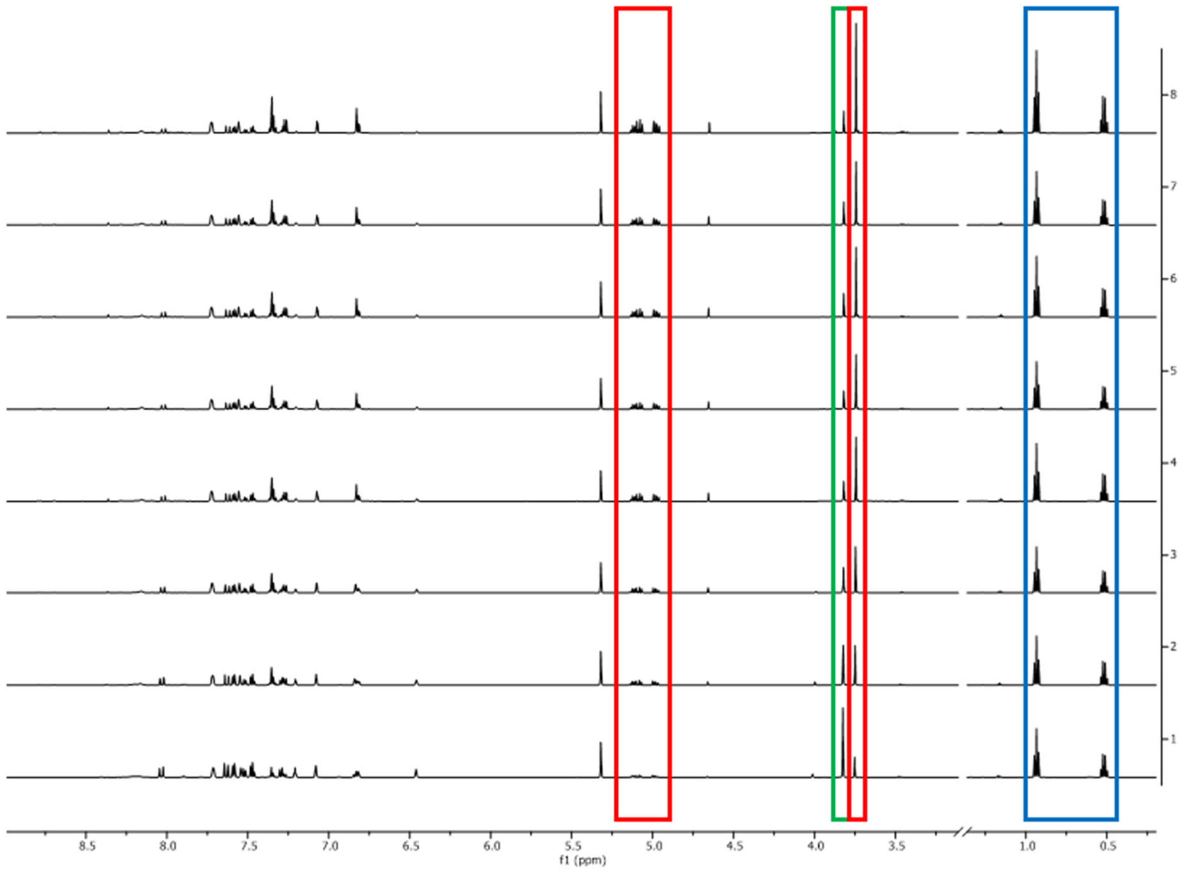

*Figure S24: 600 MHz ^{1}H-NMR spectra for the nitro-Michael addition between 5-methoxyindole (**27**) and nitrostyrene **28** employing 10 mol% of 2-methyl-1-phenyl-1H-benzo[4,5]iodolo[3,2-c]pyrazole-2,4-diium bis(tetrakis(3,5-bis(trifluoromethyl)phenyl)borate etherate complex (**7f**) as the XB-donor. Important signals for integration highlighted. In blue: TES as the internal standard; in green: Consumption of the methoxy group of 5-methoxyindole (**27**); in red: important signals of the product.*

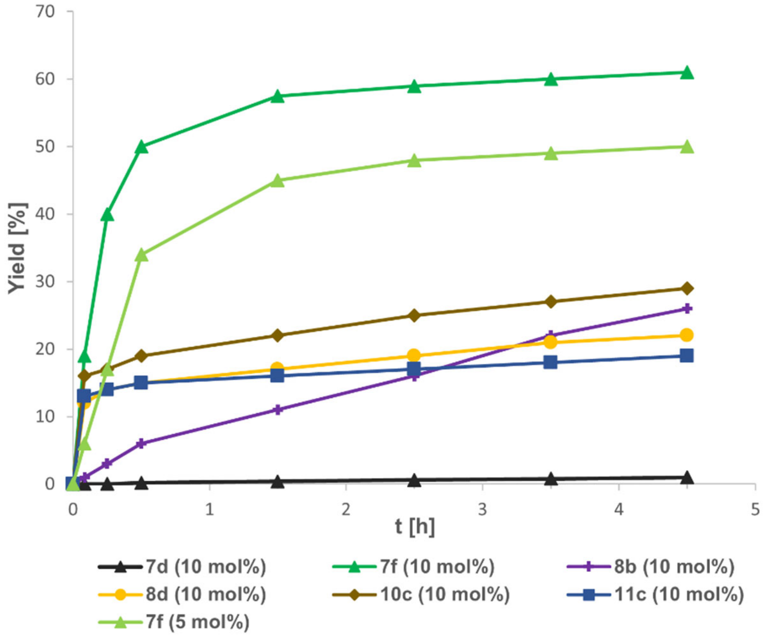

*Figure S25: Yield-vs.-time profile for the XB-mediated nitro-Michael reaction between 5-methoxyindole (**27**) and nitrostyrene **28** over the course of 4.5 h employing several cyclic iodonium salts as potential activators in CD_2Cl_2.*

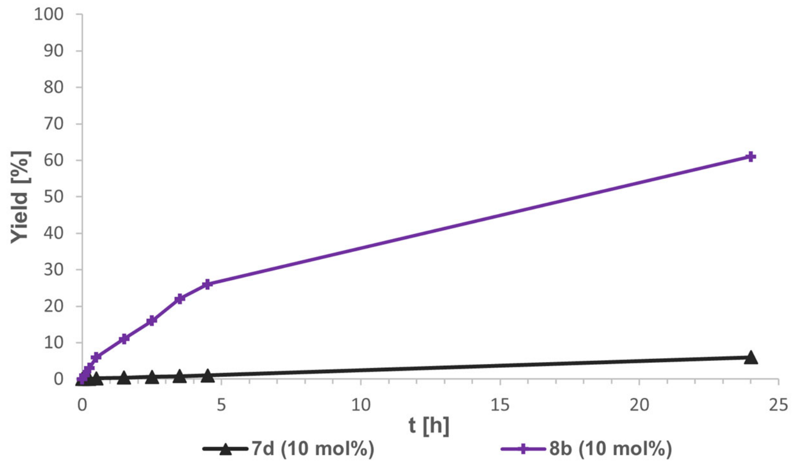

*Figure S26: Yield-vs.-time profile for the XB-mediated nitro-Michael reaction between 5-methoxyindole (**27**) and nitrostyrene **28** over the course of 24 h employing **7d** and **8b** as potential activators in CD_2Cl_2.*

Investigation upon the reaction between 5-methoxyindole (27) and 11c

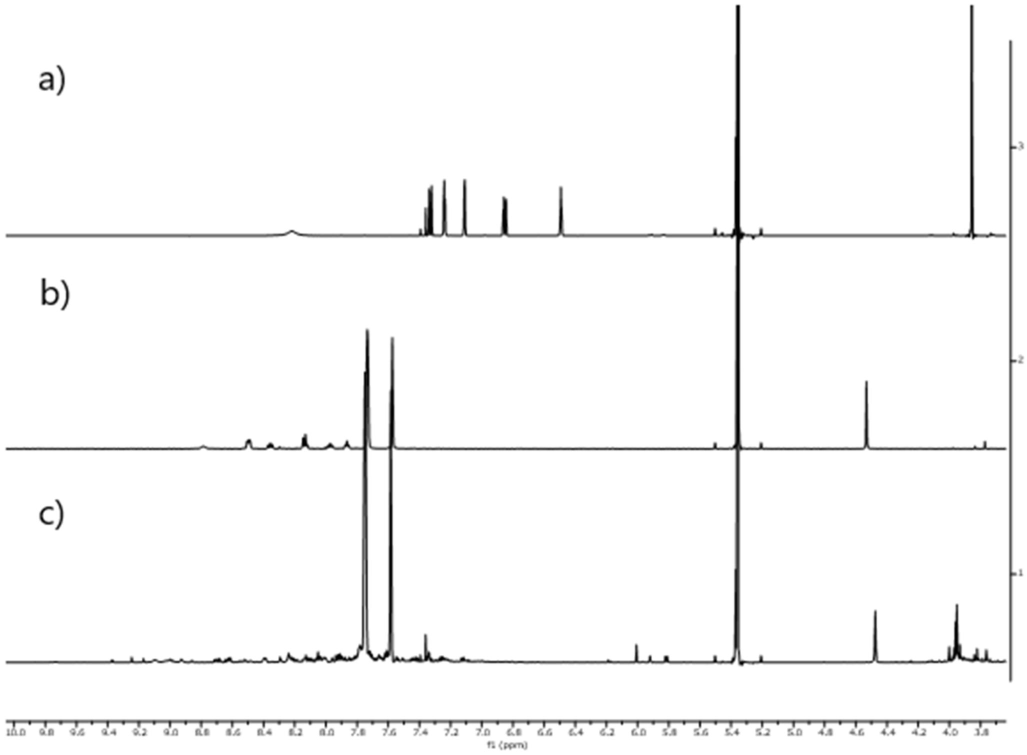

*Figure S27: Comparison of 600 MHz ^{1}H-NMR spectra in CD_2Cl_2 of 5-methoxyindole (**27**) (a), **11c** (b) and a mixture of both (c) with clearly visible degradation of both species. Especially 5-methoxyindole (**27**) shows a complex decomposition mixture in the aromatic region and the area around the methoxy signal (3.80 –4.00 ppm, a vs. c).*

7. References

[1] W. L. F. Armarego, C. L. L. Chai, *Purification of Laboratory Chemicals*, Elsevier, Amsterdam, **2009**.

[2] O. V. Dolomanov, L. J. Bourhis, R. J. Gildea, J. A. K. Howard, H. Puschmann, *J. Appl. Crystallogr.* **2009**, *42*, 339.

[3] G. M. Sheldrick, *Acta Cryst.* **2015**, *A71*, 3.

[4] G. M. Sheldrick, *Acta Cryst.* **2008**, *A64*, 112.

[5] S. Sangeetha, P. Muthupandi, G. Sekar, *Org. Lett.* **2015**, *17*, 6006.

[6] A. Boelke, T. J. Kuczmera, L. D. Caspers, E. Lork, B. J. Nachtsheim, *Organic letters* **2020**, *22*, 7261.

[7] J.-M. L'Helgoual'ch, A. Seggio, F. Chevallier, M. Yonehara, E. Jeanneau, M. Uchiyama, F. Mongin, *J. Org. Chem.* **2008**, *73*, 177.

[8] L. Zhu, P. Guo, G. Li, J. Lan, R. Xie, J. You, *J. Org. Chem.* **2007**, *72*, 8535.

[9] C.-P. Zhang, Z.-L. Wang, Q.-Y. Chen, C.-T. Zhang, Y.-C. Gu, J.-C. Xiao, *Angew. Chem. Int. Ed.* **2011**, *50*, 1896.

[10] A. Boelke, E. Lork, B. J. Nachtsheim, *Chem. Eur. J.* **2018**, *24*, 18653.

[11] G. Pandey, J. Vaitla, *Organic letters* **2015**, *17*, 4890.

[12] J. C. Rodríguez, R. A. Maldonado, G. Ramírez-García, E. Díaz Cervantes, F. N. Cruz, *J. Heterocyclic Chem.* **2020**, *57*, 2279.

[13] P. Wonner, L. Vogel, M. Düser, L. Gomes, F. Kniep, B. Mallick, D. B. Werz, S. M. Huber, *Angew. Chem. Int. Ed.* **2017**, *56*, 12009.

[14] J. G. Park, I.-R. Jeon, T. D. Harris, *Inorg. Chem.* **2015**, *54*, 359.

[15] L. Omann, M. Oestreich, *Organometallics* **2017**, *36*, 767.

[16] F. Heinen, E. Engelage, A. Dreger, R. Weiss, S. M. Huber, *Angew. Chem. Int. Ed.* **2018**, *57*, 3830.

[17] F. Heinen, D. L. Reinhard, E. Engelage, S. M. Huber, *Angew. Chem. Int. Ed.* **2020**.

[18] D. Landini, F. Rolla, *J. Org. Chem.* **1980**, *45*, 3527.

[19] C. Zhao, F. D. Toste, K. N. Raymond, R. G. Bergman, *J. Am. Chem. Soc.* **2014**, *136*, 14409.

[20] P. Wipf, Y. Aoyama, T. E. Benedum, *Organic letters* **2004**, *6*, 3593.

[21] D. von der Heiden, S. Bozkus, M. Klussmann, M. Breugst, *J. Org. Chem.* **2017**, *82*, 4037.

[22] W. J. Kerr, R. J. Mudd, J. A. Brown, *Chem. Eur. J.* **2016**, *22*, 4738.

[23] J. Wolf, F. Huber, N. Erochok, F. Heinen, V. Guérin, C. Y. Legault, S. F. Kirsch, S. M. Huber, *Angew. Chem. Int. Ed.* **2020**, *59*, 16496.

8. Crystal Structure

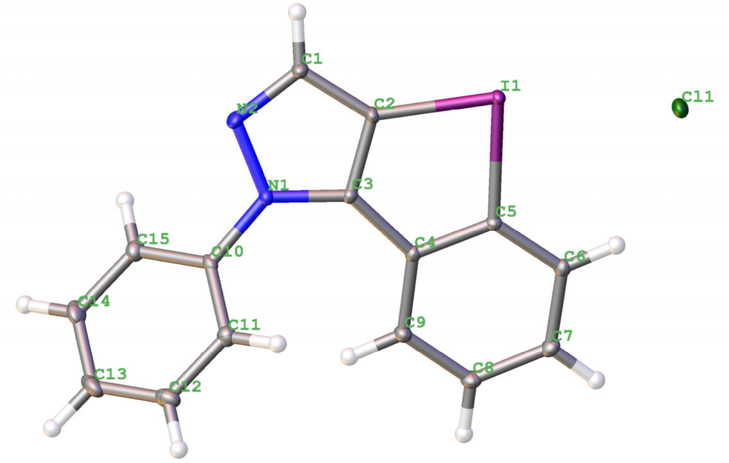

Table 1. Crystal data and structure refinement for 7c.

CCDC number	2082275
Empirical formula	$C_{15}H_{10}ClIN_2$
Formula weight	380.60
Temperature [K]	100.0
Crystal system	orthorhombic
Space group (number)	$P2_12_12_1$ (19)
a [Å]	7.3707(2)
b [Å]	9.4469(3)
c [Å]	18.7203(5)
α [Å]	90
β [Å]	90
γ [Å]	90
Volume [Å^3]	1303.50(6)
Z	4
ρ_{calc} [g/cm^3]	1.939
μ [mm^{-1}]	2.647
F(000)	736

Crystal size [mm^3]	0.21×0.18×0.17
Crystal colour	colourless
Crystal shape	block
Radiation	MoK_α (λ=0.71073 Å)
2Θ range [°]	4.35 to 66.47 (0.65 Å)
Index ranges	-11 ≤ h ≤ 11<br>-14 ≤ k ≤ 14<br>-28 ≤ l ≤ 28
Reflections collected	25818
Independent reflections	4988<br>R_{int} = 0.0224<br>R_{sigma} = 0.0199
Completeness to θ = 25.242°	99.9 %
Data / Restraints / Parameters	4988/0/172
Goodness-of-fit on F^2	1.105
Final R indexes [I≥2σ(I)]	R_1 = 0.0133<br>wR_2 = 0.0317
Final R indexes [all data]	R_1 = 0.0141<br>wR_2 = 0.0318
Largest peak/hole [eÅ^3]	0.44/-0.71
Flack X parameter	-0.014(4)

Table 2. Atomic coordinates and U_{eq} [Å^2] for 7c.

Atom	*x*	*y*	*z*	U_{eq}
I1	0.47333(2)	0.31574(2)	0.33998(2)	0.00789(3)
N1	0.4205(2)	0.58947(15)	0.50901(8)	0.0096(3)
N2	0.3844(2)	0.69538(16)	0.46082(8)	0.0117(3)
C1	0.3910(3)	0.63542(19)	0.39642(9)	0.0111(3)
H1	0.369954	0.683097	0.352510	0.013
C2	0.4334(3)	0.49188(17)	0.40320(9)	0.0096(3)
C3	0.4482(2)	0.46339(17)	0.47541(9)	0.0085(3)
C4	0.4677(2)	0.31752(17)	0.49909(8)	0.0086(3)
C5	0.4865(3)	0.21959(17)	0.44305(8)	0.0085(3)
C6	0.5032(2)	0.07489(17)	0.45375(9)	0.0100(3)
H6	0.518369	0.011737	0.414716	0.012
C7	0.4969(3)	0.02560(17)	0.52380(9)	0.0119(3)
H7	0.508340	-0.072888	0.533018	0.014
C8	0.4741(3)	0.11917(18)	0.58031(9)	0.0123(3)
H8	0.468511	0.083537	0.627713	0.015
C9	0.4592(3)	0.26405(18)	0.56873(9)	0.0110(3)
H9	0.443297	0.326594	0.607970	0.013
C10	0.4389(3)	0.62895(18)	0.58219(9)	0.0095(3)
C11	0.5814(3)	0.57771(19)	0.62277(10)	0.0121(3)
H11	0.664535	0.511395	0.603164	0.015
C12	0.6010(3)	0.6251(2)	0.69301(10)	0.0162(4)
H12	0.696232	0.589028	0.721980	0.019
C13	0.4820(3)	0.7244(2)	0.72048(10)	0.0185(4)
H13	0.497665	0.757897	0.767928	0.022
C14	0.3400(3)	0.7754(2)	0.67919(10)	0.0166(4)
H14	0.258393	0.843259	0.698422	0.020
C15	0.3174(3)	0.7270(2)	0.60968(10)	0.0127(3)
H15	0.219698	0.760693	0.581261	0.015
Cl1	0.55570(7)	0.03570(5)	0.27618(2)	0.01586(9)

U_{eq} is defined as 1/3 of the trace of the orthogonalized U_{ij} tensor.

Table 3. Bond lengths and angles for 7c.

Atom–Atom	Length [Å]
I1–C2	2.0630(16)
I1–C5	2.1349(15)
N1–N2	1.373(2)
N1–C3	1.362(2)
N1–C10	1.426(2)
N2–C1	1.333(2)
C1–H1	0.9500
C1–C2	1.397(2)
C2–C3	1.383(2)
C3–C4	1.455(2)
C4–C5	1.406(2)
C4–C9	1.399(2)
C5–C6	1.387(2)
C6–H6	0.9500
C6–C7	1.392(2)
C7–H7	0.9500
C7–C8	1.389(2)
C8–H8	0.9500
C8–C9	1.390(2)
C9–H9	0.9500
C10–C11	1.384(3)
C10–C15	1.388(3)
C11–H11	0.9500
C11–C12	1.397(3)
C12–H12	0.9500
C12–C13	1.383(3)
C13–H13	0.9500
C13–C14	1.388(3)
C14–H14	0.9500
C14–C15	1.389(3)
C15–H15	0.9500

Atom–Atom–Atom	Angle [°]
C2–I1–C5	80.28(6)
N2–N1–C10	117.32(14)
C3–N1–N2	111.27(14)
C3–N1–C10	131.14(15)
C1–N2–N1	106.11(14)
N2–C1–H1	125.1
N2–C1–C2	109.77(16)
C2–C1–H1	125.1
C1–C2–I1	139.69(13)
C3–C2–I1	113.12(12)
C3–C2–C1	107.17(15)
N1–C3–C2	105.63(14)
N1–C3–C4	134.61(15)
C2–C3–C4	119.35(14)
C5–C4–C3	113.94(13)
C9–C4–C3	128.42(15)
C9–C4–C5	117.52(15)
C4–C5–I1	112.96(11)
C6–C5–I1	123.64(12)
C6–C5–C4	123.34(15)
C5–C6–H6	121.2
C5–C6–C7	117.56(15)
C7–C6–H6	121.2
C6–C7–H7	119.7
C8–C7–C6	120.57(15)
C8–C7–H7	119.7
C7–C8–H8	119.4
C7–C8–C9	121.16(16)
C9–C8–H8	119.4
C4–C9–H9	120.1
C8–C9–C4	119.81(16)
C8–C9–H9	120.1
C11–C10–N1	120.52(16)
C11–C10–C15	121.34(17)
C15–C10–N1	117.99(16)
C10–C11–H11	120.5
C10–C11–C12	118.91(18)
C12–C11–H11	120.5
C11–C12–H12	119.9
C13–C12–C11	120.11(19)
C13–C12–H12	119.9
C12–C13–H13	119.8
C12–C13–C14	120.45(18)
C14–C13–H13	119.8
C13–C14–H14	120.1
C13–C14–C15	119.87(18)
C15–C14–H14	120.1
C10–C15–C14	119.30(18)
C10–C15–H15	120.3
C14–C15–H15	120.4

Table 4. Torsion angles for 7c

Atom–Atom–Atom–Atom	Torsion Angle [°]
I1–C2–C3–N1	179.33(12)
I1–C2–C3–C4	-6.9(2)
I1–C5–C6–C7	-175.44(13)
N1–N2–C1–C2	-0.7(2)
N1–C3–C4–C5	177.2(2)
N1–C3–C4–C9	1.3(3)
N1–C10–C11–C12	176.32(16)
N1–C10–C15–C14	-175.27(17)
N2–N1–C3–C2	1.5(2)
N2–N1–C3–C4	-170.80(19)
N2–N1–C10–C11	-133.79(18)
N2–N1–C10–C15	42.0(2)
N2–C1–C2–I1	179.93(17)
N2–C1–C2–C3	1.7(2)
C1–C2–C3–N1	-1.9(2)
C1–C2–C3–C4	171.83(16)

C2–C3–C4–C5	5.7(2)
C2–C3–C4–C9	-170.18(19)
C3–N1–N2–C1	-0.5(2)
C3–N1–C10–C11	39.6(3)
C3–N1–C10–C15	-144.7(2)
C3–C4–C5–I1	-1.5(2)
C3–C4–C5–C6	-178.68(17)
C3–C4–C9–C8	177.4(2)
C4–C5–C6–C7	1.4(3)
C5–C4–C9–C8	1.7(3)
C5–C6–C7–C8	0.2(3)
C6–C7–C8–C9	-0.8(3)
C7–C8–C9–C4	-0.2(4)
C9–C4–C5–I1	174.81(14)
C9–C4–C5–C6	-2.3(3)
C10–N1–N2–C1	174.13(16)
C10–N1–C3–C2	-172.13(18)
C10–N1–C3–C4	15.5(3)
C10–C11–C12–C13	-1.7(3)
C11–C10–C15–C14	0.4(3)
C11–C12–C13–C14	1.5(3)
C12–C13–C14–C15	-0.3(3)
C13–C14–C15–C10	-0.7(3)
C15–C10–C11–C12	0.7(3)

9. NMR spectra

Precursors and iodonium salts

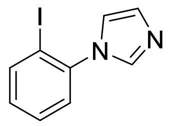

S12

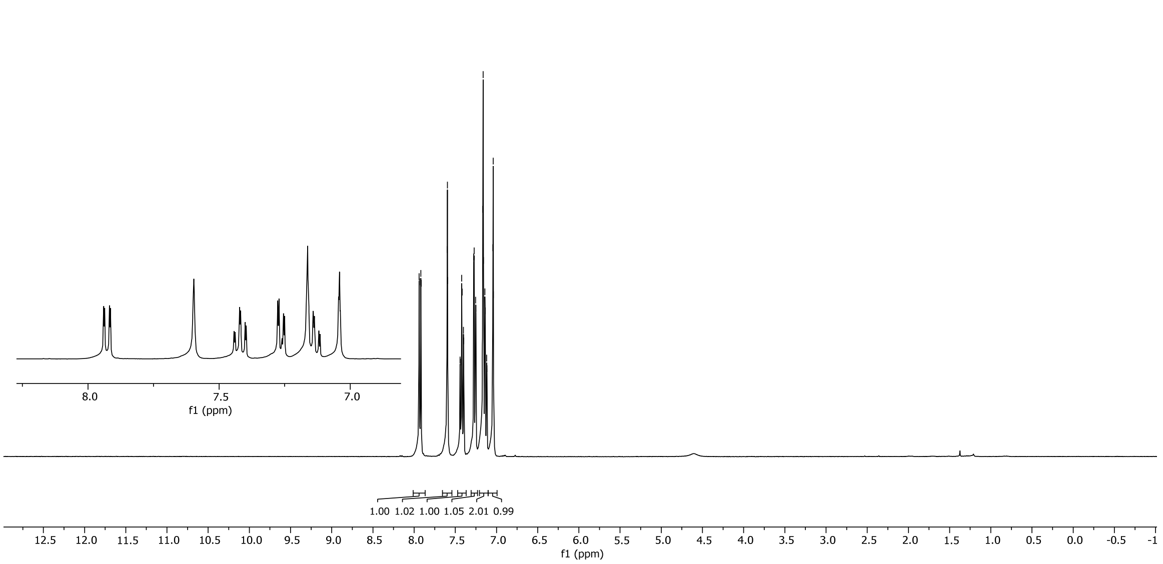

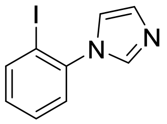

S12

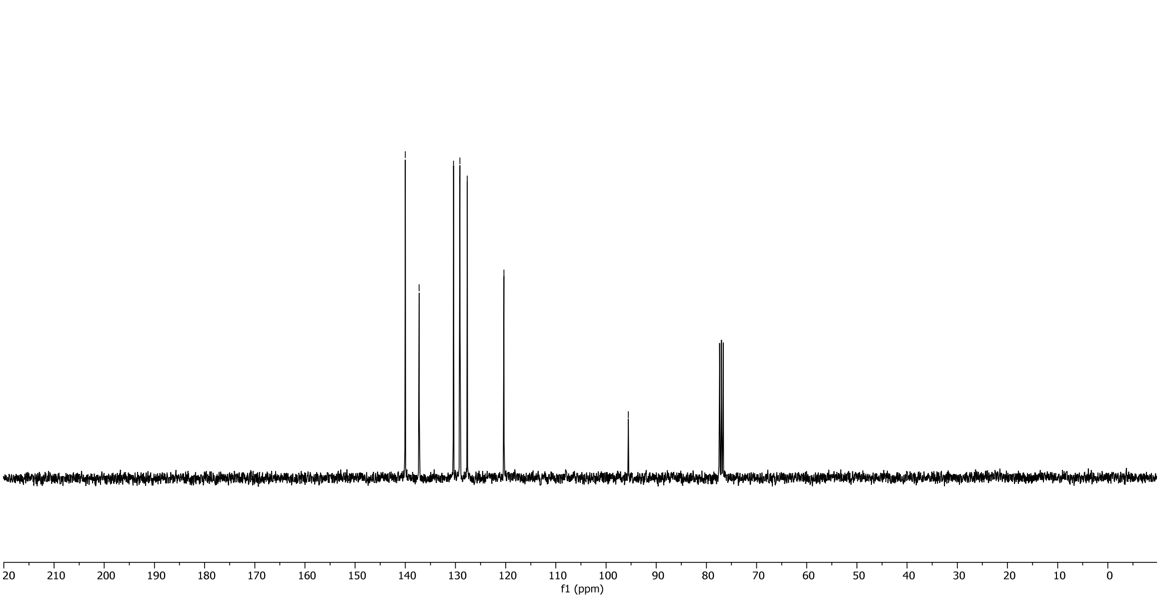

*Figure S28: 600 MHz ^{1}H- and 151 MHz ^{13}C-NMR spectra of 1-(2-iodophenyl)-1H-imidazole (**S12**) in $CDCl_3$.*

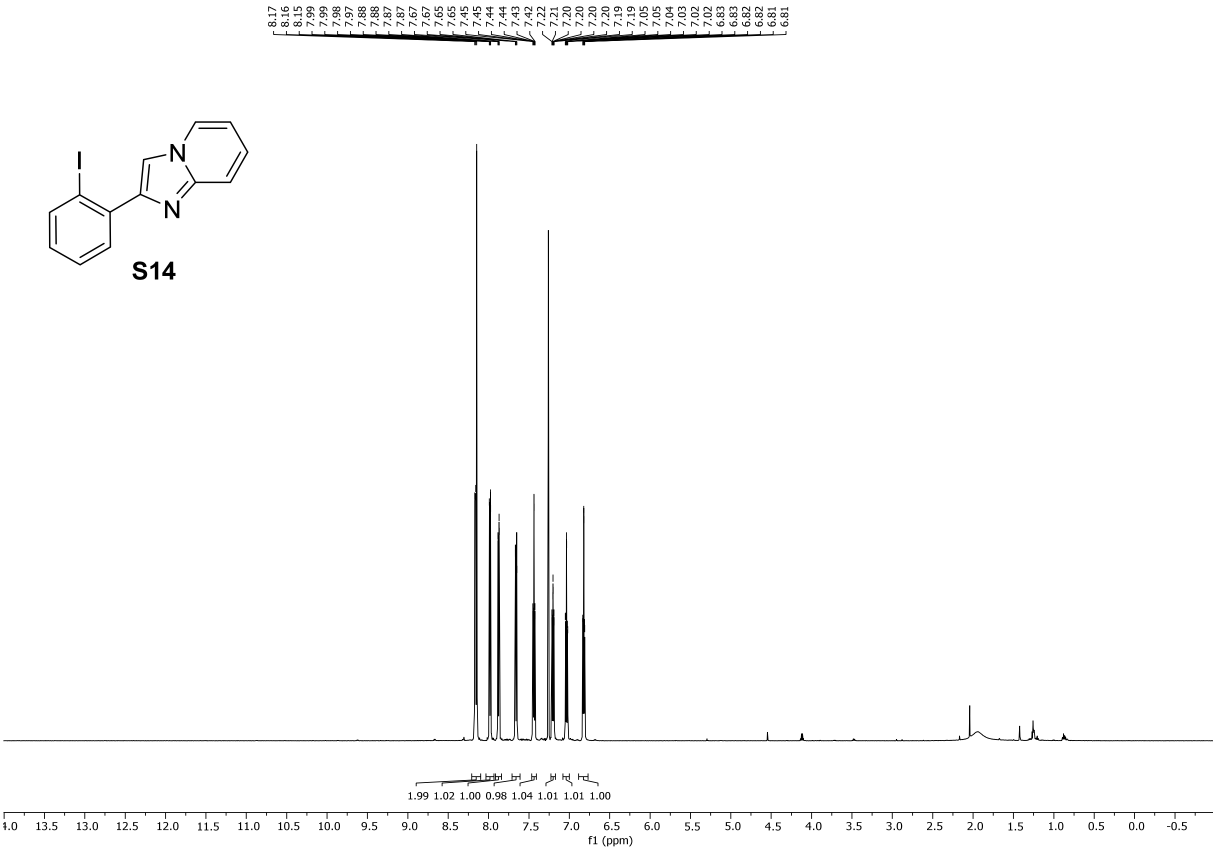

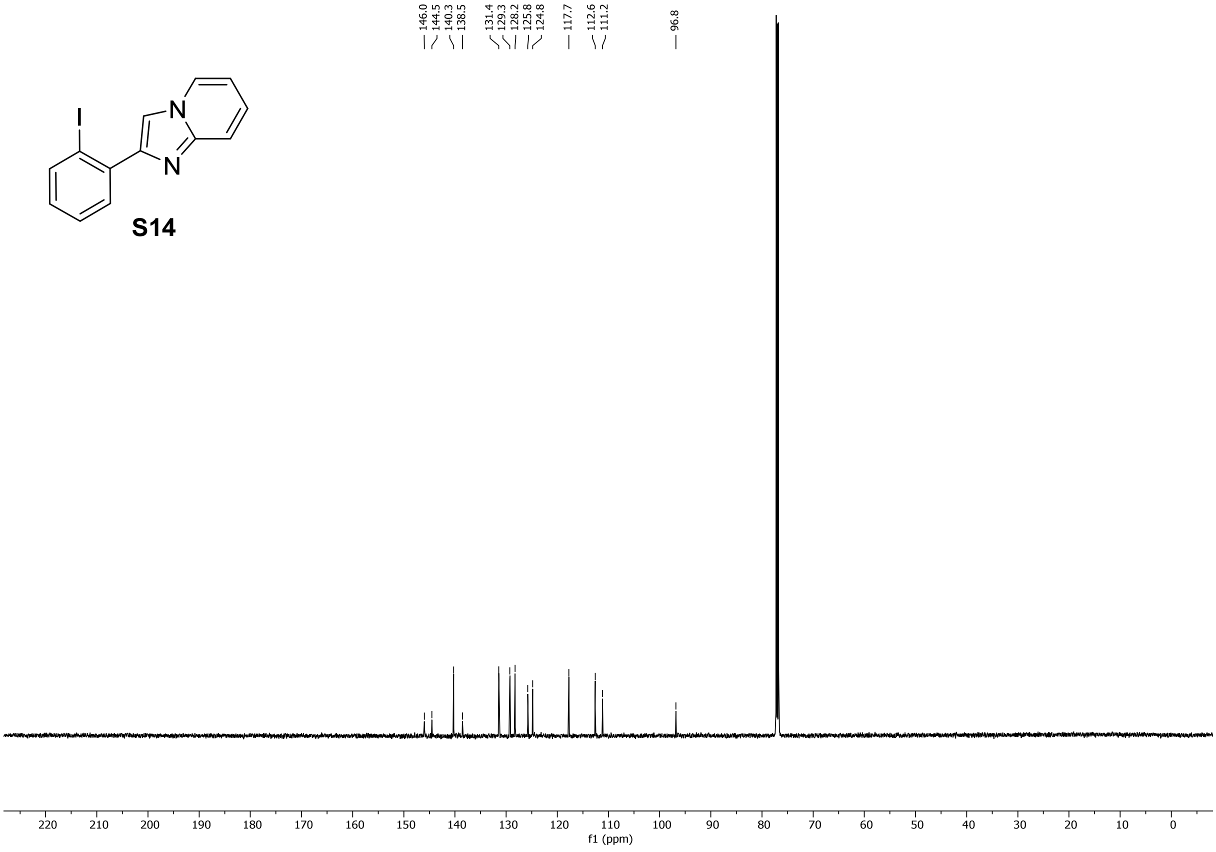

*Figure S29: 600 MHz ^{1}H- and 151 MHz ^{13}C-NMR spectra of 2-(2-iodophenyl)imidazo[1,2-a]pyridine (**S14**) in $CDCl_3$.*

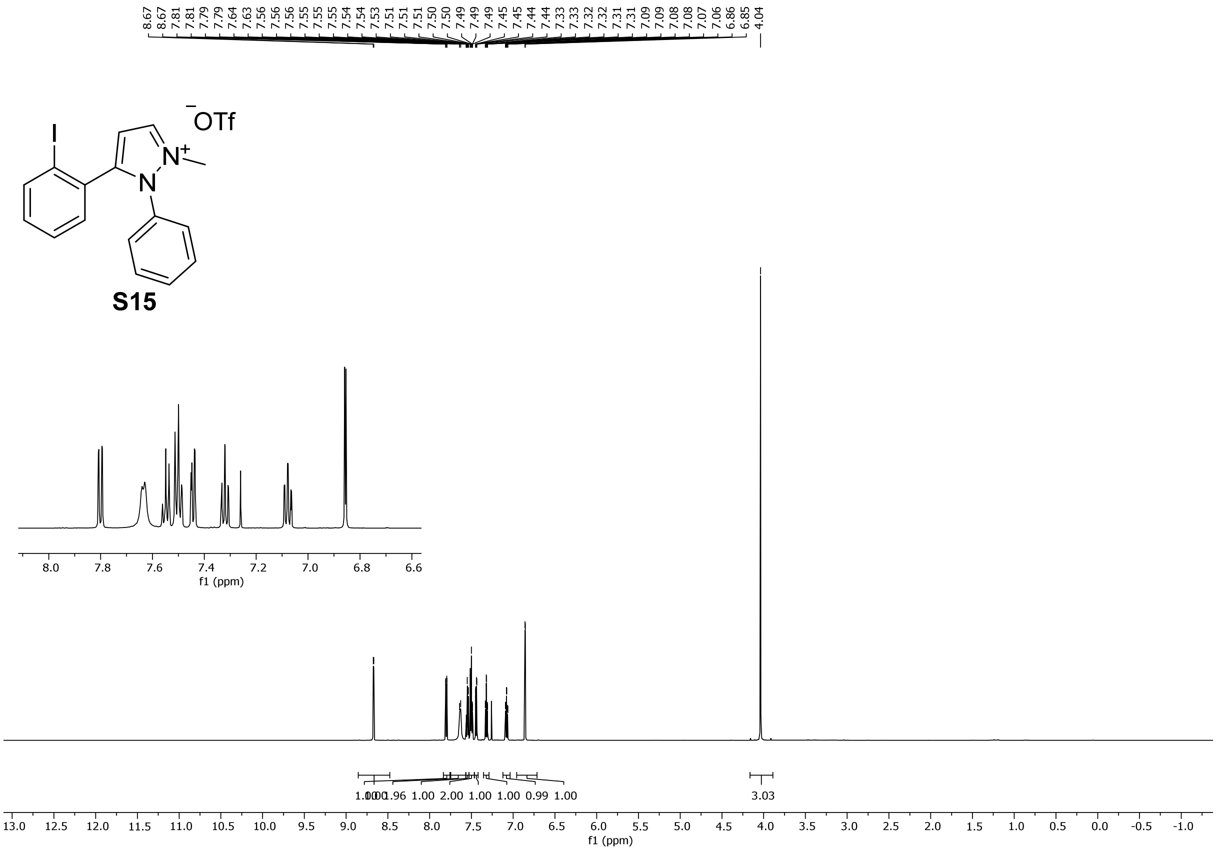
8.67 8.67 7.81 7.81 7.79 7.79 7.64 7.63 7.56 7.56 7.55 7.55 7.55 7.54 7.54 7.53 7.51 7.51 7.51 7.50 7.50 7.49 7.49 7.49 7.45 7.45 7.44 7.44 7.33 7.33 7.32 7.32 7.31 7.31 7.09 7.09 7.08 7.08 7.07 7.06 6.86 6.85 4.04
I
OTf
N+
N
S15
1.00 1.00 1.96 1.00 2.00 1.00 1.00 0.99 1.00
3.03
f1 (ppm)

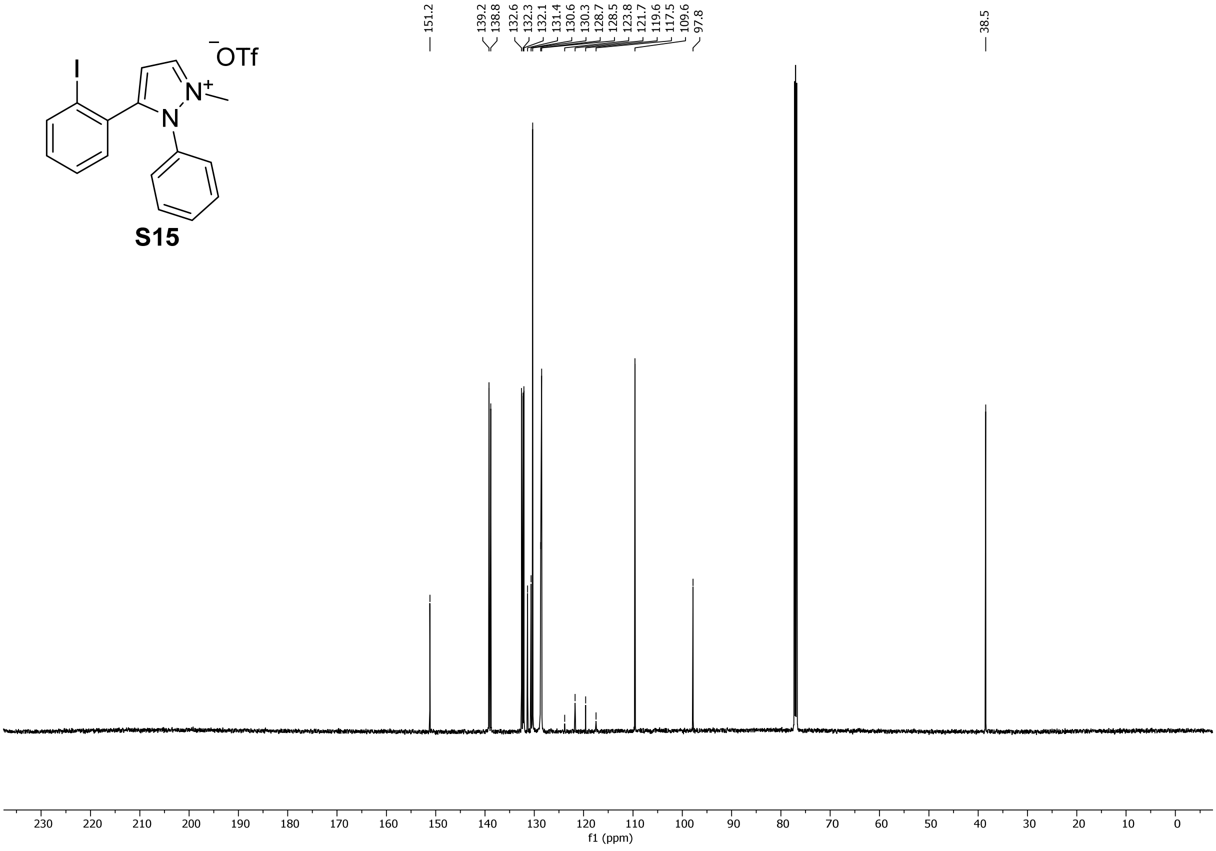
151.2
139.2 138.8 132.6 132.3 132.1 131.4 130.6 130.3 128.7 128.5 123.8 121.7 119.6 117.5 109.6 97.8
38.5
I
OTf
N+
N
S15
f1 (ppm)

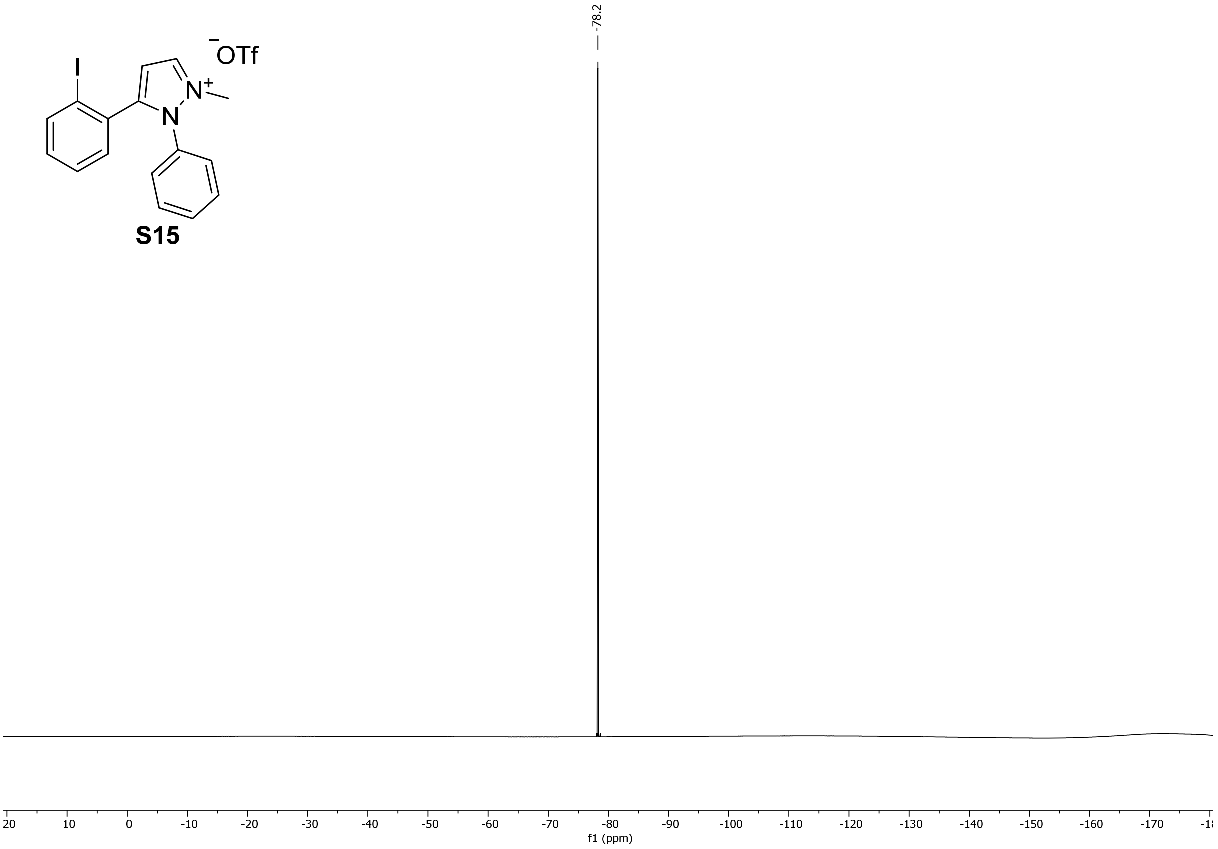

*Figure S30: 600 MHz ^{1}H- 151 MHz ^{13}C- and 565 MHz ^{19}F-NMR spectra of 5-(2-iodophenyl)-2-methyl-1-phenyl-1H-pyrazol-2-ium triflate (**S15**) in $CDCl_3$.*

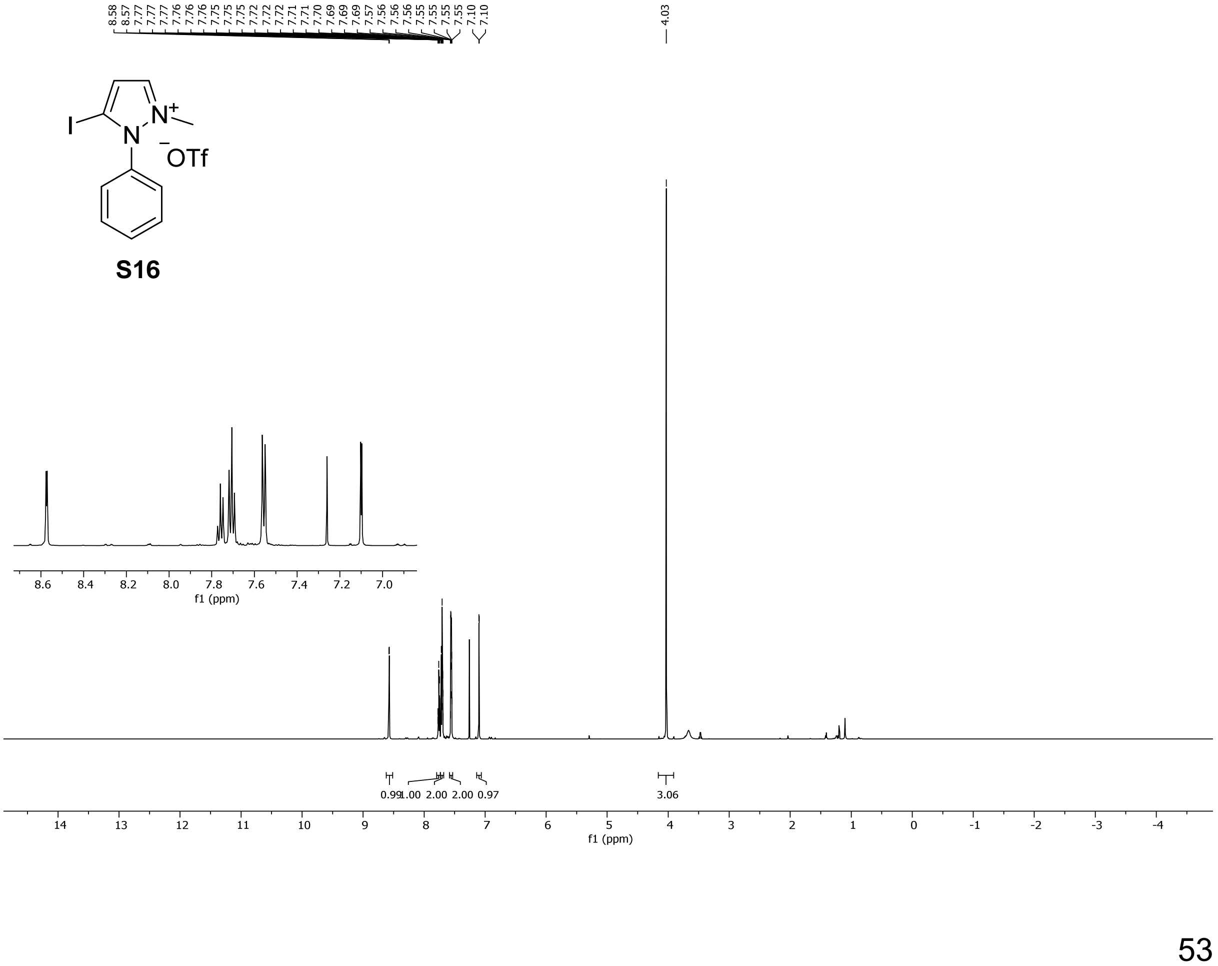

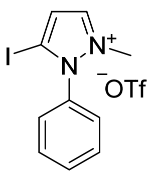

S16

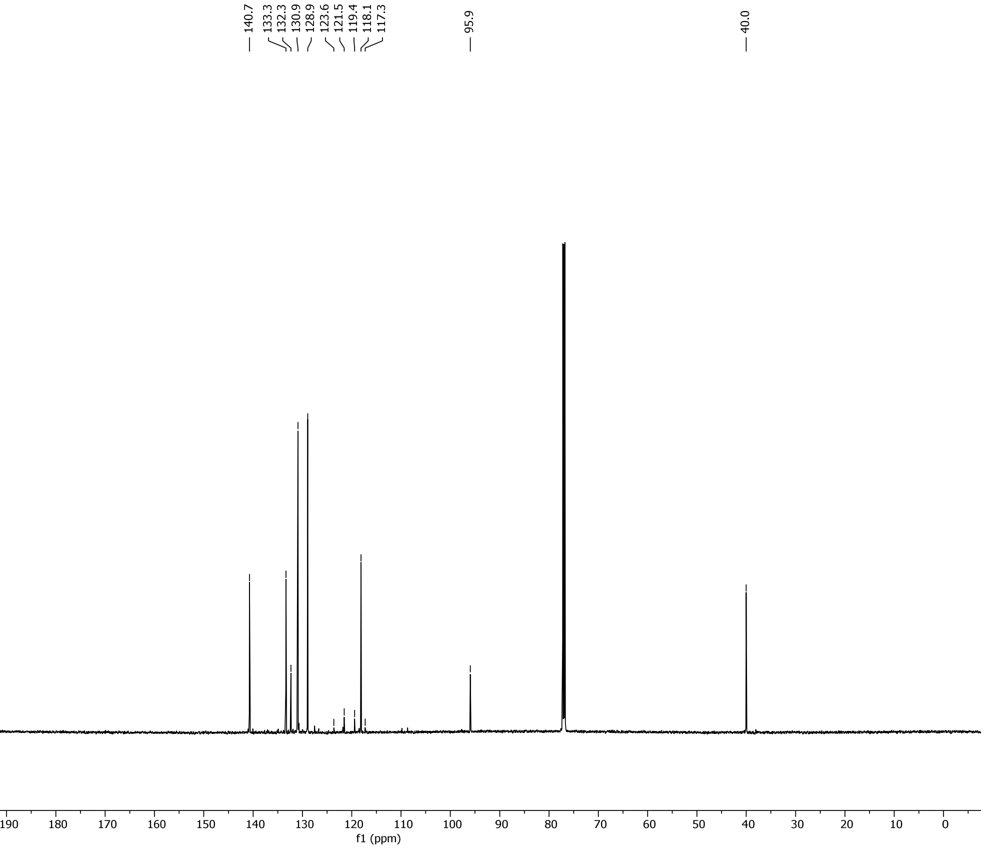

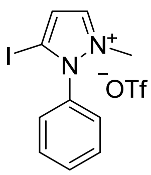

S16

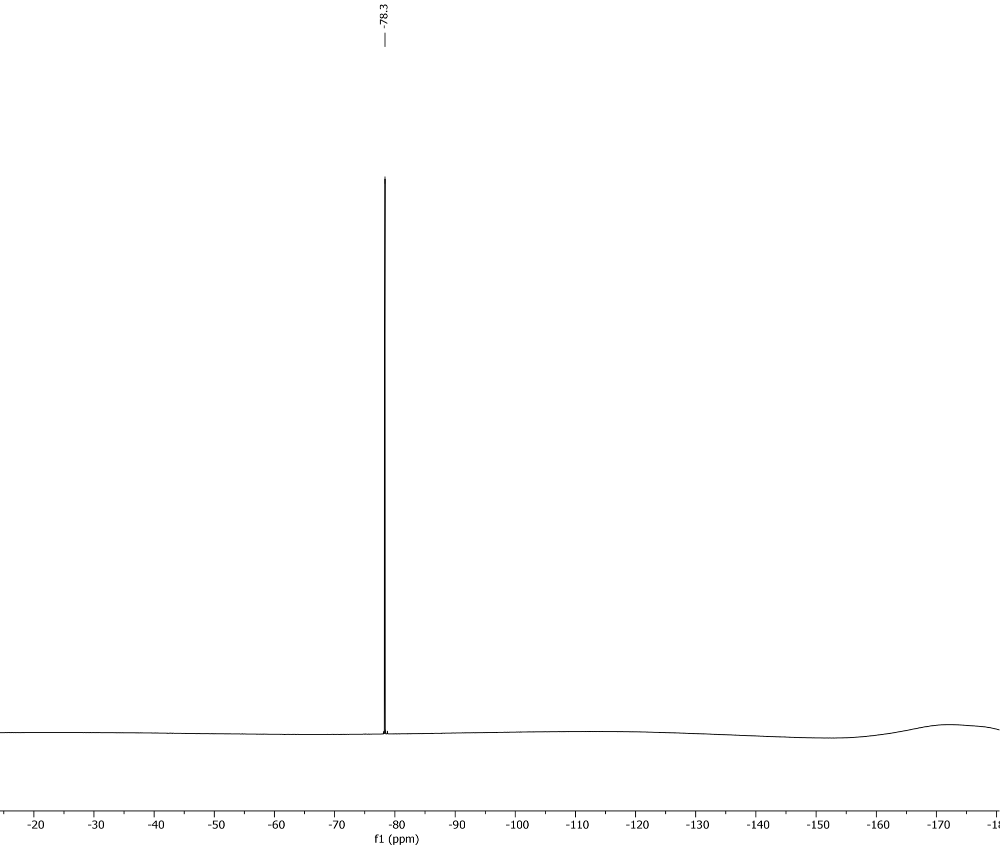

*Figure S31: 600 MHz ^{1}H-, 151 MHz ^{13}C- and 565 MHz ^{19}F-NMR spectra of 5-iodo-2-methyl-1-phenyl-1H-pyrazol-2-ium triflate (**S16**) in $CDCl_3$.*

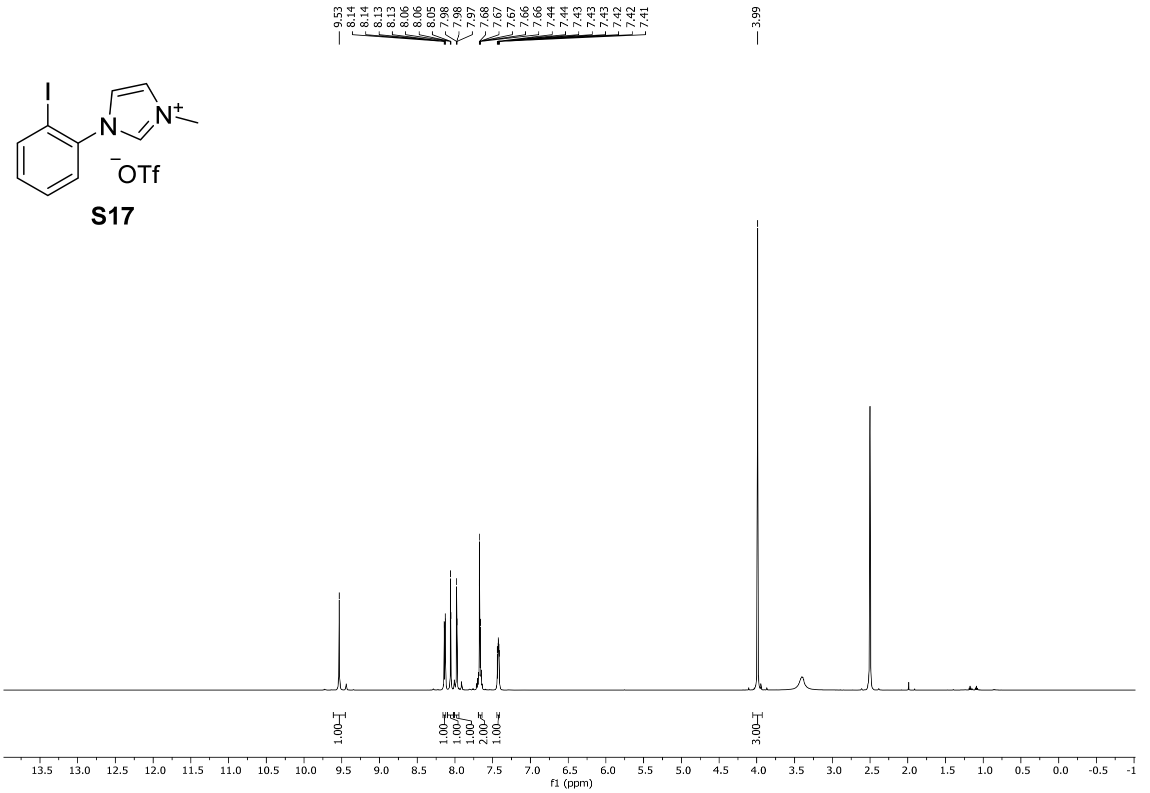
I
N
N+
−OTf
S17
9.53
8.14
8.14
8.13
8.13
8.06
8.06
8.05
7.98
7.98
7.97
7.68
7.67
7.67
7.66
7.66
7.44
7.44
7.43
7.43
7.42
7.42
7.41
3.99
1.00
1.00
1.00
1.00
2.00
1.00
3.00
13.5 13.0 12.5 12.0 11.5 11.0 10.5 10.0 9.5 9.0 8.5 8.0 7.5 7.0 6.5 6.0 5.5 5.0 4.5 4.0 3.5 3.0 2.5 2.0 1.5 1.0 0.5 0.0 -0.5 -1
f1 (ppm)

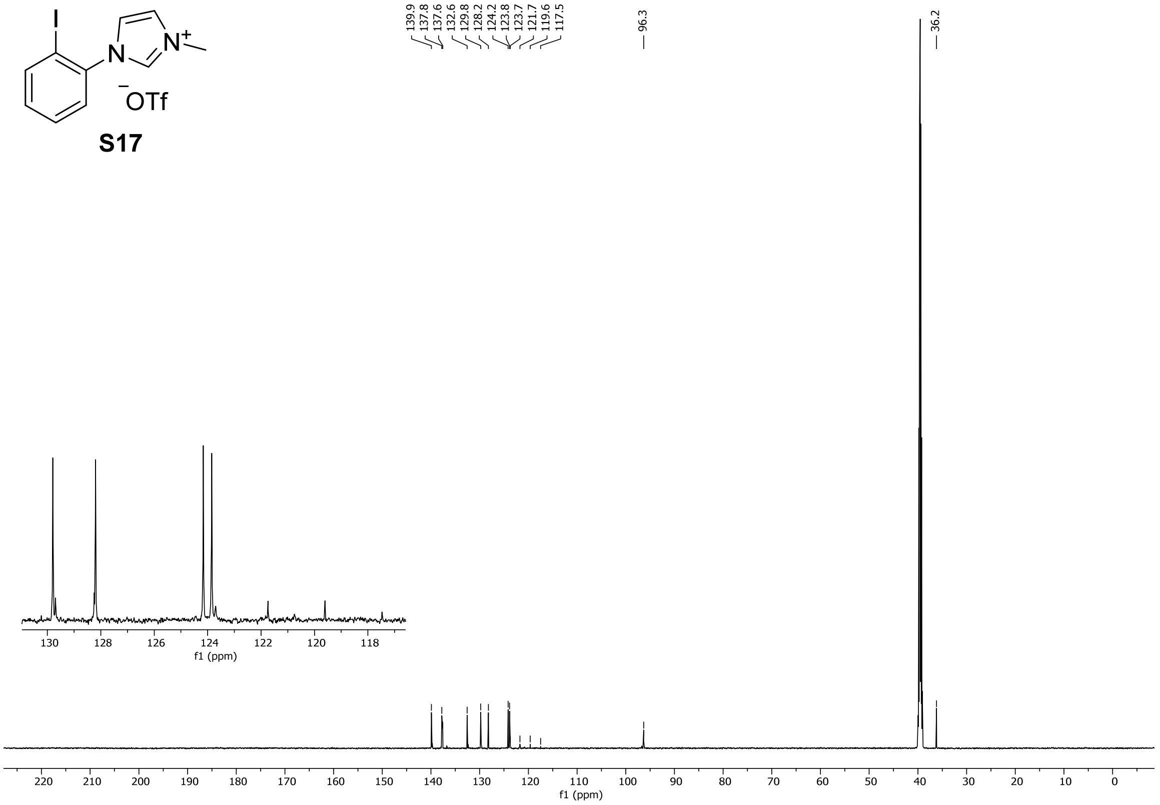
I
N
N+
−OTf
S17
139.9
137.8
137.6
132.6
129.8
128.2
124.2
123.8
123.7
121.7
119.6
117.5
96.3
36.2
130 128 126 124 122 120 118
f1 (ppm)
220 210 200 190 180 170 160 150 140 130 120 110 100 90 80 70 60 50 40 30 20 10 0
f1 (ppm)

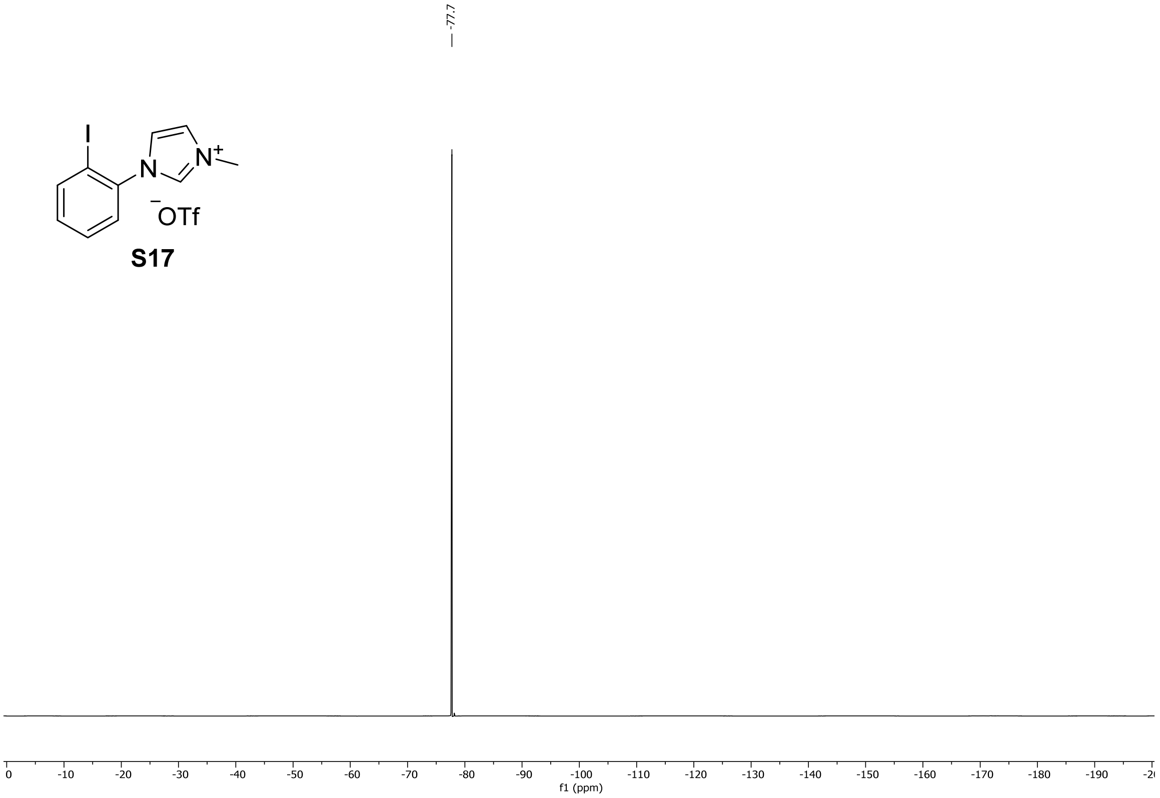

*Figure S32: 600 MHz ^{1}H-, 151 MHz ^{13}C- and 565 MHz ^{19}F-NMR spectra of 1-(2-iodophenyl)-3-methyl-1H-imidazol-3-ium triflate (**S17**) in d_6-DMSO.*

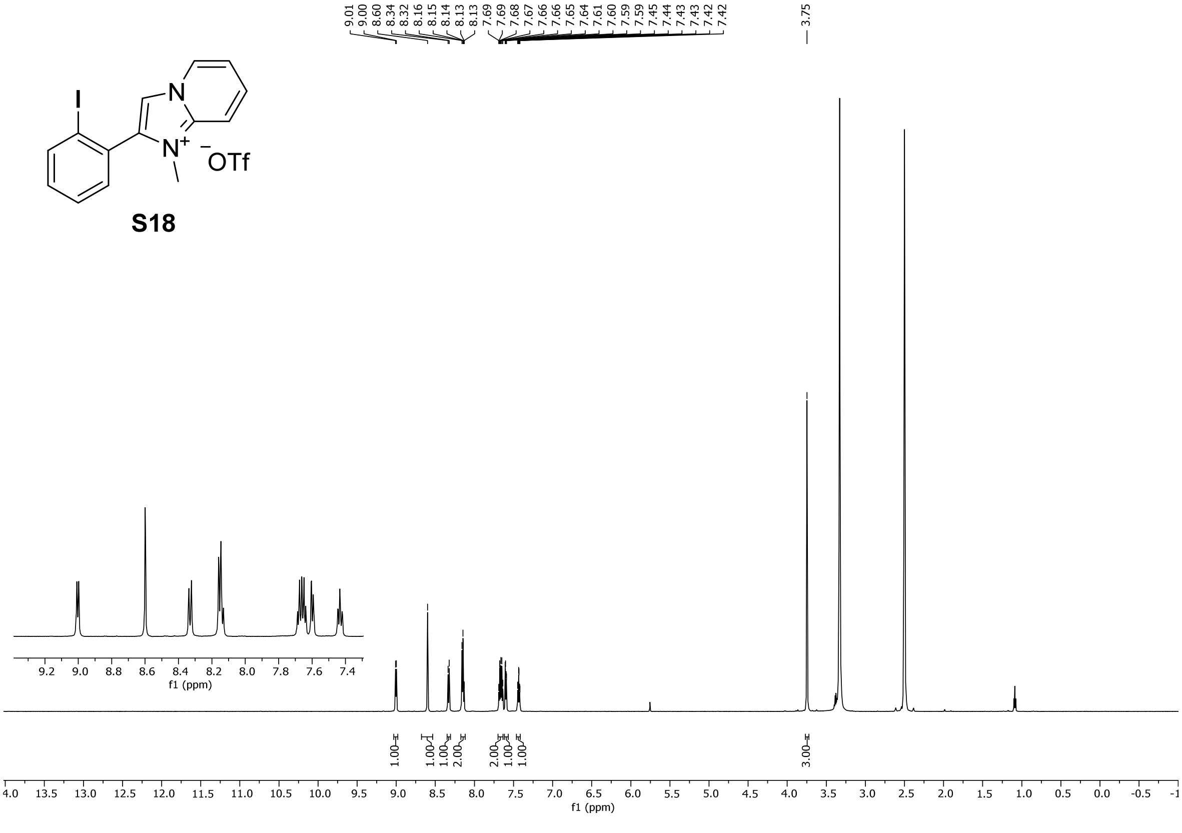

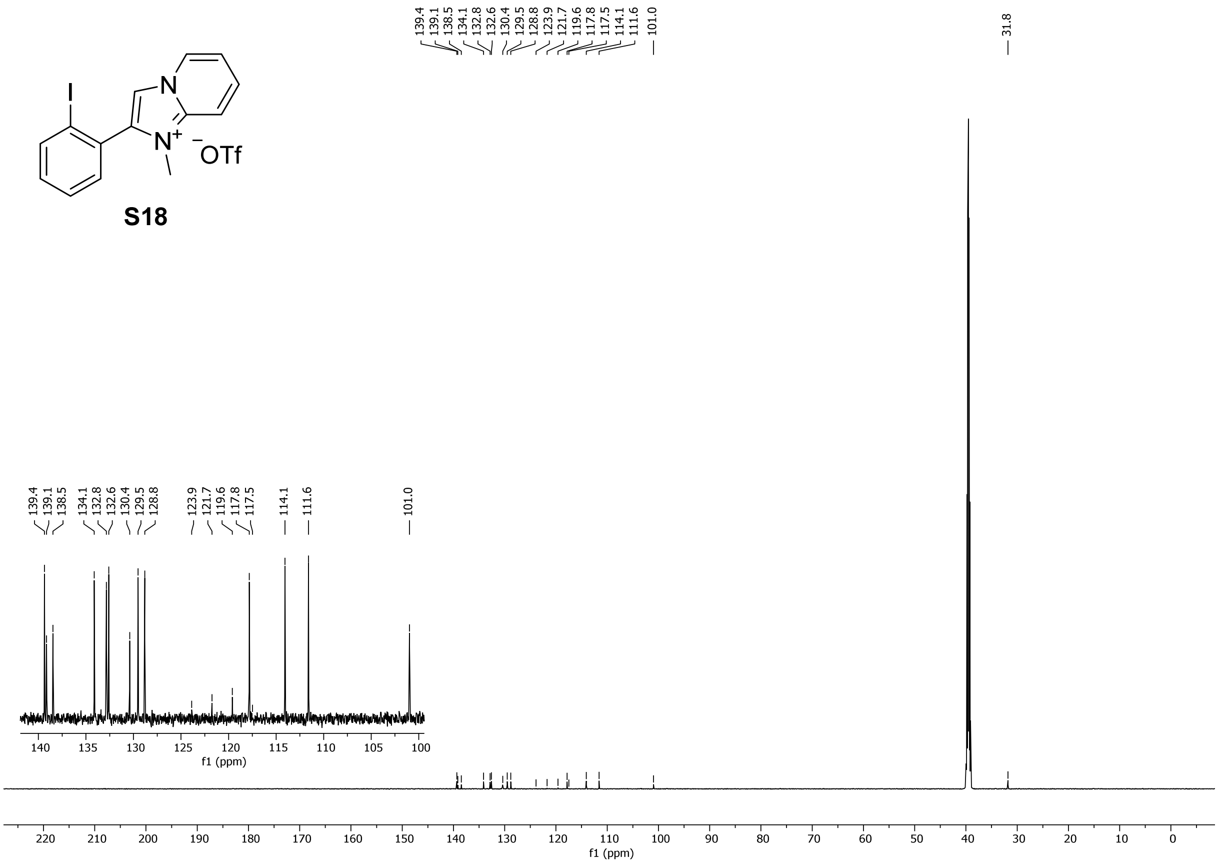

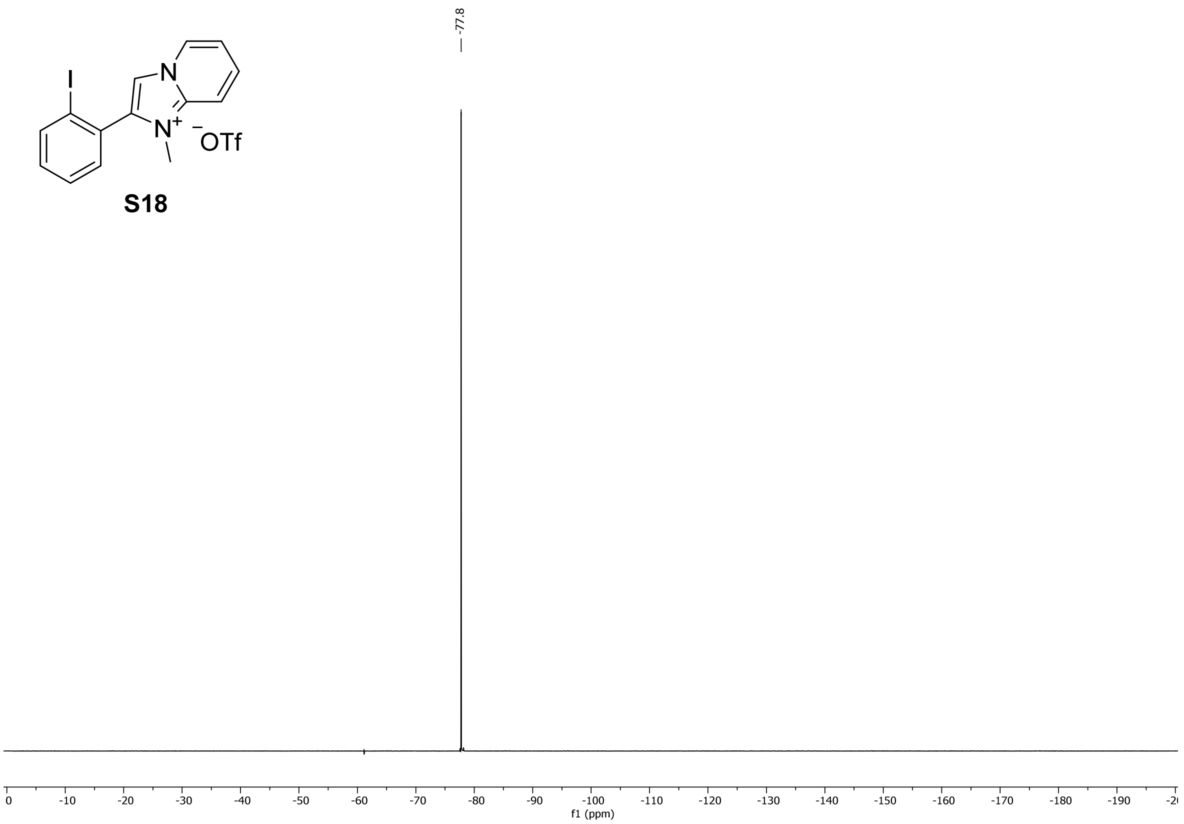

Figure S33: 600 MHz ^{1}H-, 151 MHz ^{13}C- and 565 MHz ^{19}F-NMR spectra of 2-(2-iodophenyl)-1-methylimidazo[1,2-a]pyridin-1-ium triflate (**S18**) in d_6-DMSO.

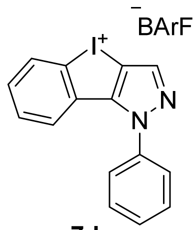
BArF
I+
N
N

7d

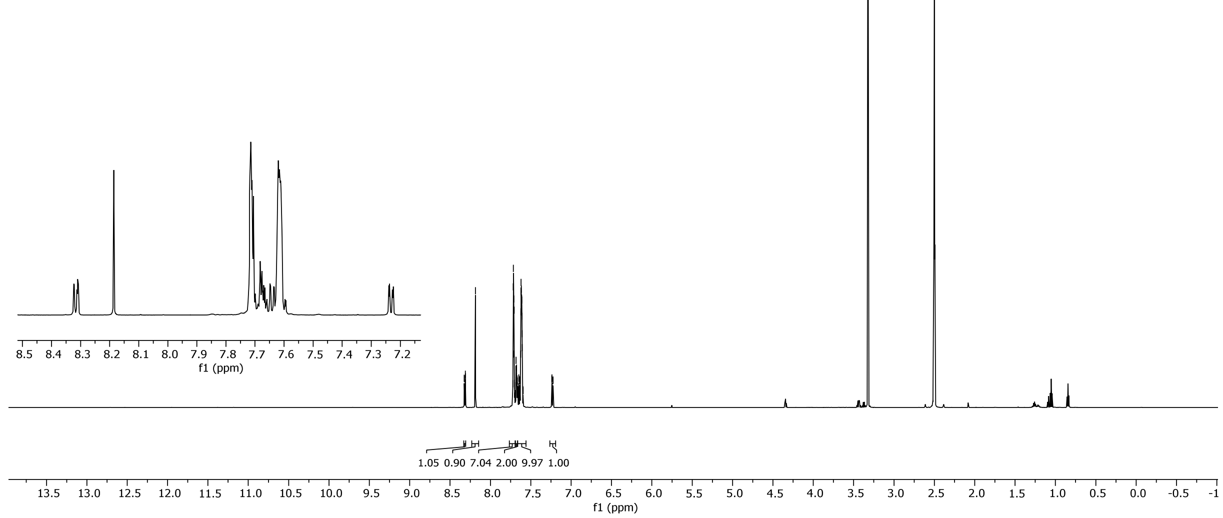
8.32 8.32 8.31 8.31 8.19 7.72 7.72 7.71 7.71 7.71 7.70 7.69 7.69 7.68 7.68 7.68 7.67 7.67 7.67 7.66 7.65 7.64 7.63 7.63 7.62 7.62 7.61 7.61 7.61 7.60 7.59 7.24 7.23 7.23 7.22
1.05 0.90 7.04 2.00 9.97 1.00
f1 (ppm)

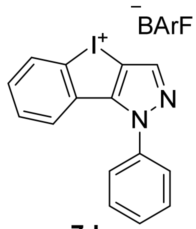
BArF
I+
N
N

7d

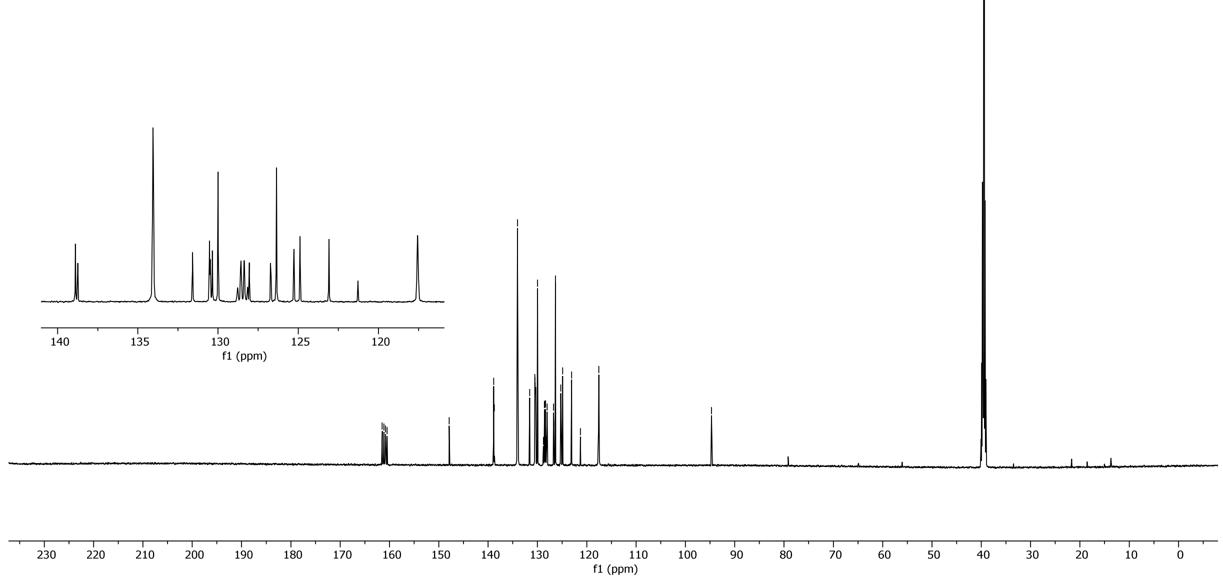
161.5 161.1 160.8 160.5 147.9 138.9 138.7 134.1 131.6 130.5 130.5 130.4 130.0 128.8 128.8 128.6 128.6 128.4 128.4 128.2 128.1 128.0 126.7 126.7 126.4 125.3 124.9 123.1 121.3 117.6 94.7
f1 (ppm)

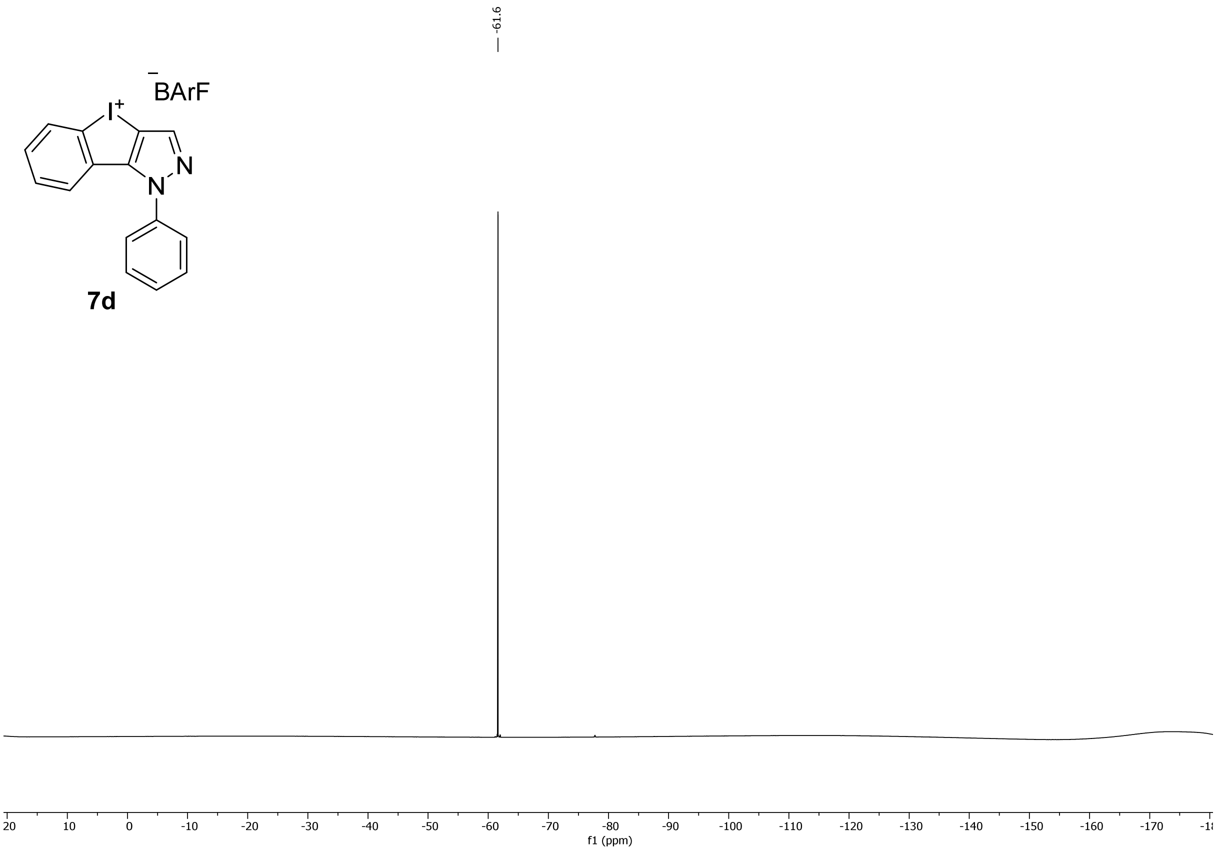

*Figure S34: 600 MHz ^{1}H-, 151 MHz ^{13}C- and 565 MHz ^{19}F-NMR spectra of 1-phenyl-1H-benzo[4,5]iodolo[3,2-c]pyrazol-4-ium tetrakis(3,5-bis(trifluoromethyl)phenyl)borate (**7d**) in d$_6$-DMSO.*

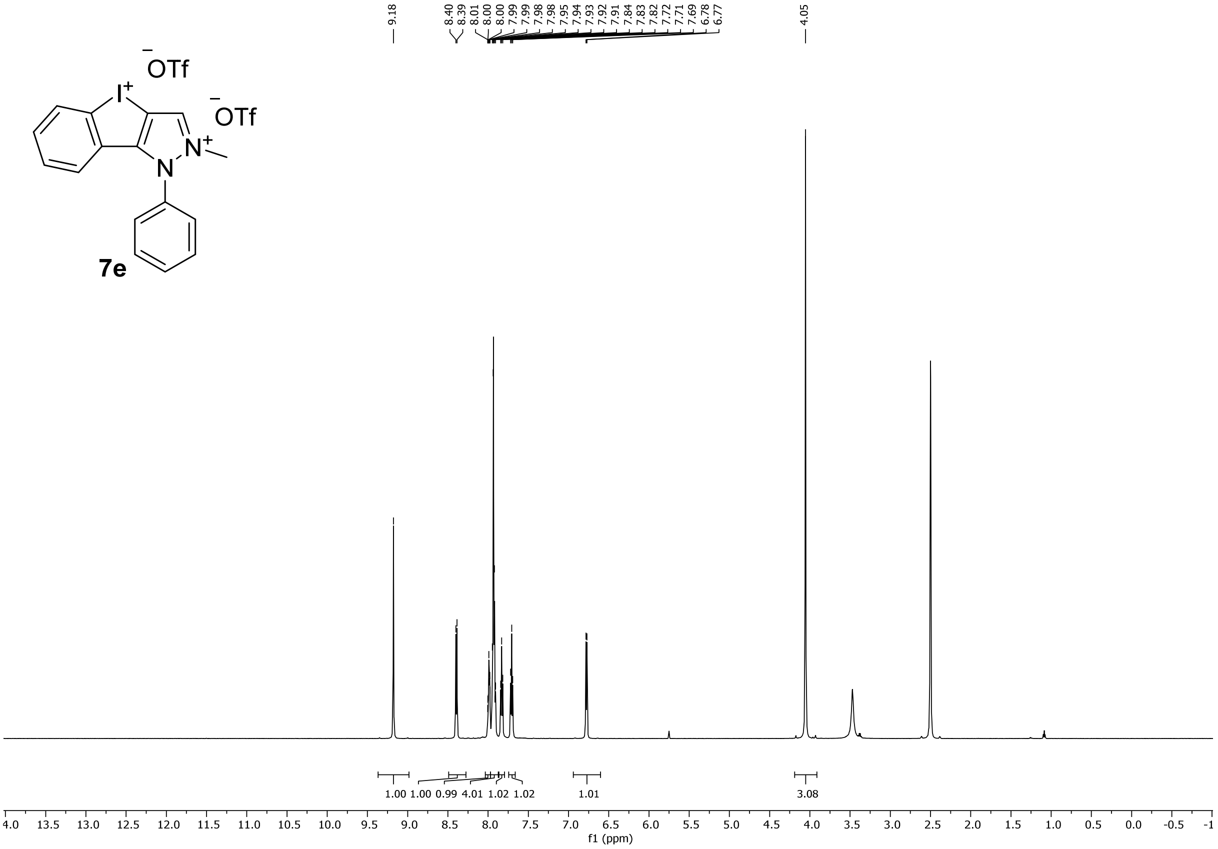

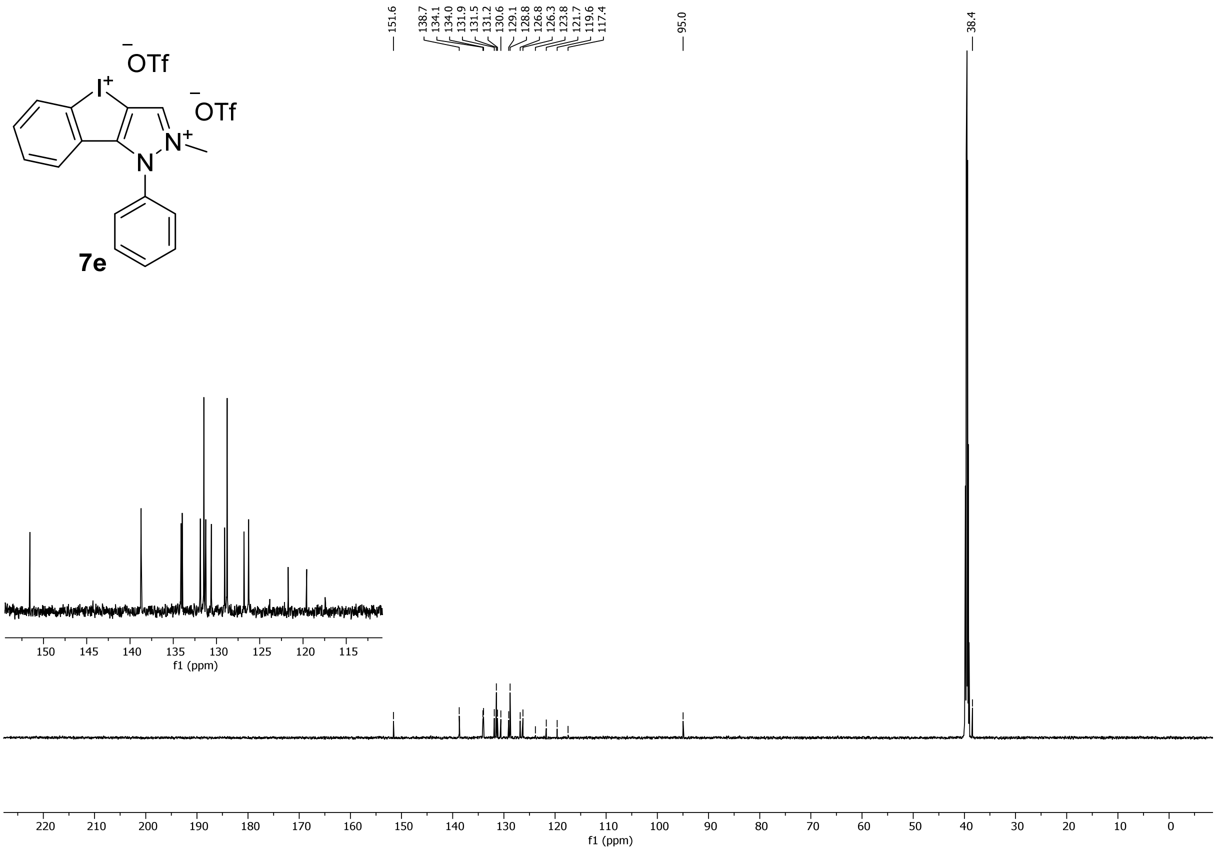

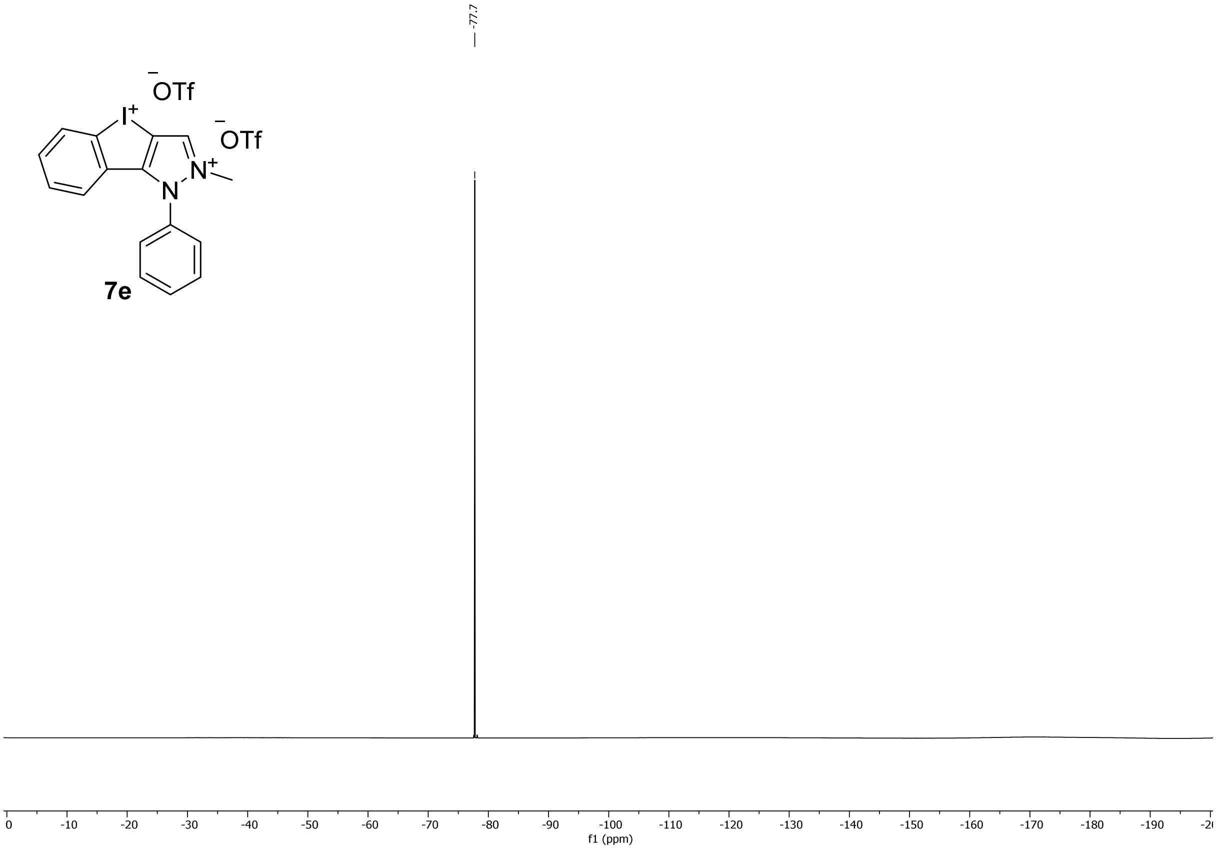

*Figure S35: 600 MHz ^{1}H-, 151 MHz ^{13}C- and 565 MHz ^{19}F-NMR spectra of 2-methyl-1-phenyl-1H-benzo[4,5]iodolo[3,2-c]pyrazole-2,4-diium bistriflate (**7e**) in d_6-DMSO.*

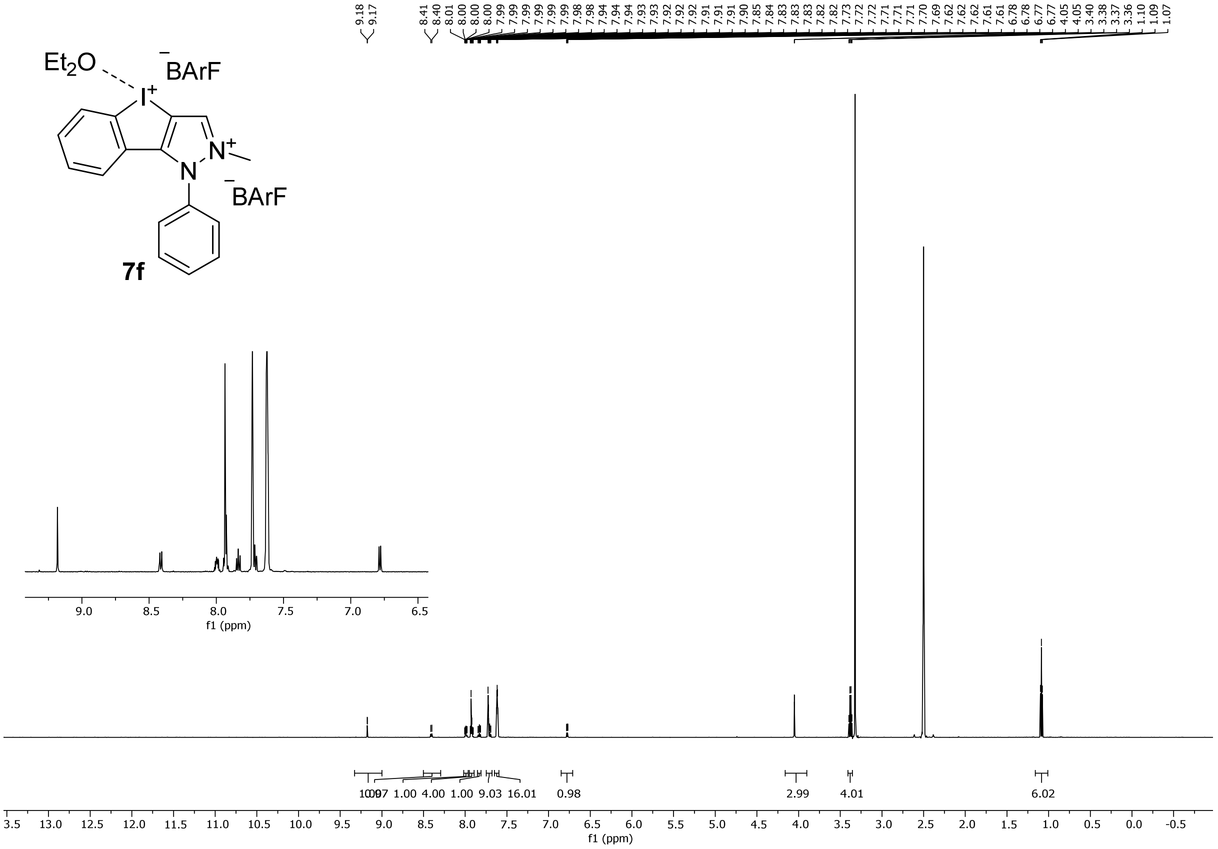
Et2O
BArF
I+
N+
N
BArF
7f
9.18 9.17 8.41 8.40 8.01 8.00 8.00 7.99 7.99 7.99 7.99 7.99 7.99 7.98 7.94 7.94 7.94 7.93 7.93 7.92 7.92 7.92 7.91 7.91 7.91 7.90 7.85 7.84 7.83 7.83 7.83 7.82 7.82 7.73 7.72 7.72 7.71 7.71 7.71 7.70 7.69 7.62 7.62 7.62 7.61 7.61 6.78 6.78 6.77 6.77 4.05 3.40 3.38 3.37 3.36 1.10 1.09 1.07
1.00 0.97 1.00 4.00 1.00 9.03 16.01 0.98 2.99 4.01 6.02
f1 (ppm)

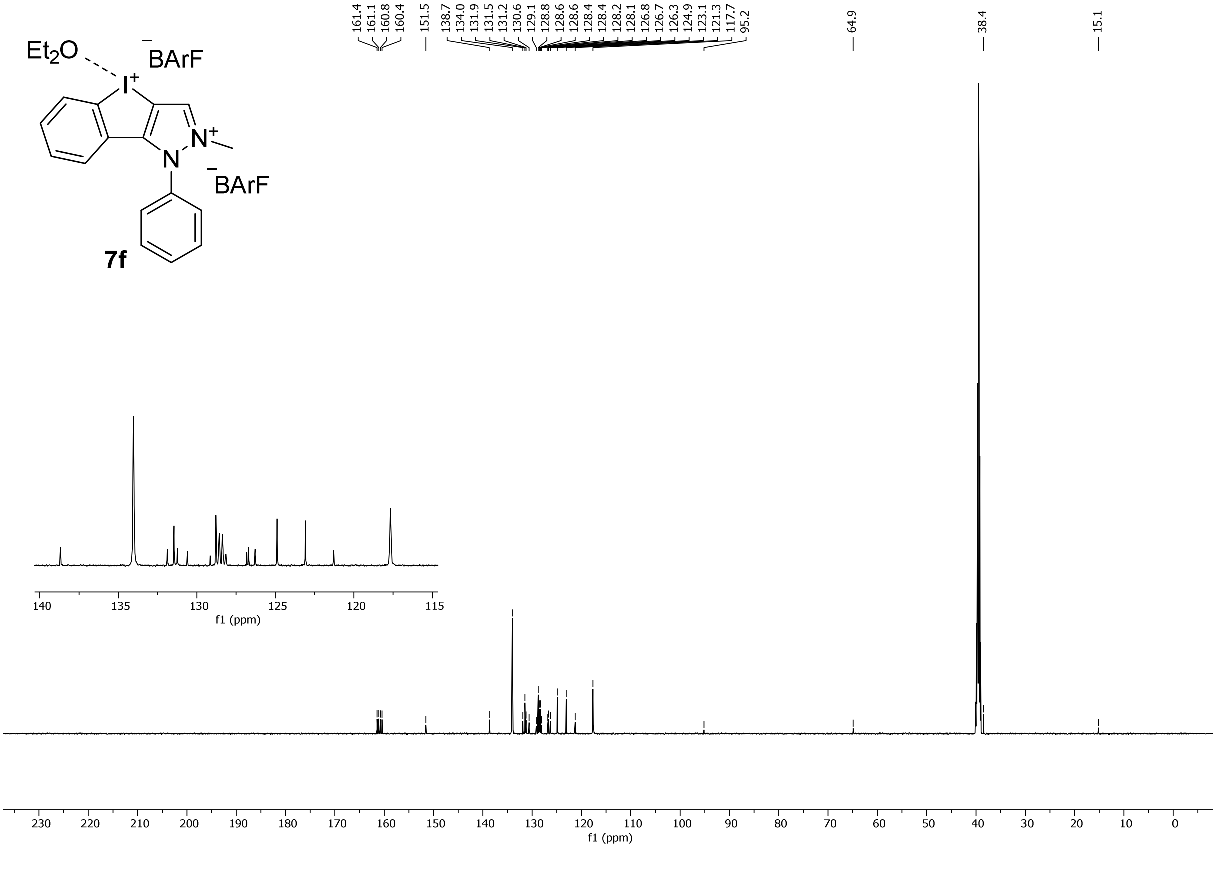
Et2O
BArF
I+
N+
N
BArF
7f
161.4 161.1 160.8 160.4 151.5 138.7 134.0 131.9 131.5 131.2 130.6 129.1 128.8 128.6 128.6 128.4 128.4 128.2 128.1 126.8 126.7 126.3 124.9 123.1 121.3 117.7 95.2 64.9 38.4 15.1
f1 (ppm)

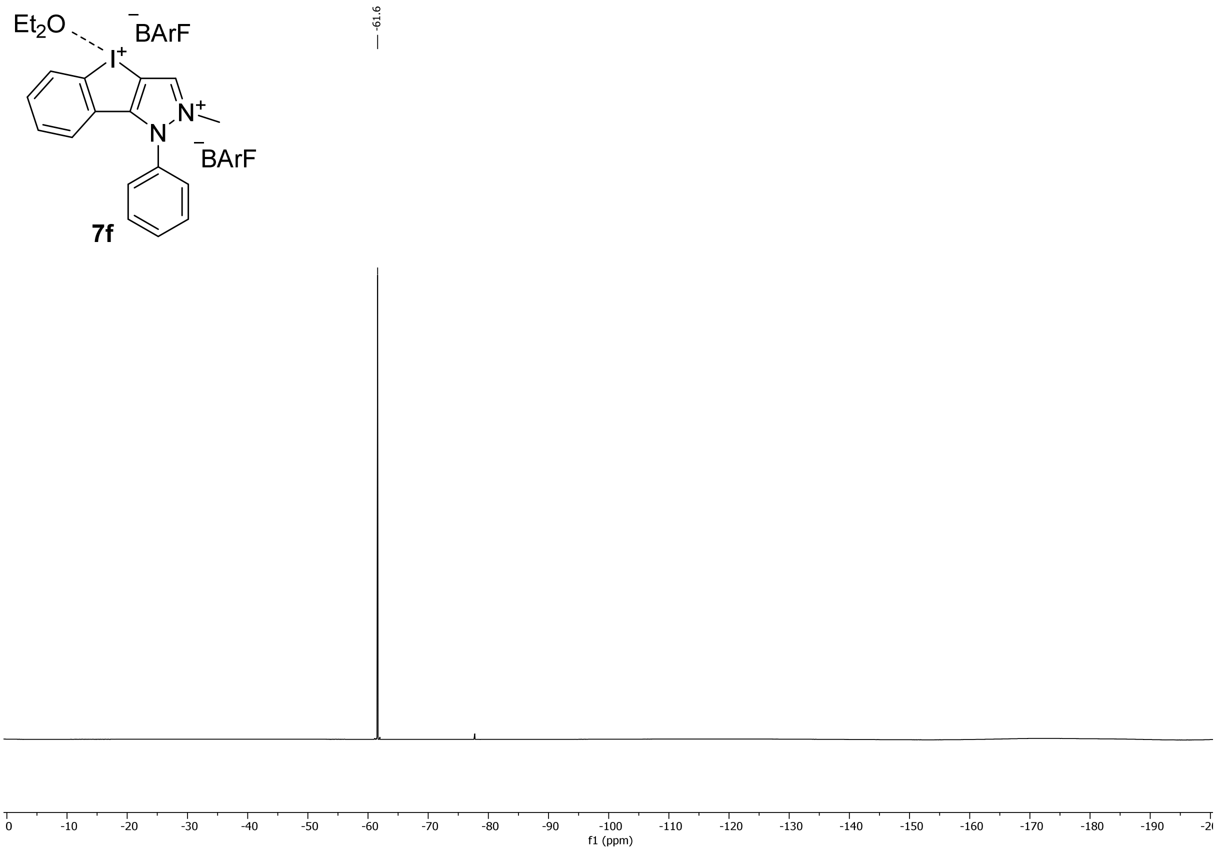

*Figure S36: 600 MHz ^{1}H-, 151 MHz ^{13}C- and 565 MHz ^{19}F-NMR spectra of 2-methyl-1-phenyl-1H-benzo[4,5]iodolo[3,2-c]pyrazole-2,4-diium bis(tetrakis(3,5-bis(trifluoromethyl)phenyl)borate) • Et_2O (**7f**) in d_6-DMSO.*

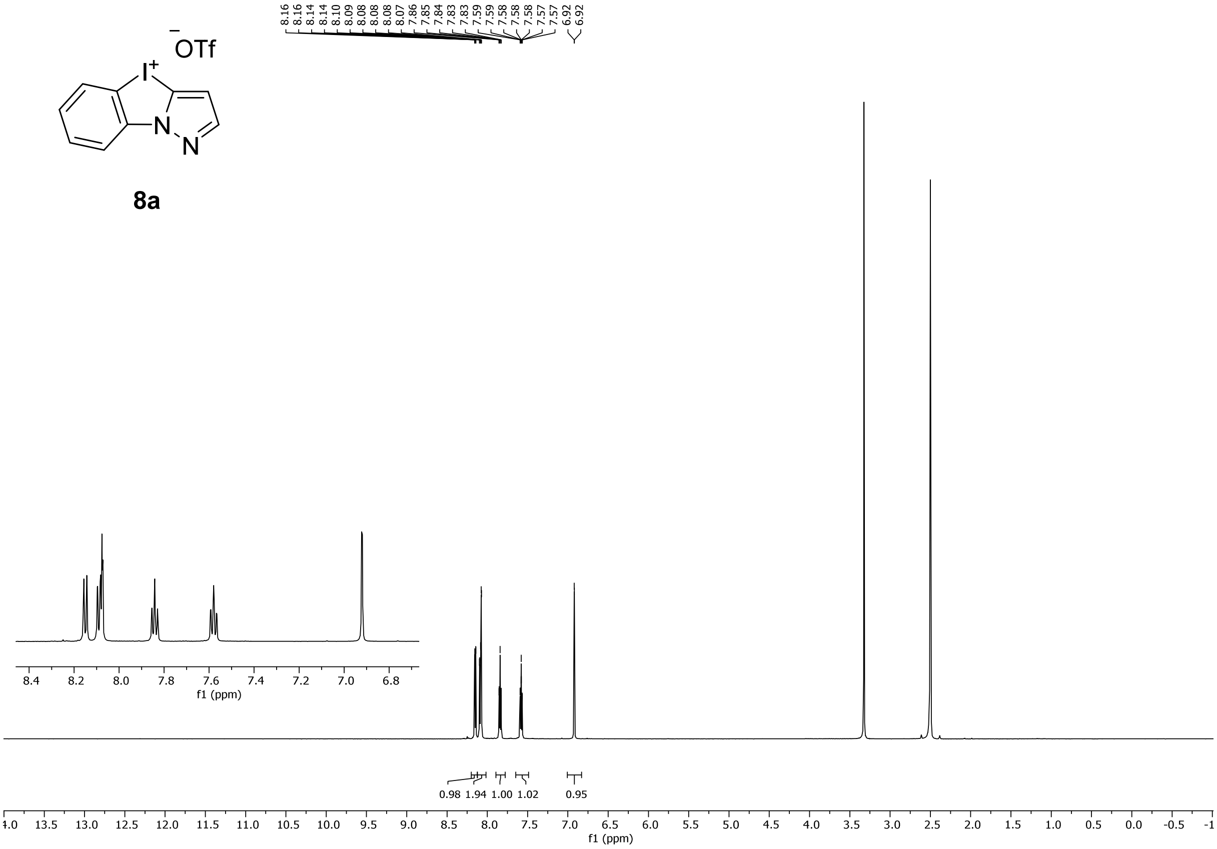

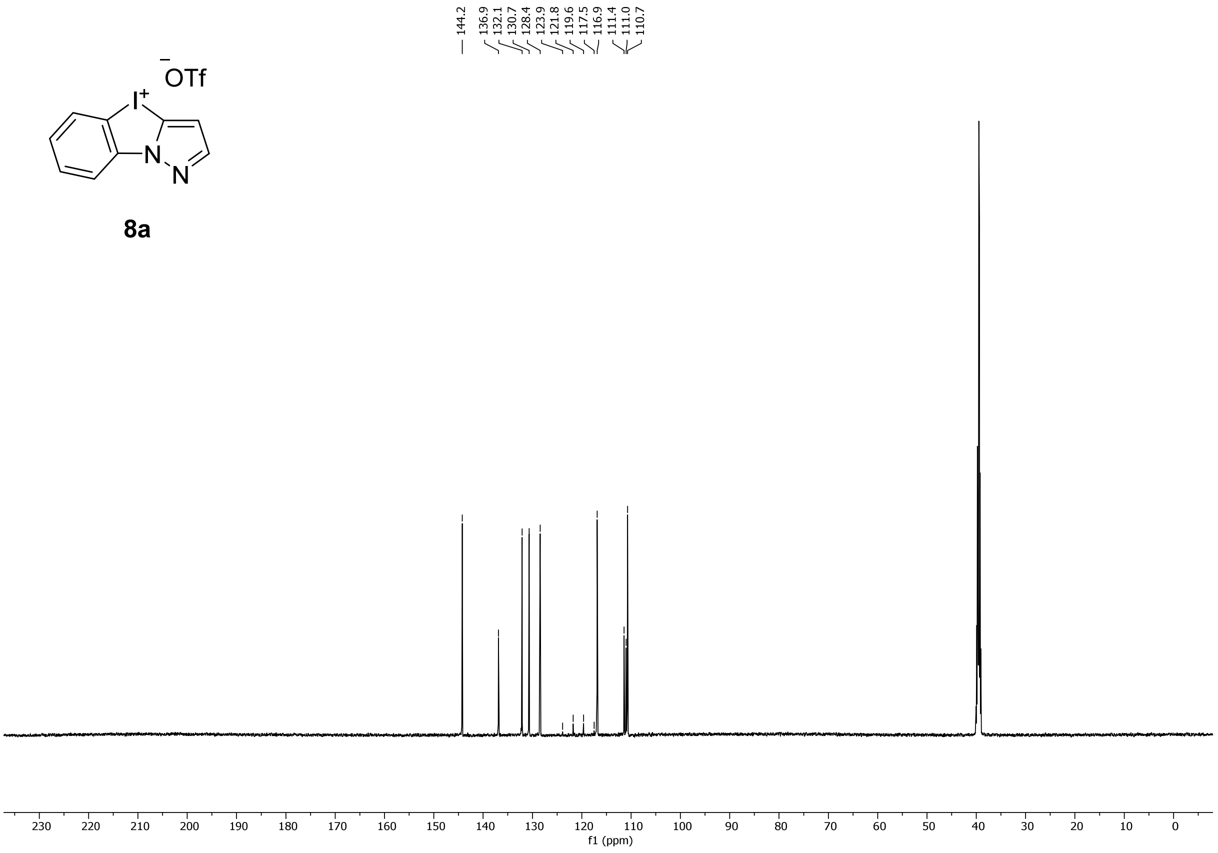
OTf
I+
N
N
8a
144.2
136.9
132.1
130.7
128.4
123.9
121.8
119.6
117.5
116.9
111.4
111.0
110.7
230 220 210 200 190 180 170 160 150 140 130 120 110 100 90 80 70 60 50 40 30 20 10 0
f1 (ppm)

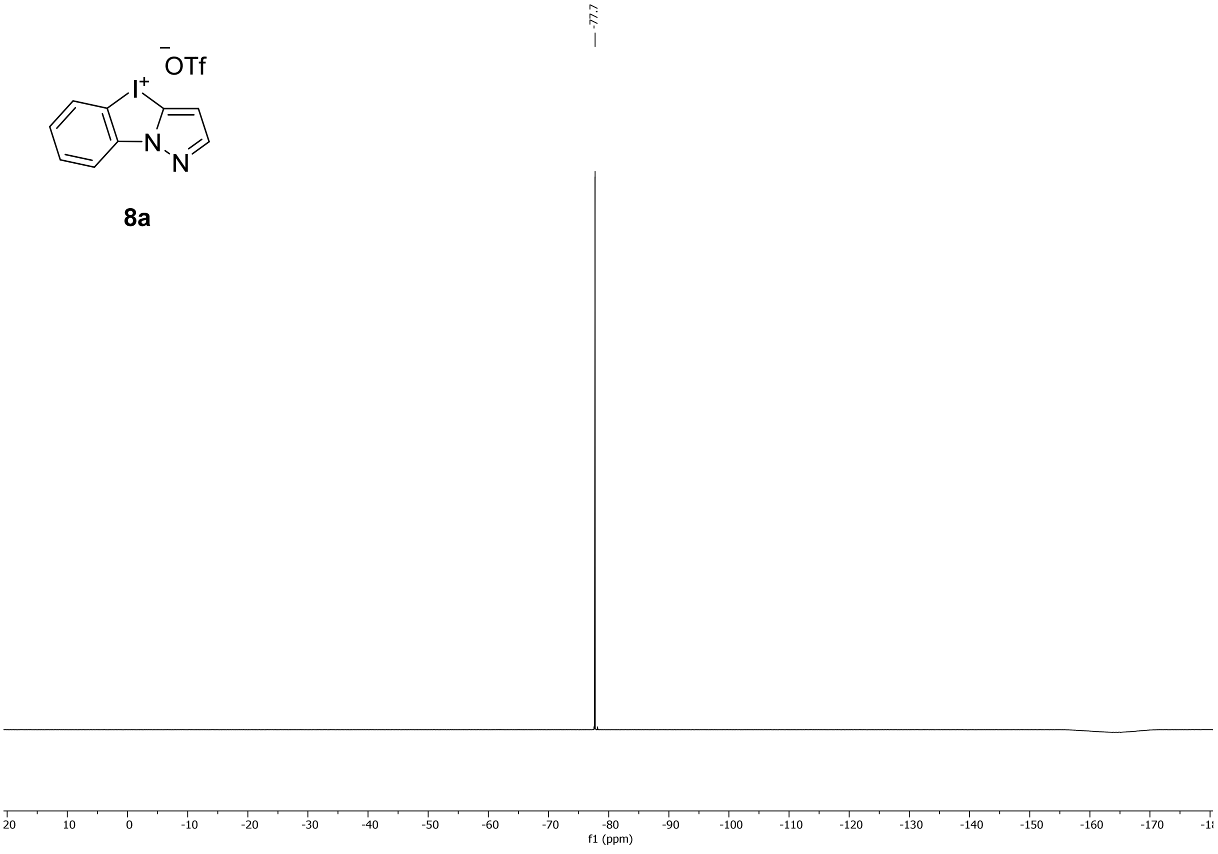
OTf
I+
N
N
8a
-77.7
20 10 0 -10 -20 -30 -40 -50 -60 -70 -80 -90 -100 -110 -120 -130 -140 -150 -160 -170
f1 (ppm)

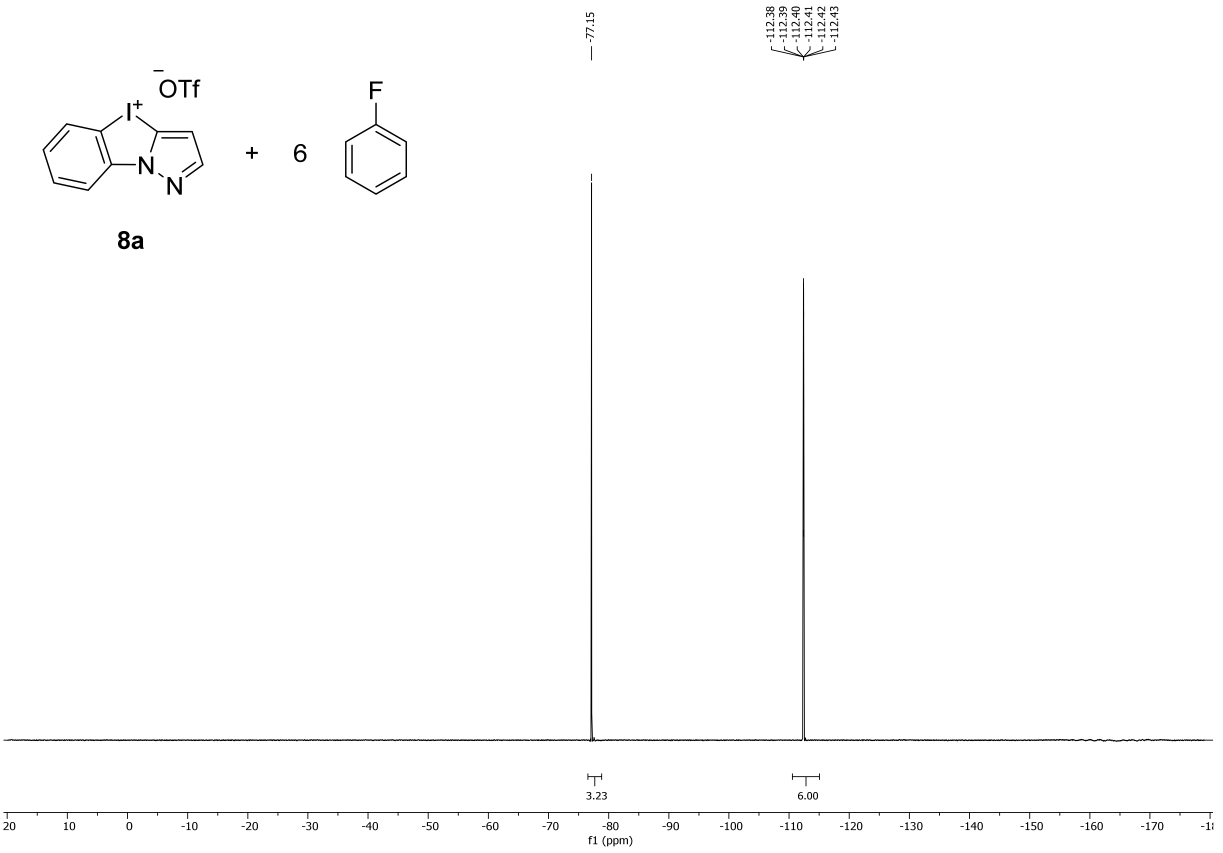

*Figure S37: 600 MHz ^{1}H-, 151 MHz ^{13}C- and 565 MHz ^{19}F-NMR (with and without 6 equiv. PhF as internal standard) spectra of benzo[d]pyrazolo[5,1-b][1,3]iodazol-4-ium triflate (**8a**) in d_6-DMSO.*

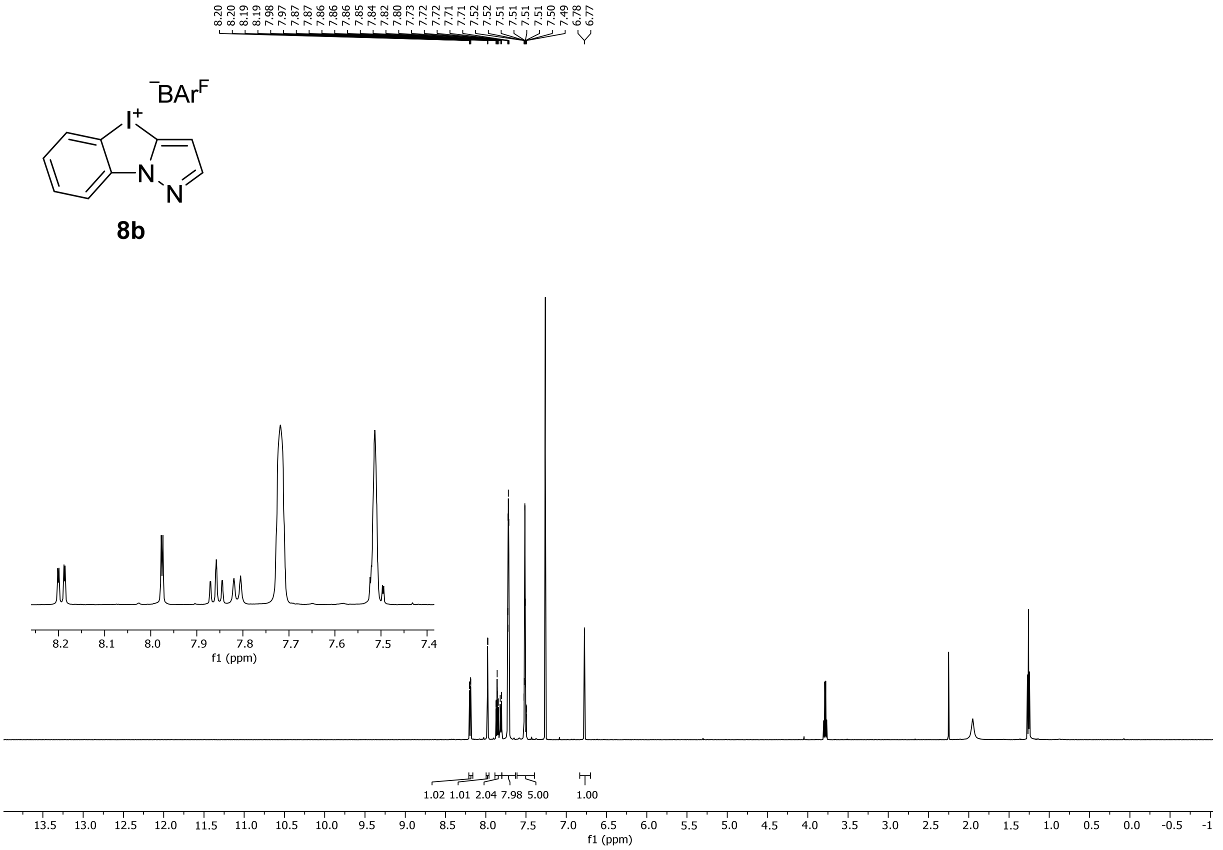

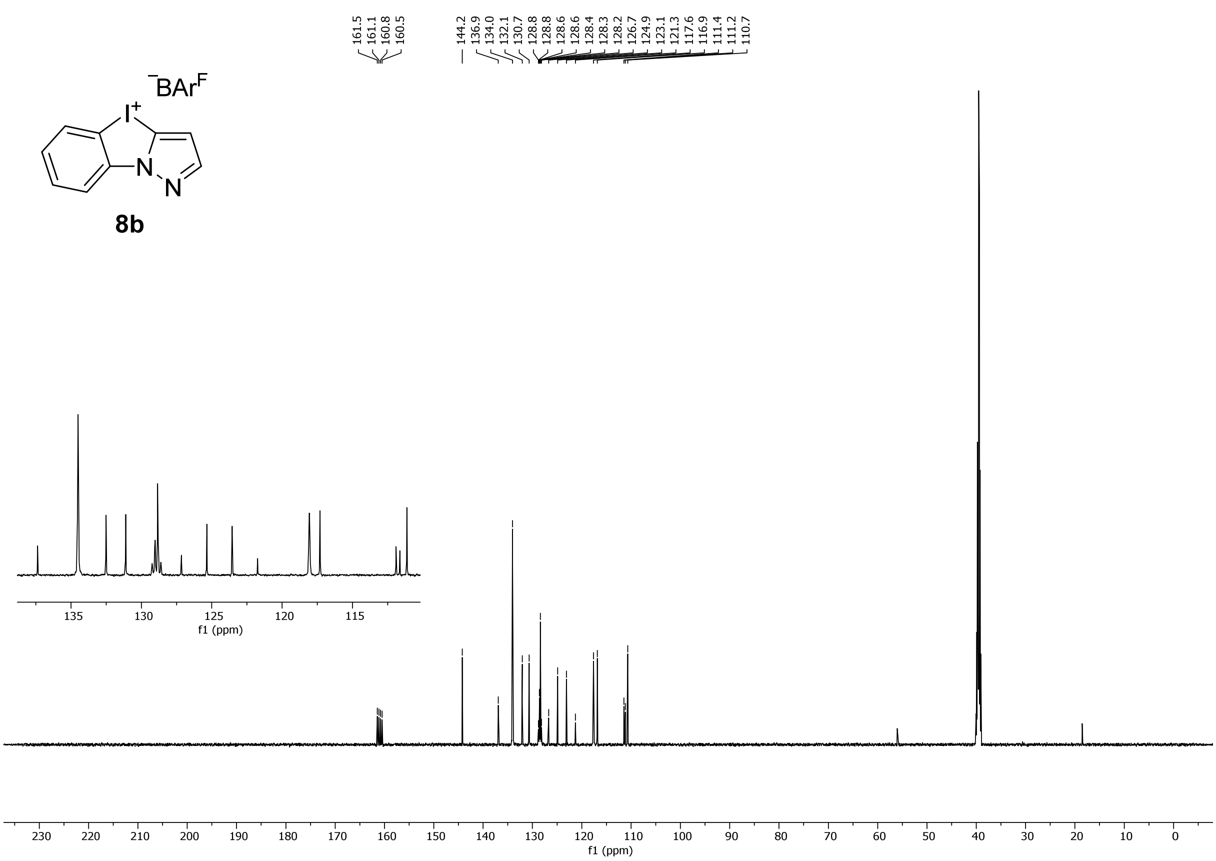

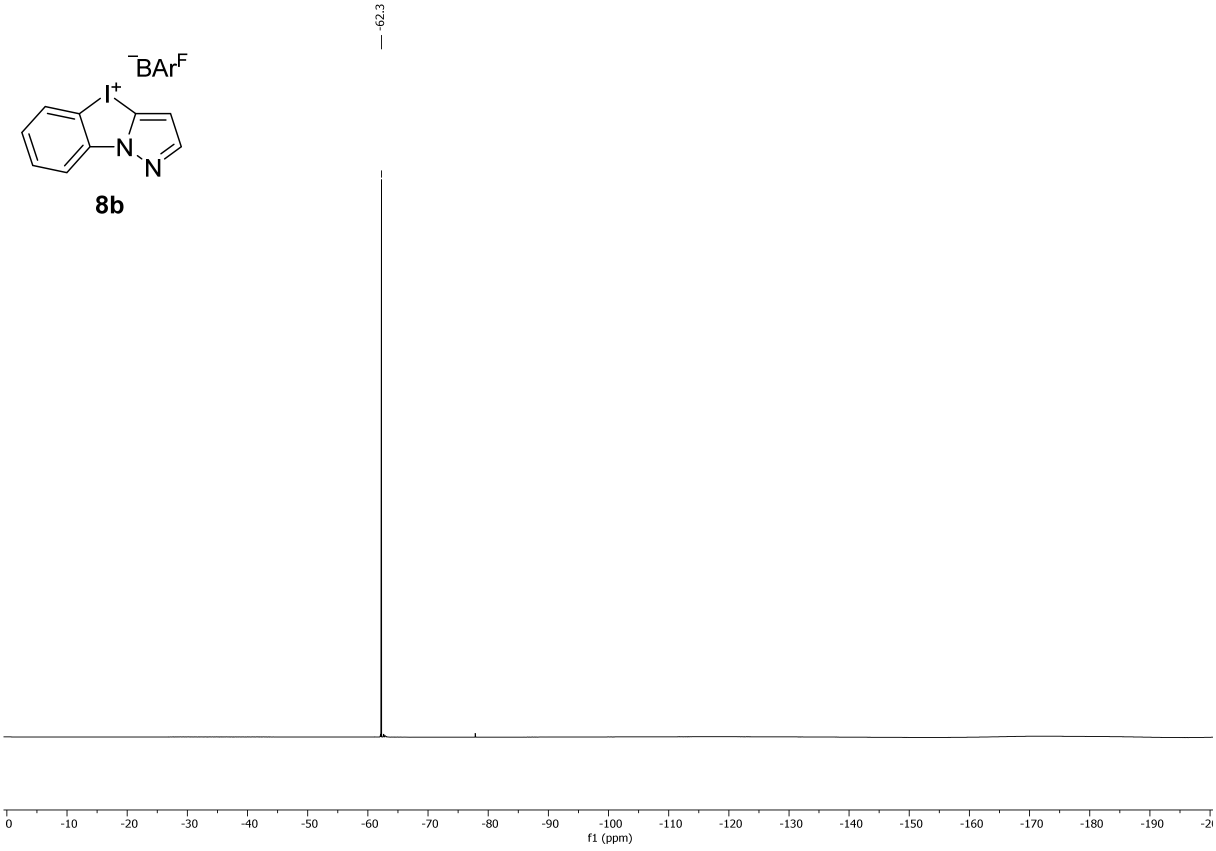

*Figure S38: 600 MHz ^{1}H-, 151 MHz ^{13}C- and 565 MHz ^{19}F-NMR spectra of benzo[d]pyrazolo[5,1-b][1,3]iodazol-4-ium tetrakis(3,5-bis(trifluoromethyl)phenyl)borate (**8b**) in $CDCl_3$ and in d_6-DMSO (^{13}C).*

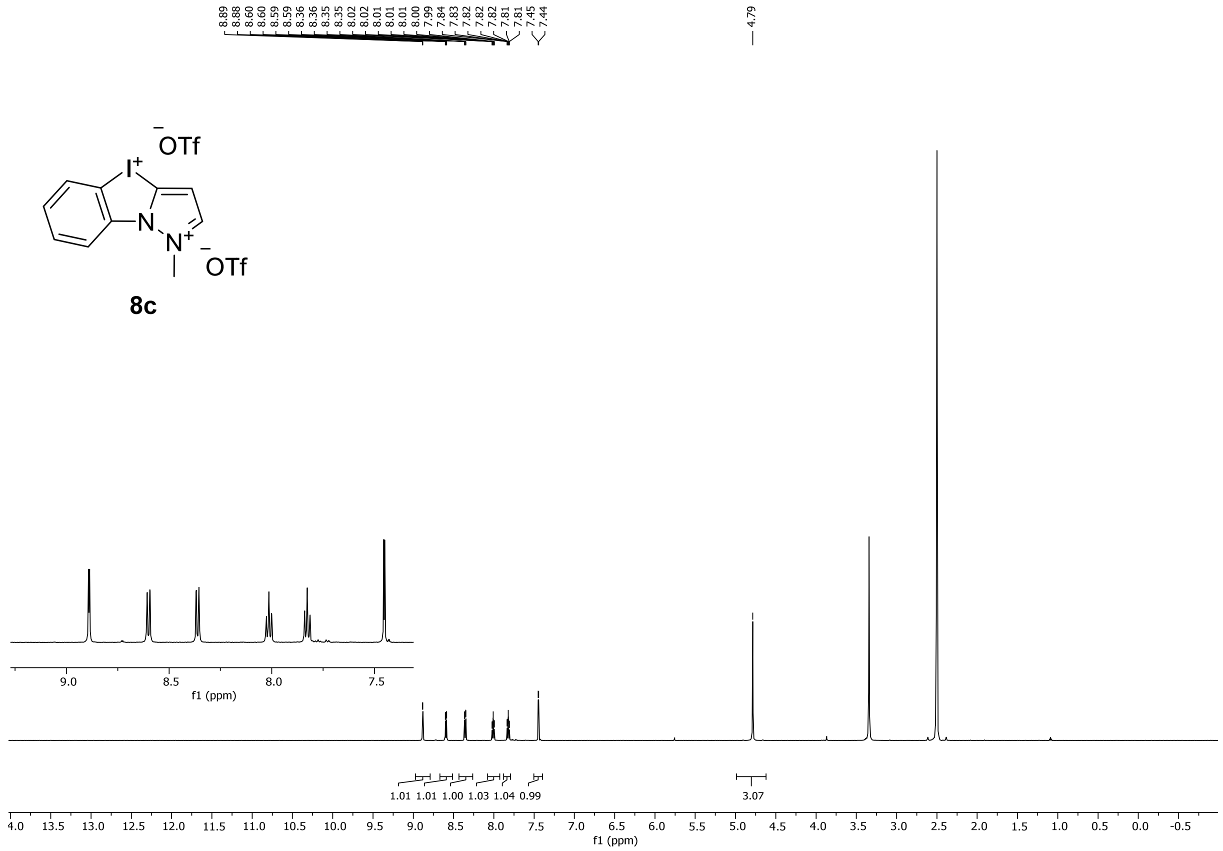
8.89 8.88 8.60 8.60 8.59 8.59 8.36 8.36 8.35 8.35 8.02 8.02 8.01 8.01 8.01 8.00 7.99 7.84 7.83 7.82 7.82 7.82 7.81 7.81 7.45 7.44
4.79
OTf
I+
N
N+
OTf
8c
f1 (ppm)
1.01 1.01 1.00 1.03 1.04 0.99
3.07
f1 (ppm)

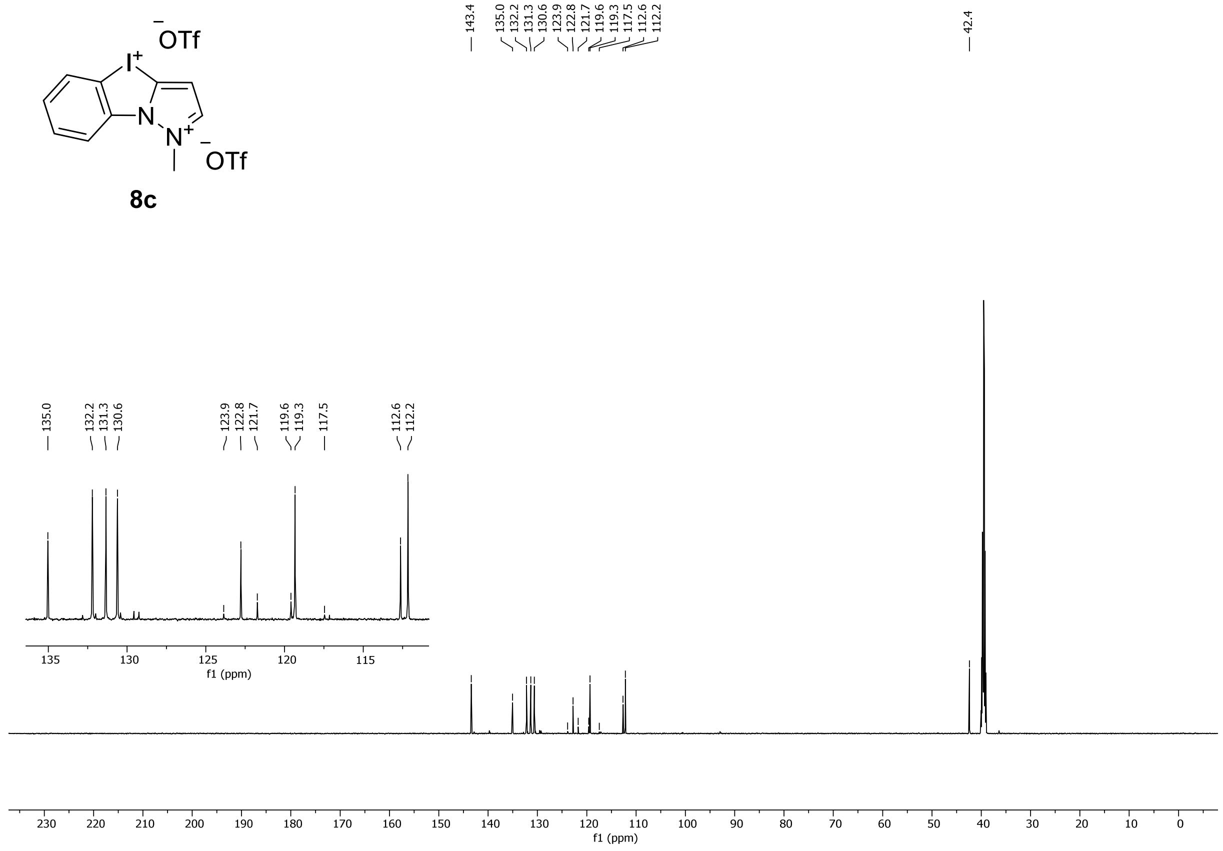
143.4
135.0 132.2 131.3 130.6 123.9 122.8 121.7 119.6 119.3 117.5 112.6 112.2
42.4
OTf
I+
N
N+
OTf
8c
135.0 132.2 131.3 130.6 123.9 122.8 121.7 119.6 119.3 117.5 112.6 112.2
f1 (ppm)
f1 (ppm)

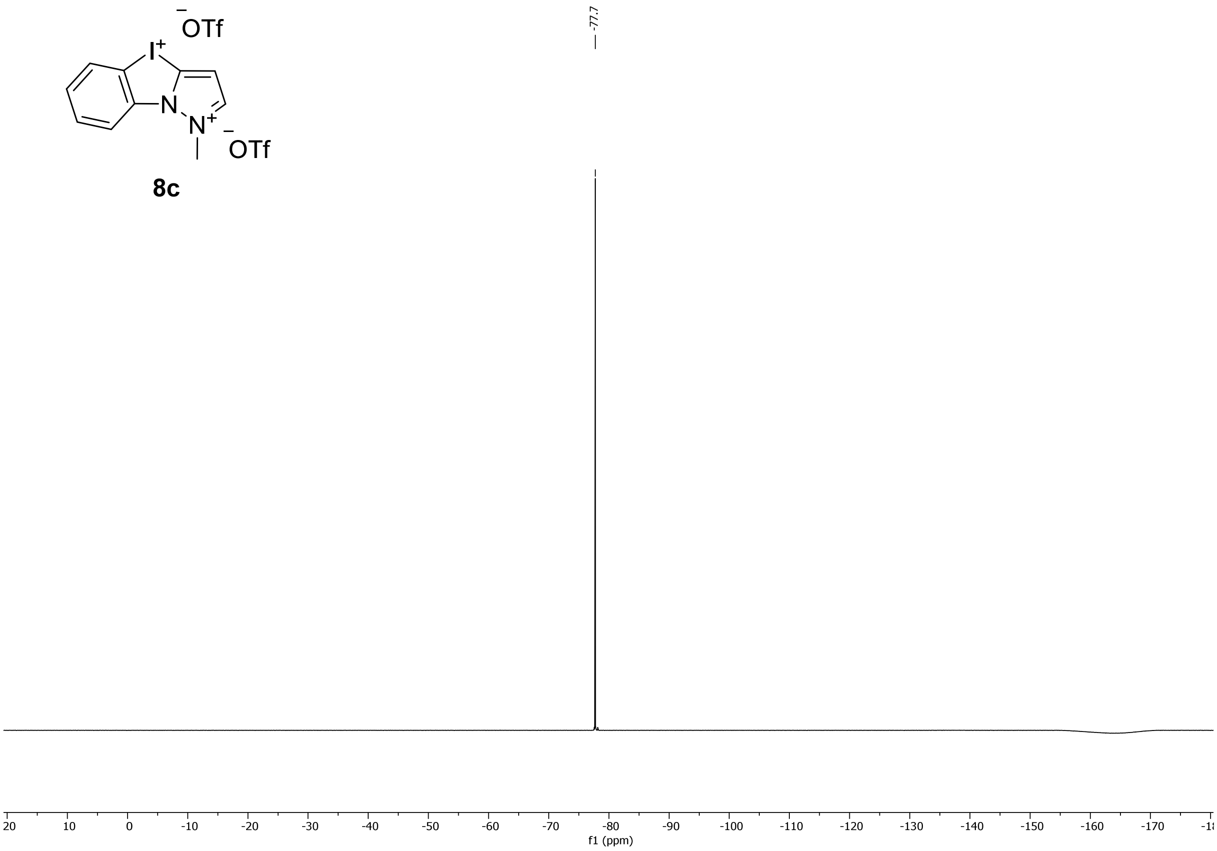

*Figure S39: 600 MHz ^{1}H-, 151 MHz ^{13}C- and 565 MHz ^{19}F-NMR spectra of 1-methylbenzo[d]pyrazolo[5,1-b][1,3]iodazole-1,4-diium bistriflate (**8c**) in d_6-DMSO.*

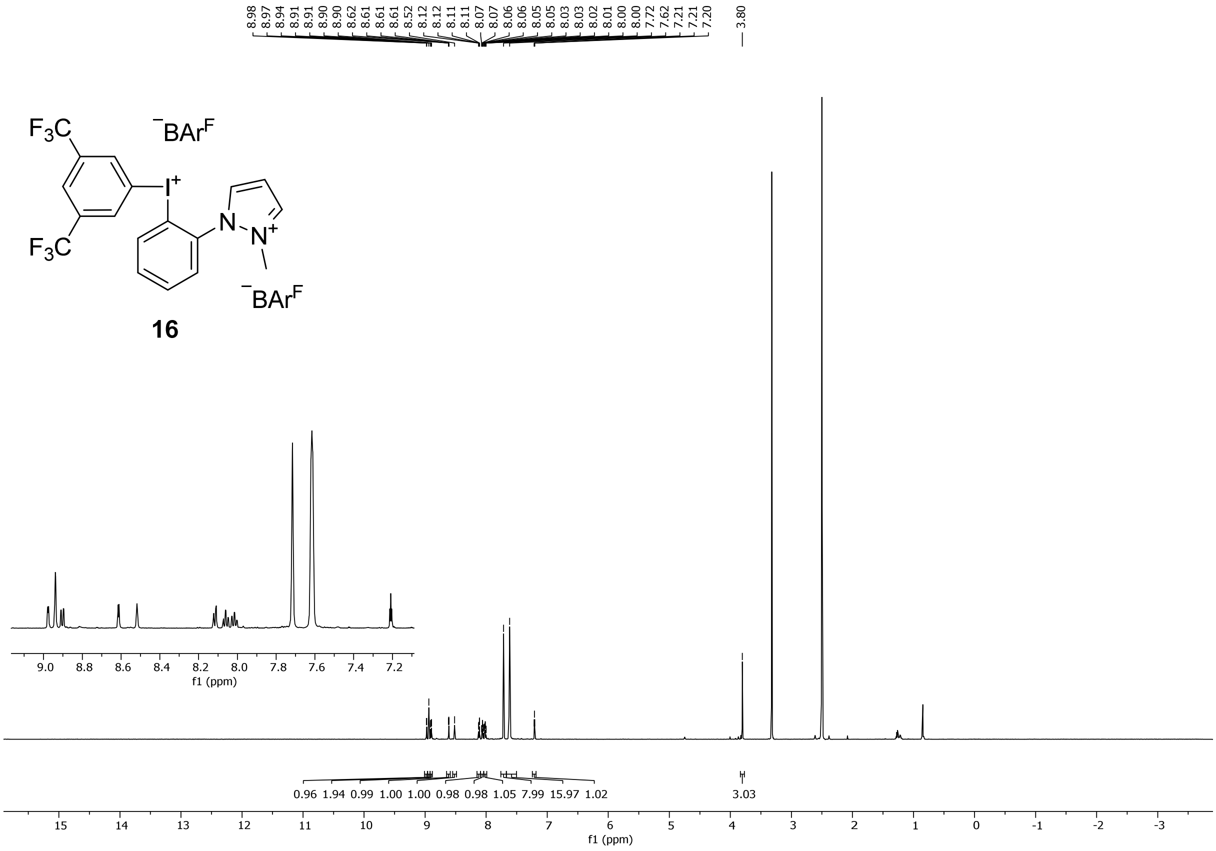

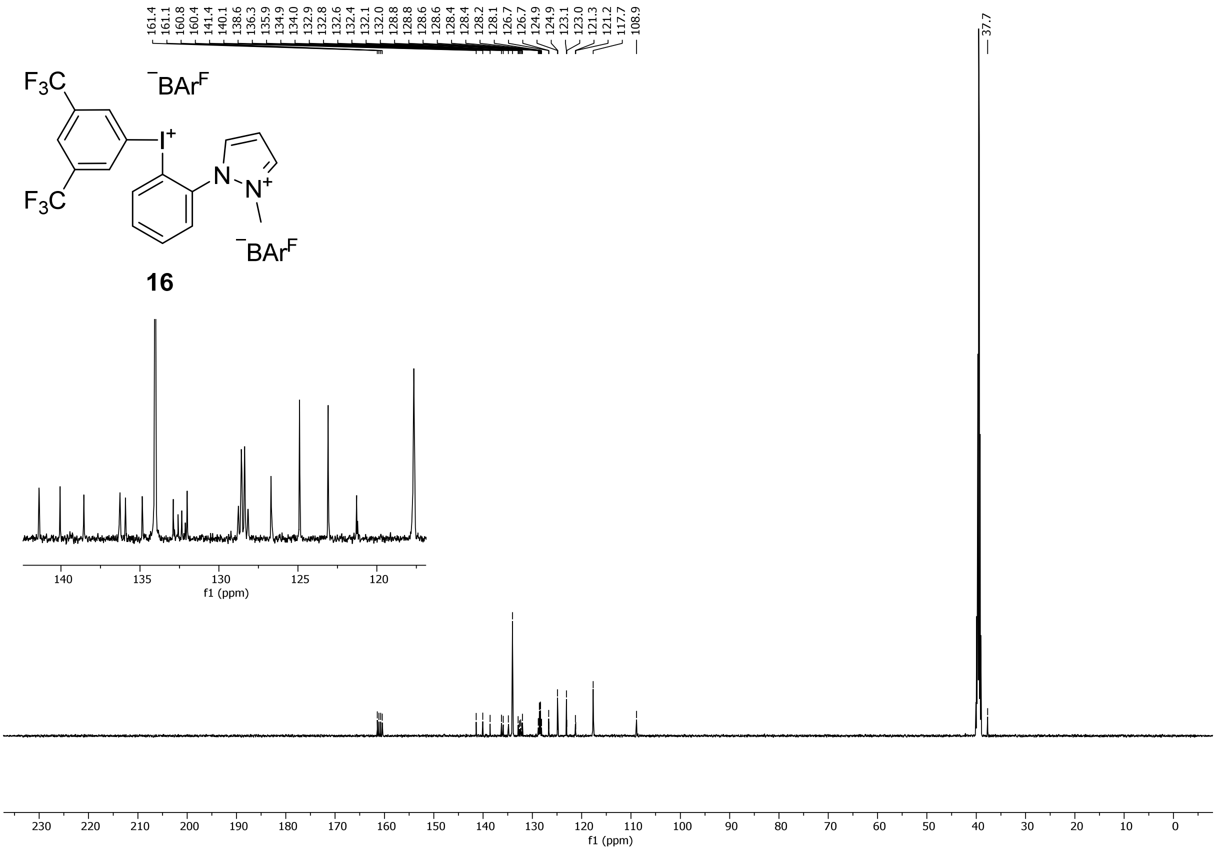

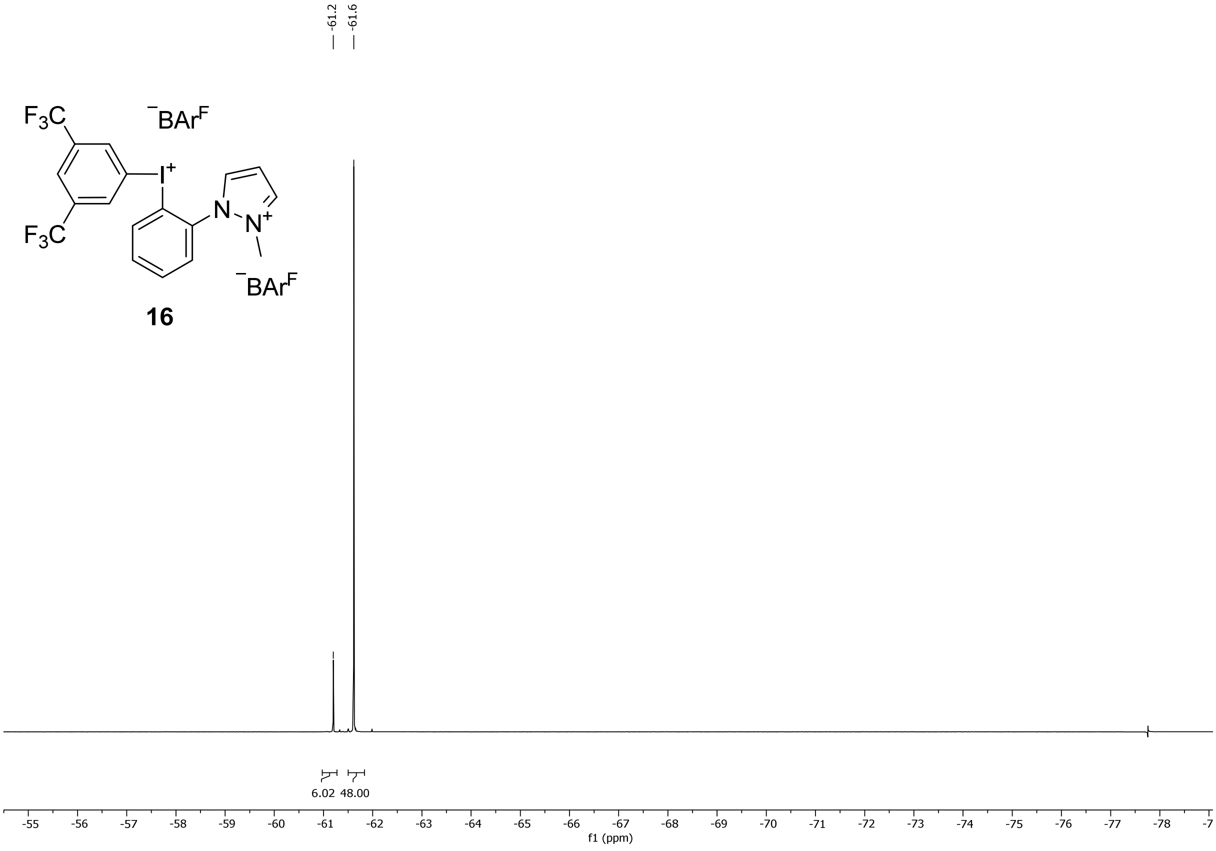

*Figure S40: 600 MHz ^{1}H-, 151 MHz ^{13}C- and 565 MHz ^{19}F-NMR spectra of 1-(2-((3,5-bis(trifluoromethyl)phenyl)iodonio)phenyl)-2-methyl-1H-pyrazol-2-ium bis(tetrakis(3,5-bis(trifluoromethyl)phenyl)borate (**16**) in d_6-DMSO.*

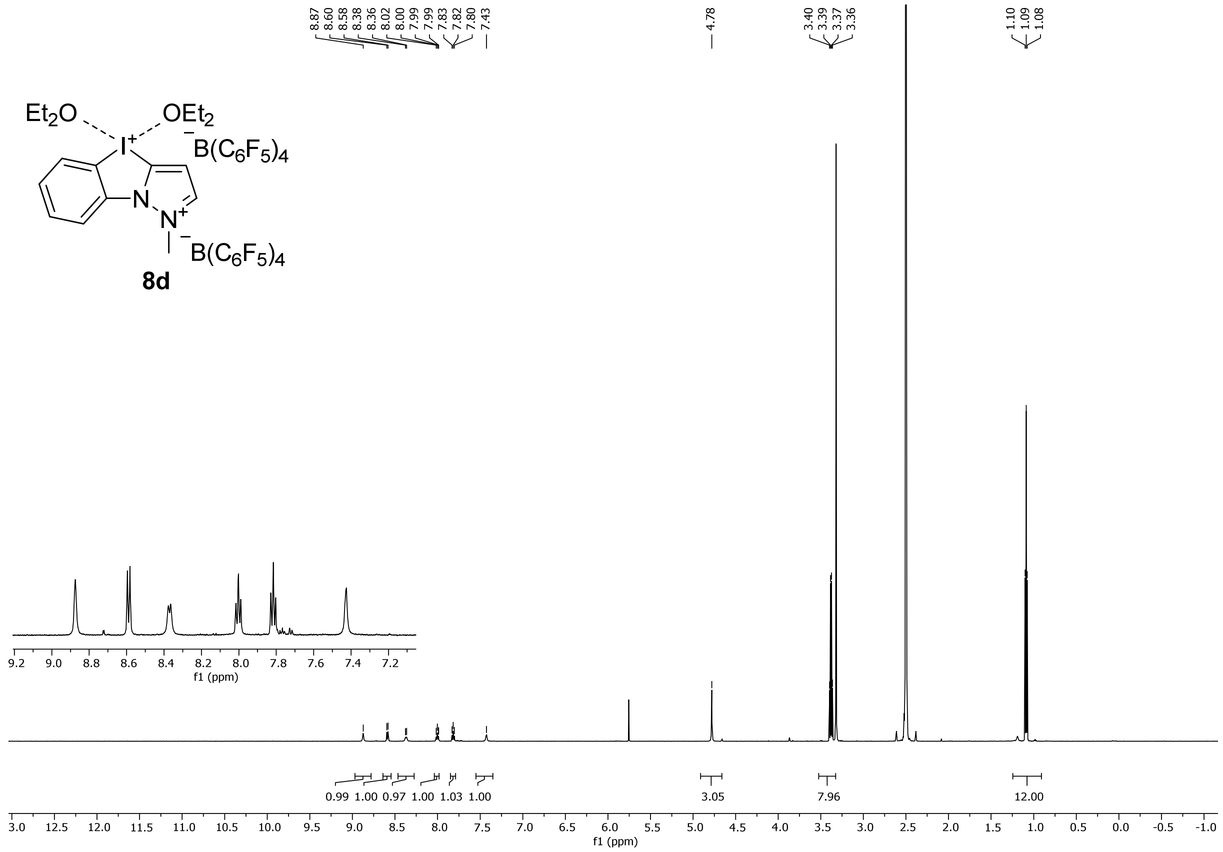
8.87 8.60 8.58 8.38 8.36 8.02 8.00 7.99 7.99 7.83 7.82 7.80 7.43
4.78
3.40 3.39 3.37 3.36
1.10 1.09 1.08
Et2O OEt2 I+ −B(C6F5)4 N N+ −B(C6F5)4
8d
0.99 1.00 0.97 1.00 1.03 1.00 3.05 7.96 12.00
f1 (ppm)

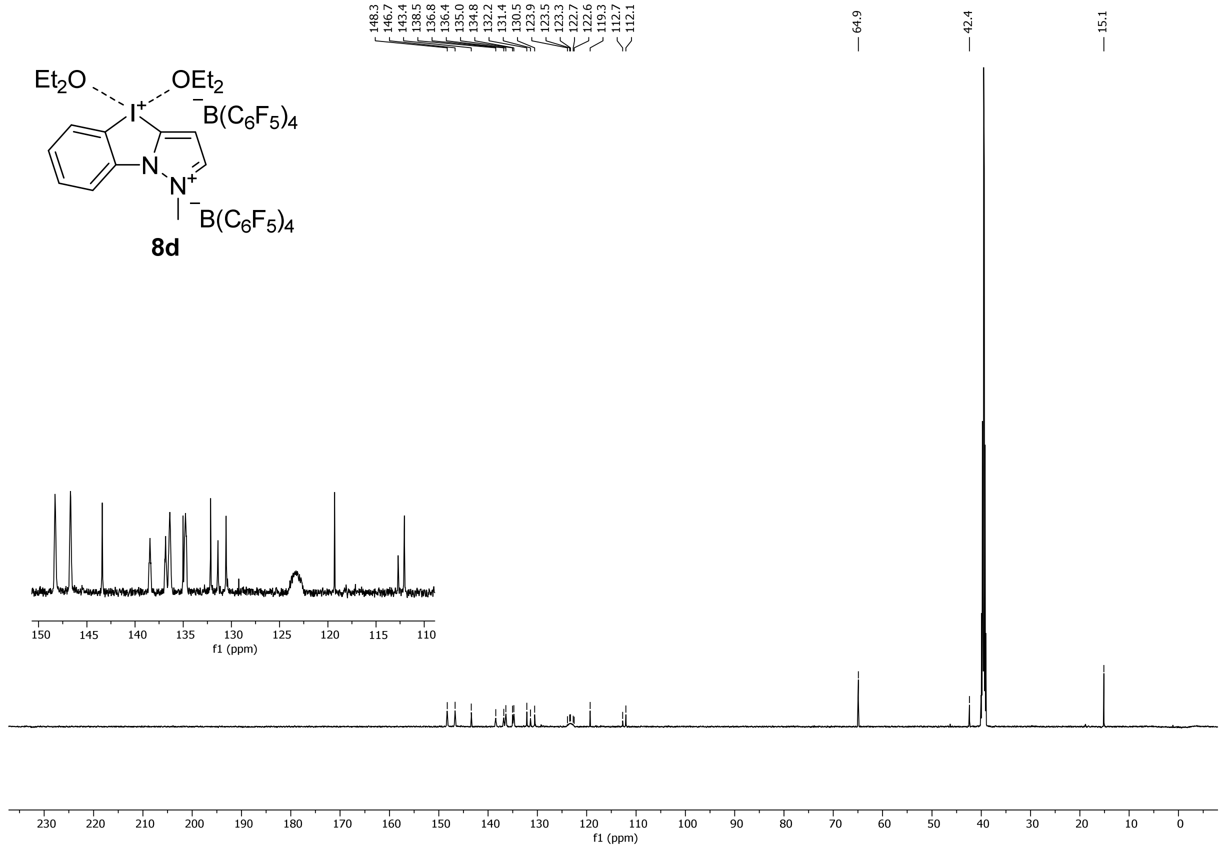
148.3 146.7 143.4 138.5 136.8 136.4 135.0 134.8 132.2 131.4 130.5 123.9 123.5 123.3 122.7 122.6 119.3 112.7 112.1
64.9
42.4
15.1
Et2O OEt2 I+ −B(C6F5)4 N N+ −B(C6F5)4
8d
f1 (ppm)

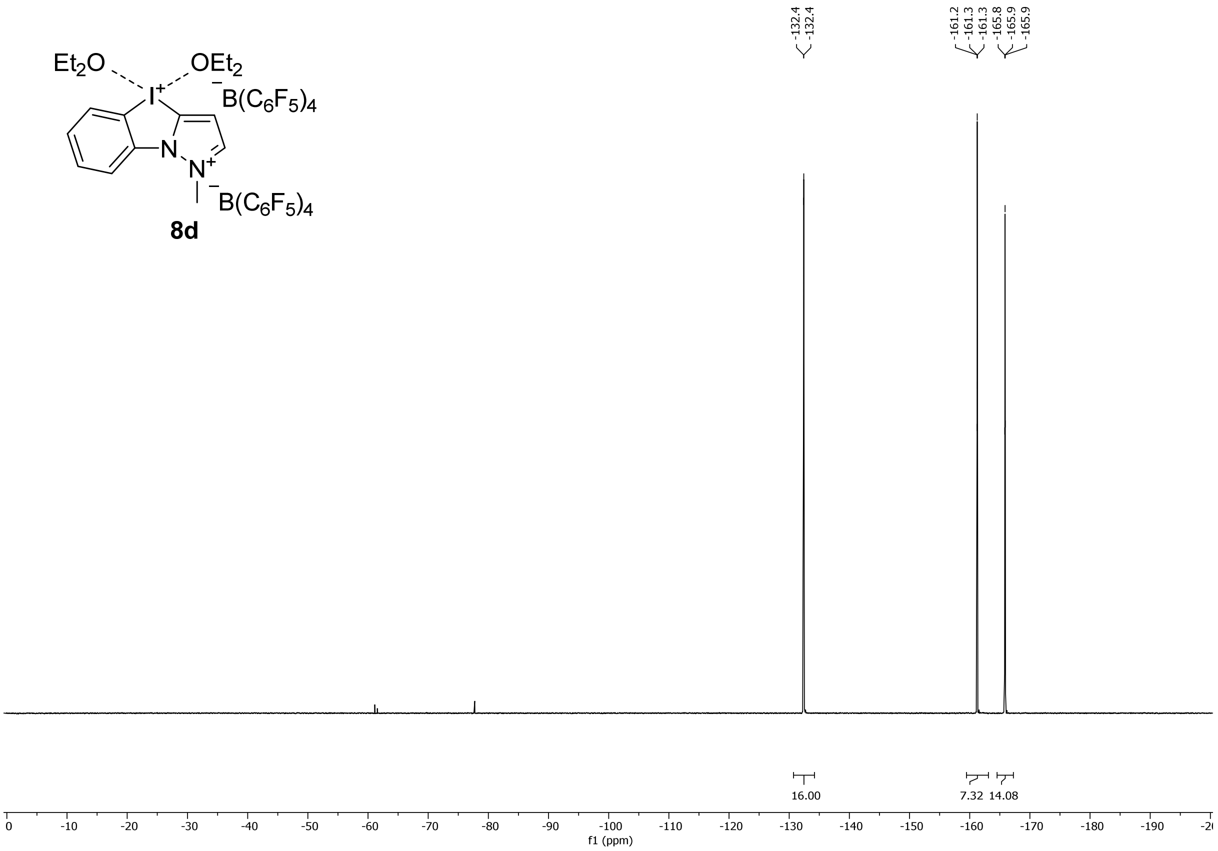

*Figure S41: 600 MHz ^{1}H-, 151 MHz ^{13}C- and 565 MHz ^{19}F-NMR spectra of 1-methylbenzo[d]pyrazolo[5,1-b][1,3]iodazole-1,4-diium bis(tetrakis(pentafluorophenyl)borate dietherate complex (**8d**) in d$_6$-DMSO.*

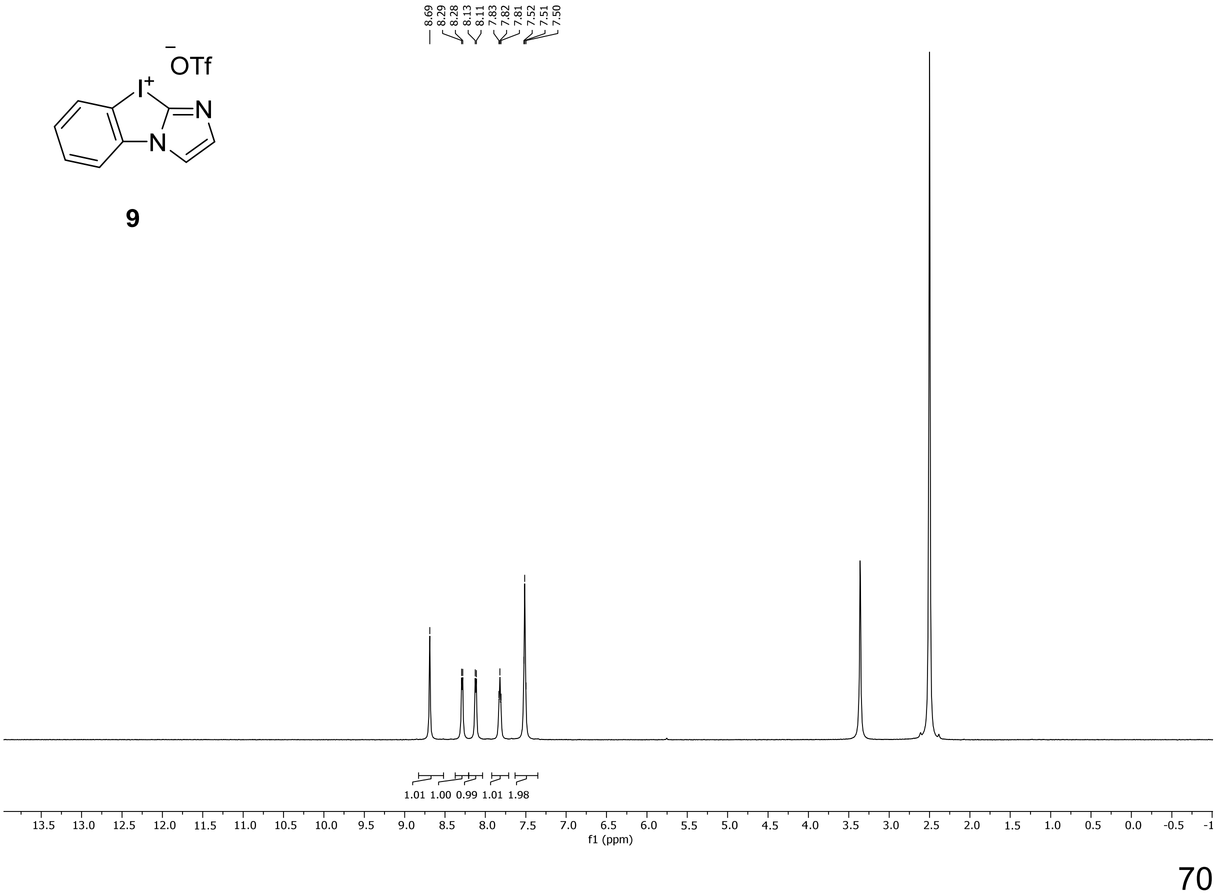

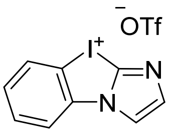
OTf
I+
N
N

9

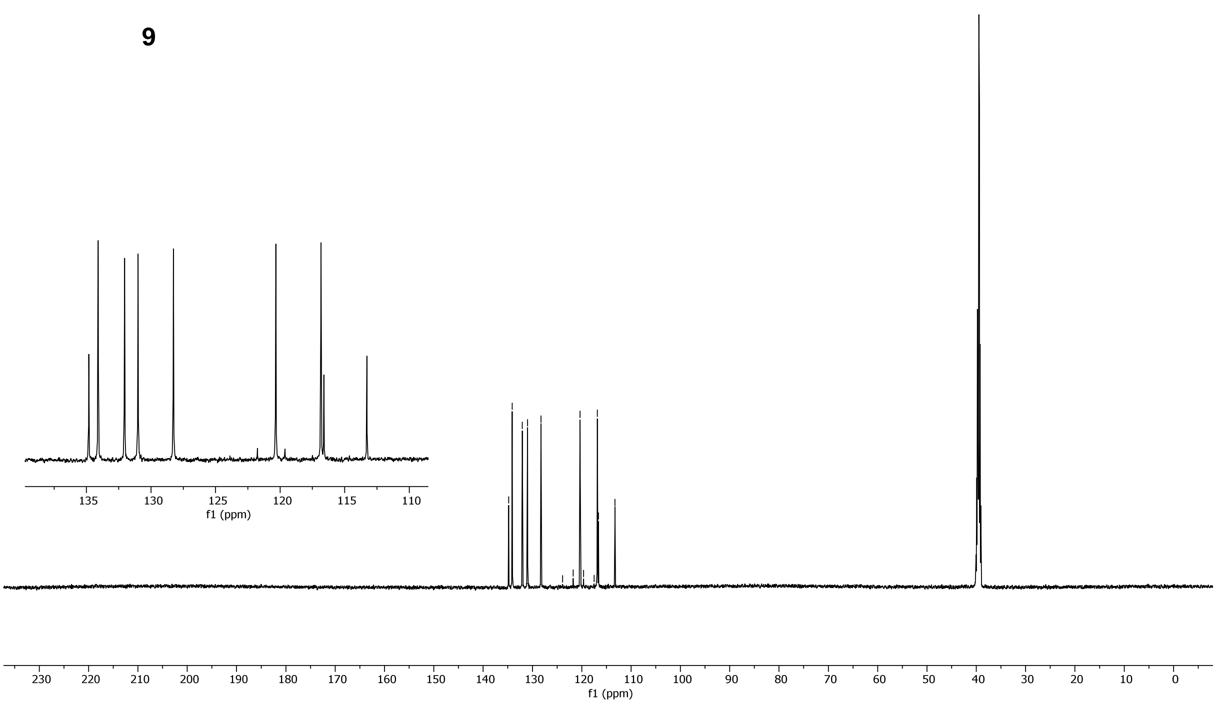
134.8
134.1
132.0
131.0
128.3
123.9
121.8
120.3
119.6
117.5
116.8
116.6
113.3
f1 (ppm)
f1 (ppm)

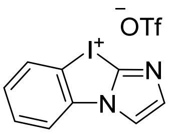
OTf
I+
N
N

9

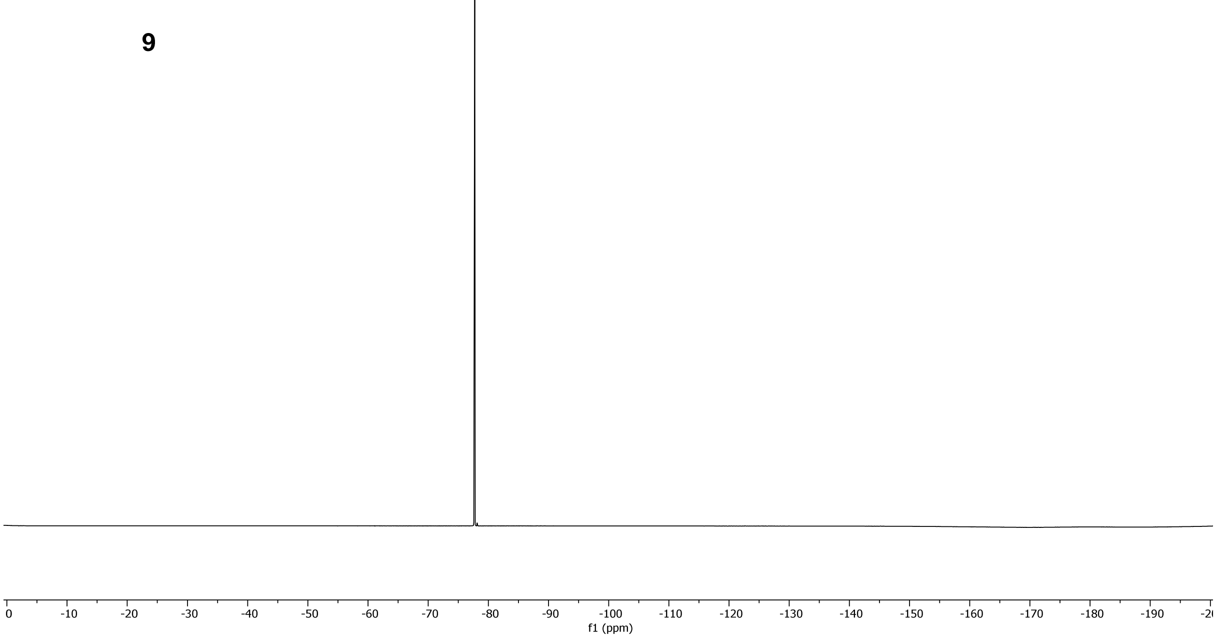
-77.7
f1 (ppm)

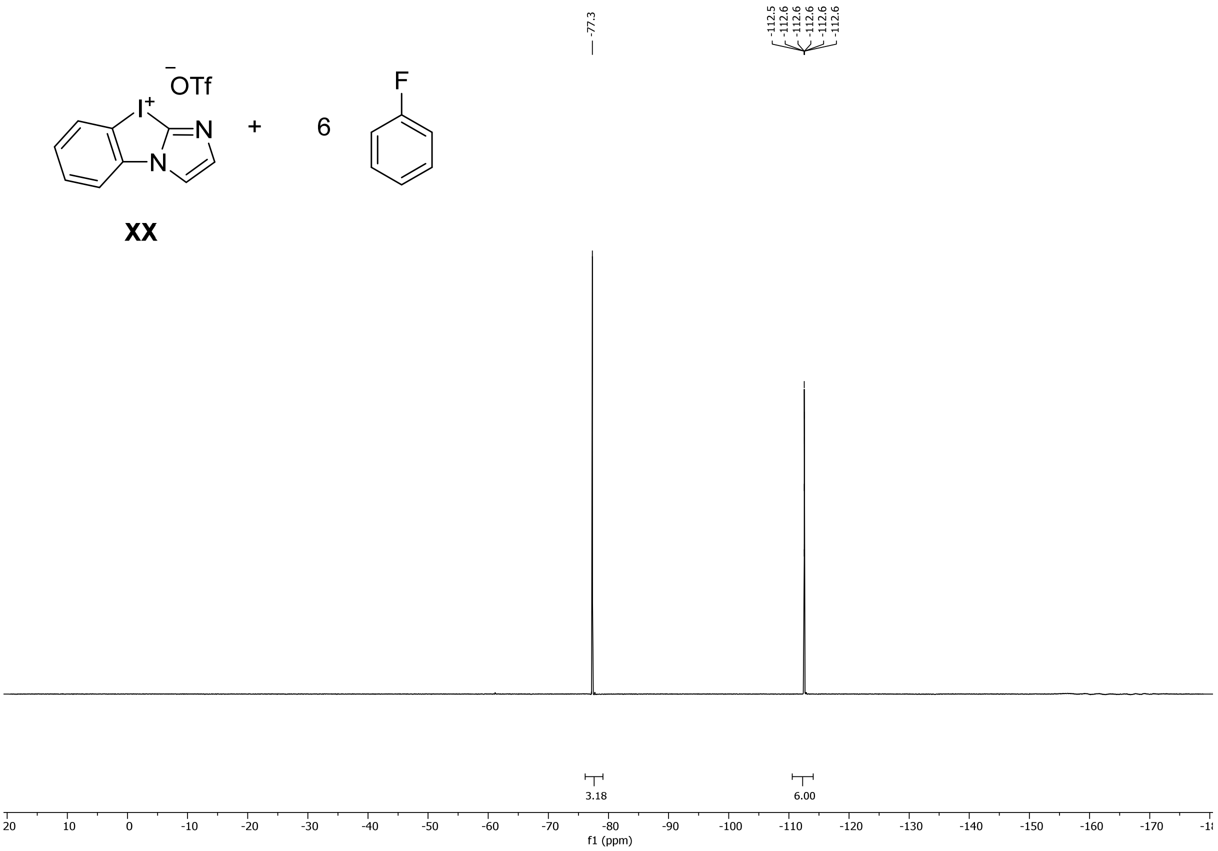

*Figure S42: 600 MHz ^{1}H-, 151 MHz ^{13}C- and 565 MHz ^{19}F-NMR (with and without 6 equiv. PhF as internal standard) spectra of benzo[d]imidazo[2,1-b][1,3]iodazol-9-ium triflate (**9**) in d_6-DMSO.*

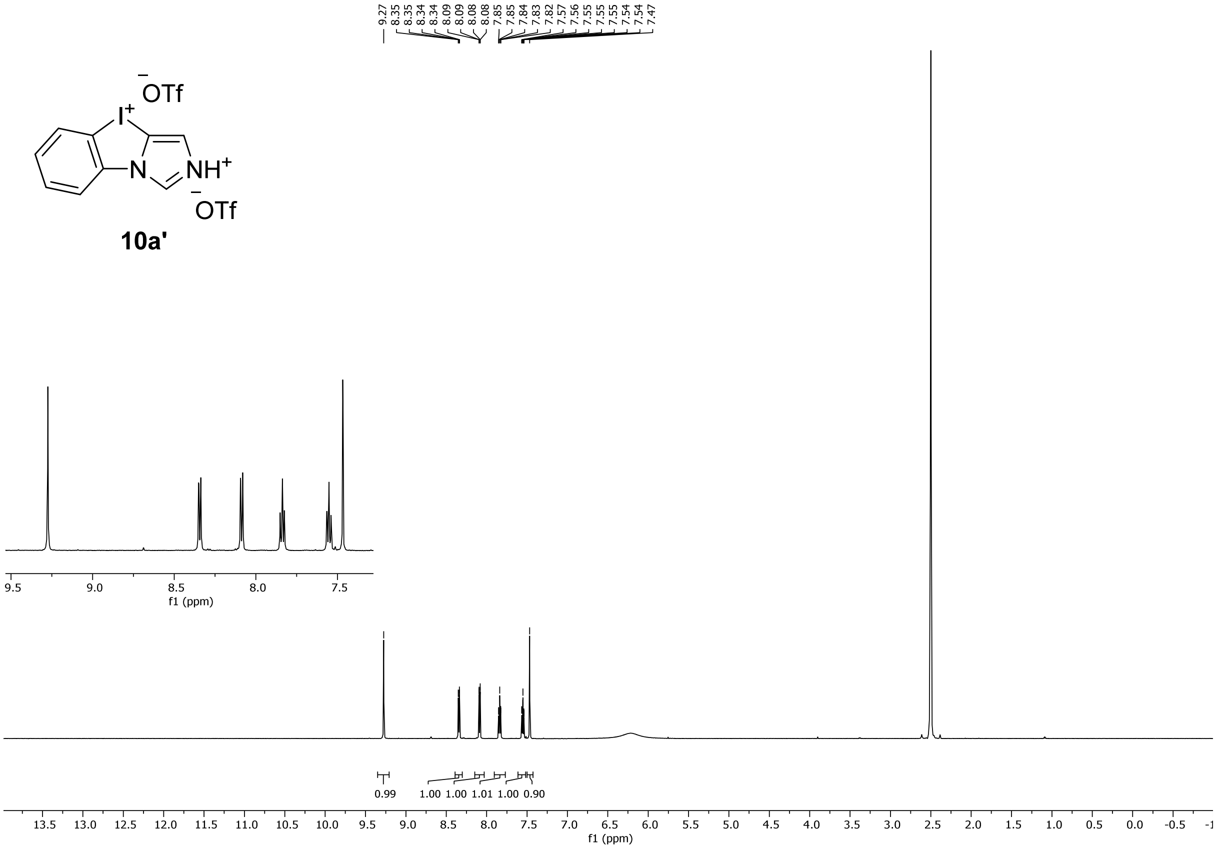

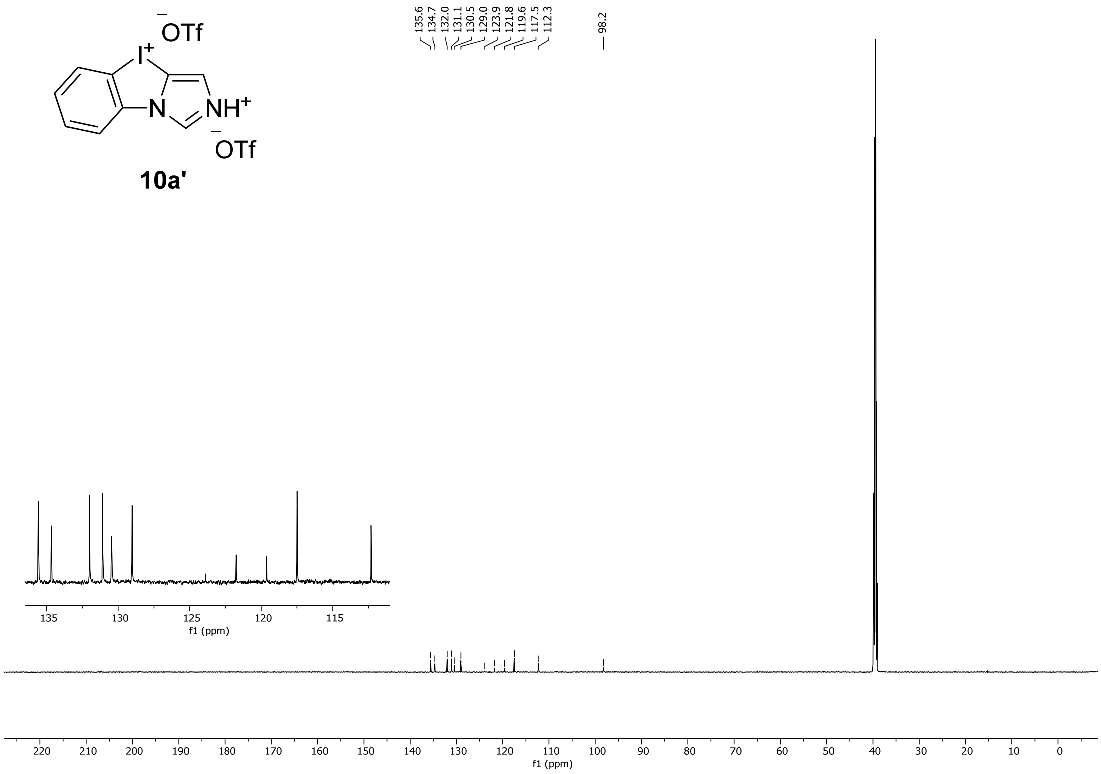

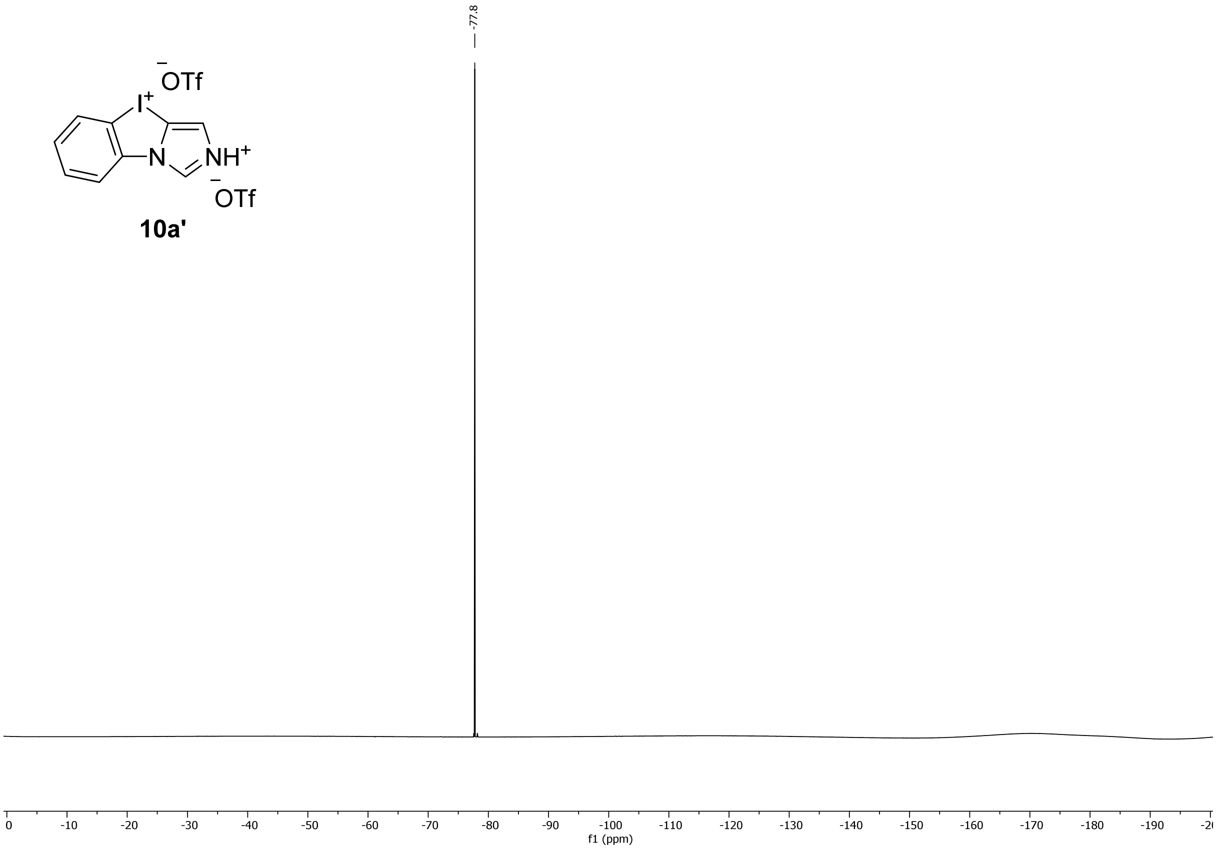

*Figure S43: 600 MHz ^{1}H-, 151 MHz ^{13}C- and 565 MHz ^{19}F-NMR spectra of benzo[d]imidazo[5,1-b][1,3]iodazol-4-diium bistriflate (**10a'**) in d_6-DMSO.*

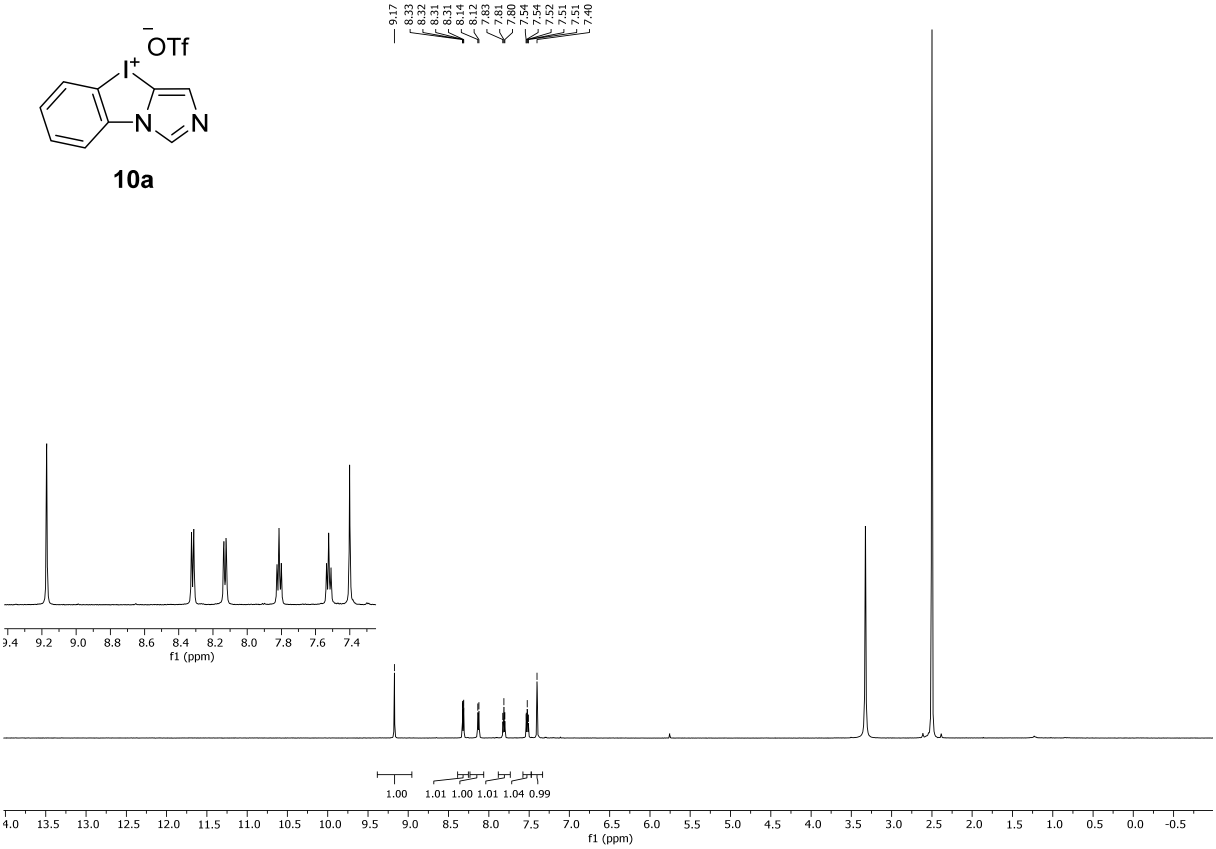
9.17
8.33
8.32
8.31
8.31
8.14
8.12
7.83
7.81
7.80
7.54
7.54
7.52
7.51
7.51
7.40
OTf
I+
N
N
10a
1.00
1.01
1.00
1.01
1.04
0.99
f1 (ppm)

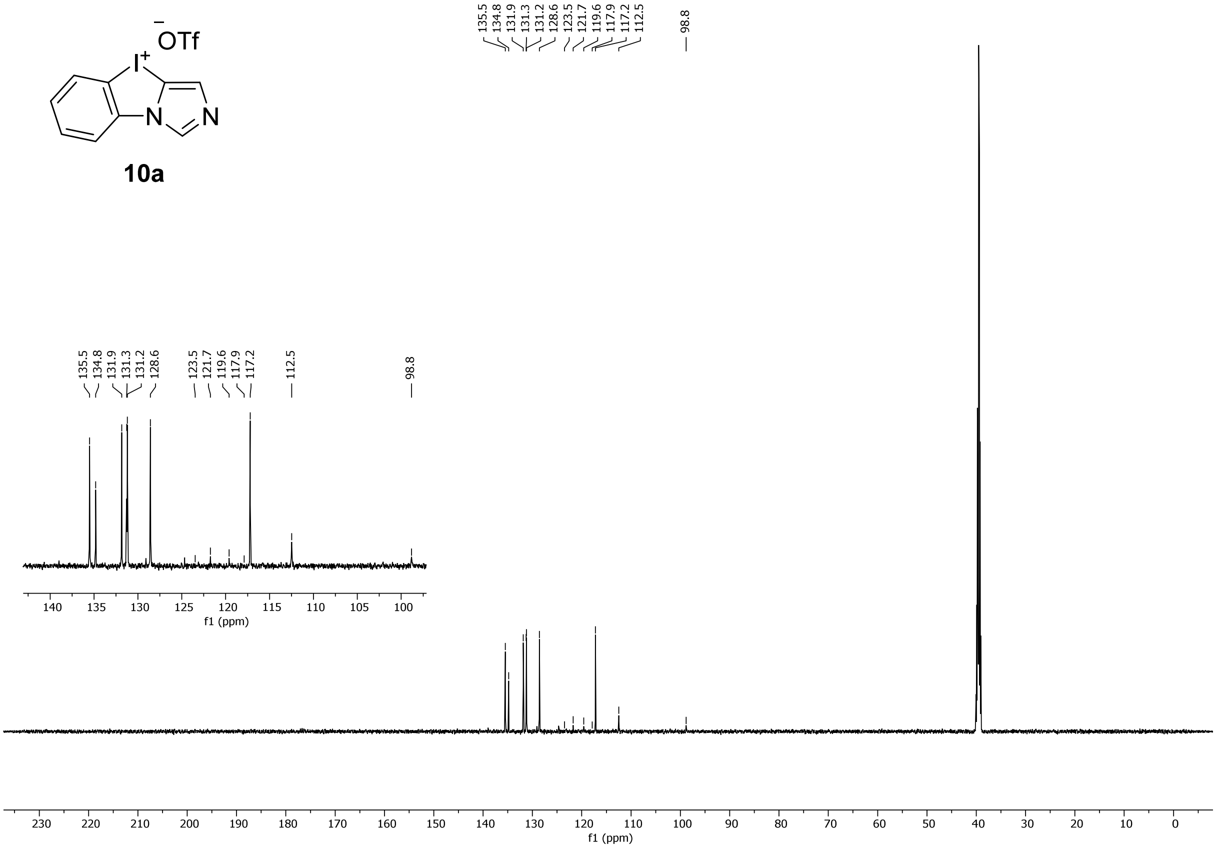
135.5
134.8
131.9
131.3
131.2
128.6
123.5
121.7
119.6
117.9
117.2
112.5
98.8
OTf
I+
N
N
10a
f1 (ppm)

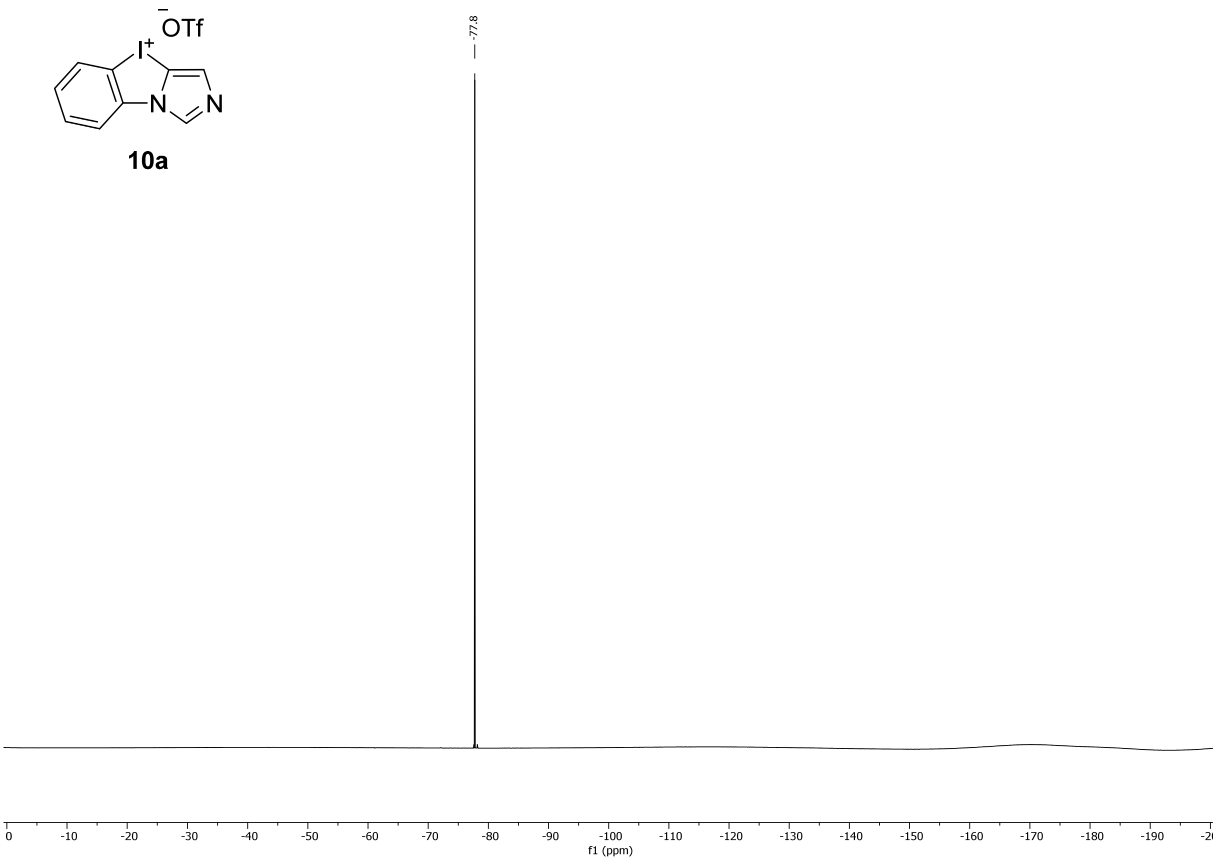

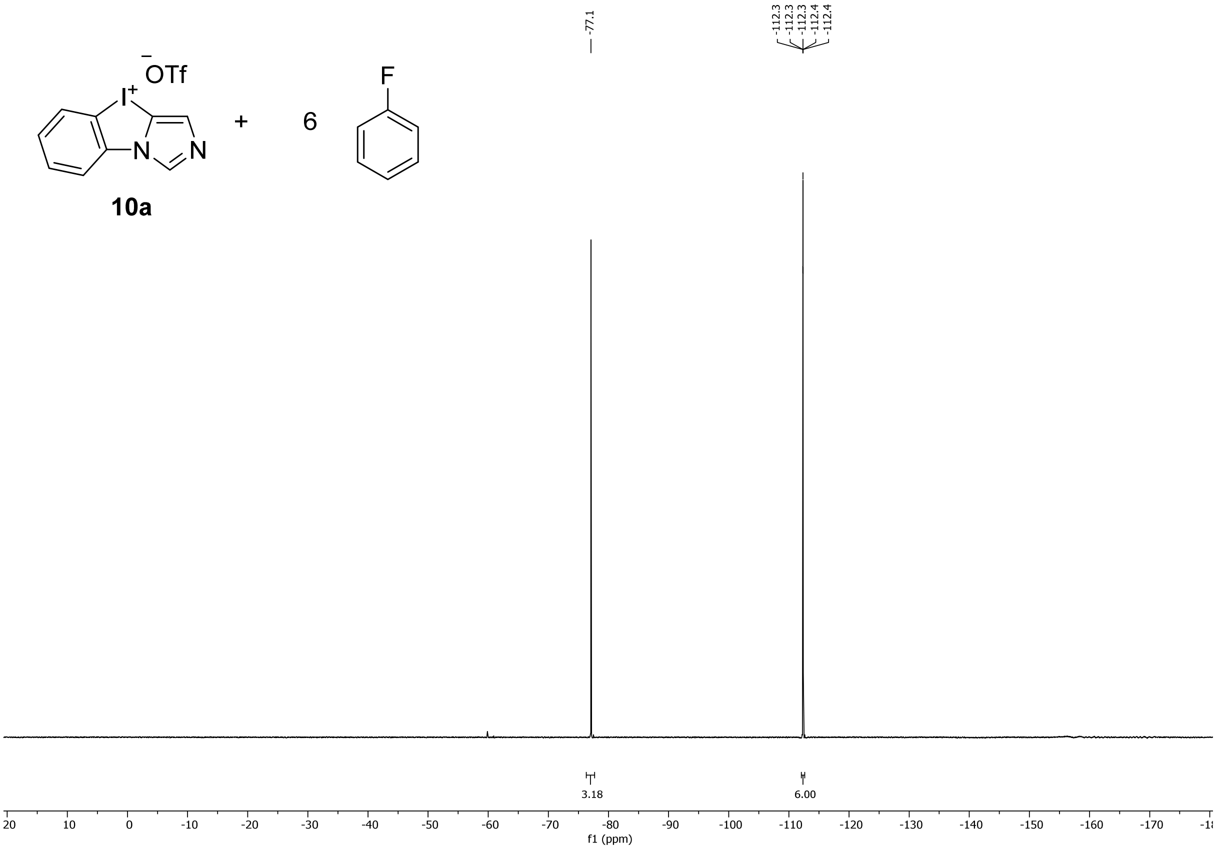

*Figure S44: 600 MHz ^{1}H-, 151 MHz ^{13}C- and 565 MHz ^{19}F-NMR (with and without 6 equiv. PhF as internal standard) spectra of benzo[d]imidazo[5,1-b][1,3]iodazol-4-diium triflate (**10a**) in d_6-DMSO.*

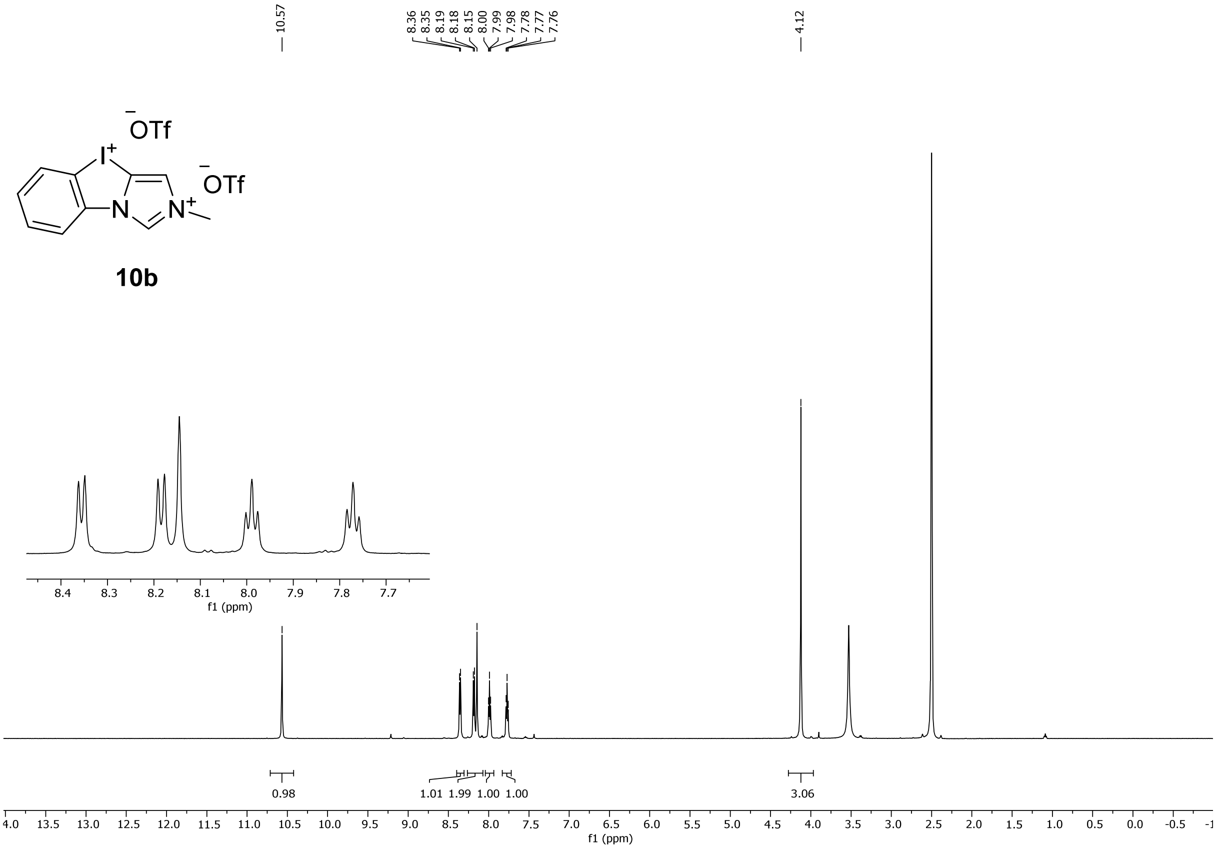

10.57
8.36
8.35
8.19
8.18
8.15
8.00
7.99
7.98
7.78
7.77
7.76
4.12
OTf
OTf
10b
0.98
1.01 1.99 1.00 1.00
3.06
f1 (ppm)

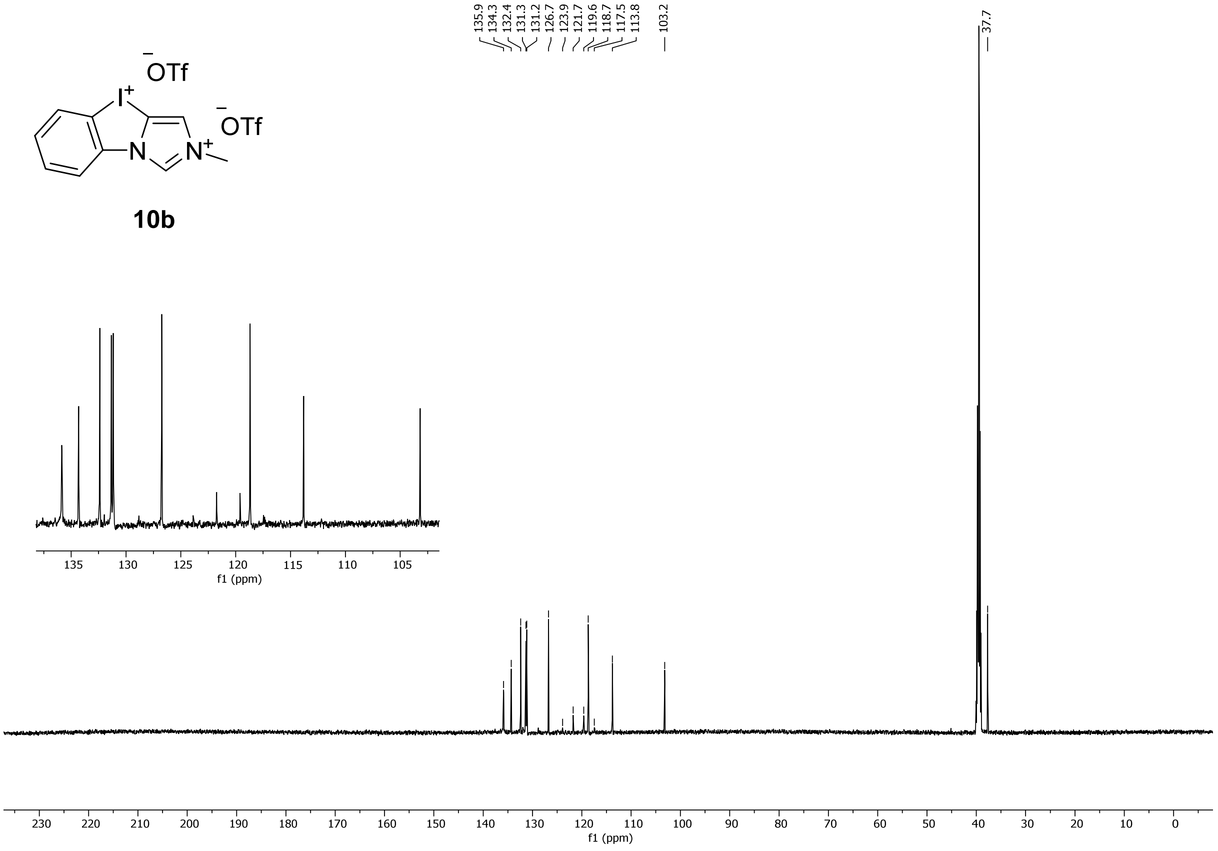

135.9
134.3
132.4
131.3
131.2
126.7
123.9
121.7
119.6
118.7
117.5
113.8
103.2
37.7
OTf
OTf
10b
f1 (ppm)

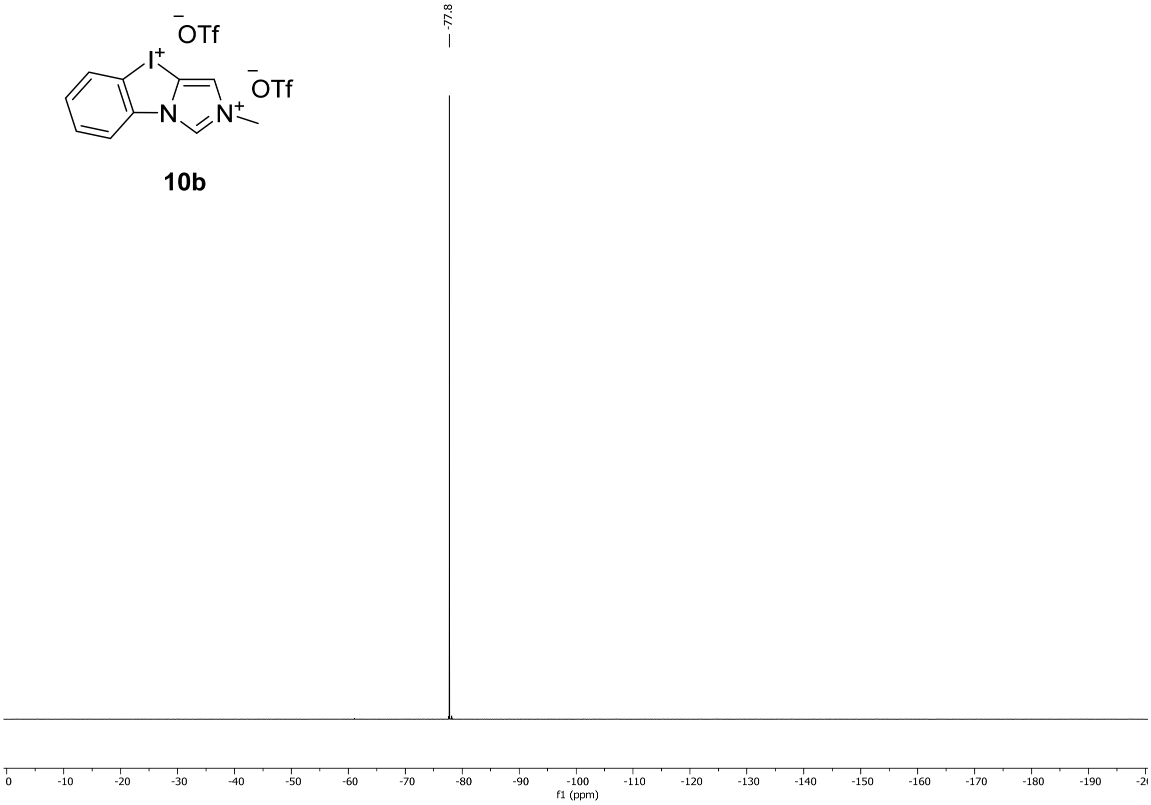

*Figure S45: 600 MHz ^{1}H-, 151 MHz ^{13}C- and 565 MHz ^{19}F-NMR spectra of 2-methylbenzo[d]imidazo[5,1-b][1,3]iodazole-2,4-diium bistriflate (**10b**) in d$_6$-DMSO.*

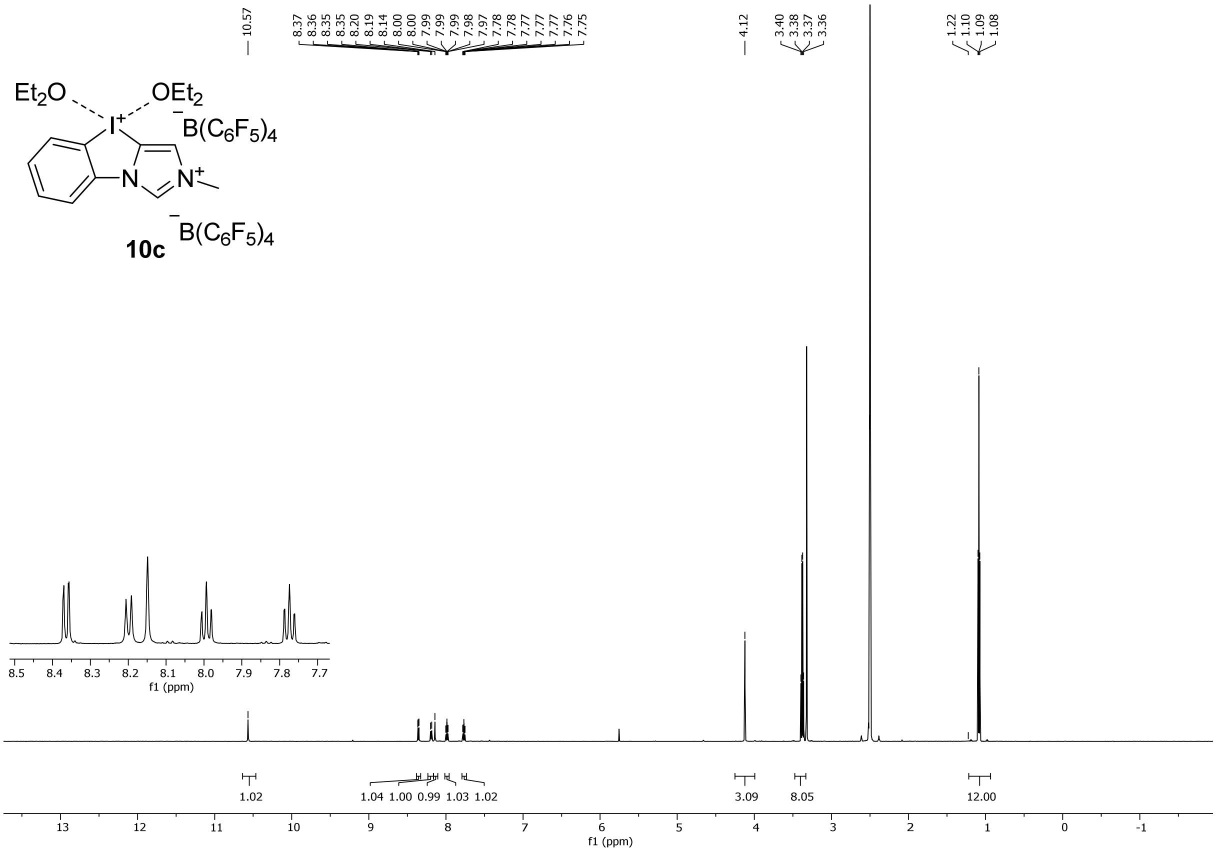

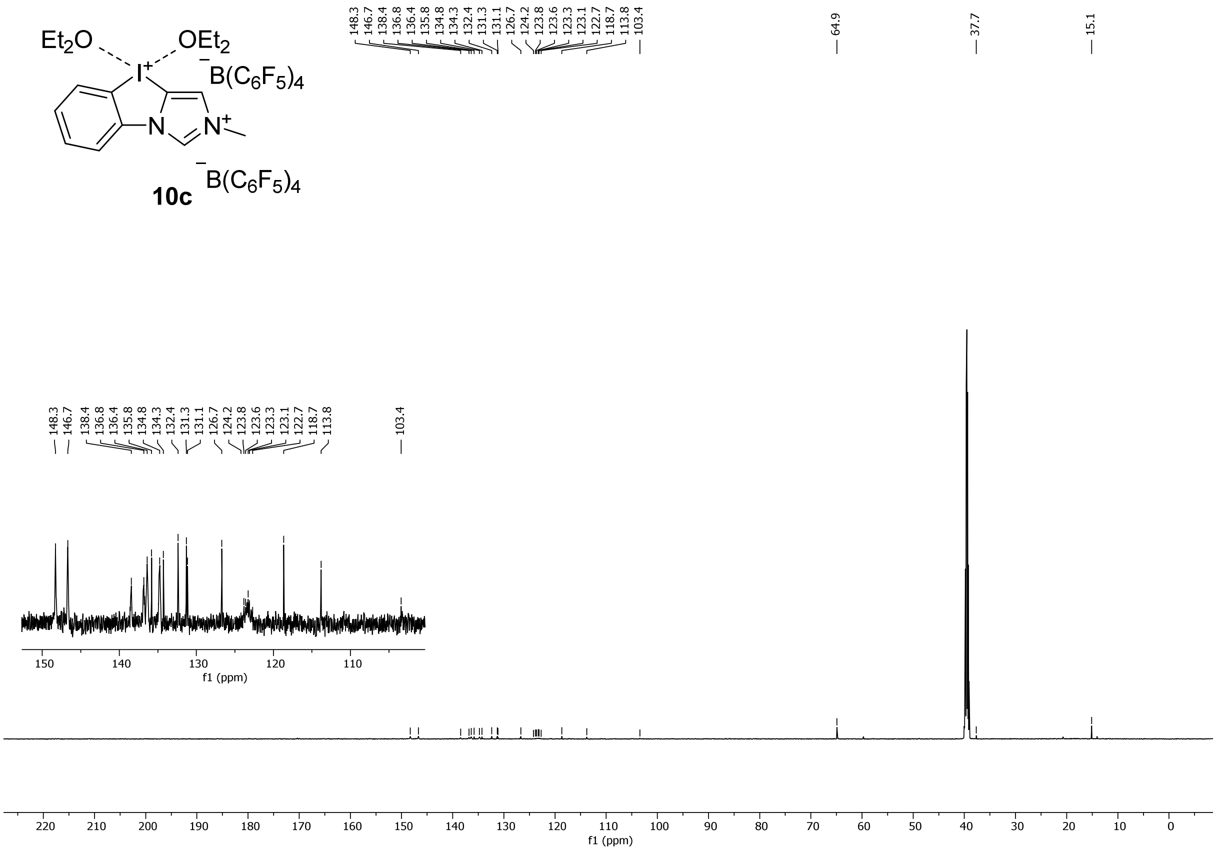

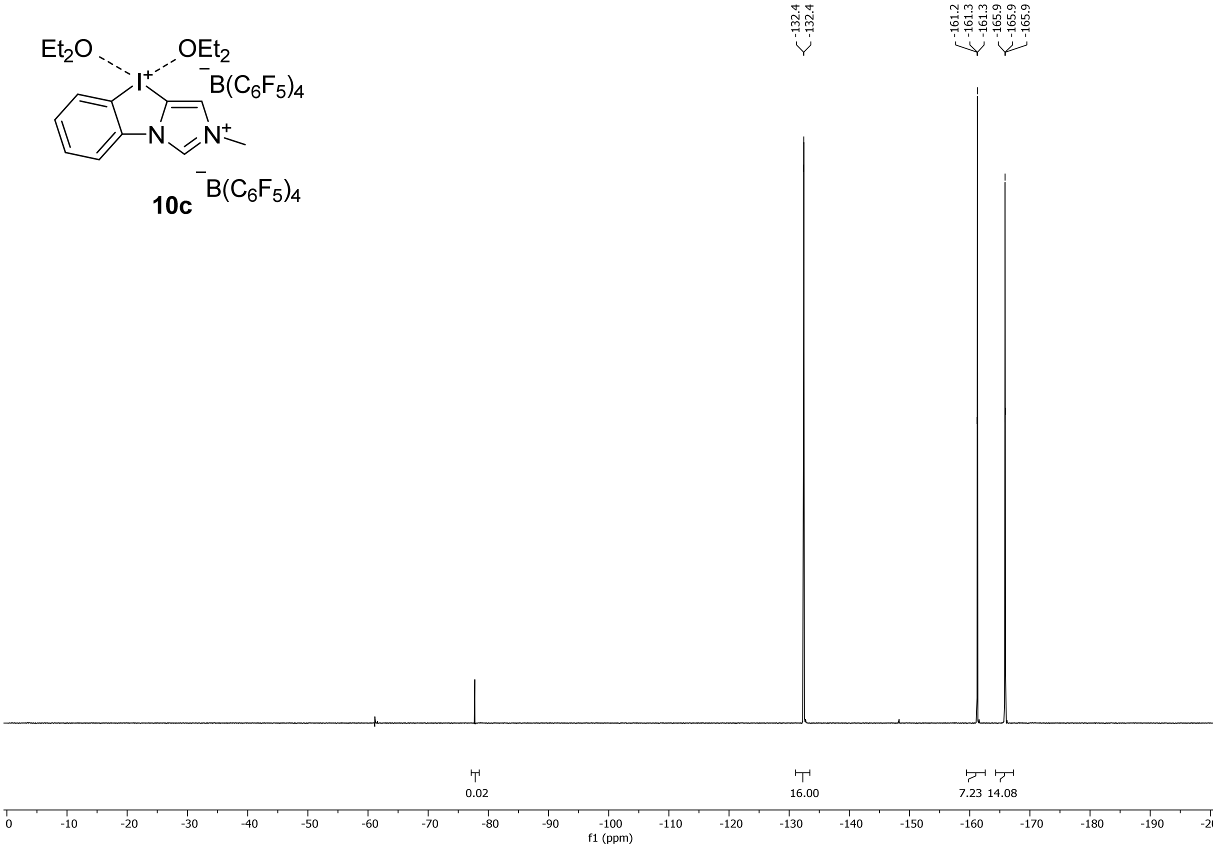

*Figure S46: 600 MHz ^{1}H-, 151 MHz ^{13}C- and 565 MHz ^{19}F-NMR spectra of 2-methylbenzo[d]imidazo[5,1-b][1,3]iodazole-2,4-diium bis(tetrakis(pentafluorophenyl)borate (**10c**) in d_6-DMSO.*

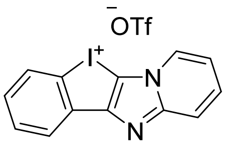

OTf
I+
N
N

11a

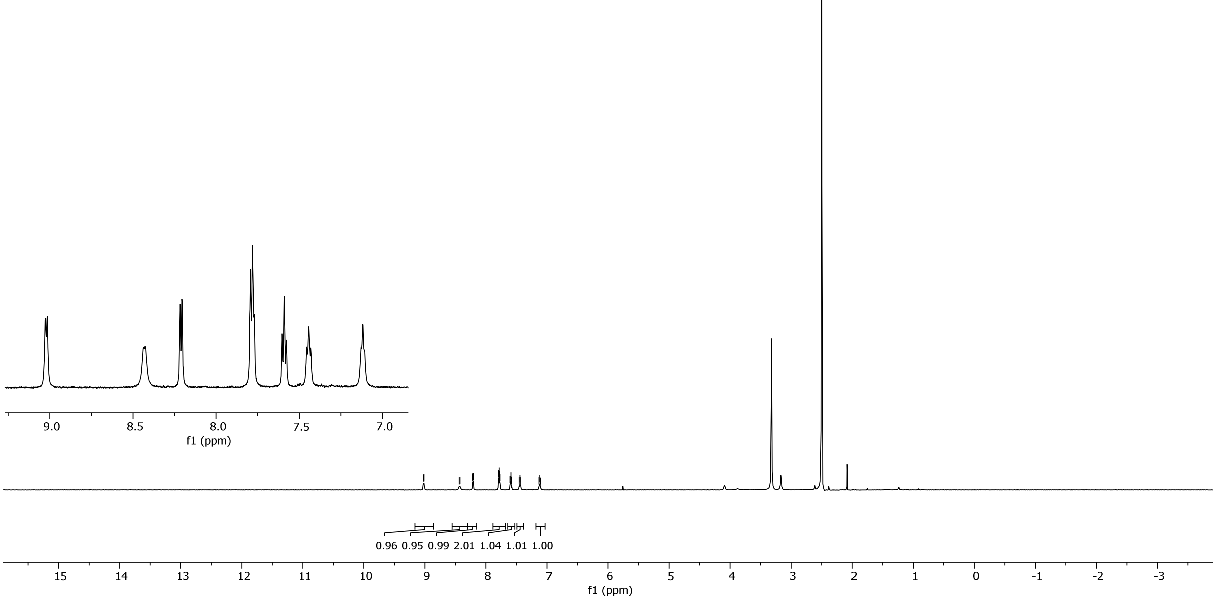

9.03
9.02
8.44
8.43
8.22
8.22
8.21
8.20
7.79
7.78
7.78
7.77
7.60
7.59
7.58
7.58
7.46
7.44
7.43
7.13
7.12
7.11
0.96 0.95 0.99 2.01 1.04 1.01 1.00
f1 (ppm)

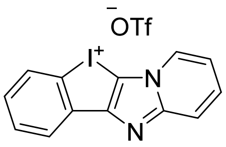

OTf
I+
N
N

11a

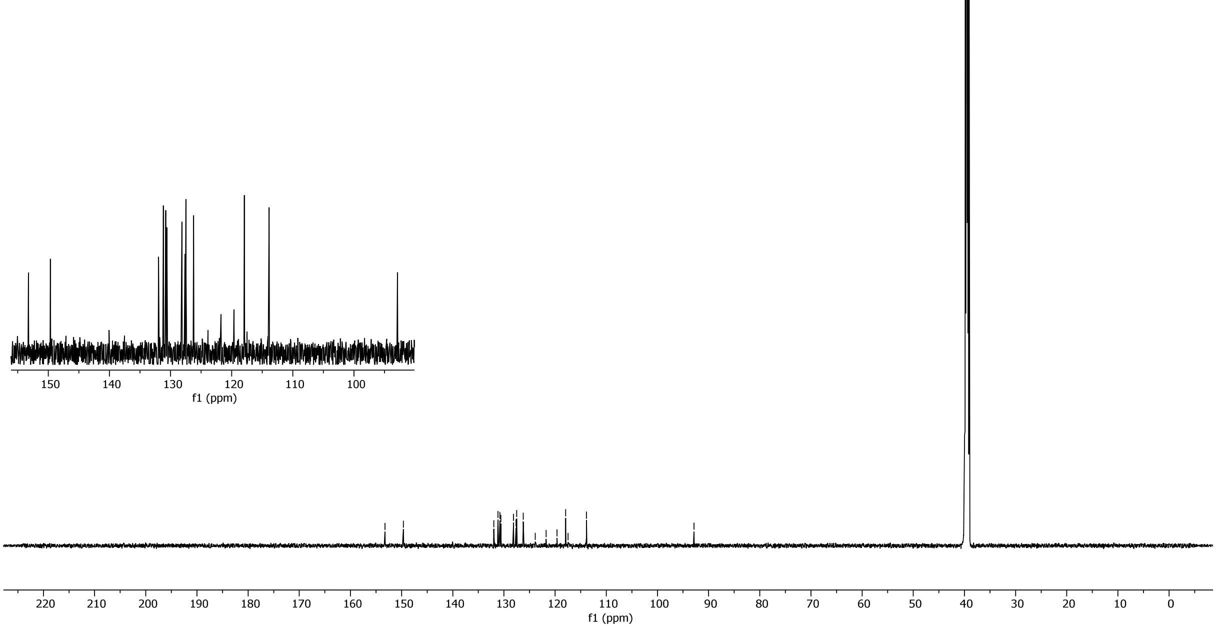

153.3
149.6
132.0
131.2
130.8
130.6
128.1
127.6
127.5
126.2
123.9
121.7
119.6
117.9
117.5
113.9
92.9
f1 (ppm)

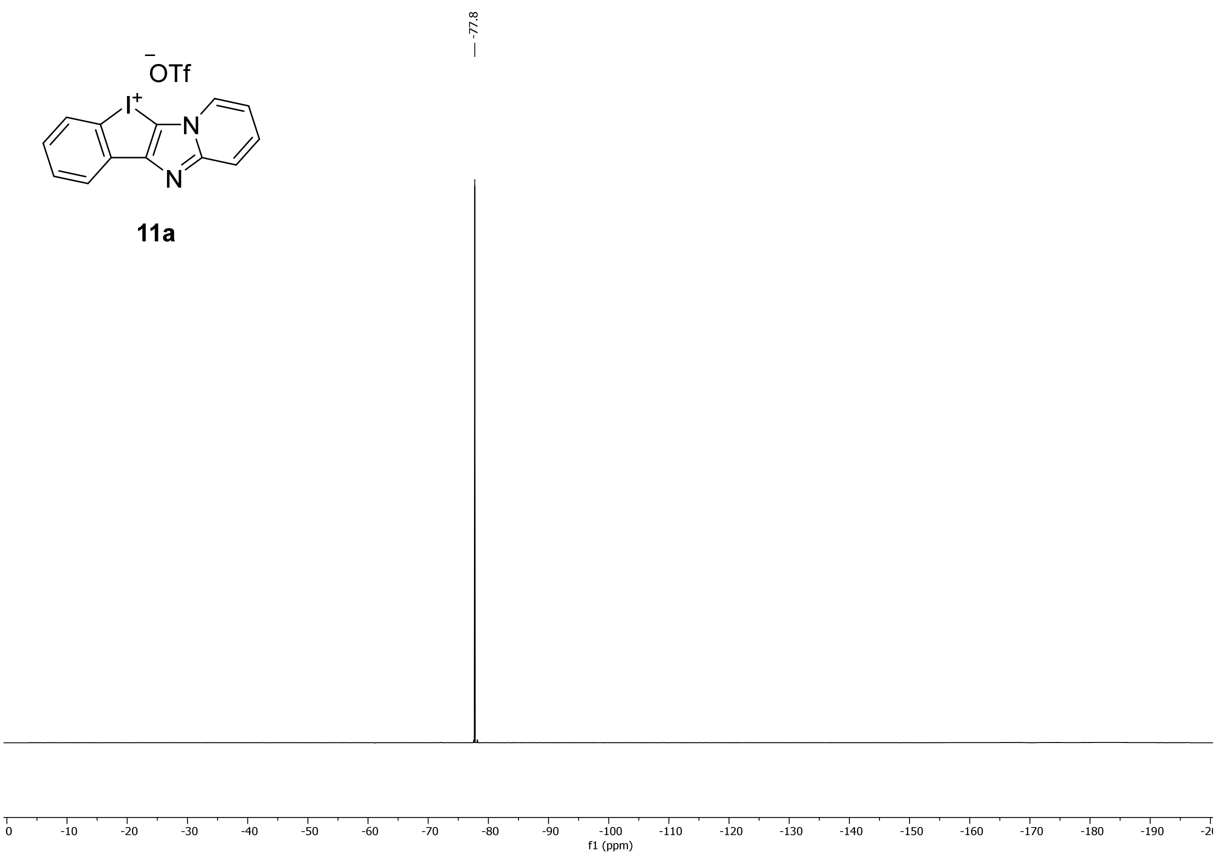

*Figure S47: 600 MHz ^{1}H-, 151 MHz ^{13}C- and 565 MHz ^{19}F-NMR spectra of benzo[4',5']iodolo[3',2':4,5]imidazo[1,2-a]pyridin-5-ium triflate (**11a**) in d_6-DMSO.*

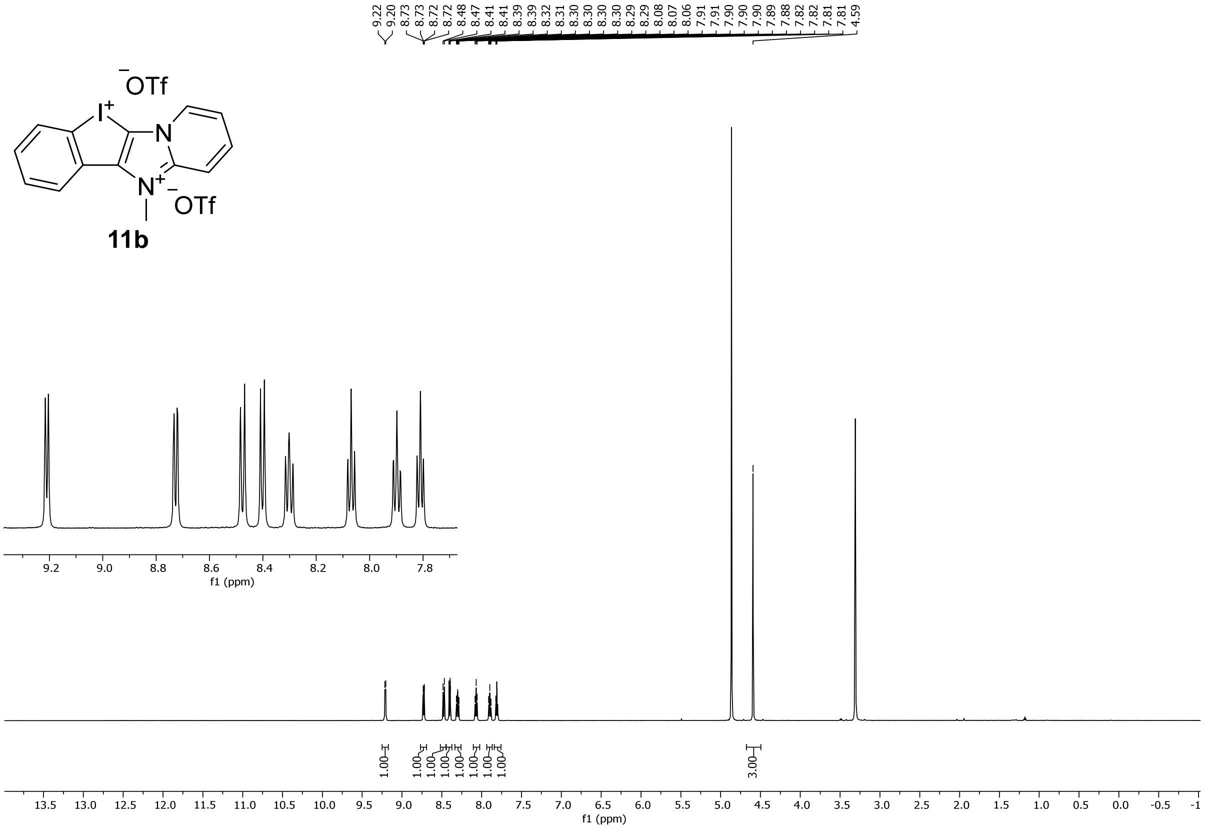

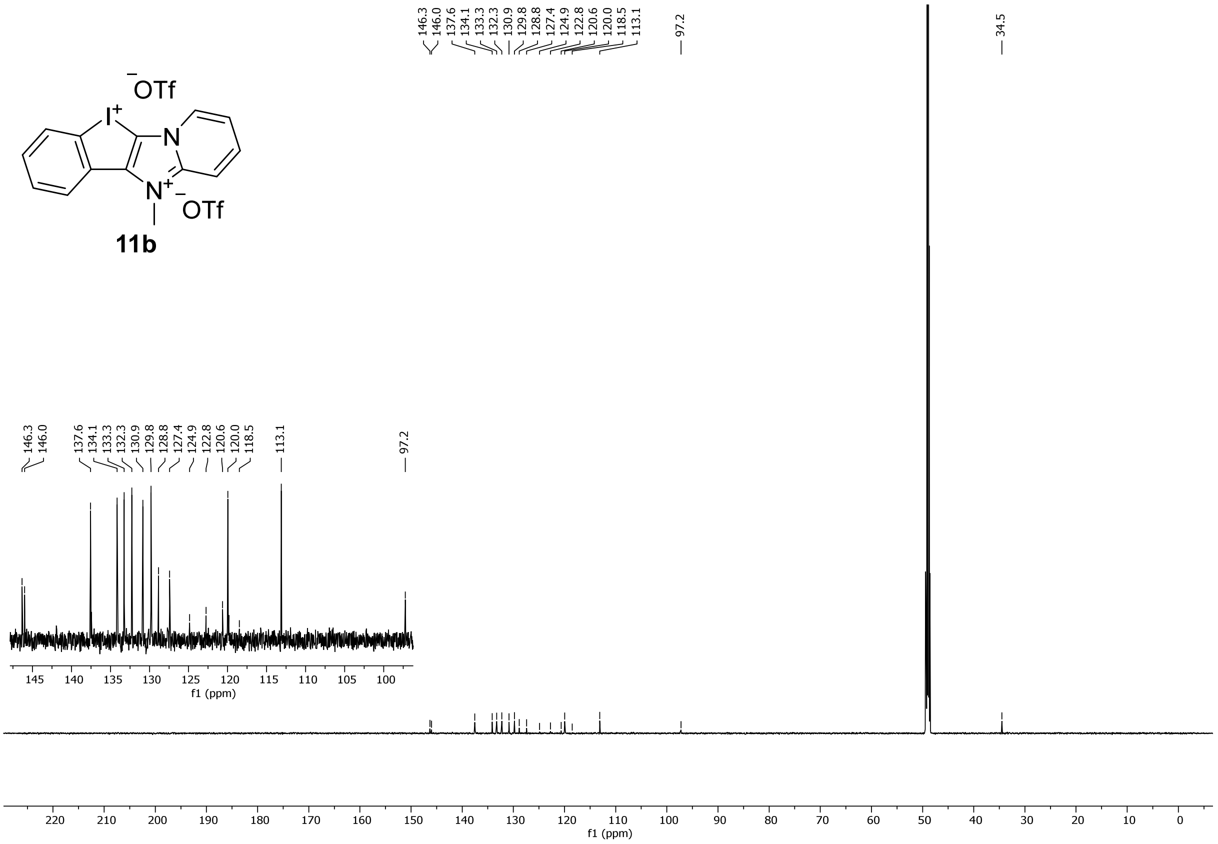

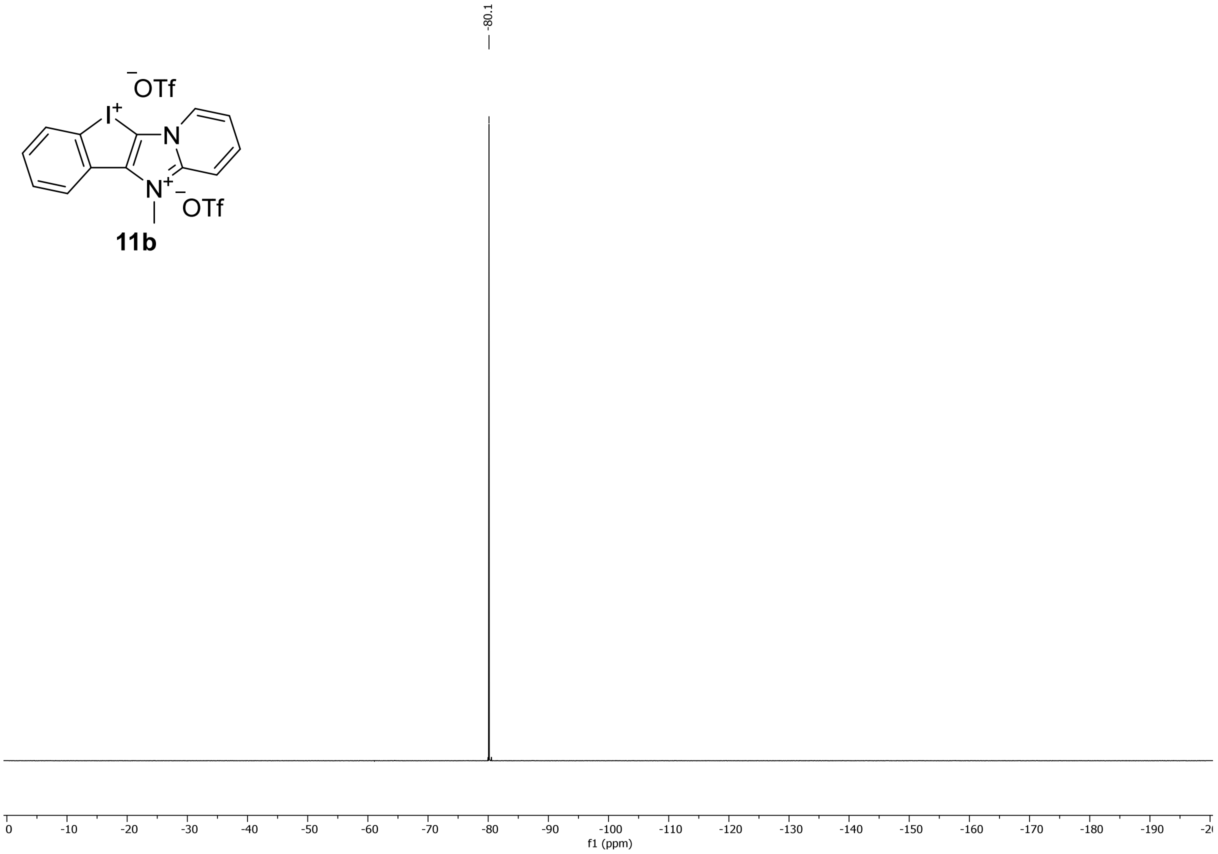

*Figure S48: 600 MHz ^{1}H-, 151 MHz ^{13}C- and 565 MHz ^{19}F-NMR spectra of 11-methylbenzo[4',5']iodolo[3',2':4,5]imidazo[1,2-a]pyridine-5,11-diium bistriflate (**11b**) in d_3-MeOD.*

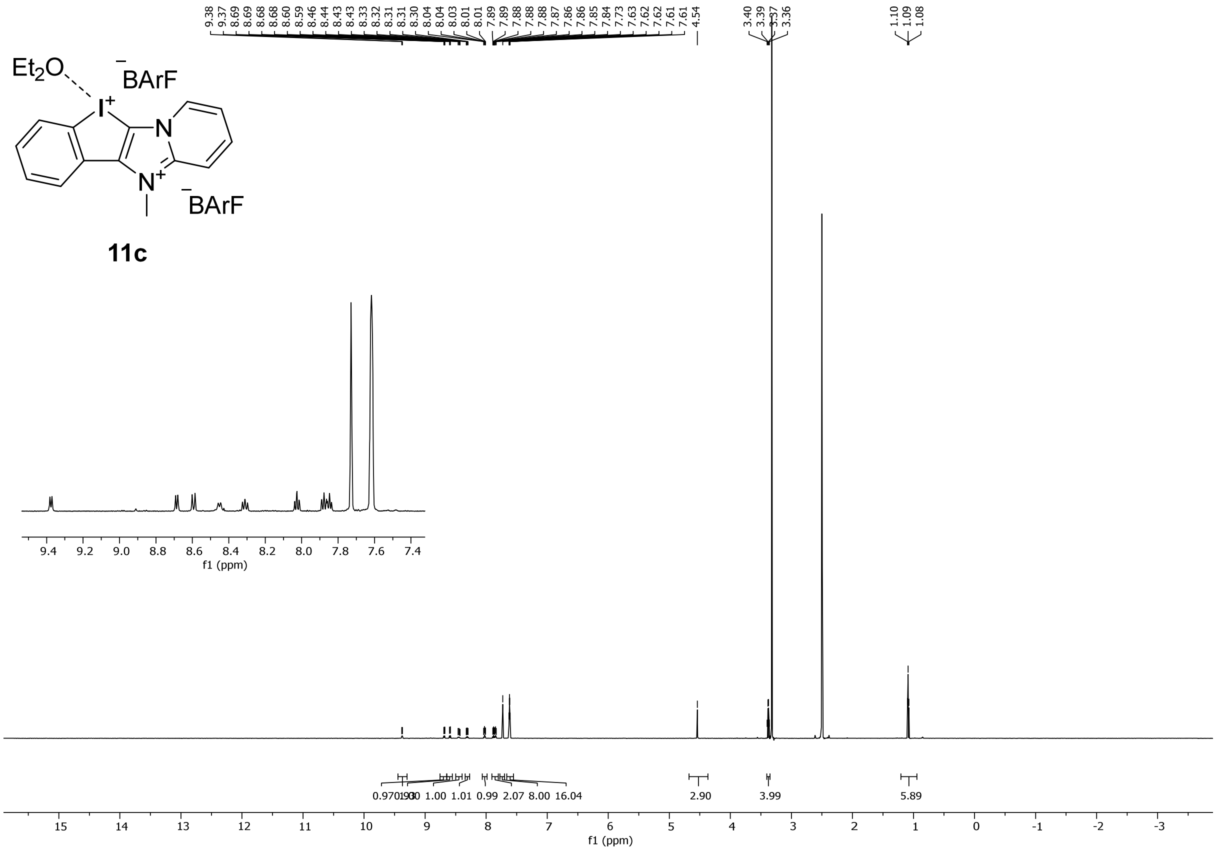
Et2O
BArF
I+
N
N+
BArF
11c
9.38 9.37 8.69 8.69 8.68 8.68 8.60 8.59 8.46 8.44 8.43 8.43 8.33 8.32 8.31 8.31 8.30 8.04 8.04 8.03 8.01 8.01 7.89 7.89 7.88 7.88 7.88 7.87 7.86 7.86 7.85 7.84 7.73 7.63 7.62 7.62 7.61 7.61 4.54
3.40 3.39 3.37 3.36
1.10 1.09 1.08
f1 (ppm)
0.970.900 1.00 1.01 0.99 2.07 8.00 16.04
2.90
3.99
5.89
f1 (ppm)

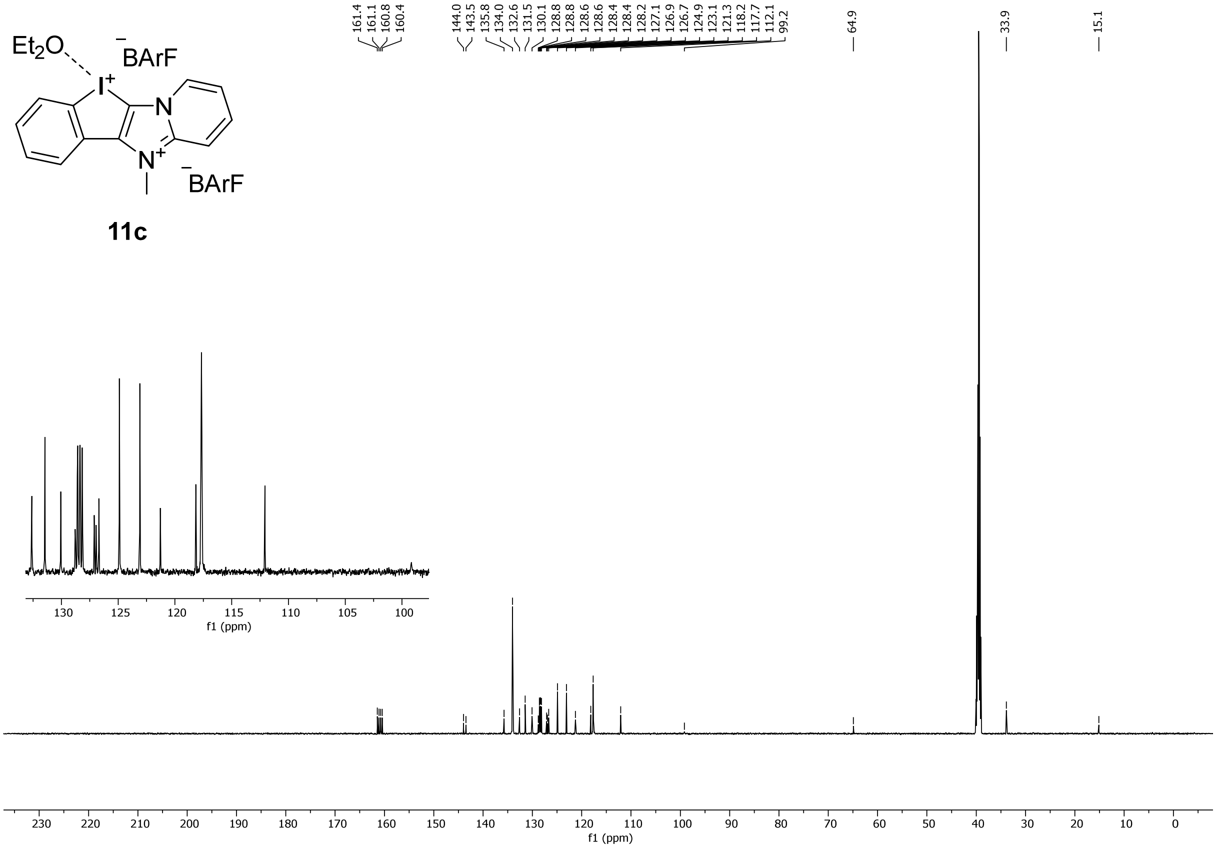
Et2O
BArF
I+
N
N+
BArF
11c
161.4 161.1 160.8 160.4
144.0 143.5 135.8 134.0 132.6 131.5 130.1 128.8 128.8 128.6 128.4 128.2 127.1 126.9 126.7 124.9 123.1 121.3 118.2 117.7 112.1 99.2
64.9
33.9
15.1
f1 (ppm)
f1 (ppm)

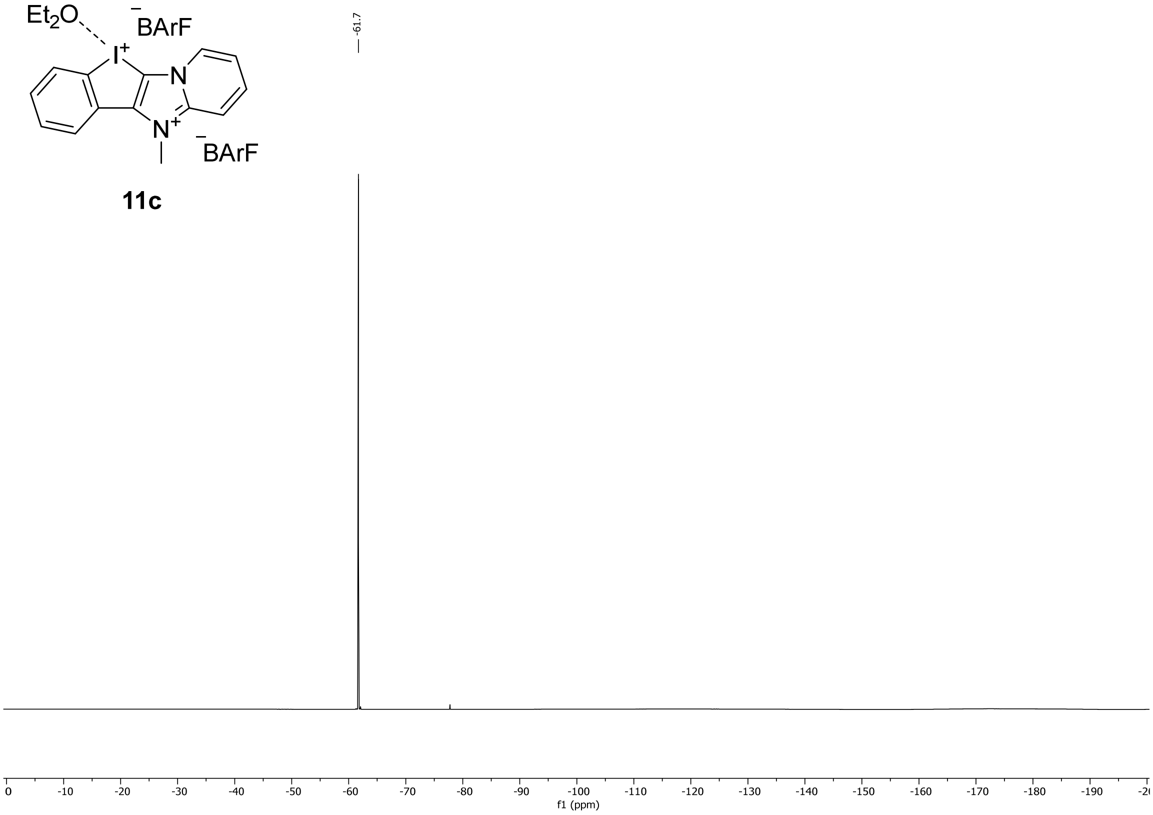

*Figure S49: 600 MHz ^{1}H-, 151 MHz ^{13}C- and 565 MHz ^{19}F-NMR spectra of 11-methylbenzo[4',5']iodolo[3',2':4,5]imidazo[1,2-a]pyridine-5,11-diium bis(tetrakis(3,5-bis(trifluoromethyl)phenyl)borate) • Et_2O (**11c**) in d_6-DMSO.*

XB-mediated reactions

Ritter-type solvolysis of benzhydryl chloride (12)

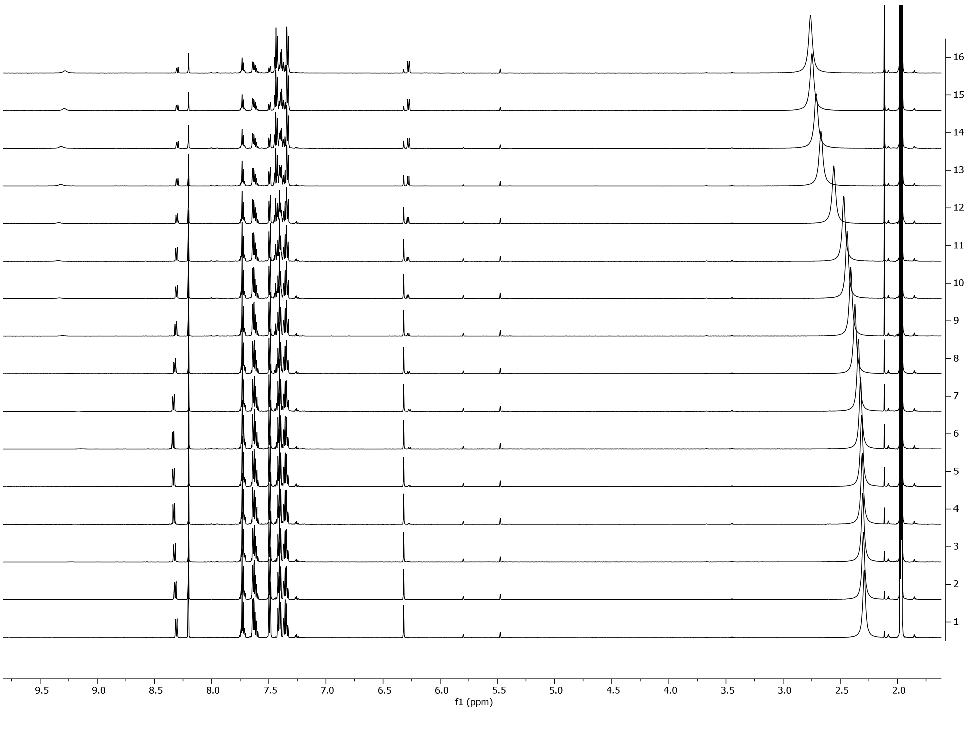

*Figure S50: 600 MHz ^{1}H-NMR spectra for the Ritter type solvolysis of benzhydryl chloride in d_3-MeCN over the course of 72 h employing 1-phenyl-1H-benzo[4,5]iodolo[3,2-c]pyrazol-4-ium triflate (**7a**) as the XB-donor.*

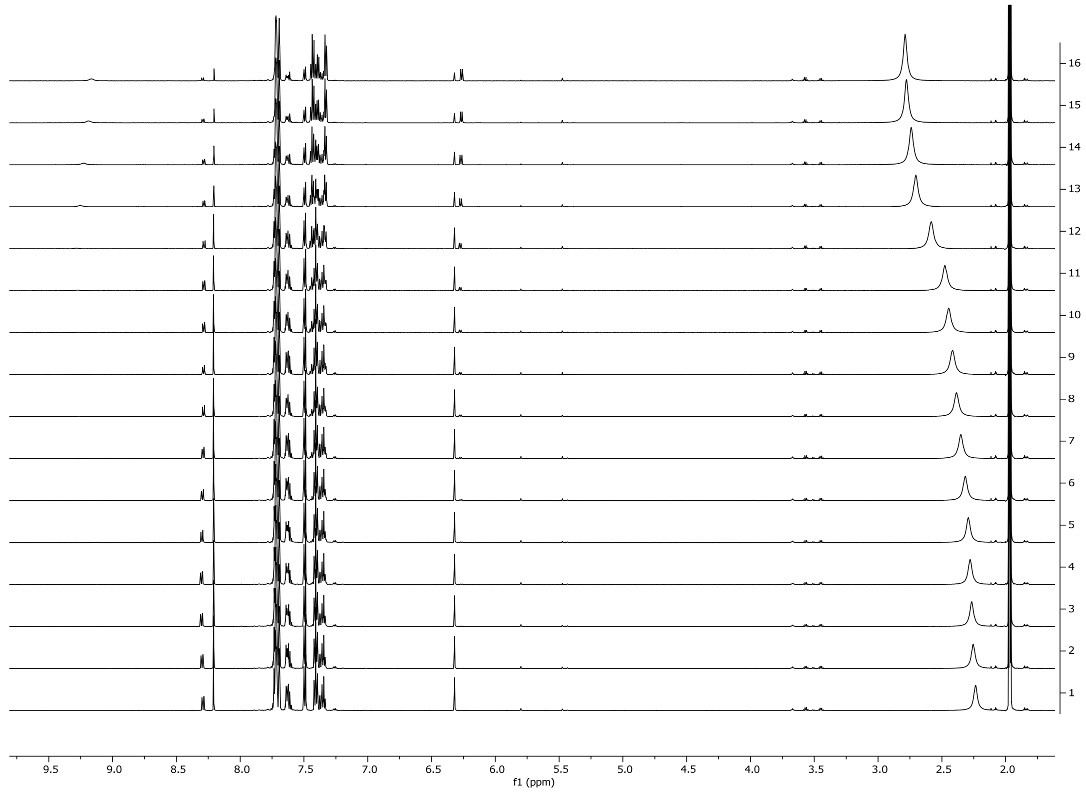

*Figure S51: 600 MHz ^{1}H-NMR spectra for the Ritter type solvolysis of benzhydryl chloride in d_3-MeCN over the course of 72 h employing 1-phenyl-1H-benzo[4,5]iodolo[3,2-c]pyrazol-4-ium tetrakis(3,5-bis(trifluoromethyl)phenyl)borate (**7d**) as the XB-donor.*

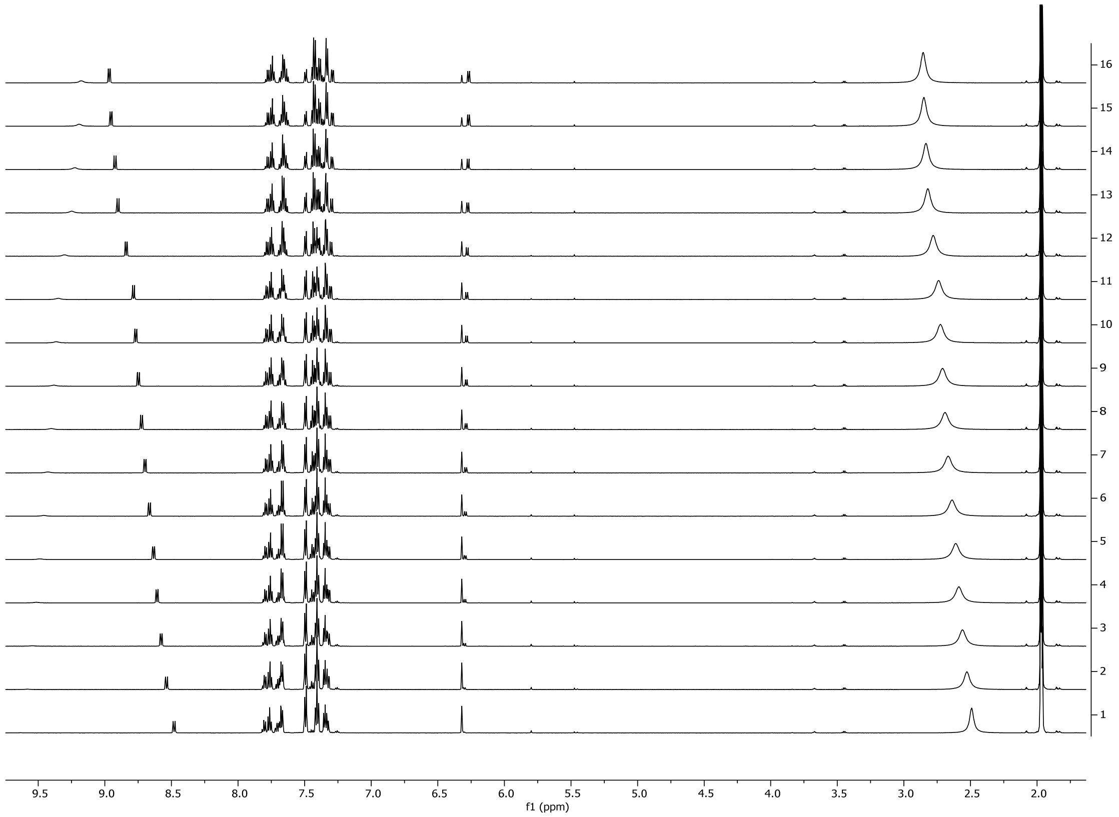

*Figure S52: 600 MHz ^{1}H-NMR spectra for the Ritter type solvolysis of benzhydryl chloride in d_3-MeCN over the course of 72 h employing 1-phenyl-3-(trifluoromethyl)-1H-benzo[4,5]iodolo[3,2-c]pyrazol-4-ium triflate (**7b**) as the XB-donor.*

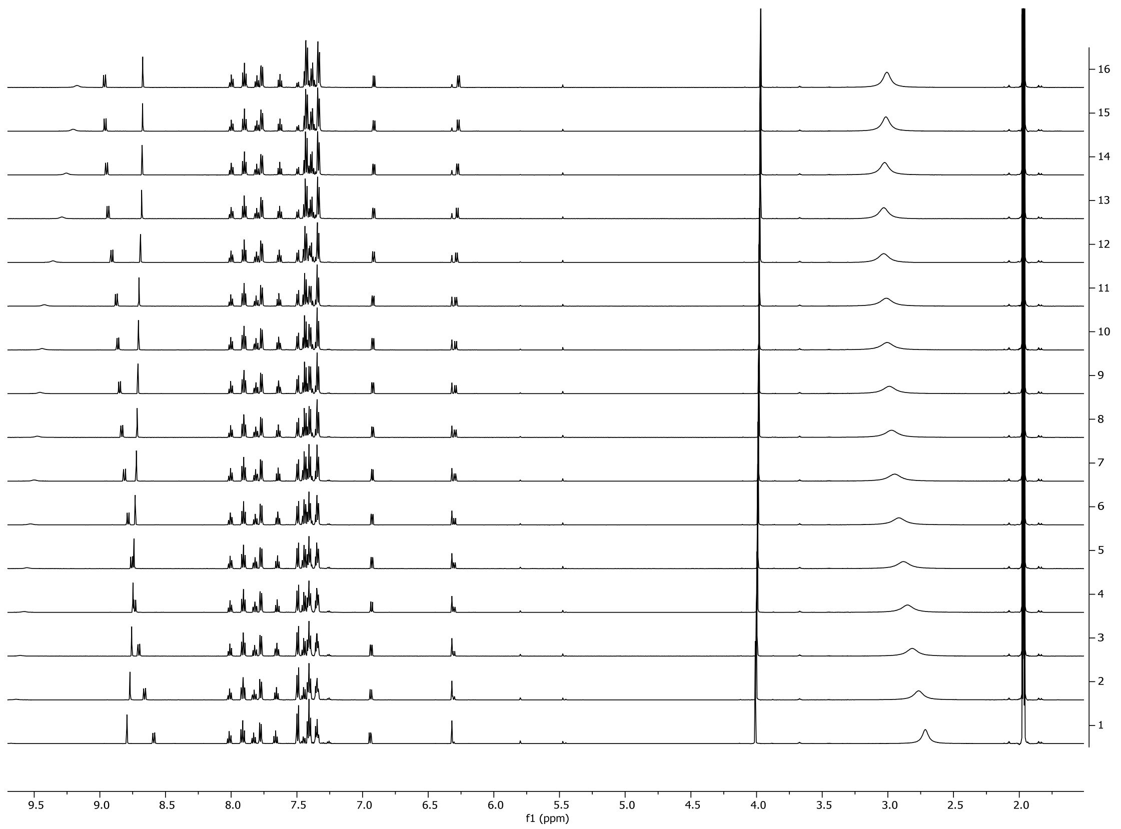

*Figure S53: 600 MHz ^{1}H-NMR spectra for the Ritter type solvolysis of benzhydryl chloride in d_3-MeCN over the course of 72 h employing 2-methyl-1-phenyl-1H-benzo[4,5]iodolo[3,2-c]pyrazole-2,4-diium bistriflate (**7e**) as the XB-donor.*

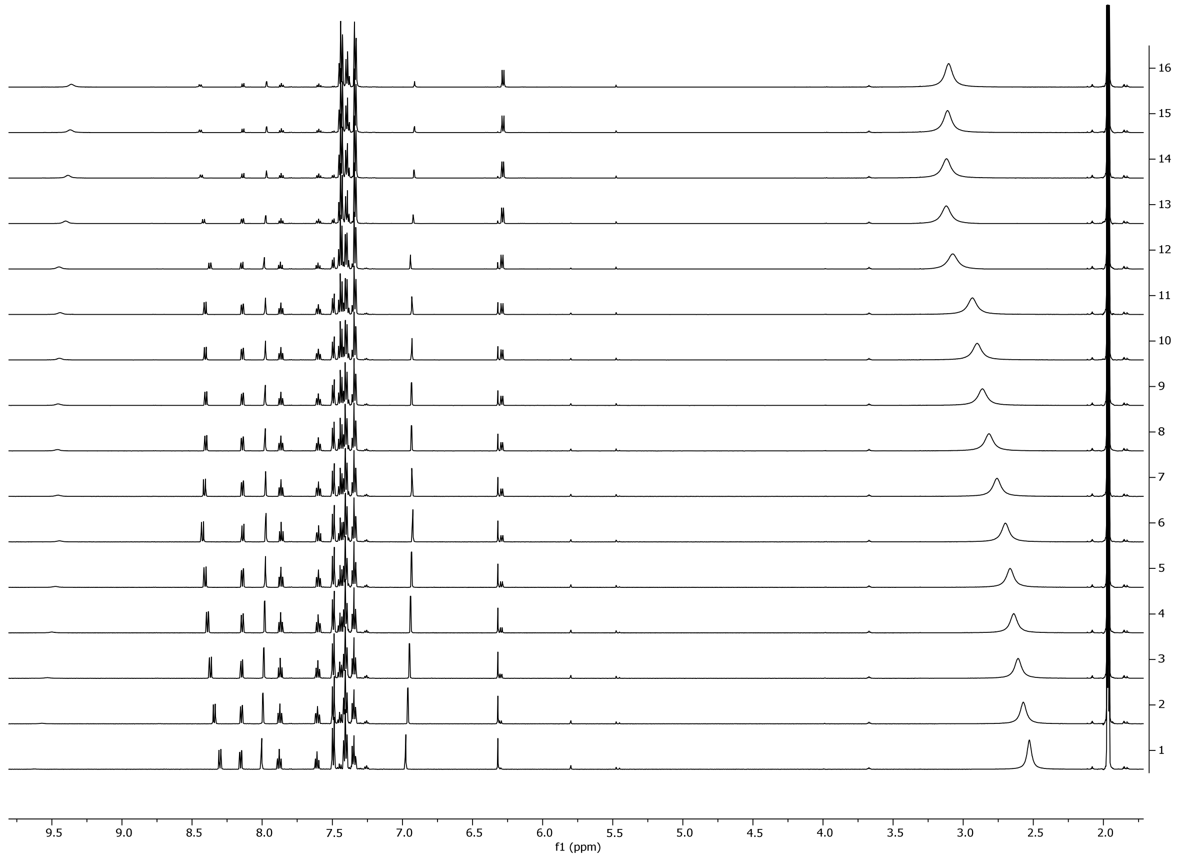

*Figure S54: 600 MHz ^{1}H-NMR spectra for the Ritter type solvolysis of benzhydryl chloride in d_3-MeCN over the course of 72 h employing benzo[d]pyrazolo[5,1-b][1,3]iodazol-4-ium triflate (**8a**) as the XB-donor.*

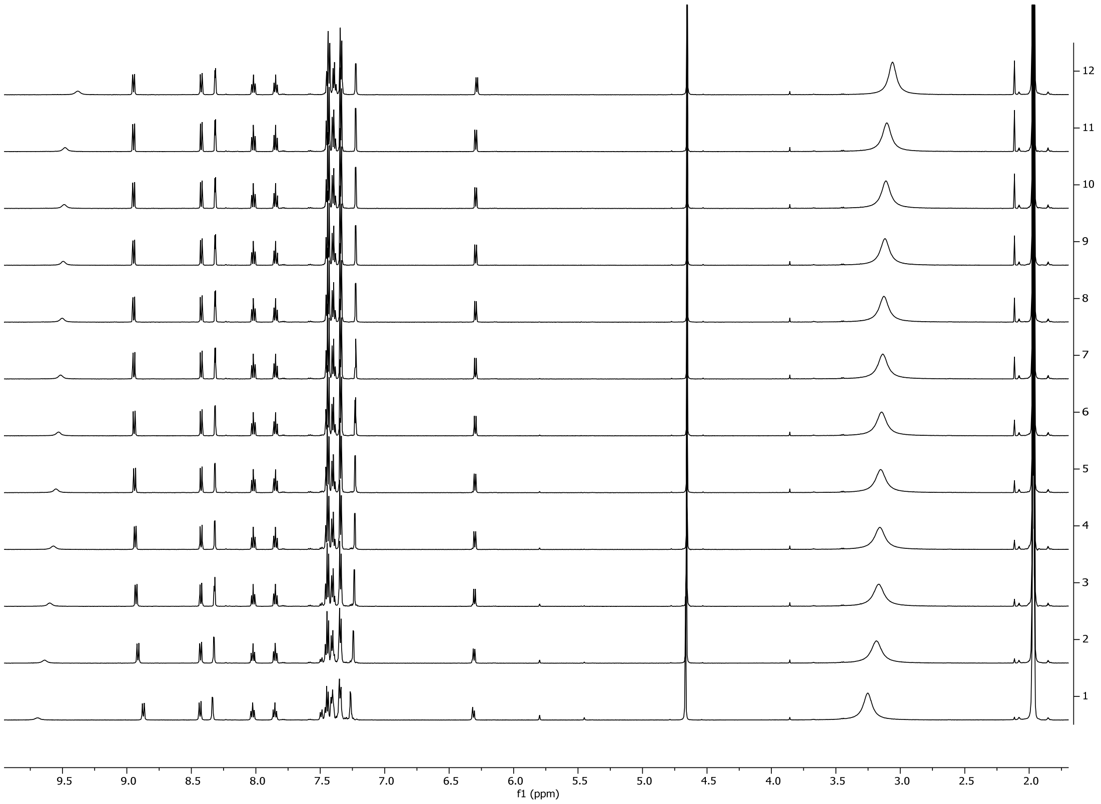

*Figure S55: 600 MHz ^{1}H-NMR spectra for the Ritter type solvolysis of benzhydryl chloride in d_3-MeCN over the course of 24 h employing 1-methylbenzo[d]pyrazolo[5,1-b][1,3]iodazole-1,4-diium bistriflate (**8c**) as the XB-donor.*

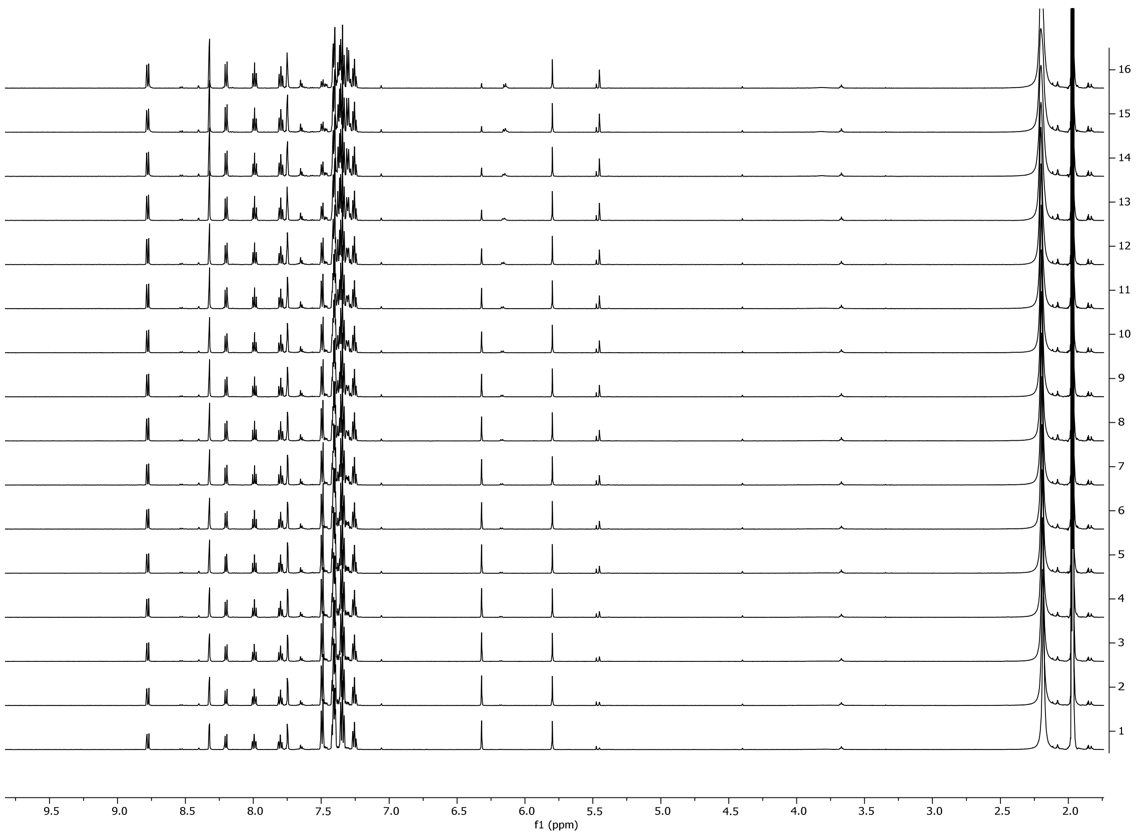

*Figure S56: 600 MHz ^{1}H-NMR spectra for the Ritter type solvolysis of benzhydryl chloride in d_3-MeCN over the course of 72 h employing benzo[d]imidazo[2,1-b][1,3]iodazol-9-ium triflate (**9**) as the XB-donor.*

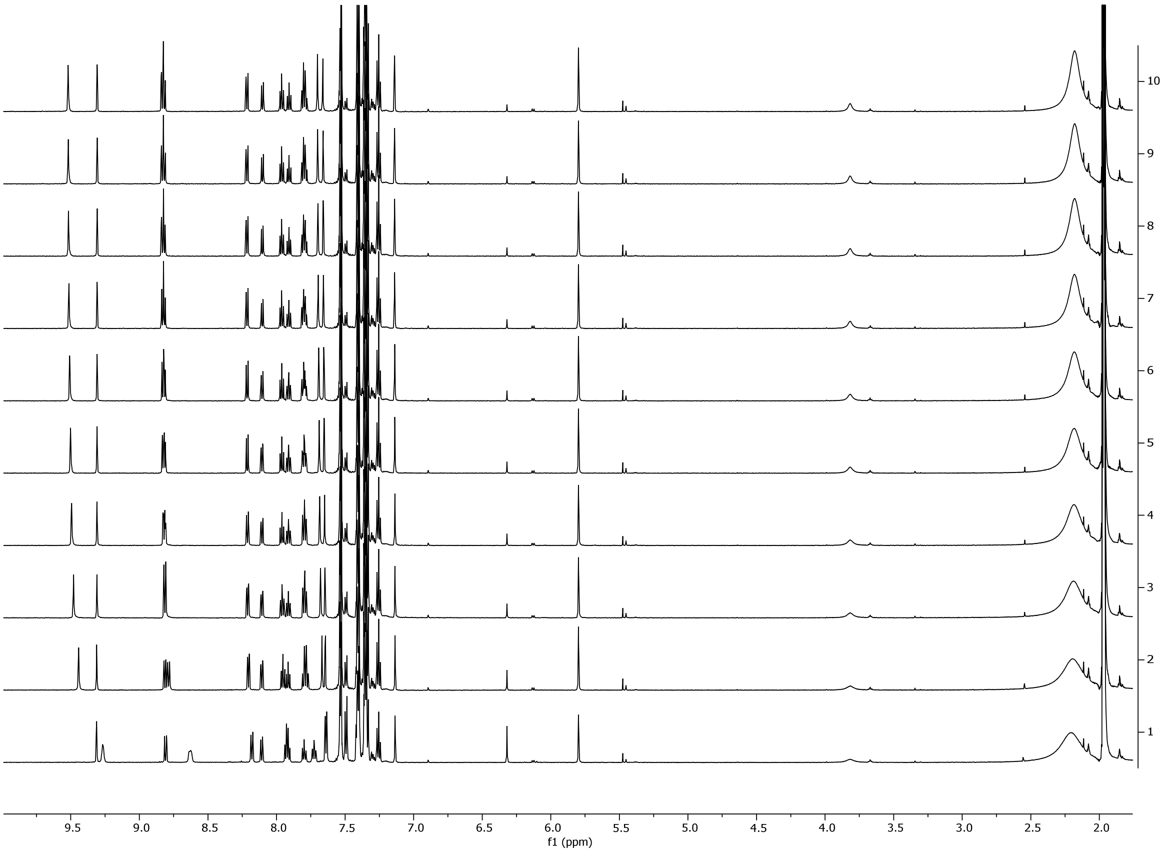

*Figure S57: 600 MHz ^{1}H-NMR spectra for the Ritter type solvolysis of benzhydryl chloride in d_3-MeCN over the course of 14 h employing benzo[d]imidazo[5,1-b][1,3]iodazol-4-diium triflate (**10a**) as the XB-donor.*

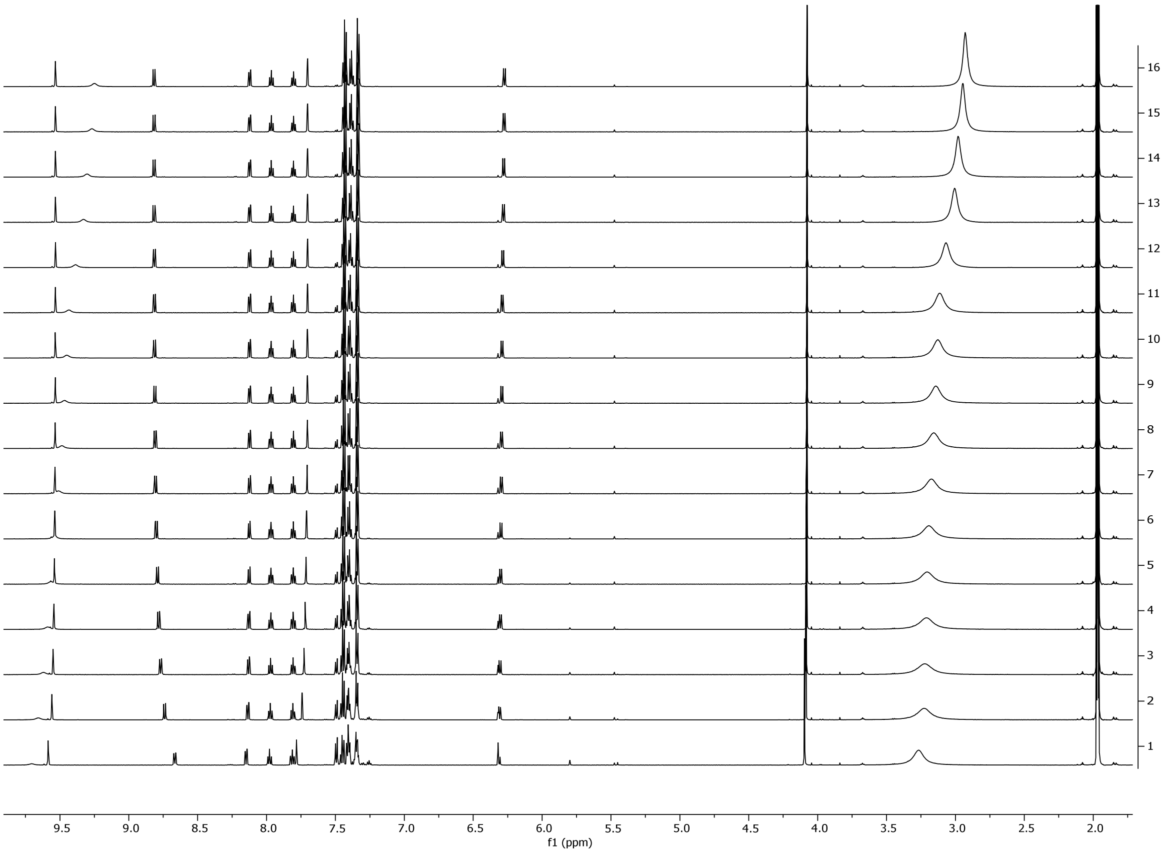

*Figure S58: 600 MHz ^{1}H-NMR spectra for the Ritter type solvolysis of benzhydryl chloride in d_3-MeCN over the course of 72 h employing 2-methylbenzo[d]imidazo[5,1-b][1,3]iodazole-2,4-diium bistriflate (**10b**) as the XB-donor.*

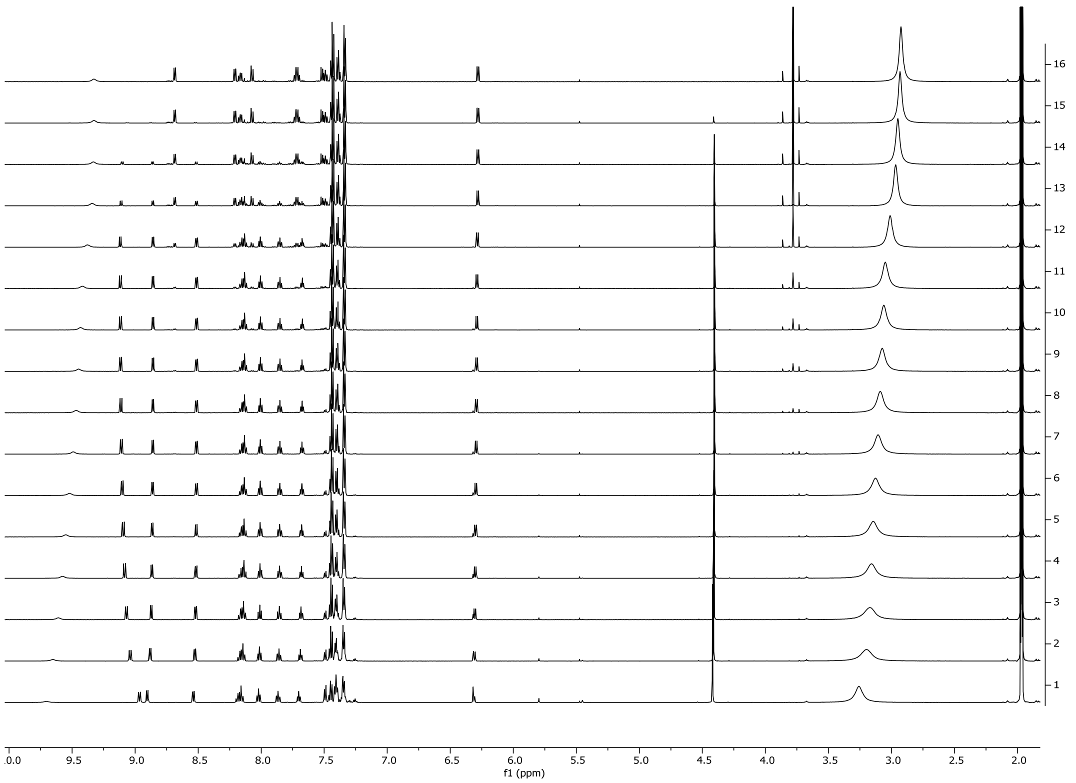

*Figure S59: 600 MHz ^{1}H-NMR spectra for the Ritter type solvolysis of benzhydryl chloride in d_3-MeCN over the course of 72 h employing 11-methylbenzo[4',5']iodolo[3',2':4,5]imidazo[1,2-a]pyridine-5,11-diium bistriflate (**11b**) as the XB-donor. As soon as most of the starting material was consumed, the iodonium salt starts to decompose, probably via ring opening with Cl^-.*

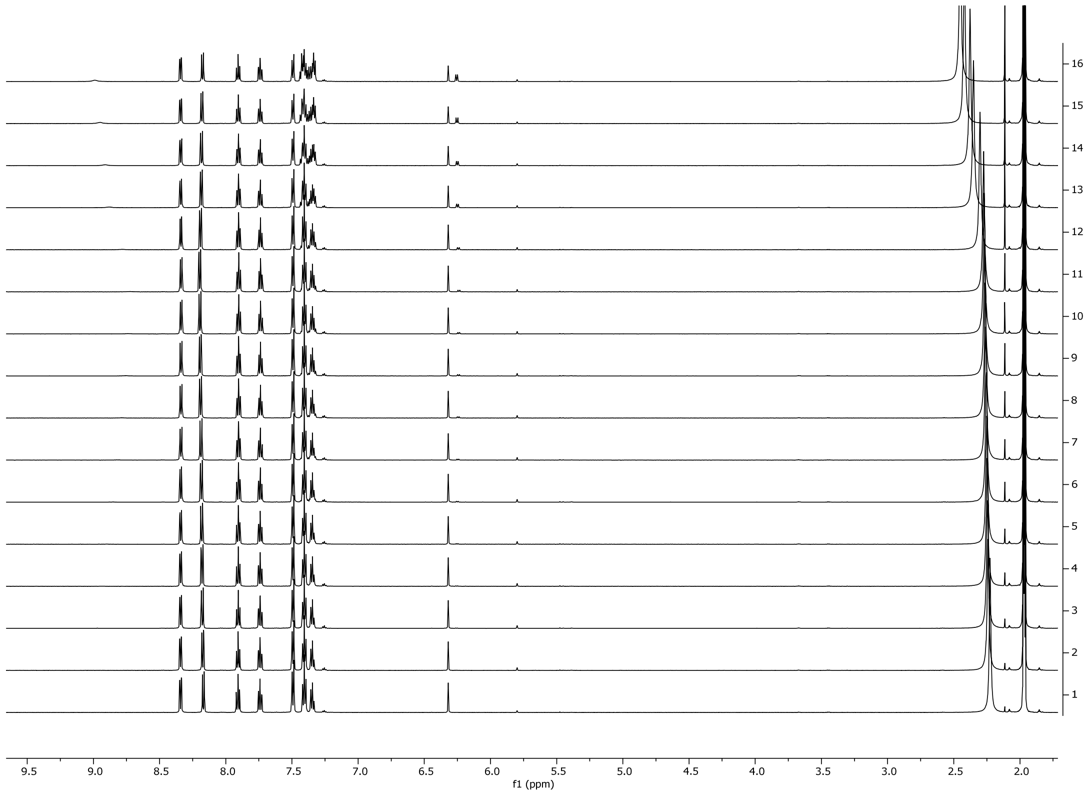

*Figure S60: 600 MHz ^{1}H-NMR spectra for the Ritter type solvolysis of benzhydryl chloride in d_3-MeCN over the course of 72 h employing dibenzo[b,d]iodol-5-ium triflate (**4a**) as the XB-donor.*

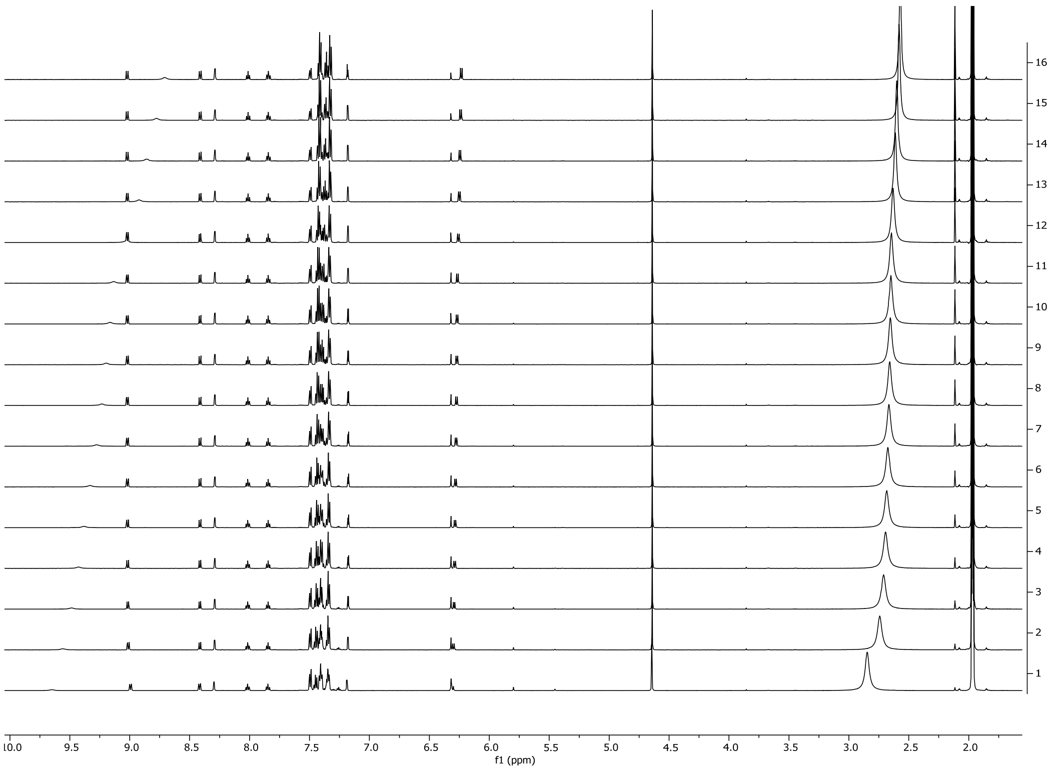

*Figure S61: 600 MHz ^{1}H-NMR spectra for the Ritter type solvolysis of benzhydryl chloride in d_3-MeCN over the course of 24 h employing 50 mol% of 1-methylbenzo[d]pyrazolo[5,1-b][1,3]iodazole-1,4-diium bistriflate (**8c**) as the XB-donor.*

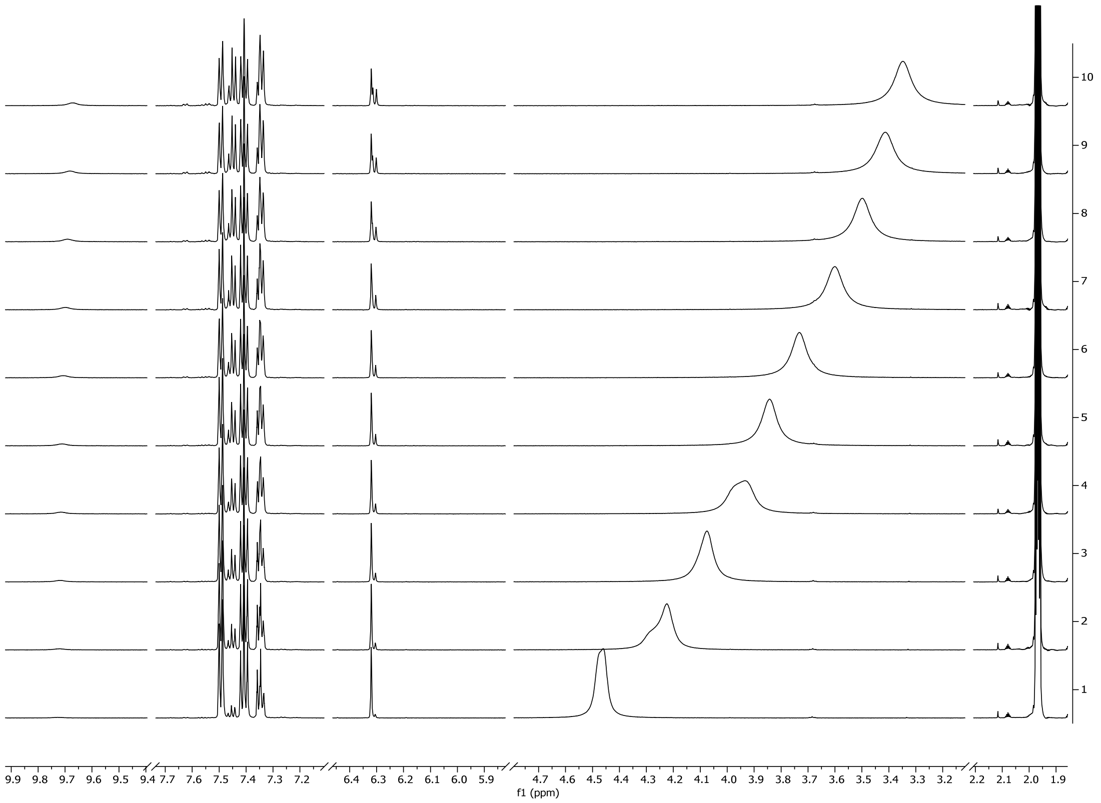

Figure S62: 600 MHz ^{1}H-NMR spectra for the Ritter type solvolysis of benzhydryl chloride in d_3-MeCN over the course of 14 h employing TfOH as the activator.

Ritter-type solvolysis of α-methylbenzyl chloride (14)

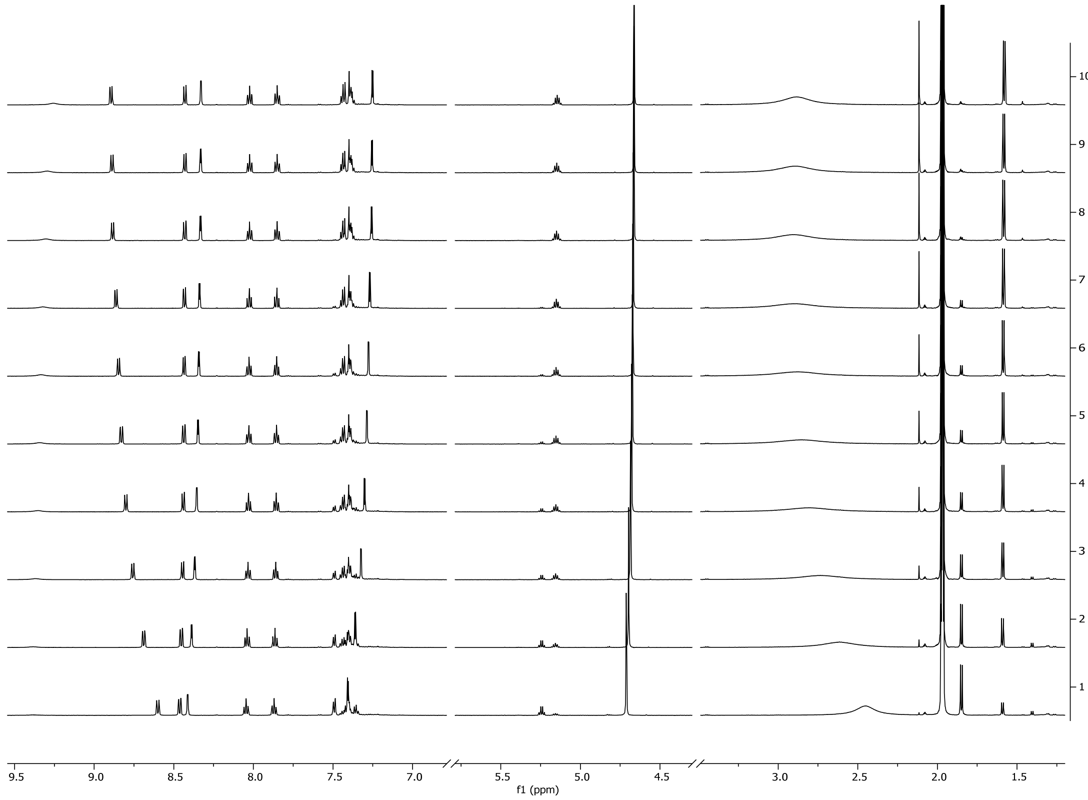

*Figure S63: 600 MHz ^{1}H-NMR spectra for the Ritter type solvolysis of α-methylbenzyl chloride in d_3-MeCN over the course of 72 h employing 1-methylbenzo[d]pyrazolo[5,1-b][1,3]iodazole-1,4-diium bistriflate (**8c**) as the XB-donor.*

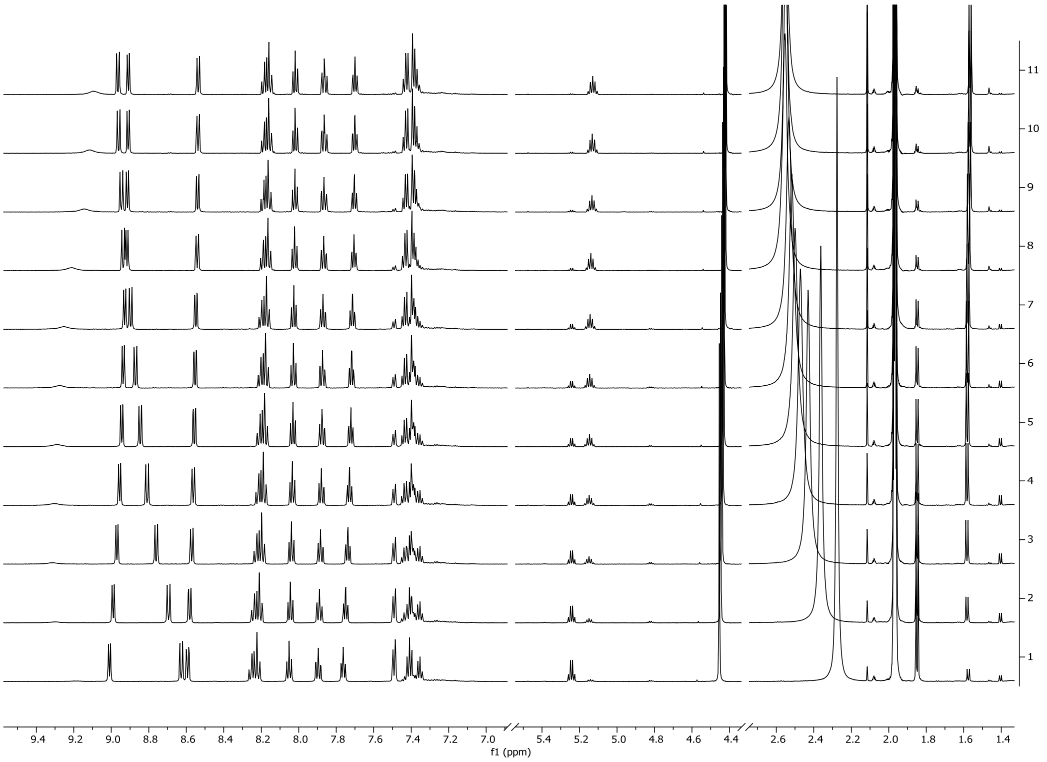

*Figure S64: 600 MHz ^{1}H-NMR spectra for the Ritter type solvolysis of α-methylbenzyl chloride in d_3-MeCN over the course of 72 h employing 11-methylbenzo[4',5']iodolo[3',2':4,5]imidazo[1,2-a]pyridine-5,11-diium bistriflate (**11b**) as the XB-donor.*

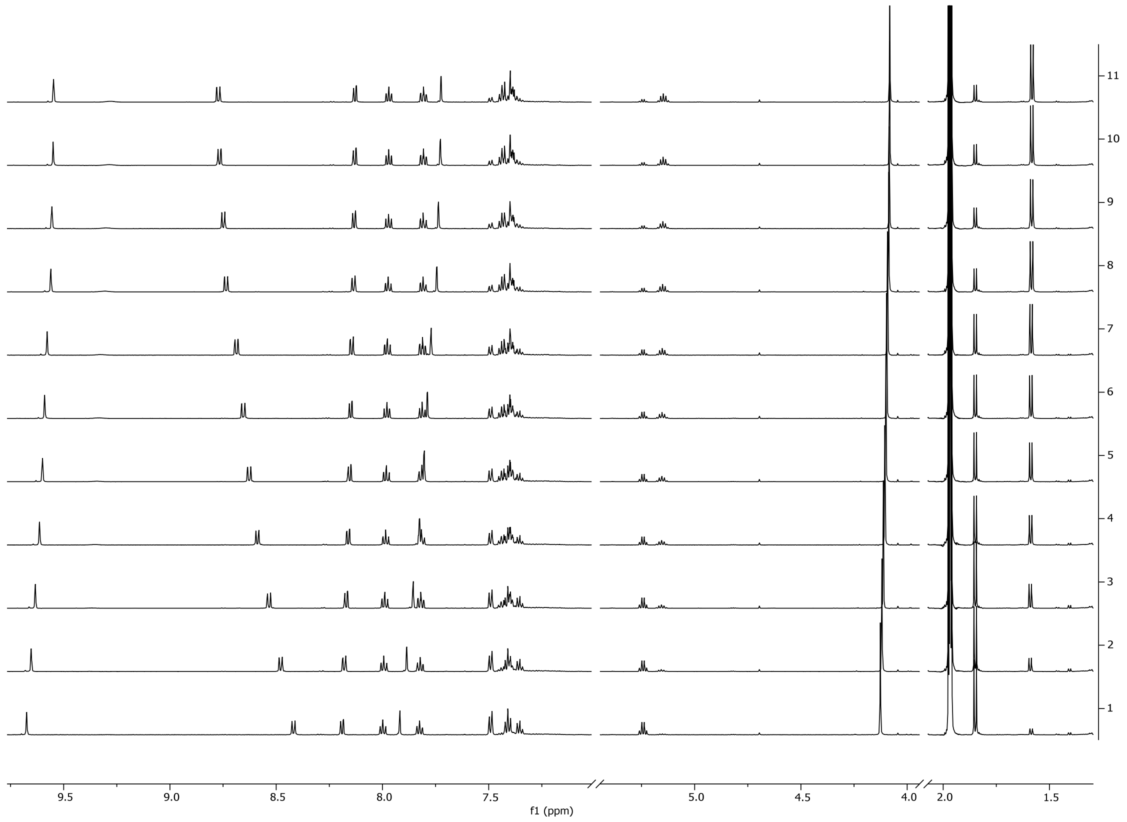

*Figure S65: 600 MHz ^{1}H-NMR spectra for the Ritter type solvolysis of α-methylbenzyl chloride in d_3-MeCN over the course of 72 h employing 2-methylbenzo[d]imidazo[5,1-b][1,3]iodazole-2,4-diium bistriflate (**10b**) as the XB-donor.*

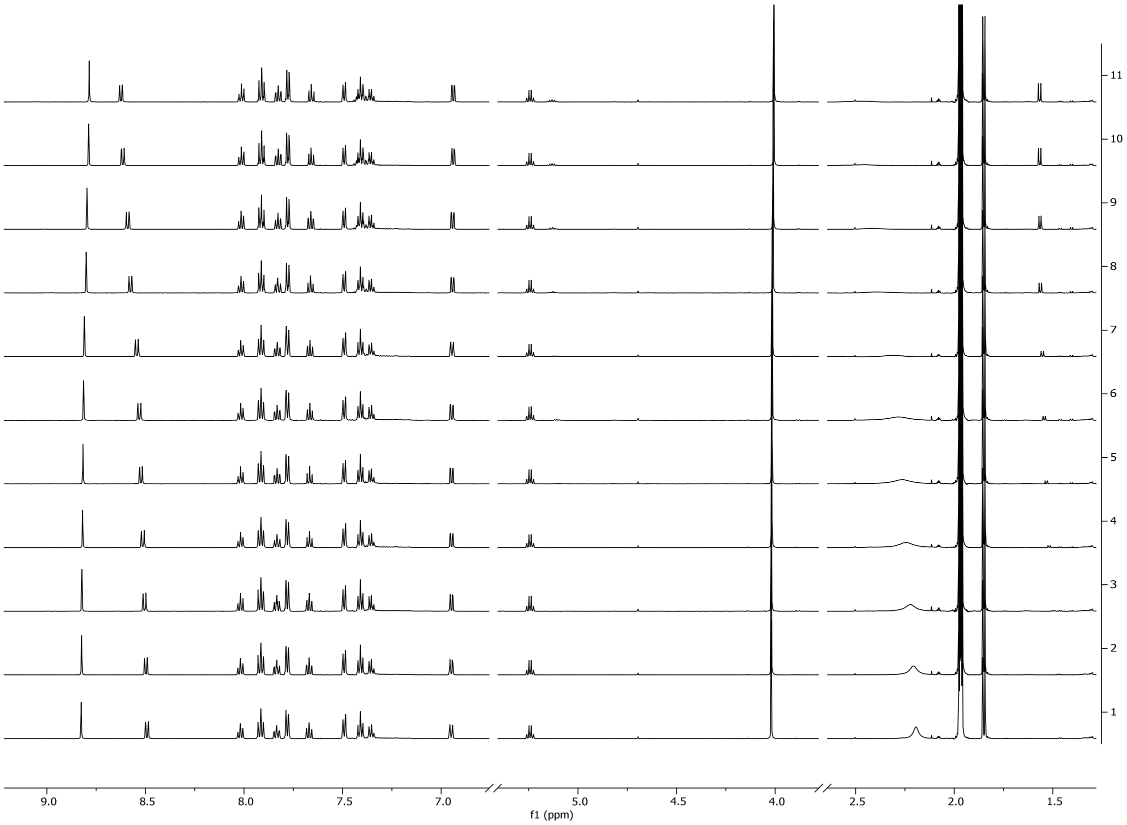

*Figure S66: 600 MHz ^{1}H-NMR spectra for the Ritter type solvolysis of α-methylbenzyl chloride in d_3-MeCN over the course of 72 h employing 2-methyl-1-phenyl-1H-benzo[4,5]iodolo[3,2-c]pyrazole-2,4-diium bistriflate (**7e**) as the XB-donor.*

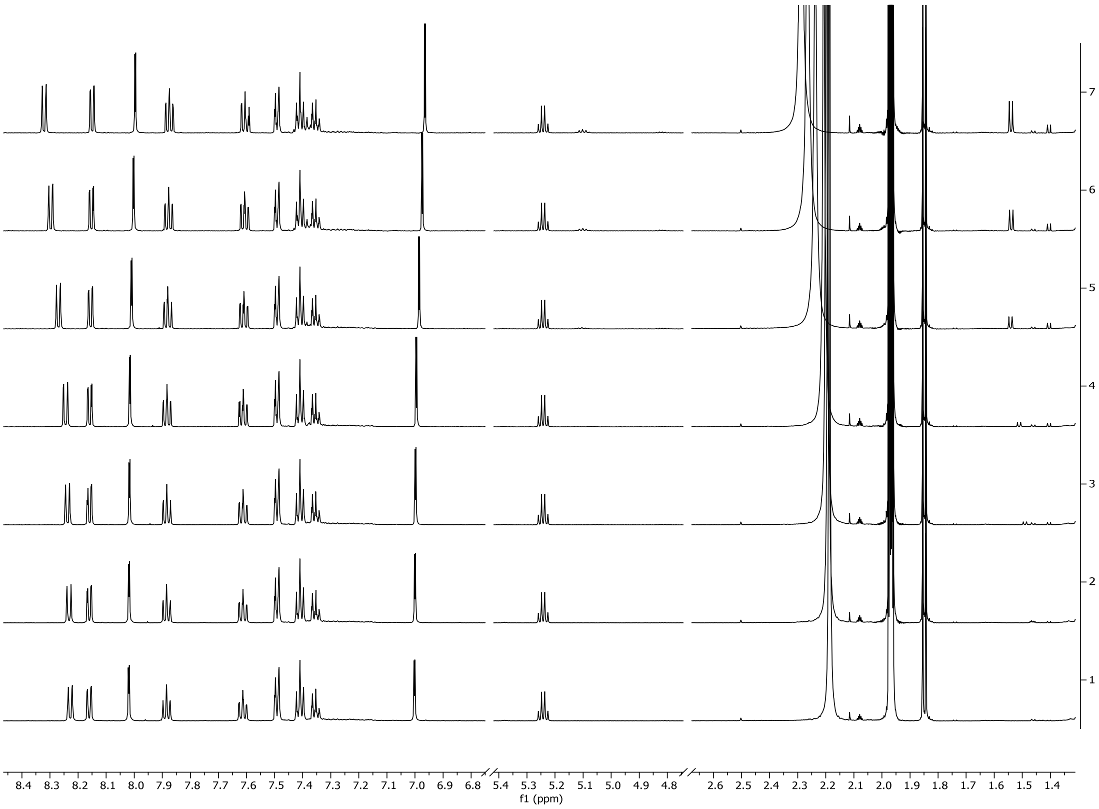

*Figure S67: 600 MHz ¹H-NMR spectra for the Ritter type solvolysis of α-methylbenzyl chloride in d_3-MeCN over the course of 72 h employing benzo[d]pyrazolo[5,1-b][1,3]iodazol-4-ium triflate (**8a**) as the XB-donor.*

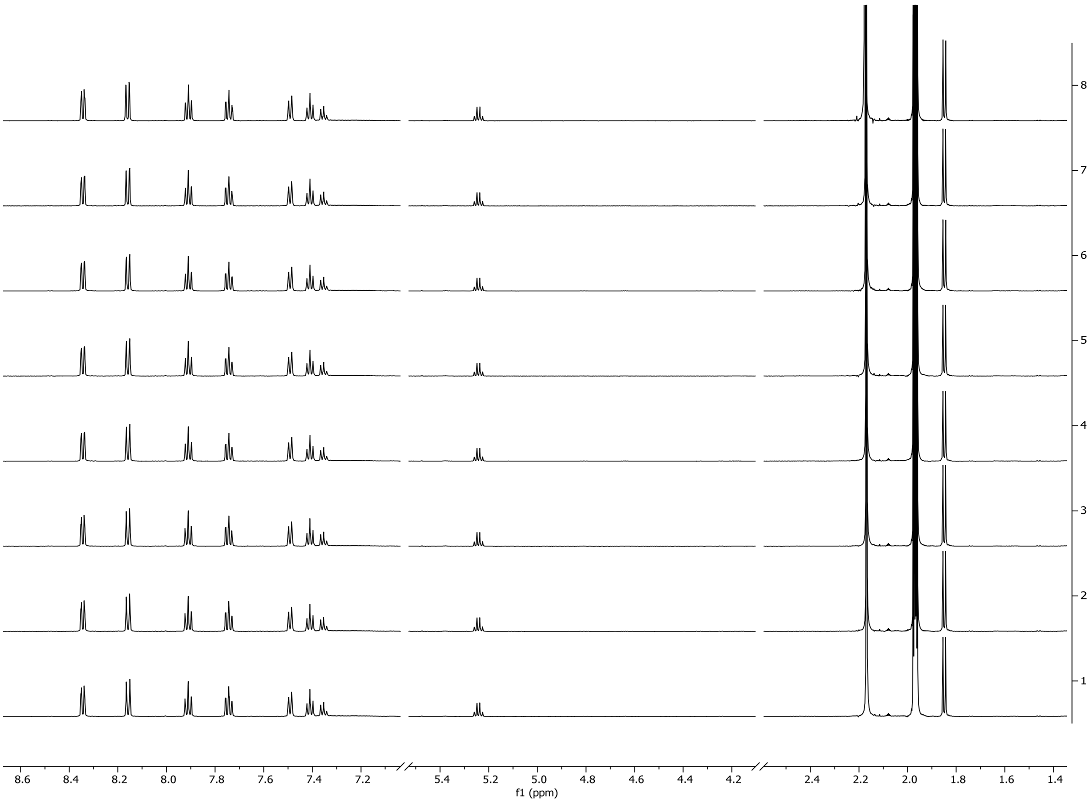

*Figure S68: 600 MHz ¹H-NMR spectra for the Ritter type solvolysis of α-methylbenzyl chloride in d_3-MeCN over the course of 72 h employing dibenzo[b,d]iodol-5-ium triflate (**4a**) as the XB-donor.*

Gold(I)-catalyzed cyclization of propargylic amide 17

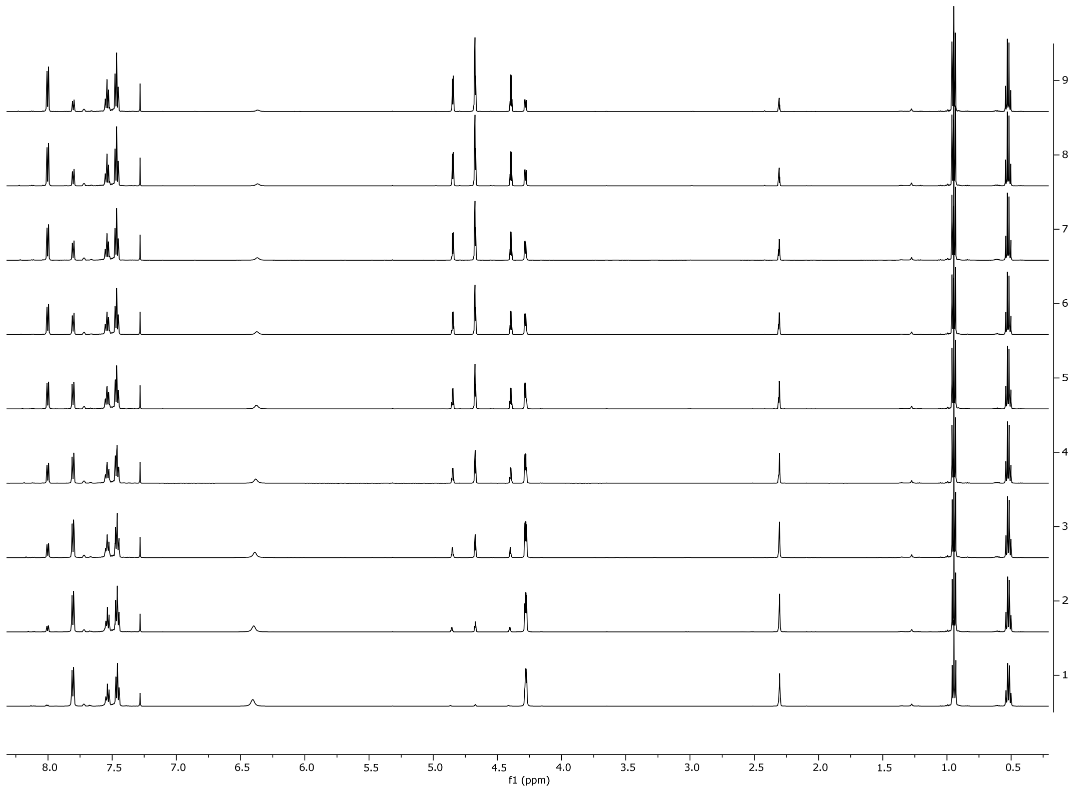

*Figure S69: 600 MHz ^{1}H-NMR spectra for the gold(I)-catalyzed cyclization of propargylic amide over the course of 10 h employing 1-phenyl-1H-benzo[4,5]iodolo[3,2-c]pyrazol-4-ium tetrakis(3,5-bis(trifluoromethyl)phenyl)borate (**7d**) as the XB-donor.*

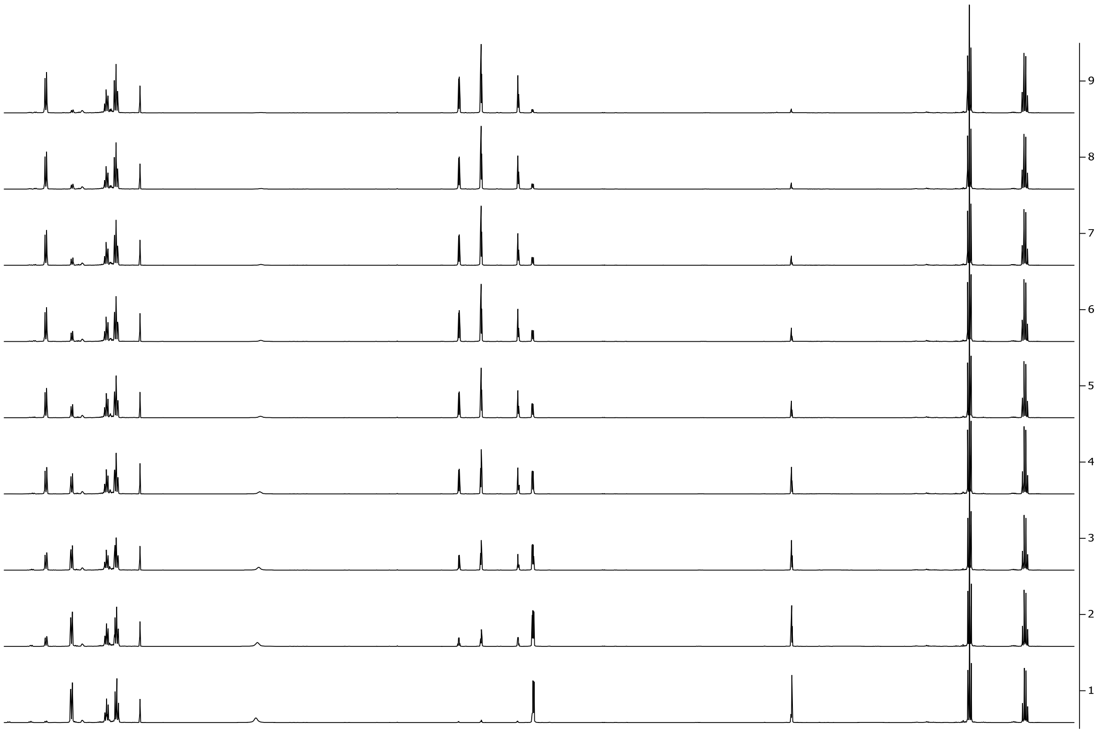

*Figure S70: 600 MHz ^{1}H-NMR spectra for the gold(I)-catalyzed cyclization of propargylic amide over the course of 10 h employing dibenzo[b,d]iodol-5-ium tetrakis(3,5-bis(trifluoromethyl)phenyl)borate (**4b**) as the XB-donor.*

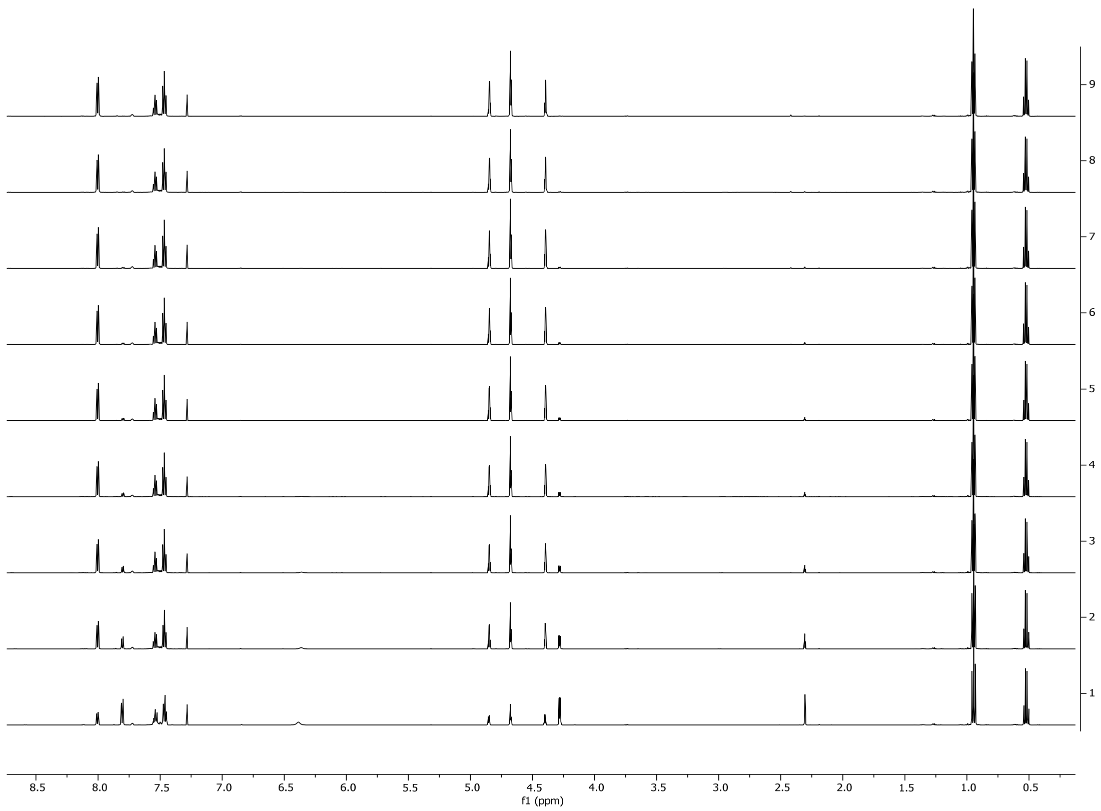

*Figure S71: 600 MHz ¹H-NMR spectra for the gold(I)-catalyzed cyclization of propargylic amide over the course of 10 h employing benzo[d]pyrazolo[5,1-b][1,3]iodazol-4-ium tetrakis(3,5-bis(trifluoromethyl)phenyl)borate (**8b**) as the XB-donor.*

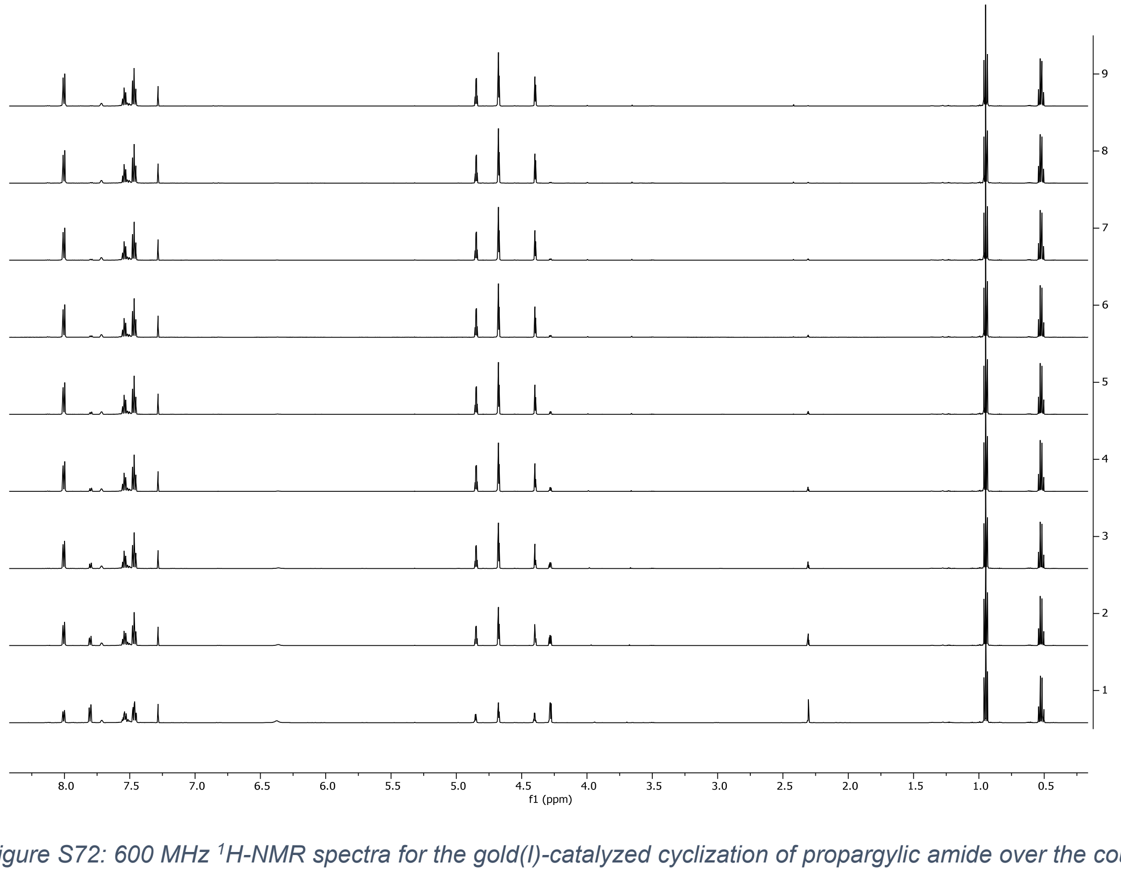

*Figure S72: 600 MHz ¹H-NMR spectra for the gold(I)-catalyzed cyclization of propargylic amide over the course of 10 h employing 2-methyl-1-phenyl-1H-benzo[4,5]iodolo[3,2-c]pyrazole-2,4-diium bis(tetrakis(3,5-bis(trifluoromethyl)phenyl)borate) • Et_2O (**7f**) as the XB-donor.*

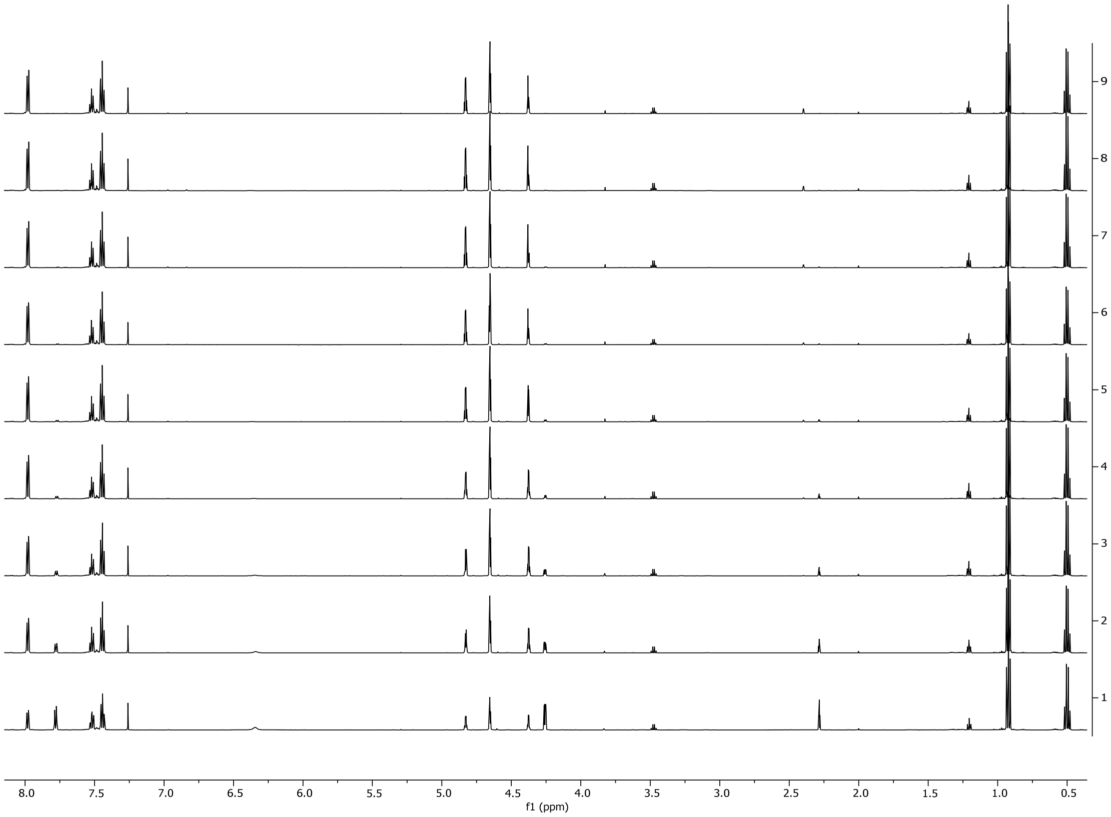

*Figure S73: 600 MHz ^{1}H-NMR spectra for the gold(I)-catalyzed cyclization of propargylic amide over the course of 10 h employing 1-methylbenzo[d]pyrazolo[5,1-b][1,3]iodazole-1,4-diium bis(tetrakis(pentafluorophenyl)borate) dietherate complex (**8d**) as the XB-donor.*

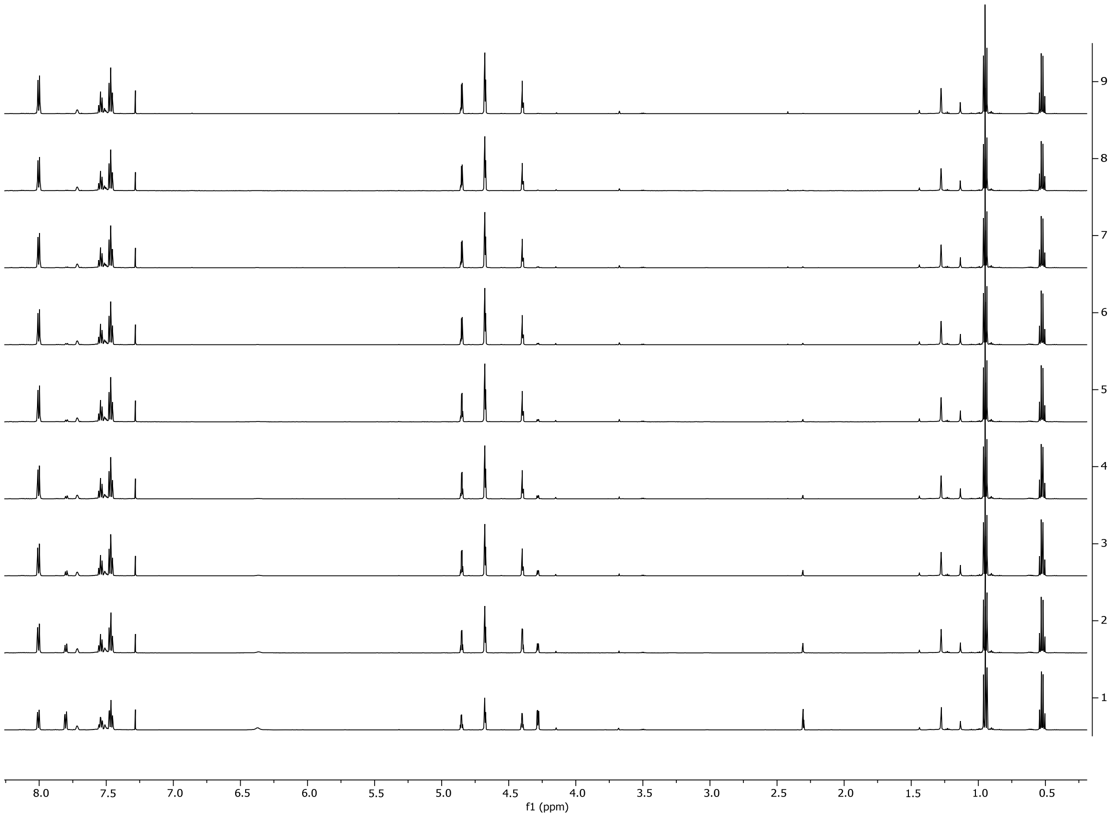

*Figure S74: 600 MHz ^{1}H-NMR spectra for the gold(I)-catalyzed cyclization of propargylic amide over the course of 10 h employing 11-methylbenzo[4',5']iodolo[3',2':4,5]imidazo[1,2-a]pyridine-5,11-diium bis(tetrakis(3,5-bis(trifluoromethyl)phenyl)borate) • Et_2O (**11c**) as the XB-donor.*

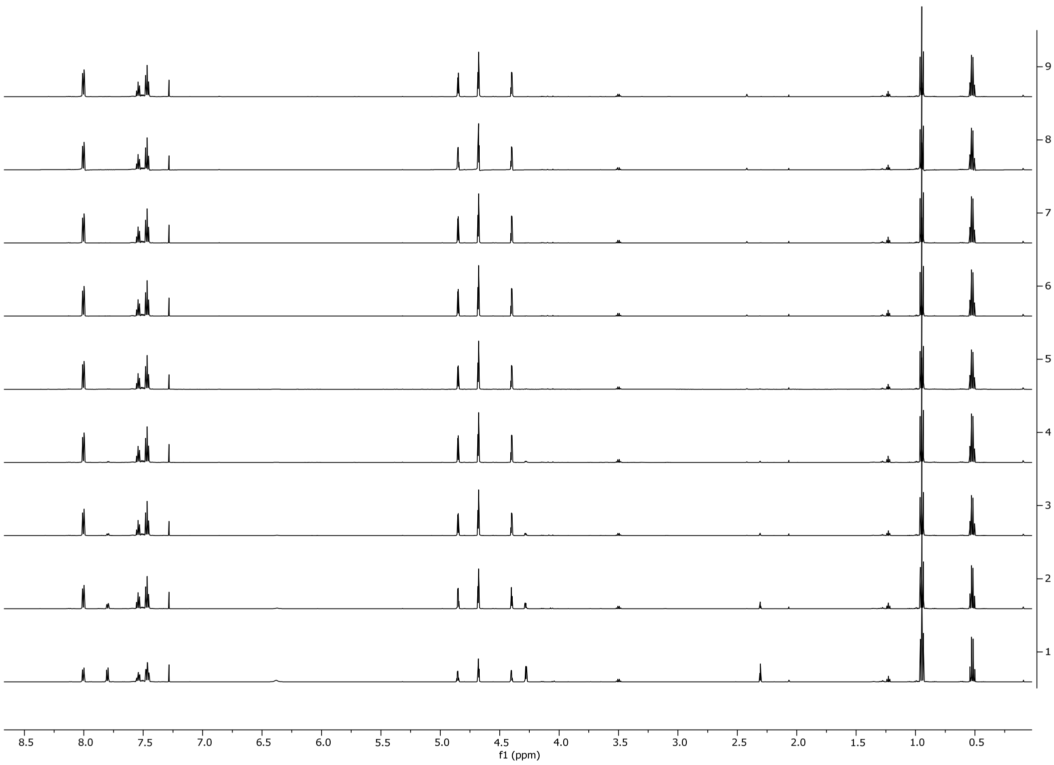

*Figure S75: 600 MHz ^{1}H-NMR spectra for the gold(I)-catalyzed cyclization of propargylic amide over the course of 10 h employing 2-methylbenzo[d]imidazo[5,1-b][1,3]iodazole-2,4-diium bis(tetrakis(pentafluorophenyl)borate) dietherate complex (**10b**) as the XB-donor.*

Diels-Alder-Reaction between CPD and MVK

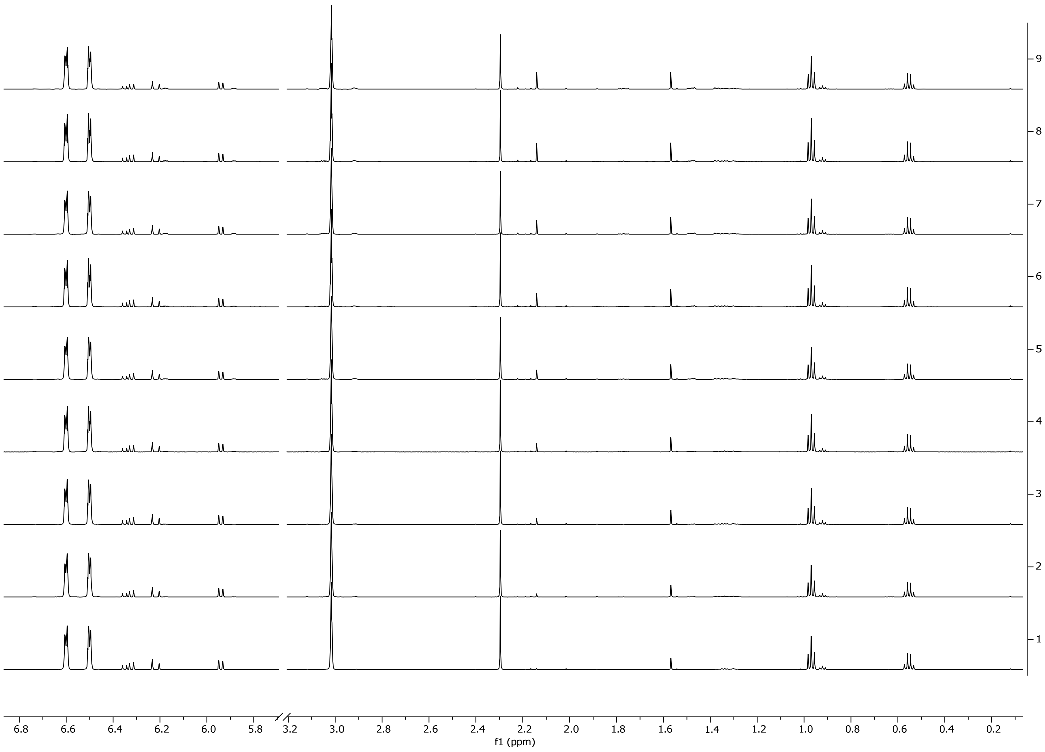

*Figure S76: 600 MHz ^{1}H-NMR spectra for the Diels-Alder-reaction between CPD and MVK over the course of 170 min employing 1-phenyl-1H-benzo[4,5]iodolo[3,2-c]pyrazol-4-ium tetrakis(3,5-bis(trifluoromethyl)phenyl)borate (**7d**) as the XB-donor.*

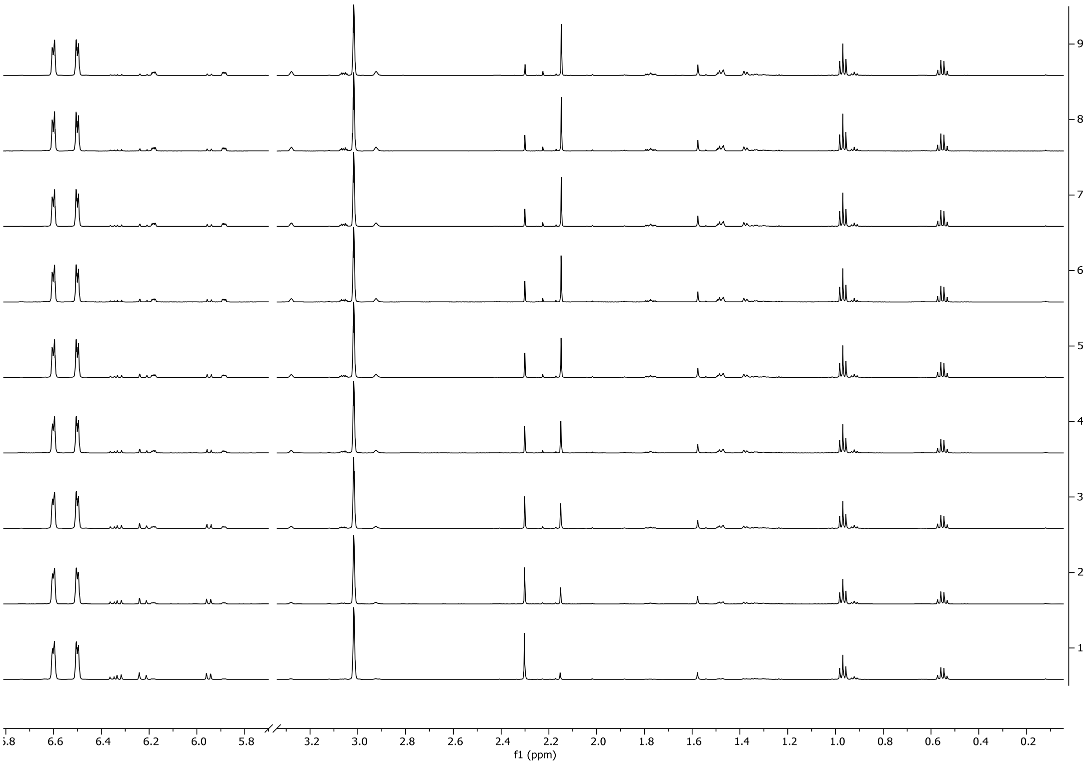

*Figure S77: 600 MHz ^{1}H-NMR spectra for the Diels-Alder-reaction between CPD and MVK over the course of 170 min employing benzo[d]pyrazolo[5,1-b][1,3]iodazol-4-ium tetrakis(3,5-bis(trifluoromethyl)phenyl)borate (**8b**) as the XB-donor.*

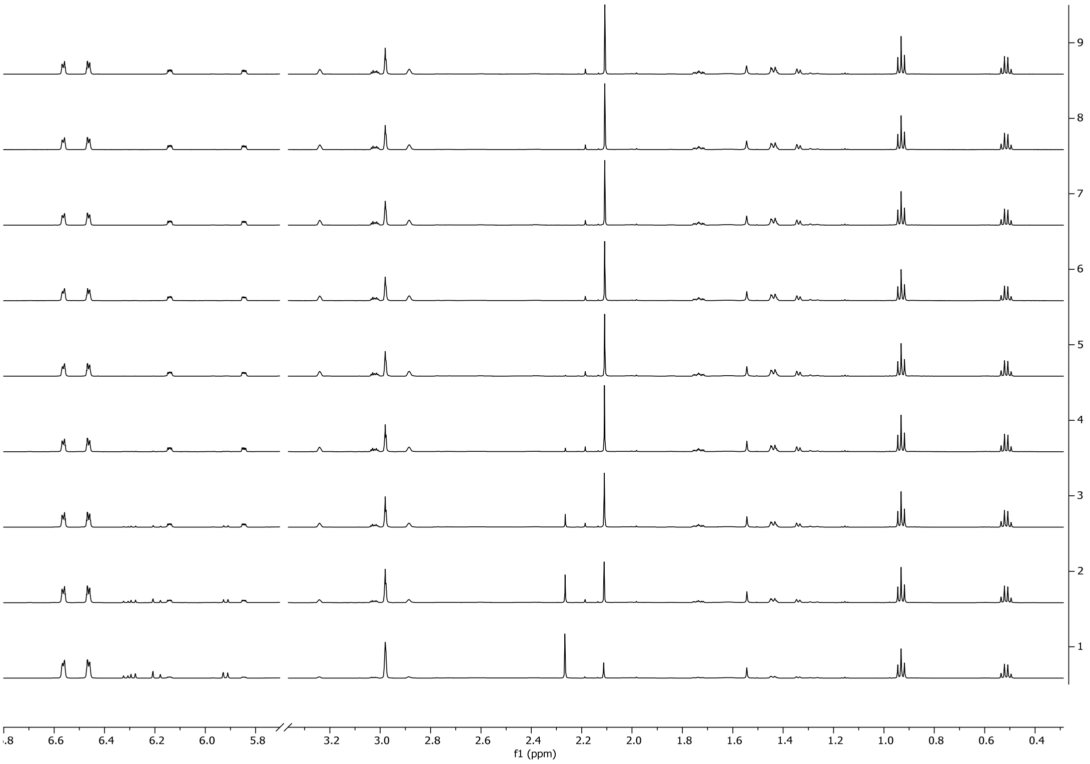

*Figure S78: 600 MHz ^{1}H-NMR spectra for the Diels-Alder-reaction between CPD and MVK over the course of 170 min employing 2-methyl-1-phenyl-1H-benzo[4,5]iodolo[3,2-c]pyrazole-2,4-diium bis(tetrakis(3,5-bis(trifluoromethyl)phenyl)borate) • Et_2O (**7f**) as the XB-donor.*

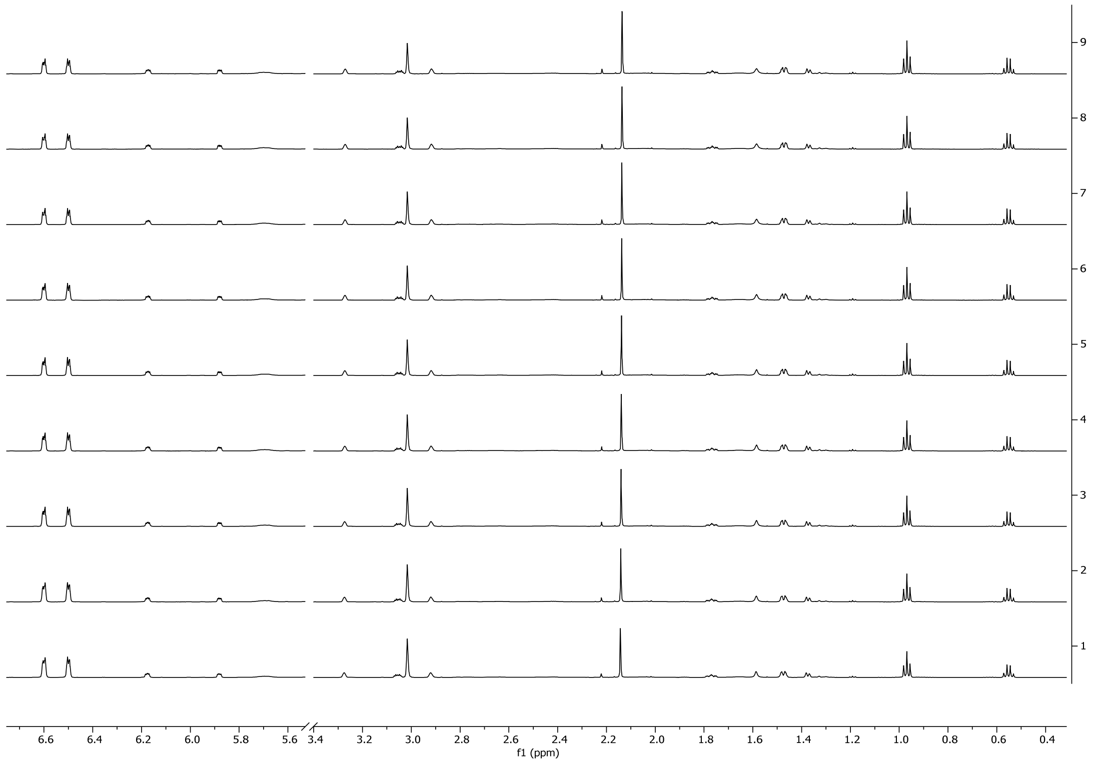

*Figure S79: 600 MHz ^{1}H-NMR spectra for the Diels-Alder-reaction between CPD and MVK over the course of 170 min employing 11-methylbenzo[4',5']iodolo[3',2':4,5]imidazo[1,2-a]pyridine-5,11-diium bis(tetrakis(3,5-bis(trifluoromethyl)phenyl)borate) • Et_2O (**11c**) as the XB-donor.*

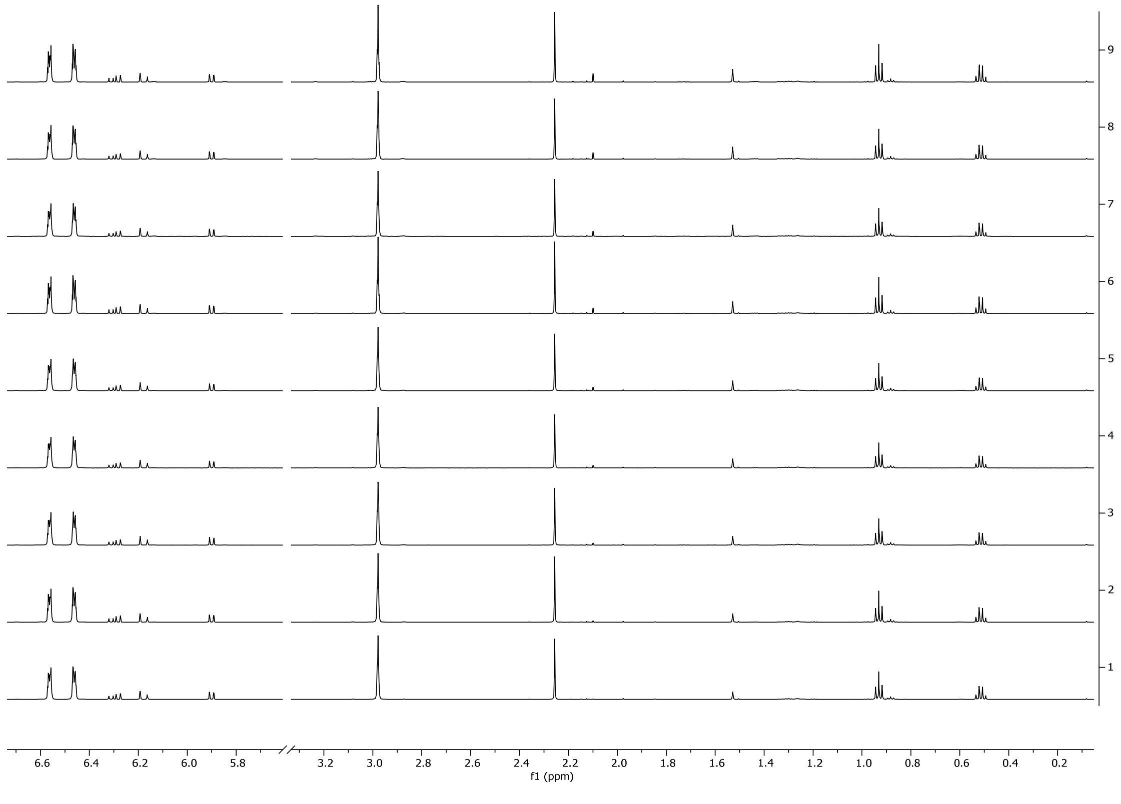

*Figure S80: 600 MHz ^{1}H-NMR spectra for the Diels-Alder-reaction between CPD and MVK over the course of 170 min employing dibenzo[b,d]iodol-5-ium tetrakis(3,5-bis(trifluoromethyl)phenyl)borate (**4b**) as the XB-donor.*

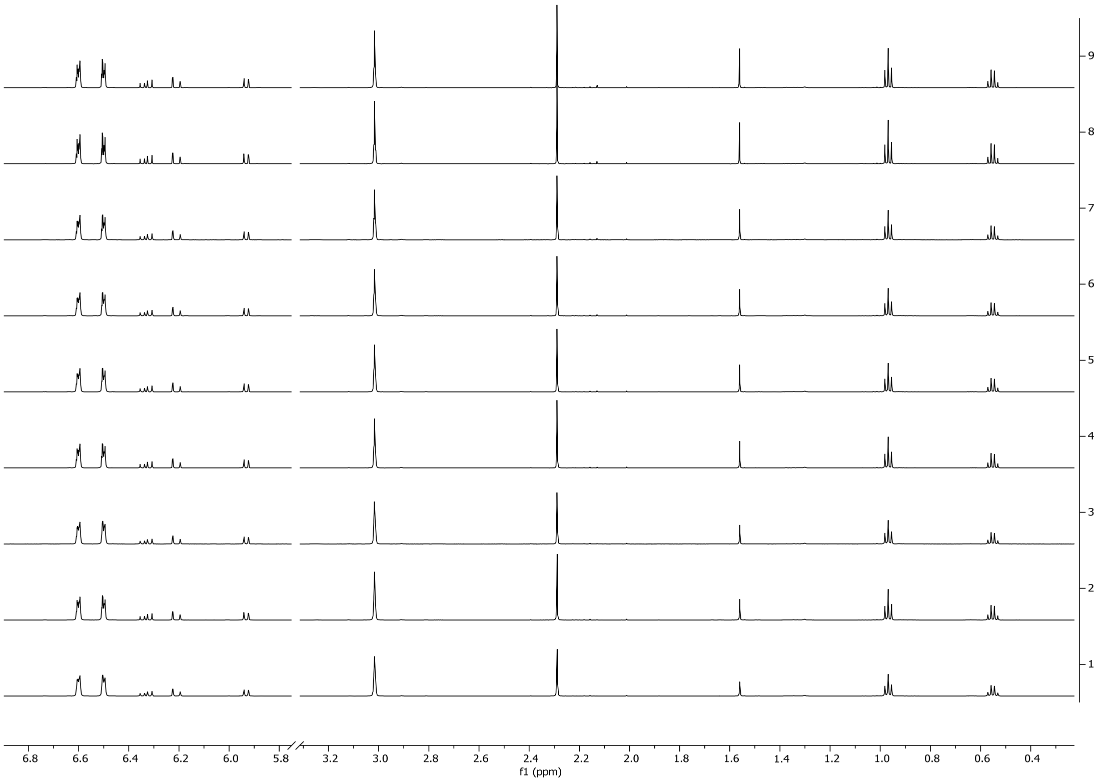

Figure S81: 600 MHz ¹H-NMR spectra for the Diels-Alder-reaction between CPD and MVK over the course of 170 min without any catalyst.

Diels-Alder-Reaction between CHD and MVK (15 mol%)

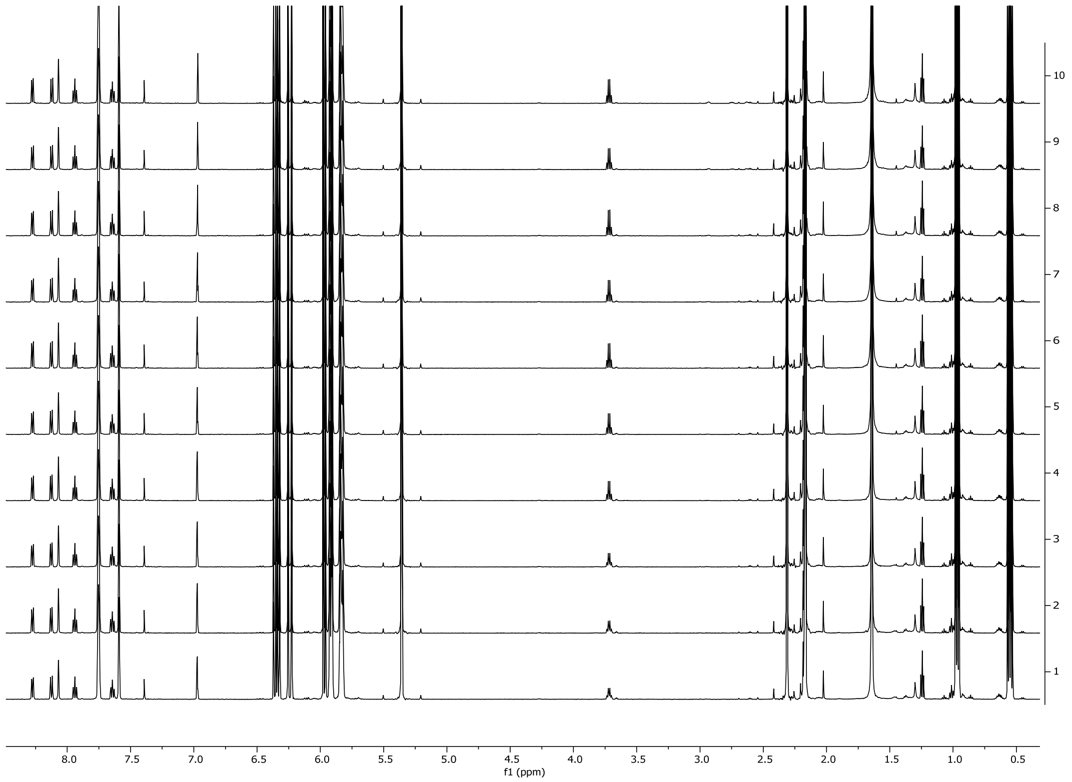

*Figure S82: 600 MHz ¹H-NMR spectra for the Diels-Alder-reaction between CHD and MVK over the course of 12 h employing benzo[d]pyrazolo[5,1-b][1,3]iodazol-4-ium tetrakis(3,5-bis(trifluoromethyl)phenyl)borate (**8b**) as the XB-donor.*

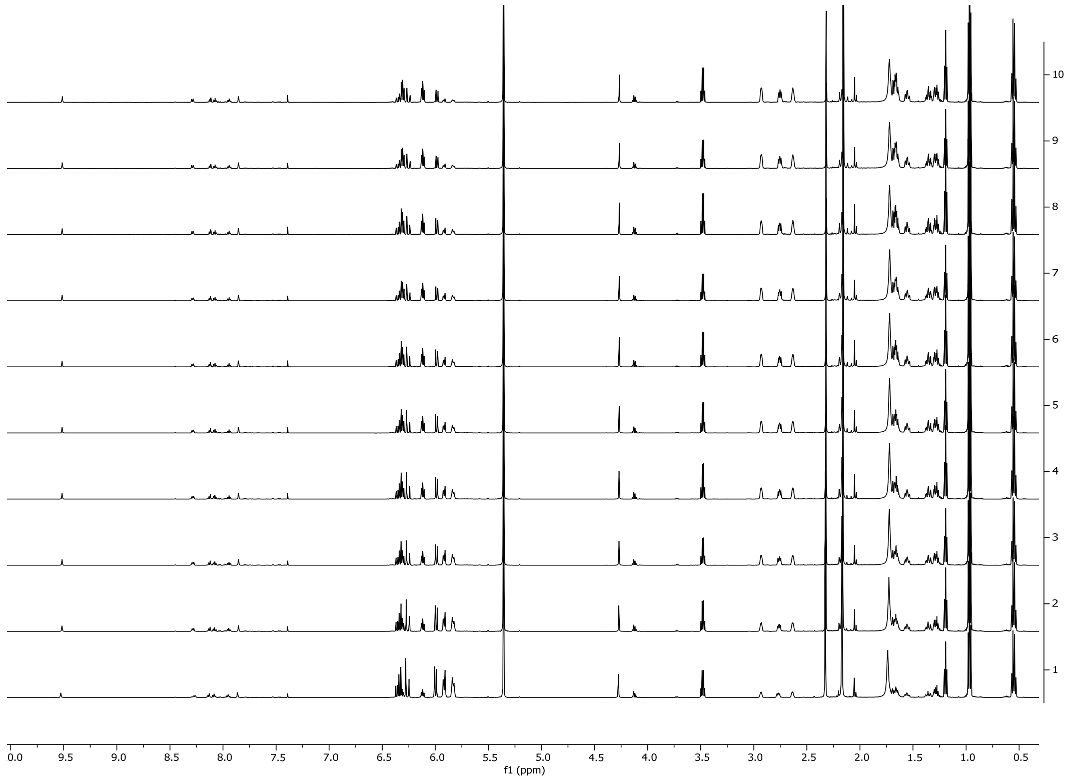

*Figure S83: 600 MHz ^{1}H-NMR spectra for the Diels-Alder-reaction between CHD and MVK over the course of 12 h employing 2-methylbenzo[d]imidazo[5,1-b][1,3]iodazole-2,4-diium bis(tetrakis(pentafluorophenyl)borate) dietherate complex (**10b**) as the XB-donor.*

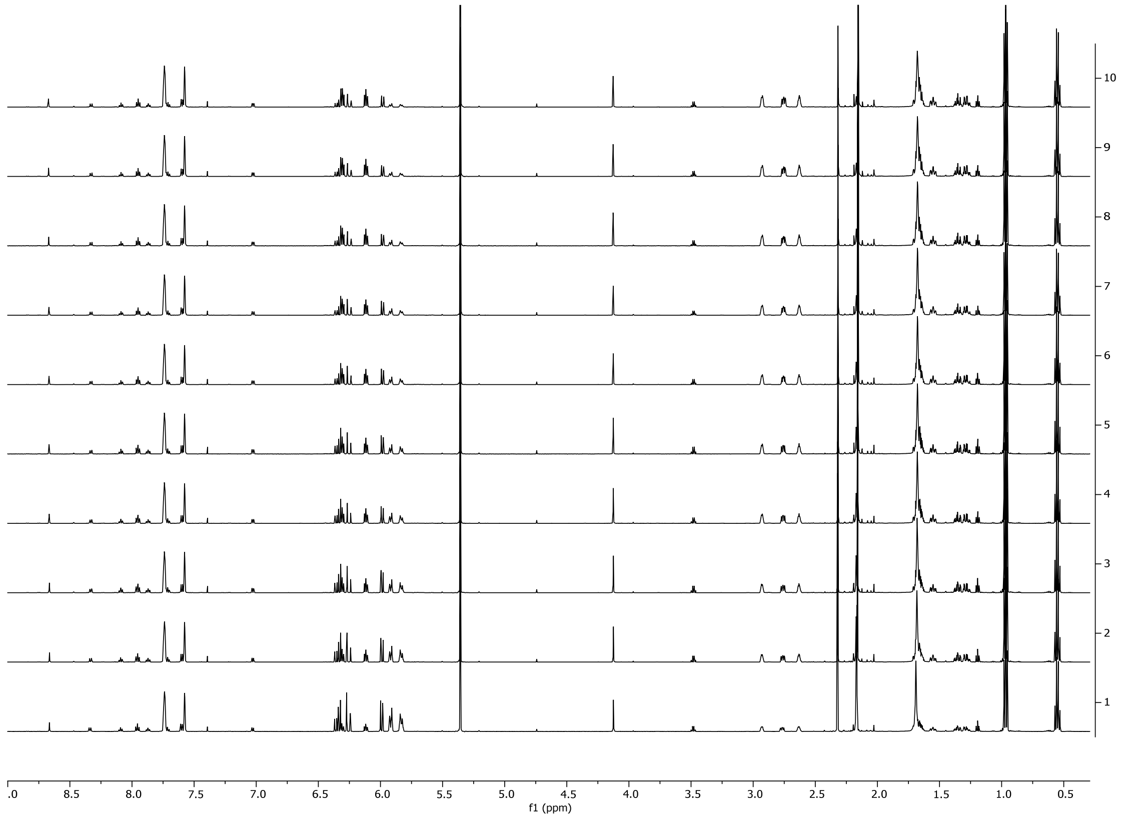

*Figure S84: 600 MHz ^{1}H-NMR spectra for the Diels-Alder-reaction between CHD and MVK over the course of 12 h employing 2-methyl-1-phenyl-1H-benzo[4,5]iodolo[3,2-c]pyrazole-2,4-diium bis(tetrakis(3,5-bis(trifluoromethyl)phenyl)borate) • Et_2O (**7f**) as the XB-donor.*

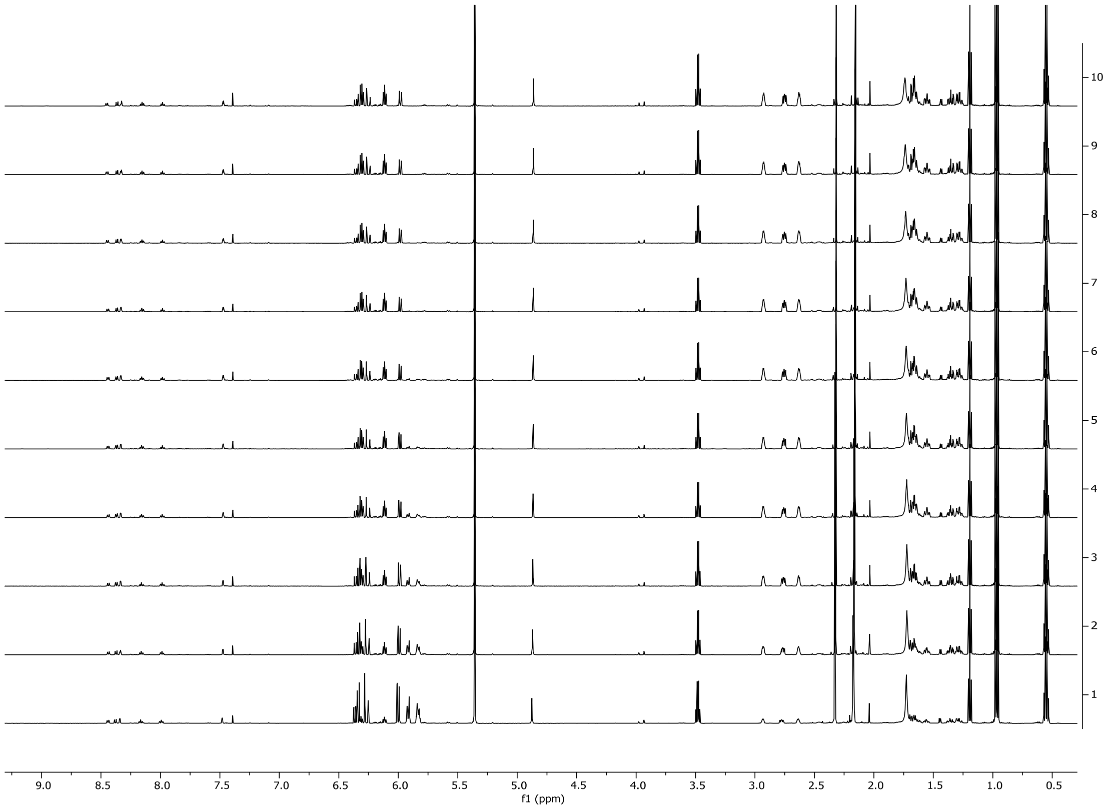

*Figure S85: 600 MHz ¹H-NMR spectra for the Diels-Alder-reaction between CHD and MVK over the course of 12 h employing 1-methylbenzo[d]pyrazolo[5,1-b][1,3]iodazole-1,4-diium bis(tetrakis(pentafluorophenyl)borate) dietherate complex (**8d**) as the XB-donor.*

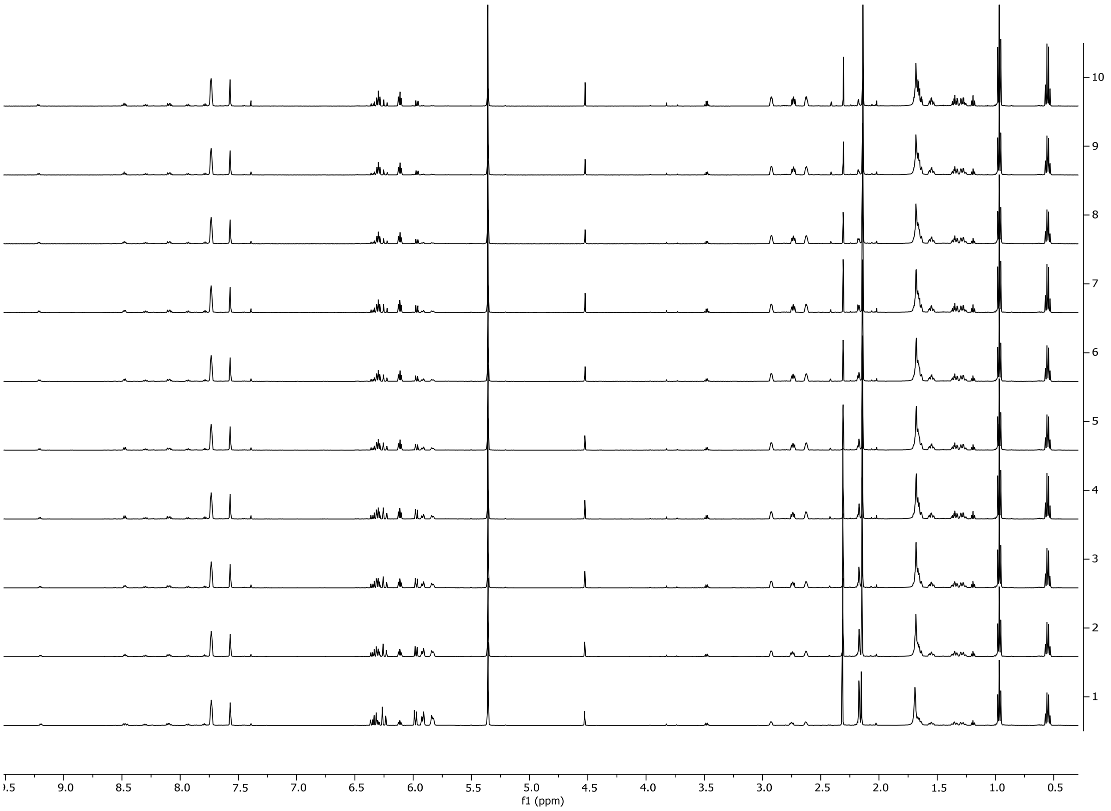

*Figure S86: 600 MHz ¹H-NMR spectra for the Diels-Alder-reaction between CHD and MVK over the course of 12 h employing 11-methylbenzo[4',5']iodolo[3',2':4,5]imidazo[1,2-a]pyridine-5,11-diium bis(tetrakis(3,5-bis(trifluoro-methyl)phenyl)borate) • Et_2O (**11c**) as the XB-donor.*

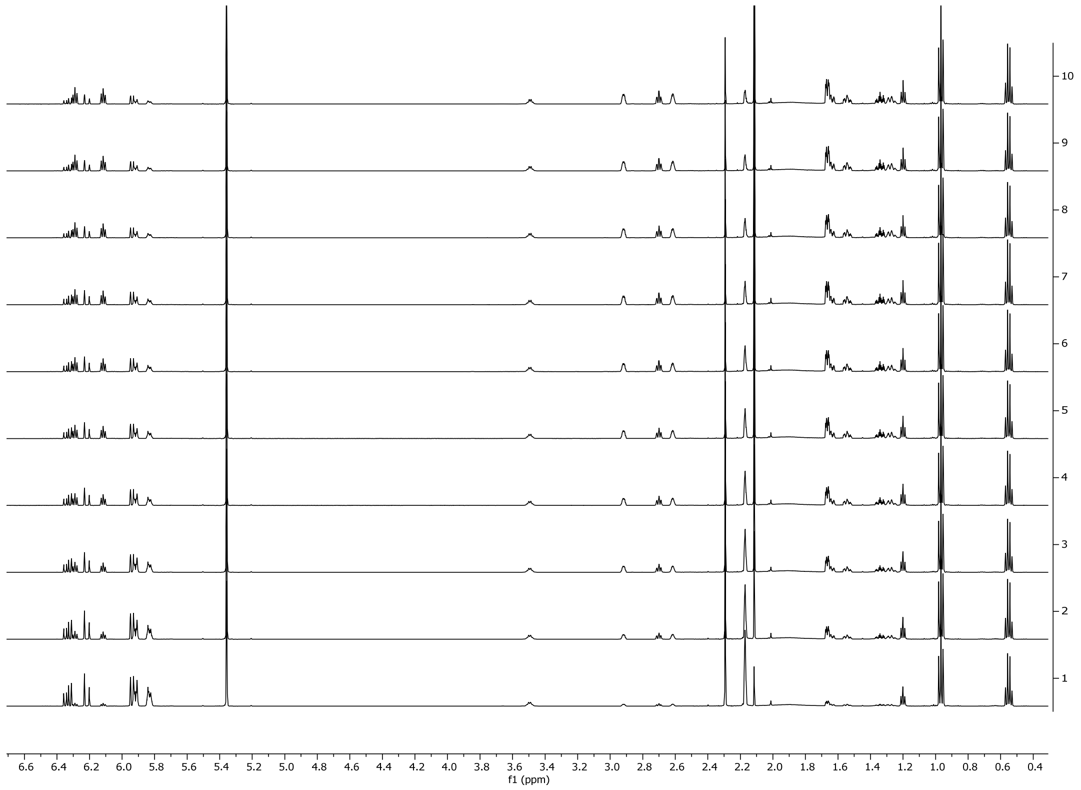

Figure S87: 600 MHz ^{1}H-NMR spectra for the Diels-Alder-reaction between CHD and MVK over the course of 12 h employing BF_3 etherate as the catalyst.

Diels-Alder-Reaction between CHD and MVK (2.5-5 mol%)

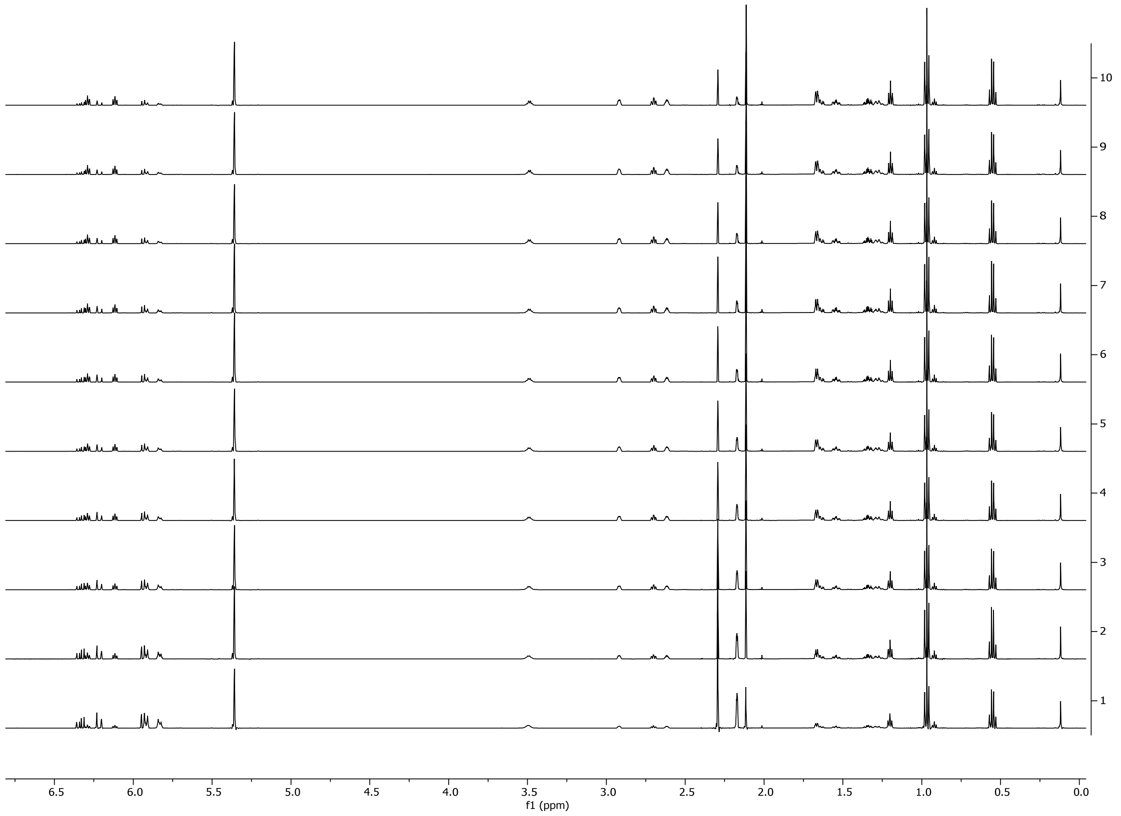

Figure S88: 600 MHz ^{1}H-NMR spectra for the Diels-Alder-reaction between CHD and MVK over the course of 12 h employing BF_3 etherate as the catalyst.

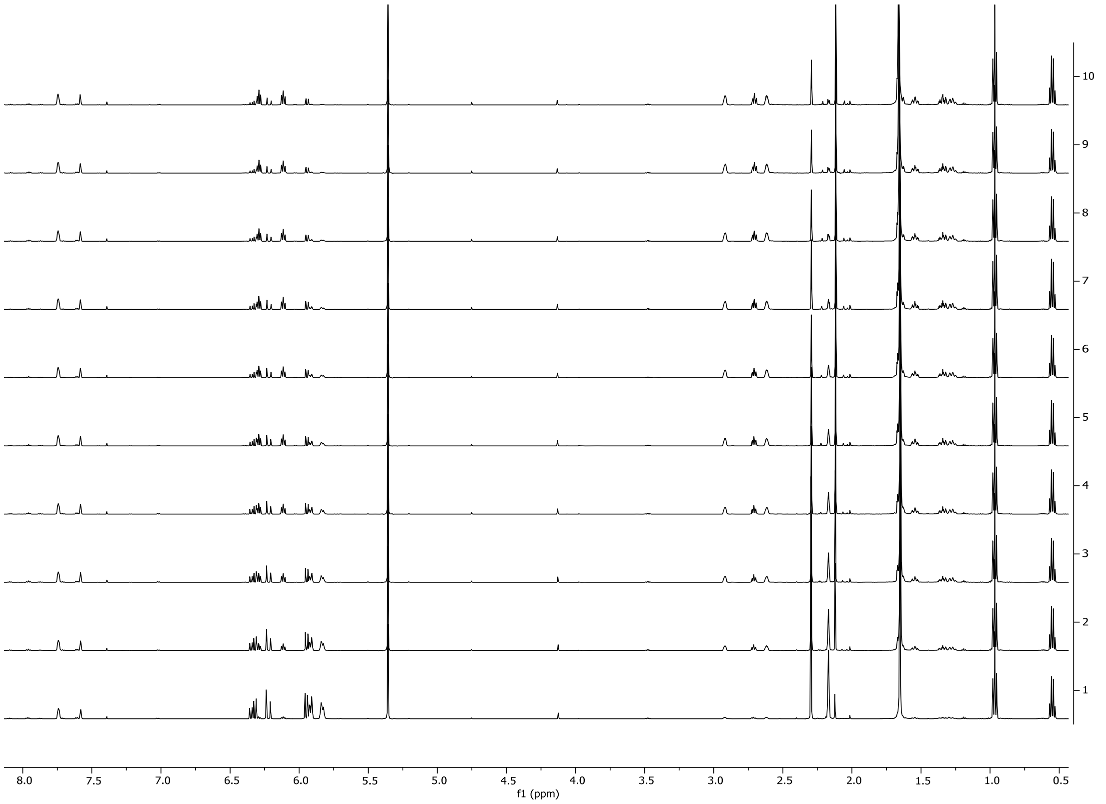

*Figure S89: 600 MHz ¹H-NMR spectra for the Diels-Alder-reaction between CHD and MVK over the course of 12 h employing 2-methyl-1-phenyl-1H-benzo[4,5]iodolo[3,2-c]pyrazole-2,4-diium bis(tetrakis(3,5-bis(trifluoromethyl)phenyl)borate) • Et_2O (**7f**) as the XB-donor.*

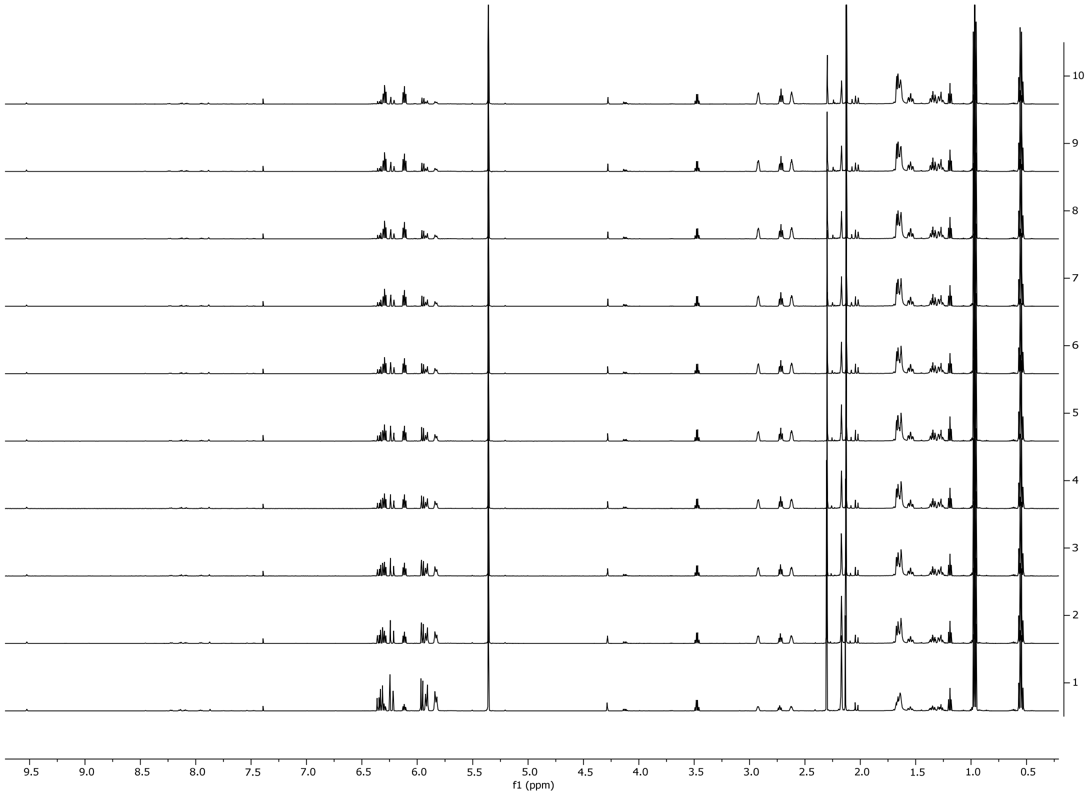

*Figure S90: 600 MHz ¹H-NMR spectra for the Diels-Alder-reaction between CHD and MVK over the course of 12 h employing 2-methylbenzo[d]imidazo[5,1-b][1,3]iodazole-2,4-diium bis(tetrakis(pentafluorophenyl)borate) dietherate complex (**10b**) as the XB-donor.*

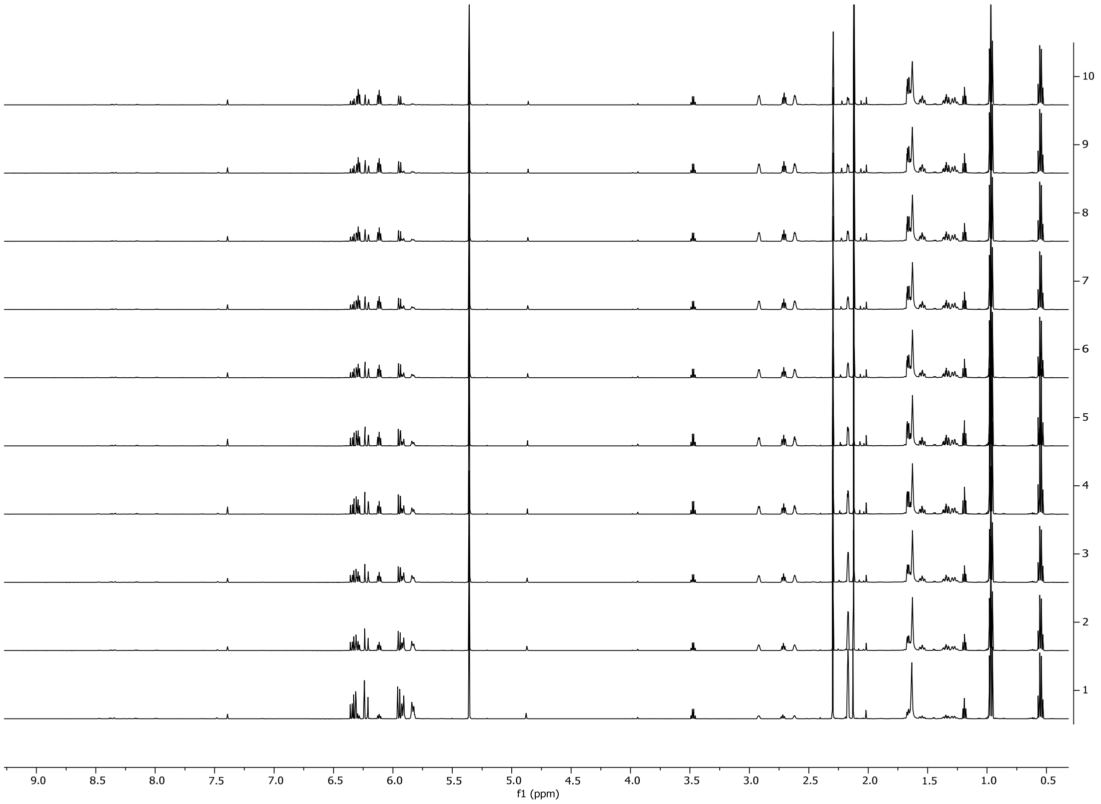

*Figure S91: 600 MHz ^{1}H-NMR spectra for the Diels-Alder-reaction between CHD and MVK over the course of 12 h employing 1-methylbenzo[d]pyrazolo[5,1-b][1,3]iodazole-1,4-diium bis(tetrakis(pentafluorophenyl)borate) dietherate complex (**8d**) as the XB-donor.*

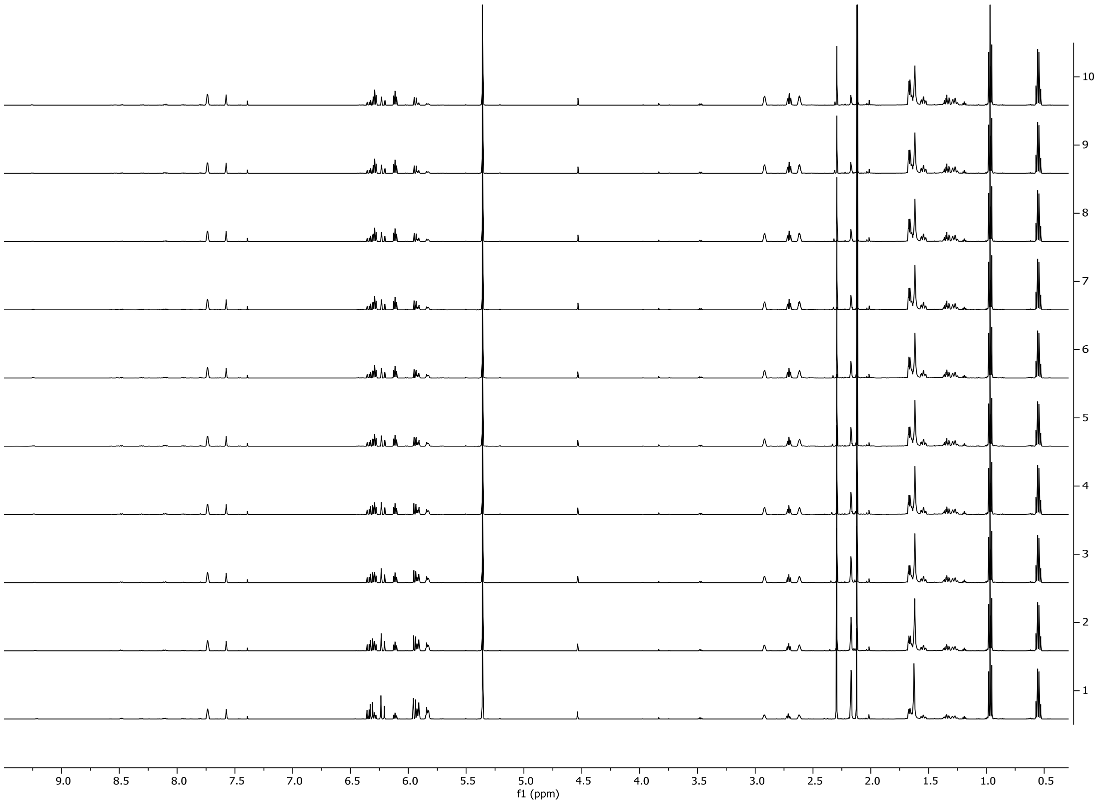

*Figure S92: 600 MHz ^{1}H-NMR spectra for the Diels-Alder-reaction between CHD and MVK over the course of 12 h employing 11-methylbenzo[4',5']iodolo[3',2':4,5]imidazo[1,2-a]pyridine-5,11-diium bis(tetrakis(3,5-bis(trifluoromethyl)phenyl)borate) • Et_2O (**11c**) as the XB-donor.*

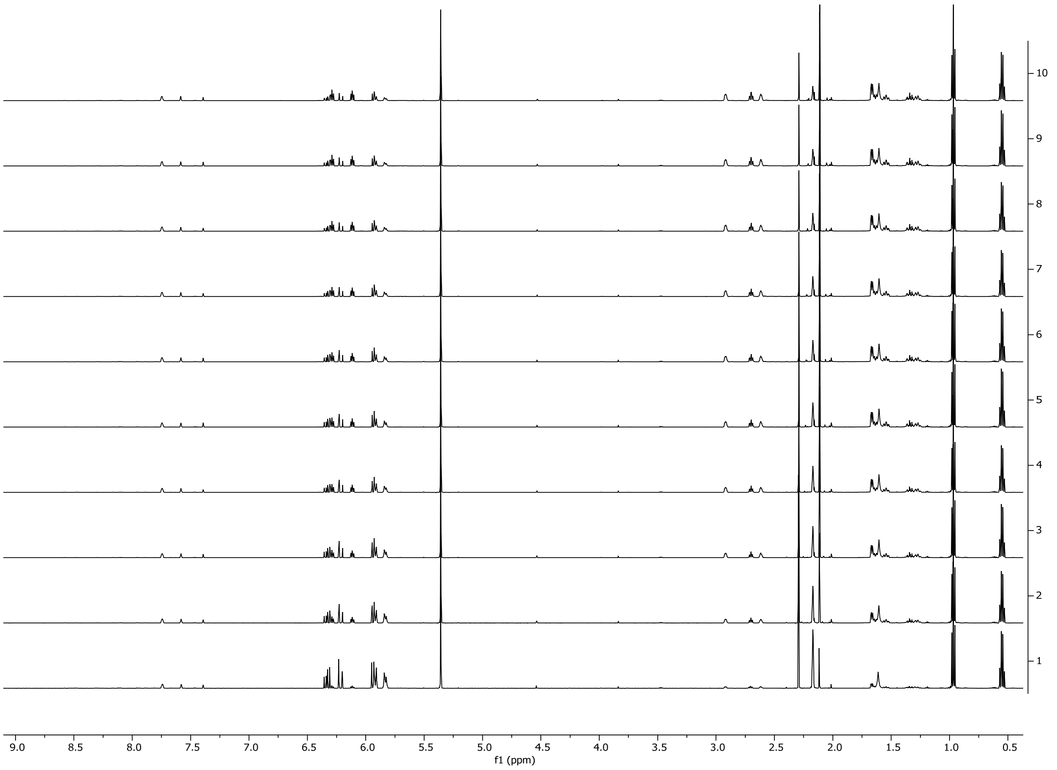

*Figure S93: 600 MHz ^{1}H-NMR spectra for the Diels-Alder-reaction between CHD and MVK over the course of 12 h employing 2.5 mol% of 11-methylbenzo[4',5']iodolo[3',2':4,5]imidazo[1,2-a]pyridine-5,11-diium bis(tetrakis(3,5-bis(trifluoromethyl)phenyl)borate) • Et_2O (**11c**) as the XB-donor.*

Michael addition between 1-methylindole and crotonophenone

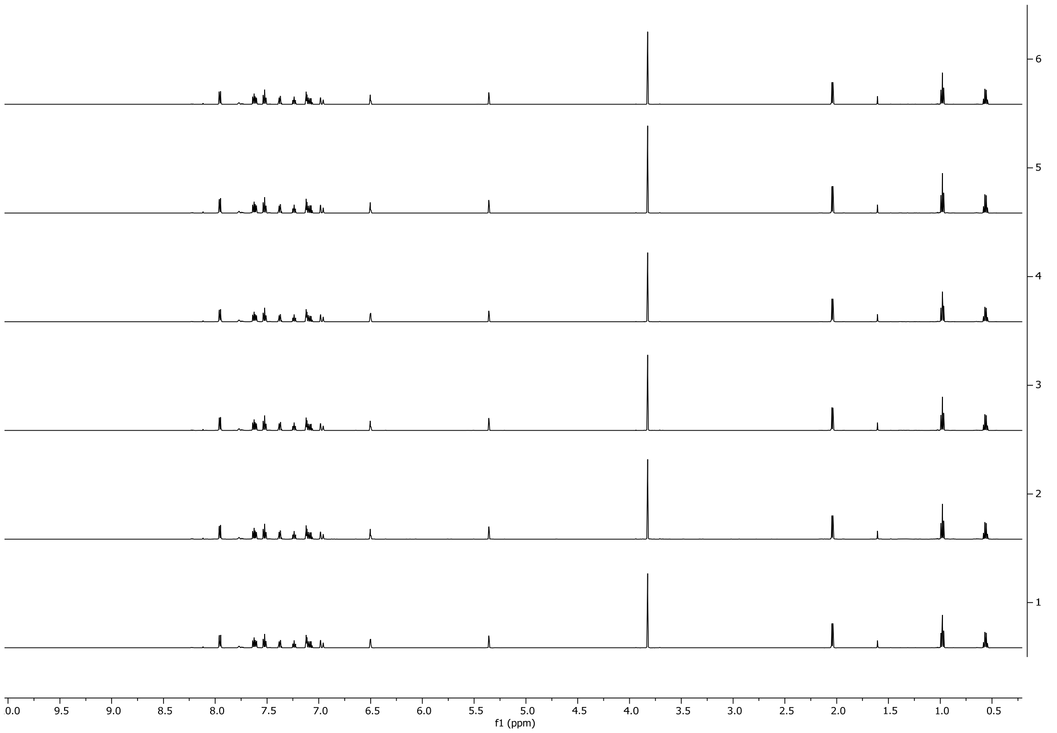

*Figure S94: 600 MHz ^{1}H-NMR spectra for the Michael addition between 1-methylindole and crotonophenone over the course of 4.75 h employing 1-phenyl-1H-benzo[4,5]iodolo[3,2-c]pyrazol-4-ium tetrakis(3,5-bis(trifluoromethyl)phenyl)borate (**7d**) as the XB-donor.*

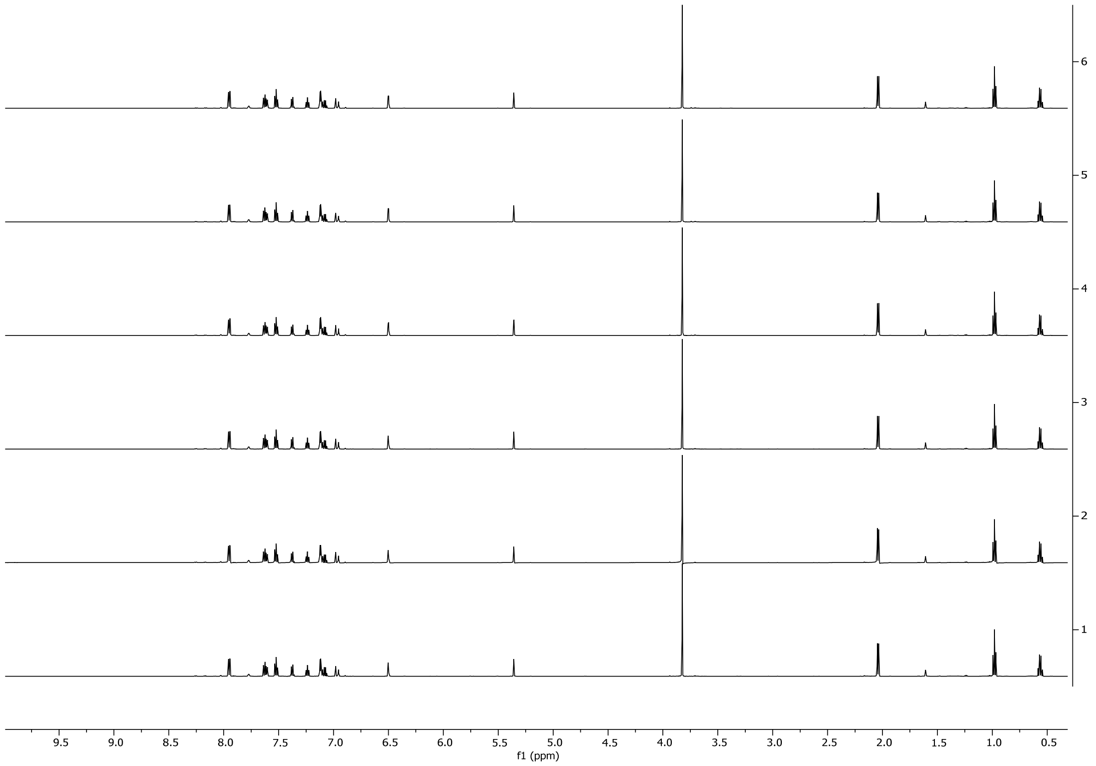

*Figure S95: 600 MHz ^{1}H-NMR spectra for the Michael addition between 1-methylindole and crotonophenone over the course of 4.75 h employing benzo[d]pyrazolo[5,1-b][1,3]iodazol-4-ium tetrakis(3,5-bis(trifluoromethyl)phenyl)borate (**8b**) as the XB-donor.*

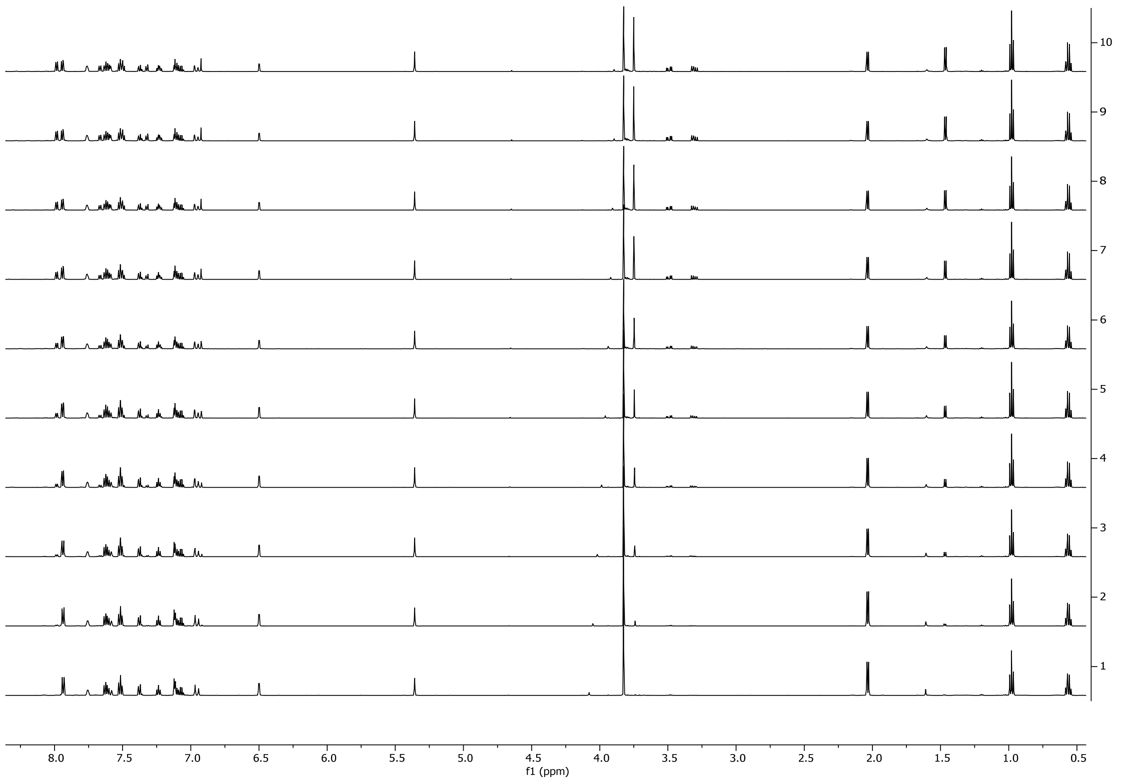

*Figure 96: 600 MHz ^{1}H-NMR spectra for the Michael addition between 1-methylindole and crotonophenone over the course of 4.75 h employing 2-methyl-1-phenyl-1H-benzo[4,5]iodolo[3,2-c]pyrazole-2,4-diium bis(tetrakis(3,5-bis(trifluoromethyl)phenyl)borate) • Et_2O (**7f**) as the XB-donor.*

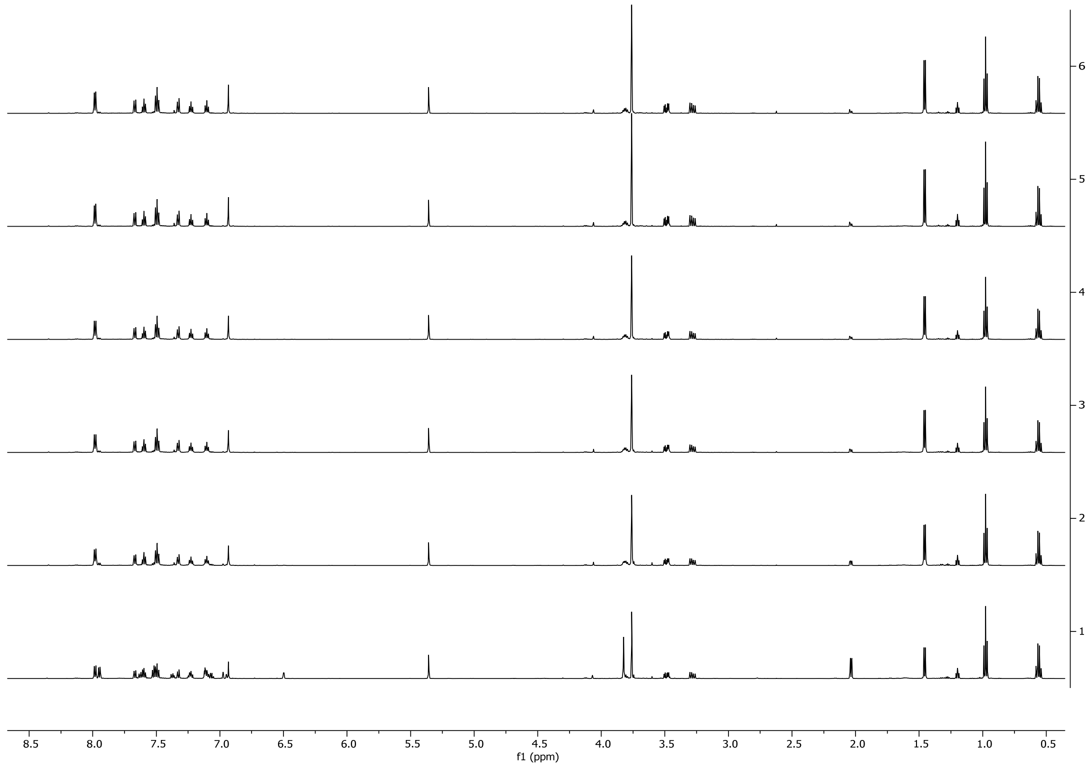

*Figure S97: 600 MHz ^{1}H-NMR spectra for the Michael addition between 1-methylindole and crotonophenone over the course of 2.25 h employing 2-methylbenzo[d]imidazo[5,1-b][1,3]iodazole-2,4-diium bis(tetrakis(pentafluoro-phenyl)borate) dietherate complex (**10b**) as the XB-donor.*

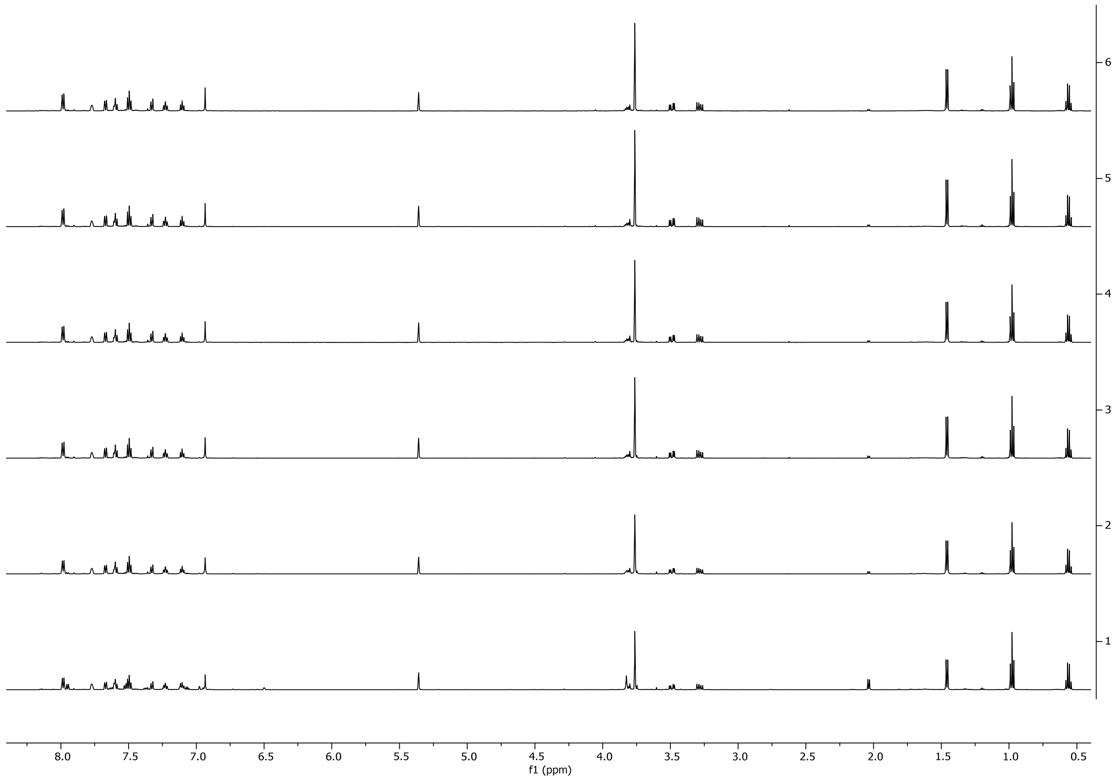

*Figure S98: 600 MHz ^{1}H-NMR spectra for the Michael addition between 1-methylindole and crotonophenone over the course of 2.25 h employing 11-methylbenzo[4',5']iodolo[3',2':4,5]imidazo[1,2-a]pyridine-5,11-diium bis(tetrakis(3,5-bis(trifluoromethyl)phenyl)borate) • Et_2O (**11c**) as the XB-donor.*

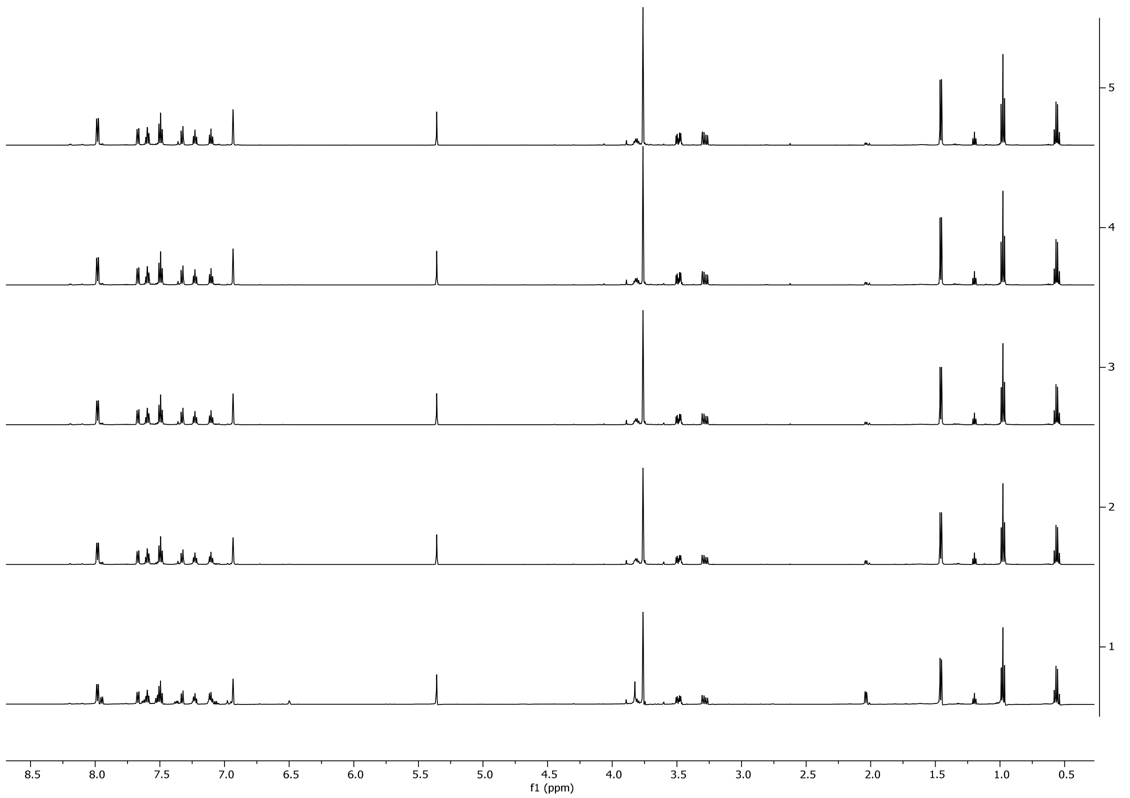

*Figure S99: 600 MHz ^{1}H-NMR spectra for the Michael addition between 1-methylindole and crotonophenone over the course of 1.75 h employing 1-methylbenzo[d]pyrazolo[5,1-b][1,3]iodazole-1,4-diium bis(tetrakis(pentafluorophenyl)borate) dietherate complex (**8d**) as the XB-donor.*

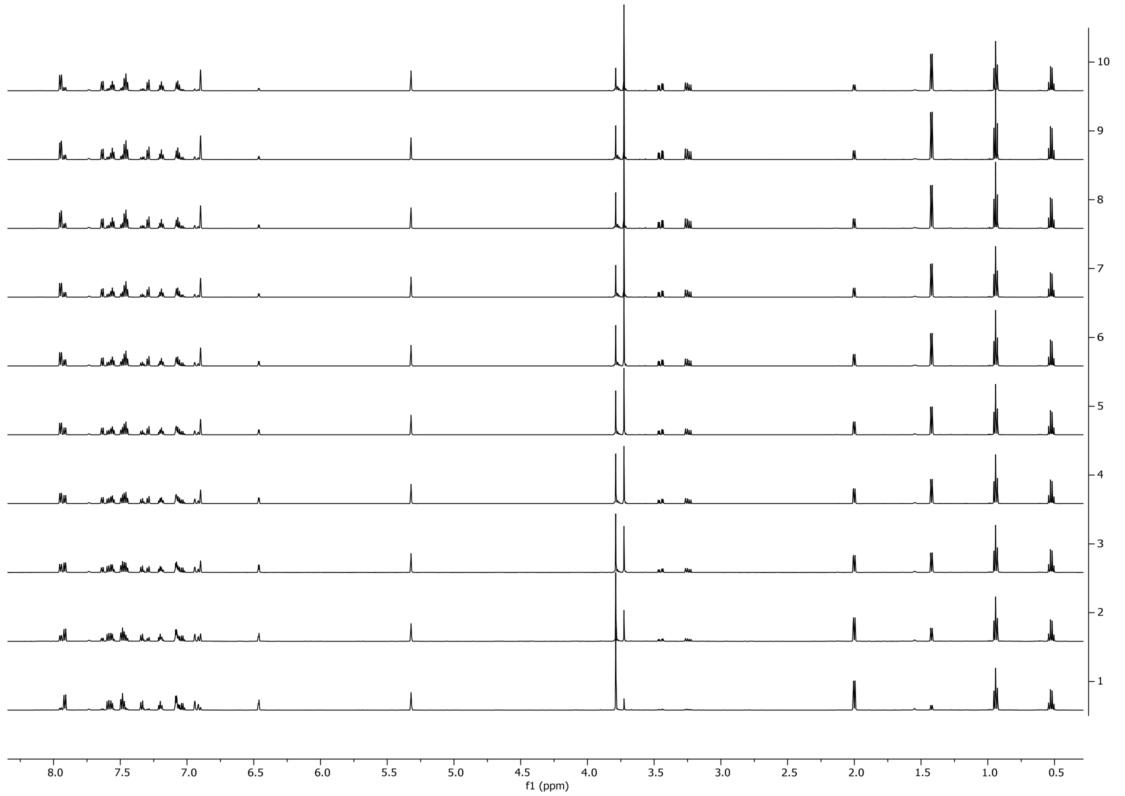

*Figure S100: 600 MHz ^{1}H-NMR spectra for the Michael addition between 1-methylindole and crotonophenone over the course of 4.75 h employing 1 mol% of 11-methylbenzo[4',5']iodolo[3',2':4,5]imidazo[1,2-a]pyridine-5,11-diium bis(tetrakis(3,5-bis(trifluoromethyl)phenyl)borate) • Et_2O (**11c**) as the XB-donor.*

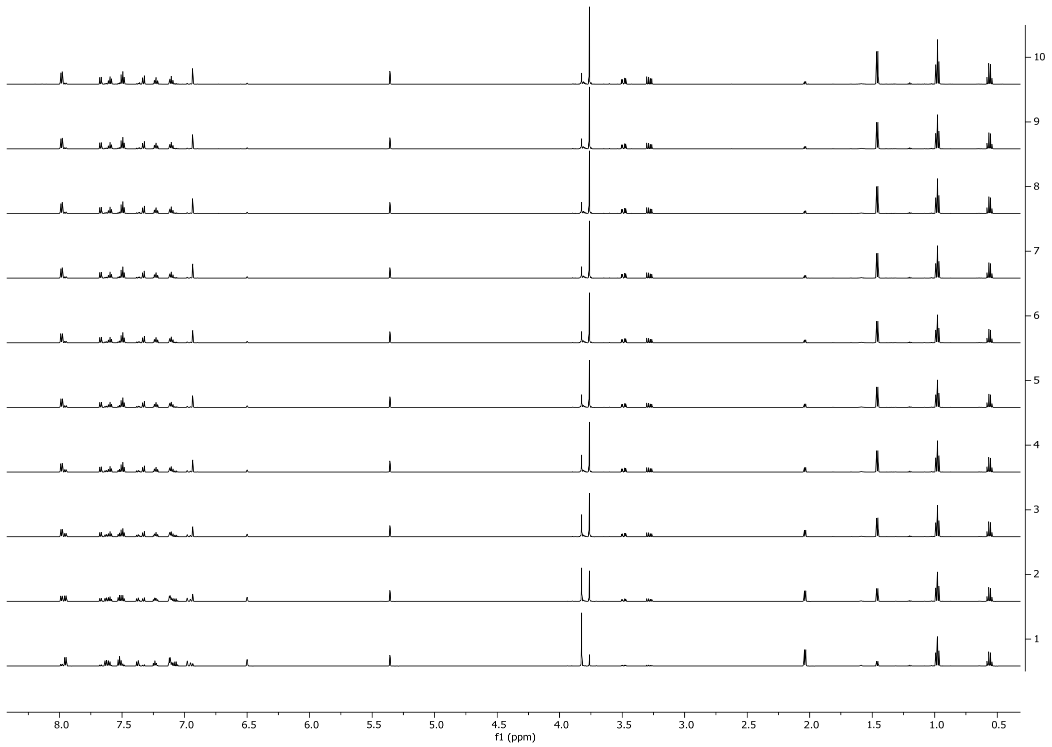

*Figure S101: 600 MHz ^{1}H-NMR spectra for the Michael addition between 1-methylindole and crotonophenone over the course of 4.75 h employing 1 mol% of 1-methylbenzo[d]pyrazolo[5,1-b][1,3]iodazole-1,4-diium bis(tetrakis(pentafluorophenyl)borate) dietherate complex (**8d**) as the XB-donor.*

Nitro-Michael reaction between 5-methoxyindole and nitrostyrene

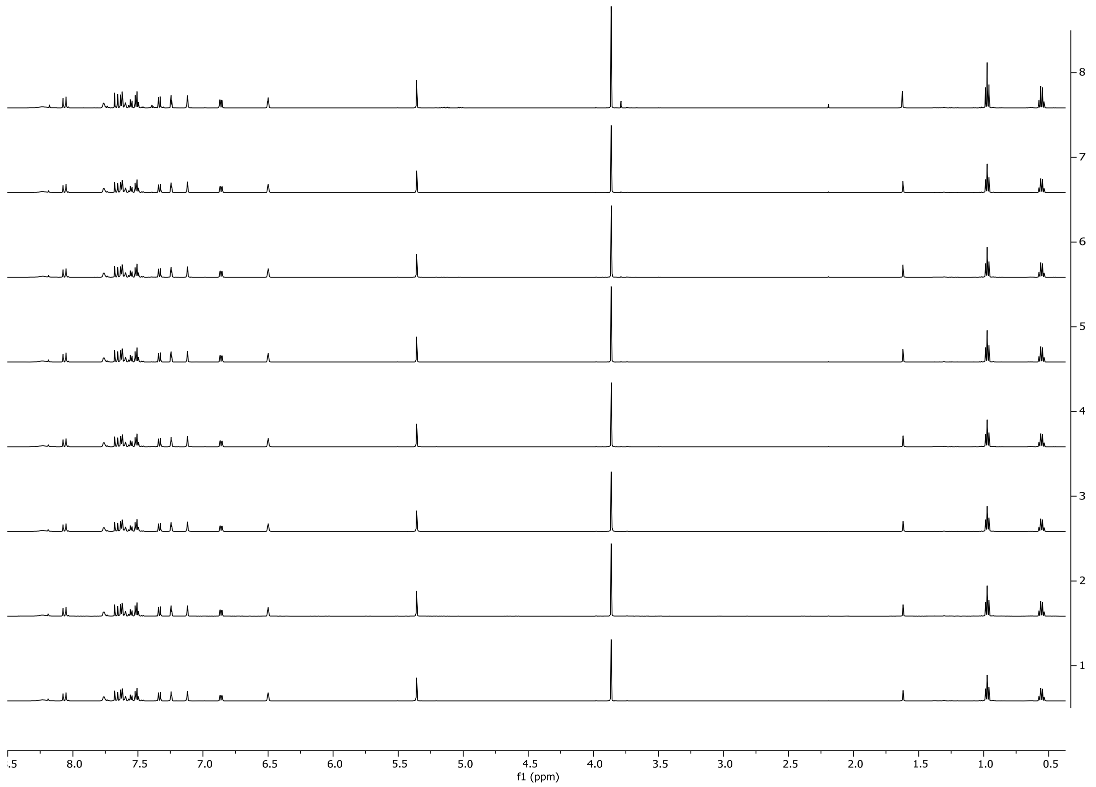

*Figure S102: 600 MHz ^{1}H-NMR spectra for the nitro-Michael addition between 5-methoxyindole and nitrostyrene over the course of 24 h employing 1-phenyl-1H-benzo[4,5]iodolo[3,2-c]pyrazol-4-ium tetrakis(3,5-bis(trifluoromethyl)phenyl)borate (**7d**) as the XB-donor.*

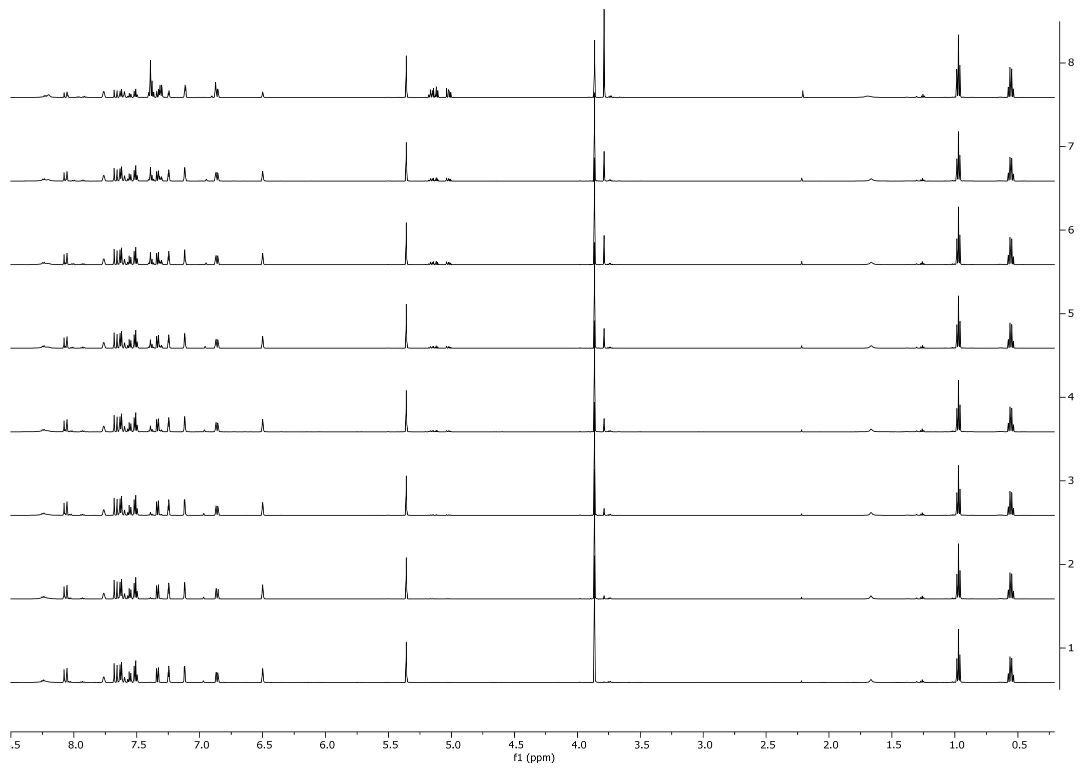

*Figure S103: 600 MHz ¹H-NMR spectra for the nitro-Michael addition between 5-methoxyindole and nitrostyrene over the course of 24 h employing benzo[d]pyrazolo[5,1-b][1,3]iodazol-4-ium tetrakis(3,5-bis(trifluoromethyl)phenyl)borate (**8b**) as the XB-donor.*

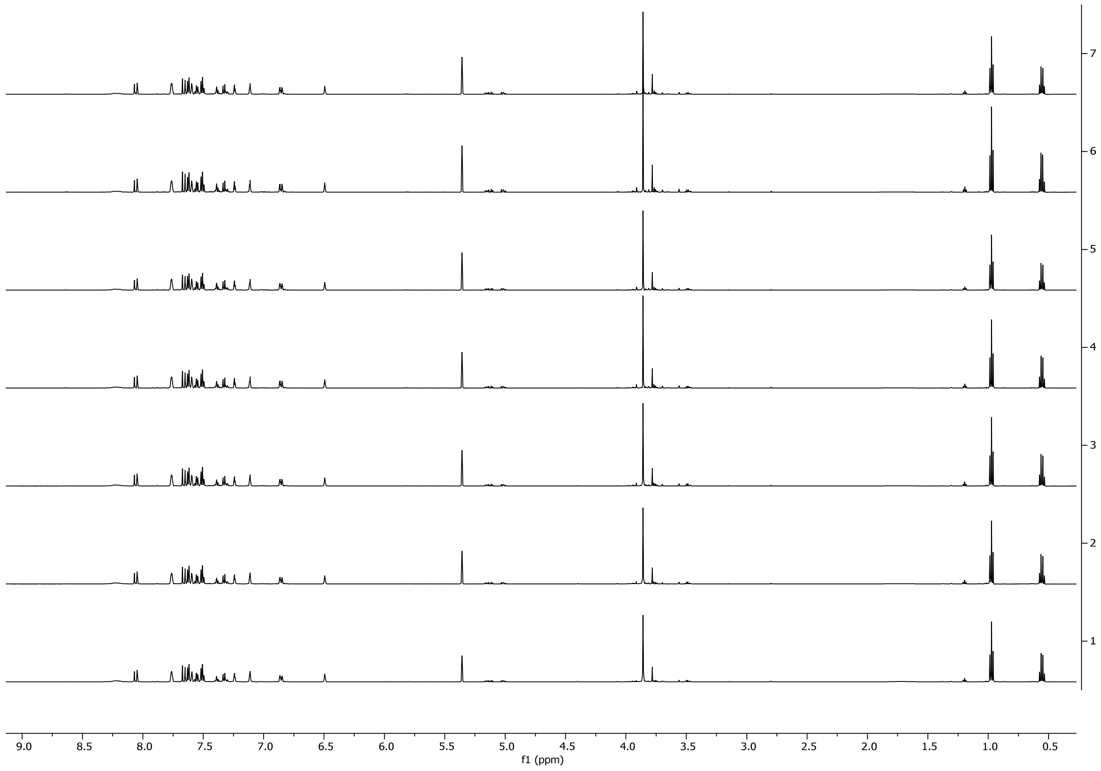

*Figure S104: 600 MHz ¹H-NMR spectra for the nitro-Michael addition between 5-methoxyindole and nitrostyrene over the course of 4.5 h employing 11-methylbenzo[4',5']iodolo[3',2':4,5]imidazo[1,2-a]pyridine-5,11-diium bis(tetrakis(3,5-bis(trifluoromethyl)phenyl)borate) • Et$_2$O (**11c**) as the XB-donor.*

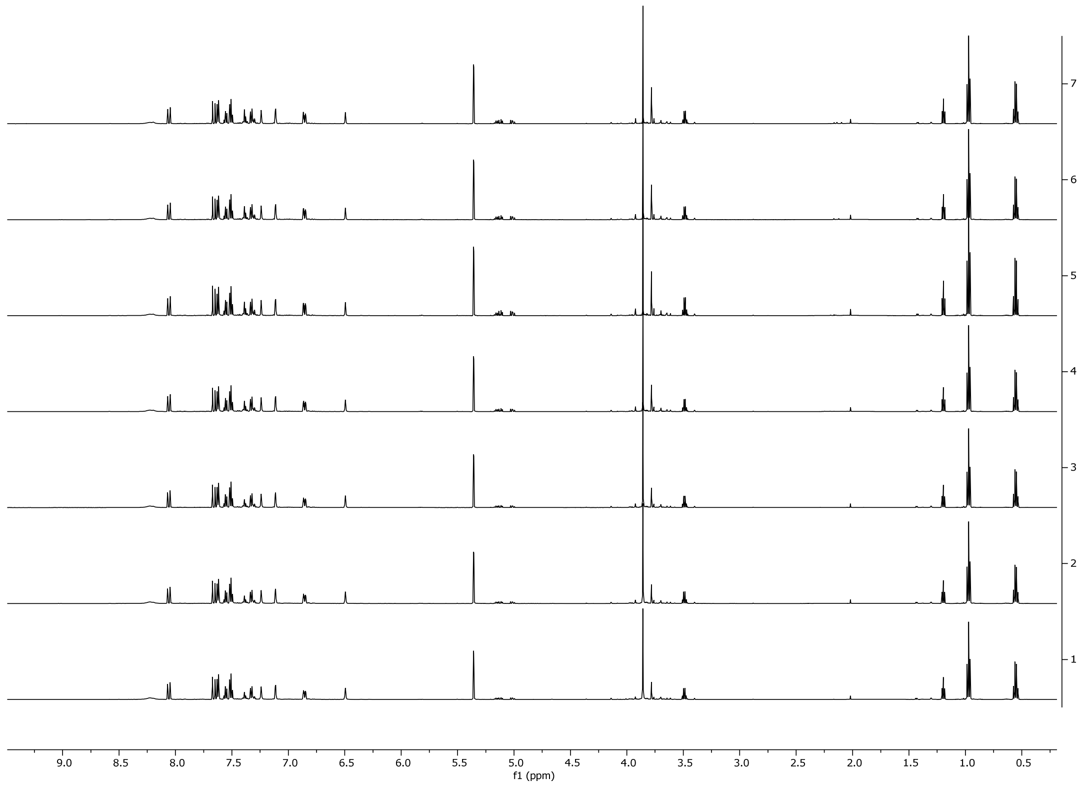

*Figure S105: 600 MHz ¹H-NMR spectra for the nitro-Michael addition between 5-methoxyindole and nitrostyrene over the course of 4.5 h employing 1-methylbenzo[d]pyrazolo[5,1-b][1,3]iodazole-1,4-diium bis(tetrakis(pentafluorophenyl)borate) dietherate complex (**8d**) as the XB-donor.*

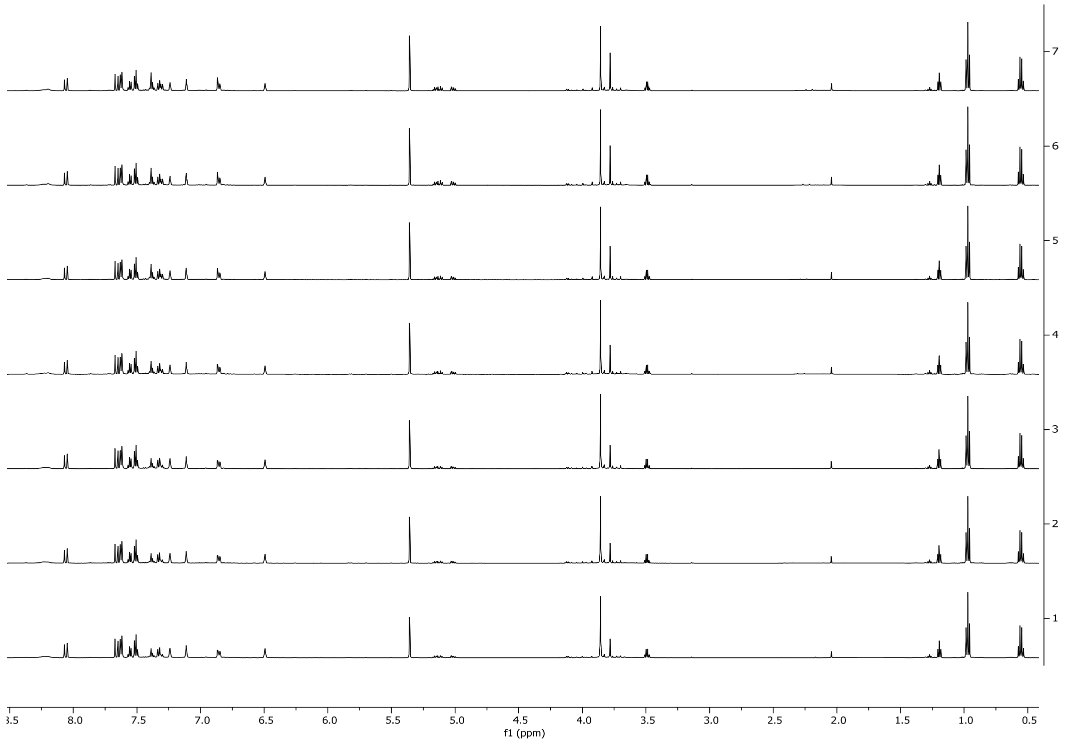

*Figure S106: 600 MHz ¹H-NMR spectra for the nitro-Michael addition between 5-methoxyindole and nitrostyrene over the course of 4.5 h employing 2-methylbenzo[d]imidazo[5,1-b][1,3]iodazole-2,4-diium bis(tetrakis(pentafluorophenyl)borate) dietherate complex (**10b**) as the XB-donor.*

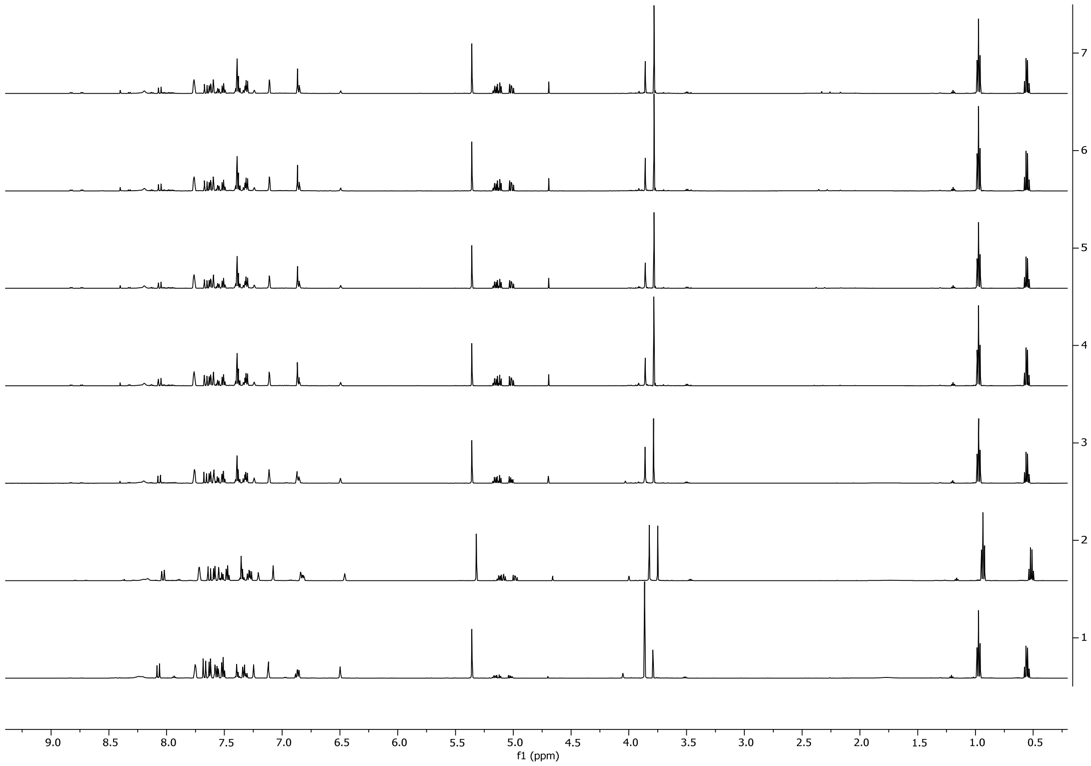

*Figure S107: 600 MHz ^{1}H-NMR spectra for the nitro-Michael addition between 5-methoxyindole and nitrostyrene over the course of 4.5 h employing 2-methyl-1-phenyl-1H-benzo[4,5]iodolo[3,2-c]pyrazole-2,4-diium bis(tetrakis(3,5-bis(trifluoromethyl)phenyl)borate) • Et_2O (**7f**) as the XB-donor.*

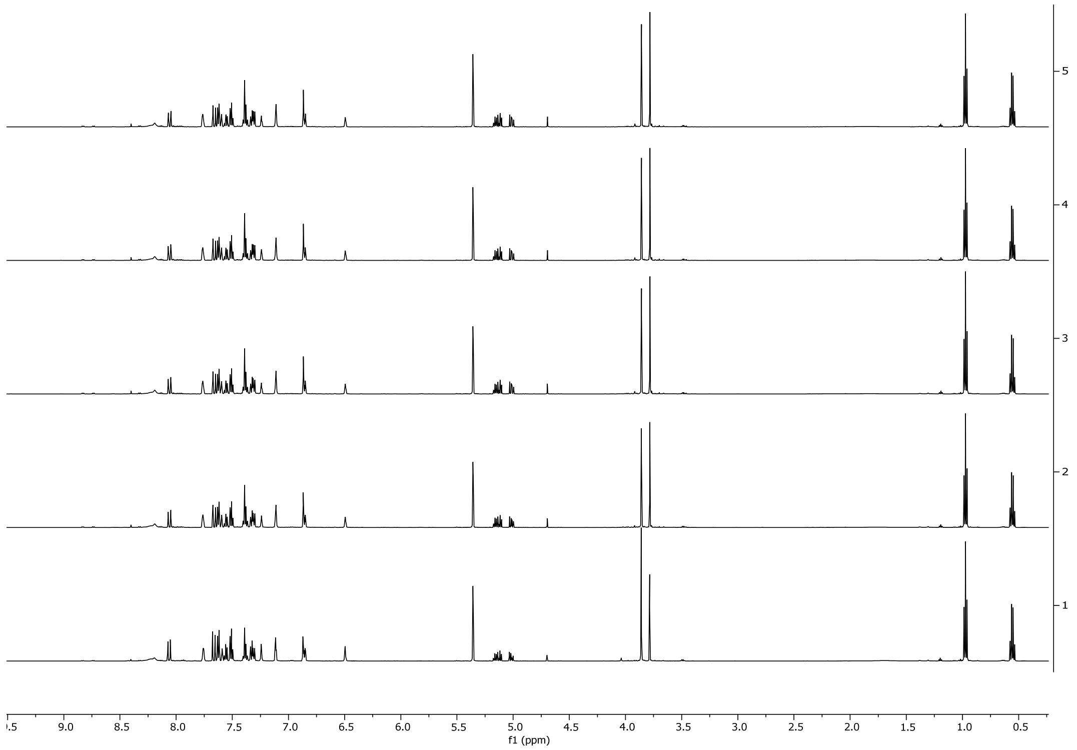

*Figure S108: 600 MHz ^{1}H-NMR spectra for the nitro-Michael addition between 5-methoxyindole and nitrostyrene over the course of 4.5 h employing 5 mol% of 2-methyl-1-phenyl-1H-benzo[4,5]iodolo[3,2-c]pyrazole-2,4-diium bis(tetrakis(3,5-bis(trifluoromethyl)phenyl)borate) • Et_2O (**7f**) as the XB-donor.*
